# CopyDetective: Detection threshold–aware copy number variant calling in whole-exome sequencing data

**DOI:** 10.1093/gigascience/giaa118

**Published:** 2020-11-02

**Authors:** Sarah Sandmann, Marius Wöste, Aniek O de Graaf, Birgit Burkhardt, Joop H Jansen, Martin Dugas

**Affiliations:** Institute of Medical Informatics, University of Münster, Albert-Schweitzer-Campus 1, Building A11, Münster 48149, Germany; Institute of Medical Informatics, University of Münster, Albert-Schweitzer-Campus 1, Building A11, Münster 48149, Germany; Laboratory Hematology, RadboudUMC, Geert Grooteplein Zuid 10, Nijmegen 6525 GA, Netherlands; Paediatric Hematology & Oncology, University Hospital Münster, Albert-Schweitzer-Campus 1, Building A1, Münster 48149, Germany; Laboratory Hematology, RadboudUMC, Geert Grooteplein Zuid 10, Nijmegen 6525 GA, Netherlands; Institute of Medical Informatics, University of Münster, Albert-Schweitzer-Campus 1, Building A11, Münster 48149, Germany

**Keywords:** copy number variant, polymorphism, cell fraction

## Abstract

**Background:**

Copy number variants (CNVs) are known to play an important role in the development and progression of several diseases. However, detection of CNVs with whole-exome sequencing (WES) experiments is challenging. Usually, additional experiments have to be performed.

**Findings:**

We developed a novel algorithm for somatic CNV calling in matched WES data called “CopyDetective". Different from other approaches, CNV calling with CopyDetective consists of a 2-step procedure: first, quality analysis is performed, determining individual detection thresholds for every sample. Second, actual CNV calling on the basis of the previously determined thresholds is performed. Our algorithm evaluates the change in variant allele frequency of polymorphisms and reports the fraction of affected cells for every CNV. Analyzing 4 WES data sets (*n* = 100) we observed superior performance of CopyDetective compared with ExomeCNV, VarScan2, ControlFREEC, ExomeDepth, and CNV-seq.

**Conclusions:**

Individual detection thresholds reveal that not every WES data set is equally apt for CNV calling. Initial quality analyses, determining individual detection thresholds—as realized by CopyDetective—can and should be performed prior to actual variant calling.

## Background

In recent years, next-generation sequencing (NGS) has found its way to clinical routine [1]. With the sequencing costs still getting cheaper—currently working on the “$100 genome” [2]—whole-exome sequencing (WES) and whole-genome sequencing (WGS) are performed for an increasing number of patients to improve their diagnosis, prognosis, and therapy by the help of personalized medicine [3,4].

Despite continuously decreasing costs for experiments, it is desirable to keep the number of necessary genetic experiments to a minimum—not least because of limited tumor material [5,6]. Thus, it would be most practical if there were valid algorithms to determine single-nucleotide variants (SNVs), short insertions and deletions (indels), structural variants (SVs), and copy number variants (CNVs) by just a single NGS experiment.

Although there still remain challenges to be addressed, relatively short mutations, such as SNVs and indels, can already be determined reliably [7, 8]. In contrast to this, large mutations such as CNVs still impose a major challenge [9].

Numerous algorithms, all following different approaches, exist for calling CNVs in WES data. While some concentrate on normalizing coverage, e.g., VarScan [10], others analyze single-nucleotide polymorphisms (SNPs) and coverage information similar to SNP arrays, e.g., ExomeCNV [11]. Some algorithms require matched control samples, while others do not require any controls. However, all of these algorithms usually are hindered by low precision and low recall [9], which raises the question of whether NGS data from WES experiments are after all suitable to determine valid CNV calls. Or—to make this question more specific—whether every data set is equally apt to determine every kind of CNV, independent of the number of base pairs or fraction of cells affected by the mutation. Especially in the field of cancer research this is highly relevant, specifically regarding cancer cell fractions and clonal evolution.

Considering SNV and indel calling in NGS data, it is obvious that every data set’s characteristics define its individual detection thresholds. An essential characteristic is coverage. If data are sequenced with only 10× coverage, they are not apt to detect mutations at allelic frequencies of 5% because only 0.5 reads are expected to carry the mutation. When calling CNVs in NGS data, it is only consistent to assume that comparable detection thresholds exist.

We present a novel algorithm, performing detection threshold–aware CNV calling in WES data: CopyDetective [12]. Prior to determining the actual CNVs, CopyDetective addresses the data quality of every sample. We consider (i) coverage of the case sample, (ii) coverage of the matching control sample, (iii) CNV length, and (iv) CNV value with respect to the fraction of affected cells. For every sample, individual detection thresholds are determined. These thresholds define the minimum cell fraction (CF) and the minimum CNV length that is still detectable at a given sensitivity.

Subsequently, CopyDetective analyzes data according to these thresholds. Comparing a case sample to its matching control sample, coverage and SNP information are evaluated to identify regions of significant difference. CopyDetective reports merged and filtered CNVs along with additional information on the calls, e.g., quality values and information on the estimated CF.

Analyzing 4 real WES data sets (*n* = 100) the performance of our novel approach is evaluated and compared with that of 5 established approaches for CNV calling in WES data: ExomeCNV [11], VarScan2 [10], ExomeDepth [13], Control-FREEC [14], and CNV-seq [15].

## Methods

### Data sets analyzed

We analyze 4 data sets, covering real data from *n* = 100 samples. An overview of the different data sets and the available samples can be found in Table 1.

**Table 1:** Overview of the samples analyzed with CopyDetective

Data set	Disease	Samples	Mean coverage (×)		Coverage ≥1× (%)		Coverage ≥10× (%)		Heterozygous SNPs
			Germline	Tumor	Germline	Tumor	Germline	Tumor	
1	MDS	47	114.90	119.71	99.28	99.07	97.29	97.04	10,543
2	BL								
	Primary	10	44.21	277.28	97.73	98.41	89.66	97.38	11,884
	Relapse	5	44.00	298.79	97.70	98.41	89.44	97.75	11,665
3	T-LBL								
	Primary	15	60.25	190.46	94.62	98.41	84.23	96.70	9,659
	Relapse	5	57.60	290.15	90.98	98.52	75.41	97.97	7,893
4	NMZL	18	41.63	41.24	99.08	99.10	78.61	80.71	9,341

The first set covers 47 samples from 11 patients with myelodysplastic syndromes (MDS; sequencing data published at the NCBI SRA PRJNA355124). Data from all patients have been sequenced 2–8 times. CNV-calling results based on CytoScan HD Array (Affymetrix), containing information on deletions, duplications, and loss of heterozygosity (LOH), have been published. Additionally, information on clonal evolution of all patients has been published by da Silva-Coelho et al. [16]. Thus, CNV value can be considered with respect to CF. For example, a simple duplication leads to CNV value = 3. However, if the mutation is present in only 60% of the cells, the CNV value over all cells would be 2.6.

The second set covers 15 samples from 10 patients with Burkitt lymphoma (BL; sequencing data published at the NCBI Sequence Read Archive PRJNA561490). Data from 5 of 10 patients have been sequenced twice—at the point of primary and relapse. The remaining 5 patients did not experience relapse and only 1 tumor sample (primary) is available. CNV-calling results (deletions, duplications, and LOH) are based on SNP arrays (Infinium OmniExpressExome-8v1.3kit; using Illumina GenomeStudio 2.0 [GenomeStudio, RRID:SCR_010973] and cnvPartition v3.2.0 [CNVPartition, RRID:SCR_010925], minimum 100 probes for 1 call, for analysis) [17]. Additionally, clonal evolution was reconstructed for all patients. Again, CNV value with respect to CF can be considered.

The third set covers 20 samples from 15 patients with T-lymphoblastic lymphoma (T-LBL; sequencing data published at the EMBL-EBI European Nucleotide Archive PRJEB36436). Data from 5 of 15 patients have been sequenced twice—at the point of primary and relapse. Because the remaining 10 patients did not experience relapse, only 1 tumor sample (primary) is available. CNV-calling results (deletions, duplications, and LOH) are based on SNP arrays (InfiniumOmni2-5Exome-8; using Illumina GenomeStudio 2.0 [GenomeStudio, RRID:SCR_010973] and cnvPartition v3.2.0 [CNVPartition, RRID:SCR_010925], minimum 100 probes for 1 call [18]; data published at Array Express E-MTAB-8763; [19]).

The fourth data set covers 18 samples from patients with nodal marginal zone lymphoma (NMZL; sequencing data published at the NCBI SRA PRJNA285732 [20]). CNV data have been deposited in Gene Expression Omnibus (accession No. GSE68078; CytoScan HD Array; Affymetrix). Analysis of the CNV data was performed using Rawcopy [21]. Raw calls with a missing allelic imbalance or an imbalance <0.2 were removed. The remaining calls were merged if they were located close to each other (<20 Mb) and characterized by a similar log*R*-ratio (2^log*R*^ < 0.2). The resulting CNV calls were classified as deletions if CNV value ≤1.9 and as duplications if the CNV value ≥2.1. The remaining calls were classified as LOH.

For all samples, detailed information on data quality can be found in Supplementary Tables S1–S4.

### CopyDetective

**Figure 1:**
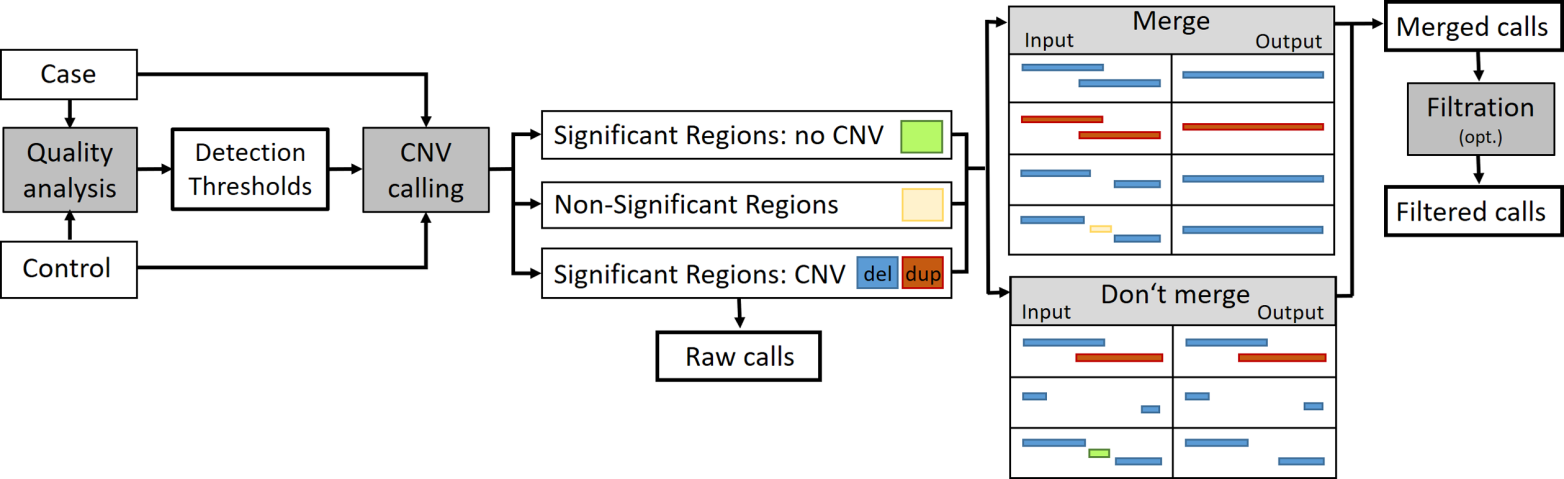
Overview of the analysis performed by CopyDetective. The analysis can be separated into 4 major steps: (i) Quality analysis: the detection thresholds for CNV calling are estimated. (ii) CNV calling: significant regions with and without CNV are determined. (iii) Merging of overlapping and adjacent regions with CNV. (iv) Optional filtration.

CopyDetective is a novel algorithm for calling somatic CNVs in matched WES samples, automatically determining and evaluating individual detection thresholds for every sample. The analysis with CopyDetective can be separated into 4 major steps: (i) quality analysis, (ii) CNV calling, (iii) merging, and optional (iv) filtration. An overview of the analysis is provided in Fig. 1.

#### Quality analysis

**Figure 2:**
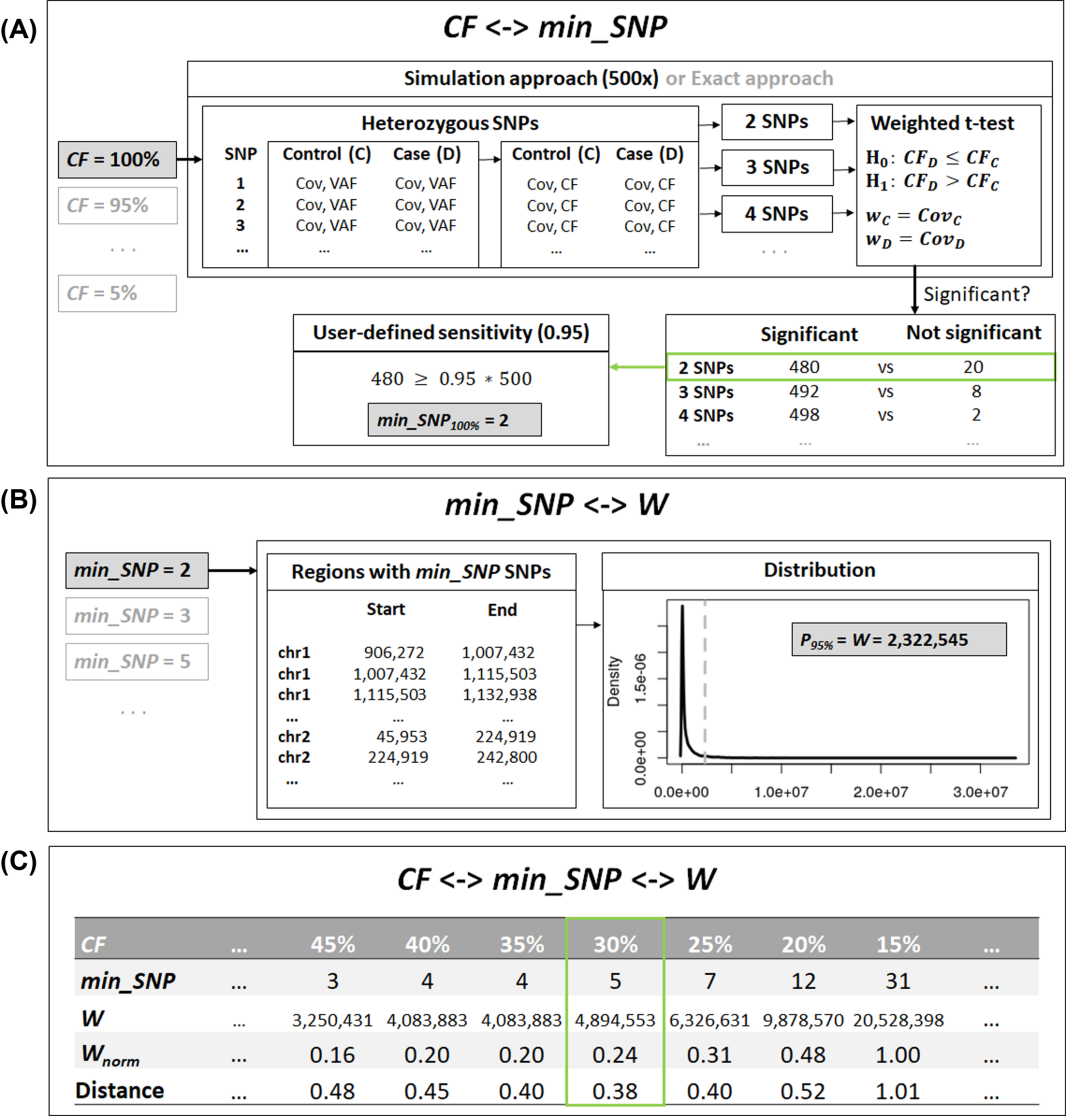
Overview of the quality analysis performed by CopyDetective. (A) A connection between CF and min_SNP is established to reach user-defined sensitivity. Following the simulation approach (default: 500×; alternative: exact approach), heterozygous SNPs are simulated. An increasing number of SNPs is evaluated, applying a weighted *t*-test. If a significant result is observed in ≥95% of the cases, min_SNP has been found. (B) For every relevant number of SNPs, the location of regions with min_SNP polymorphisms is determined. Based on the locations, the distribution of the region lengths is determined and $P_{95\%}$, i.e., *W* is calculated. (C) A connection between CF and *W* has been established. The optimal detection thresholds (by default: optimal trade-off between low CF and small window W) are determined. CF: cell fraction; min_SNP: minimum number of SNPs; Cov: coverage; VAF: variant allele frequency; $P_{95\%}$: 95% percentile; *W*: window size; *W*_norm_: normalized window size.

Different from other CNV-calling algorithms, an initial analysis of data quality is automatically performed by CopyDetective to determine individual detection thresholds for every sample. These thresholds include the minimum CFs for deletions and duplications, CF_Del_ and CF_Dup_, and the minimum CNV lengths, *W*_Del_ and *W*_Dup_. An overview of CopyDetective’s quality analysis, which is performed for both deletions and duplications, is provided in Fig. 2.

Quality analysis itself is split into 3 steps: first, CFs are considered (see Fig. 2A). Our analysis is based on the actual coverage of all heterozygous polymorphisms detected in a matching case-control pair. Analyses show that coverage distribution can be approximated by a log-normal distribution (see exemplary coverage distribution of patient MDS_01 germline in Supplementary Fig. S1). We evaluate all CFs in a user-defined range (default: 5–100% with steps of 5%; smaller step sizes are possible) for deletions and duplications separately. The user can choose between a simulation approach (default) and an exact approach. For the simulation approach, an artificial case-control pair is considered. To keep run-time low, we do not simulate the actual reads. Instead, just the coverage and variant allele frequency (VAF) of heterozygous polymorphisms are simulated (default: 1–100 SNPs). In the control sample, the expected value for VAF is 50%. In the case sample, VAF is dependent on CF: if CF = 100%, the expected value for VAF is 0% for deletions and 33% for duplications (100% and 67% are equally valid expected values; for reasons of simplicity we always work with expected VAFs <50% because CopyDetective automatically transforms all VAFs to CFs and performs all calculations on CF level only).

For the exact approach, all called heterozygous polymorphisms in the control sample are considered. Coverage for both the control and the case sample are given and do not have to be simulated. VAF for the control sample can either be based on the available data or be simulated. VAF for the case sample is always simulated on the basis of CF, just like in the case of the simulation approach.

To identify CNVs, we apply a weighted *t*-test. It is investigated whether a significant difference between case and control, evaluating the CFs of an increasing number of simulated polymorphisms, can be observed. By repeating this analysis (default: 500 times; or for all SNPs following the exact approach), we can estimate the lowest number of polymorphisms (min_SNP) that have to be evaluated to reach user-defined sensitivity (default: sens ≥ 0.95).

In the second quality analysis step, we establish a connection between min_SNP and window size *W* (see Fig. 2B). Polymorphisms are not evenly distributed across the genome. Instead, some regions show a much higher polymorphism density than others. Additionally, CopyDetective evaluates WES and not WGS data. Thus, to evaluate, e.g., 3 polymorphisms, it can be sufficient to analyze a very short region, or it might be necessary to consider a much larger one. We base our analysis on all SNPs detected in the control sample. For all relevant numbers of heterozygous SNPs, i.e., all values of min_SNP, we determine the positions and lengths of the corresponding regions. For example, region chr1:906,272–1,007,432 covers 2 heterozygous SNPs. Subsequently, the distribution of the region lengths is determined and the 95th percentile ($P_{95\%}$) is calculated. If, for example, the 95th percentile for 2 polymorphisms is 2,322,545 bp, we can expect that 95% of all genetic regions of 2,322,545 bp contain ≥2 polymorphisms. The 95th percentile is referred to as window size *W* within the framework of detection thresholds.

In the third step, the connection between CF and *W* via min_SNP is summed up and the actual detection thresholds are determined (see Fig. 2C). CopyDetective allows the user to force CNV calling with the minimum possible CF or the minimum possible window size. However, by default, we are aiming at optimizing both parameters: we normalize window size (*W*_norm_) and minimize the distance between CF and *W*_norm_ (for details see Supplementary Fig. S2). The cell fraction CF and window size *W* at minimum distance represent the detection thresholds for subsequent CNV calling.

Note that the quality analysis depicted in Fig. 2 is performed for both deletions and duplications. Thus, 4 detection thresholds are determined: CF_Del_, *W*_Del_, CF_Dup_, and *W*_Dup_.

#### CNV calling

Once the thresholds CF_Del_, *W*_Del_, CF_Dup_, and *W*_Dup_ have been estimated, as described in the quality analysis step, actual CNV calling is performed. CNV calling with CopyDetective is based on the analysis of VAFs, comparing heterozygous polymorphisms in matching case-control samples. Coverage is considered by determining 99% confidence intervals (CI_0.99_) for the VAFs.

It can be assumed that a heterozygous polymorphism is present at VAF = 50% in control samples and—if not affected by CNV—also in case samples. Deviations from this expected frequency may be observed, resulting from low coverage. However, the CI_0.99_ should cover the true VAF of 50% in 99 of 100 cases. Therefore, we only evaluate polymorphisms that fulfill this criterion.

In case samples, deviations from the expected VAF of 50% can, once again, result from either low coverage or presence of a CNV. Thus, a polymorphism with VAF = 67% can indicate a 1-fold duplication present in 100% of the cells. However, the observed VAF can also be explained by a 1-fold deletion present in 50% of the cells. If the CI_0.99_ of a polymorphism’s VAF covers either 33% or 67%, we assume that it can be explained by either a duplication or a deletion. If this is not the case, we only consider deletions. Copy numbers <1 and >3 are currently not taken into account.

CopyDetective identifies regions of significant difference comparing 1 case sample to its matching control. To improve direct interpretability of the results, we decided to work with CFs instead of VAFs. Thus, prior to actual testing, the observed VAFs are transferred to CF level, considering deletions and duplications. Similarly, the CI_0.99_ are determined for the CFs. Note that the CFs for all heterozygous polymorphisms in the control samples are expected to be zero but, in reality, show certain variation (for details on the relation between CF and VAF see Supplementary Figs S3 and S4).

The actual test we perform to identify regions of significant difference is a weighted *t*-test (2-sample, 1-tailed, α-adjusted according to Bonferroni correction: α = 0.05/4 = 0.0125). For deletions, a sliding window of size *W*_Del_ with all its covered SNPs is analyzed:

(1) \begin{eqnarray*}
\mathrm{Del:}\qquad H_0: \mathrm{CF}_{\mathrm{DelD}}\le \mathrm{CF}_{\mathrm{DelC}}\quad H_1: \mathrm{CF}_{\mathrm{DelD}} > \mathrm{CF}_{\mathrm{DelC}}
\end{eqnarray*}

 

(2) \begin{eqnarray*}
\text{No del:}\quad H_0: \mathrm{CF}_{\mathrm{DelD}}\ge \mathrm{CF}_{\mathrm{DelC}}\quad H_1: \mathrm{CF}_{\mathrm{DelD}} < \mathrm{CF}_{\mathrm{DelC}}
\end{eqnarray*}

CF_DelD_ is defined as the fraction of cells containing a deletion in the case sample (“D" for disease). CF_DelC_ is defined as the fraction of cells containing a deletion in the control sample (“C" for control). If a true deletion is present in the tumor sample, we expect CF_DelD_ to be significantly larger compared with CF_DelC_.

We expect that CF_DelC_ = 0. However, in reality, this is usually not the case. If significantly more cells with a deletion are detected in the control sample compared with the case sample (CF_DelD_ < CF_DelC_), this result indicates that no deletion is likely to be present in the case sample.

Similar to the analysis of deletions, duplications are considered by evaluating a sliding window of size *W*_Dup_ with all its covered SNPs:

(3) \begin{eqnarray*}
\text{Dup:}\quad H_0: \mathrm{CF}_{\mathrm{DupD}}\le \mathrm{CF}_{\mathrm{DupC}}\quad H_1: \mathrm{CF}_{\mathrm{DupD}} > \mathrm{CF}_{\mathrm{DupC}}
\end{eqnarray*}

 

(4) \begin{eqnarray*}
\text{No dup:}\quad H_0: \mathrm{CF}_{\mathrm{DupD}}\ge \mathrm{CF}_{\mathrm{DupC}}\quad H_1: \mathrm{CF}_{\mathrm{DupD}} < \mathrm{CF}_{\mathrm{DupC}}
\end{eqnarray*}

Instead of an ordinary *t*-test, we decided to apply a weighted *t*-test to account for the influence of coverage on the estimated cell fractions: if an evaluated polymorphism is characterized by low coverage—in either 1 or both, case and control—an observed difference between CF_DelD_ and CF_DelC_ (or between CF_DupD_ and CF_DupC_) might not result from an actual deletion but just occur at random. Thus, a decreased weight should be assigned to the low-coverage sample(s). In contrast, if an evaluated polymorphism is characterized by high coverage, any observed difference between CF_DelD_ and CF_DelC_ is likely to result from an actual deletion. Thus, an increased weight should be assigned. We define the weights for deletions (case: *w*_DelD_; control: *w*_DelC_) and duplications (case: *w*_DupD_; control: *w*_DupC_) as follows:

(5) \begin{eqnarray*}
\text{Del:}\quad w_{\mathrm{DelD}}=\mathrm{Cov}_{\mathrm{DelD}}\quad w_{\mathrm{DelC}}=\mathrm{Cov}_{\mathrm{DelC}}, \end{eqnarray*}

 

(6) \begin{eqnarray*}
\text{Dup:}\quad w_{\mathrm{DupD}}=\mathrm{Cov}_{\mathrm{DupD}}\quad w_{\mathrm{DupC}}=\mathrm{Cov}_{\mathrm{DupC}}. \end{eqnarray*}

To determine a list of raw CNV calls for every sample, we exclude non-significant regions as well as significant regions containing no CNV. Furthermore, regions with an estimated CF below the thresholds CF_Del_ and CF_Dup_ (−5% to account for variation of the estimate) are excluded.

It should be noted that the basis of our approach—the list of polymorphisms—is generated using VarDict [22] (for details on variant calling see Additional File 1, Section 1.3.2).

#### Merging

**Figure 3:**
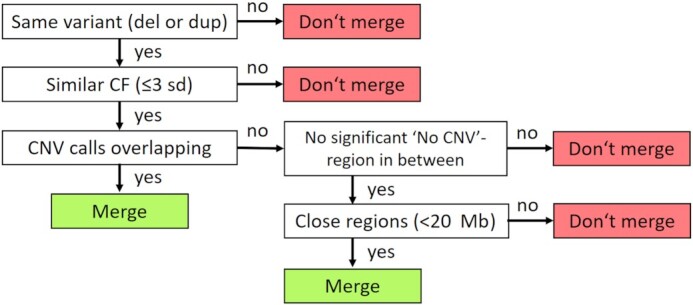
Decision tree for the merging process. Two CNV calls are merged according to the displayed merging scheme. Defining close regions as being separated by <20 Mb allows for merging of 2 regions separated by a centromere, which is ≤7.4 Mb, and for detecting monosomy or trisomy of the smallest chromosomes by only 2 significant regions. sd: standard deviations.

Raw CNV calls that are reported as being overlapping or located in close vicinity are likely corresponding to 1 event. Thus, merging of the raw calls is performed. The merging scheme is visualized in Fig. 3.

Two CNV calls are merged if the same variant is reported (deletion or duplication), CF is similar (≤3 standard deviations [sd]), and the calls are overlapping. If two CNV calls are not overlapping but no significant “no CNV" region is located in between and the regions are close (<20 Mb), they are likewise merged.

Note that for estimating the CFs of the merged regions, all significant SNPs are re-evaluated. This can, in some rare cases, lead to a merged region with an overall estimated CF below the actual detection thresholds. However, these regions always contain ≥2 raw CNV calls with CFs above the detection thresholds.

#### Filtration (optional)

Optionally, the merged results can be filtered on the basis of the CNV call quality. Depending on the data being analyzed, it can be useful to consider the merged calls directly. However, we recommend filtration of low-quality calls.

### Comparison to common approaches

Over recent years, several review articles have been published, considering tools available for CNV calling in NGS data [9,23,24]. To evaluate performance of our novel CNV-calling algorithm, we compare it with 5 common CNV-calling tools for WES data: ExomeCNV (ExomeCNV, RRID:SCR_010815) [11], VarScan2 (VARSCAN, RRID:SCR_006849) [10], ExomeDepth (ExomeDepth, RRID:SCR_002663) [13], Control-FREEC (Control-FREEC, RRID:SCR_010822) [14], and CNV-seq (CNV-seq, RRID:SCR_013357) [15].

ExomeCNV uses read depth and B-allele frequencies (BAF) from matched WES data to detect deletions, duplications, and LOH. It is frequently used for benchmarking [23]. We analyze the CNV calls reported in <Sample>.cnv.txt. The copy number reported in column copy.number is evaluated.

VarScan2 analyzes normalized read depth in matched WES samples to detect deletions and duplications. For every region, num.mark and seg.mean are reported. We exclude all variants with num.mark <10. If seg.mean ≥0.25, the variant is considered a duplication. If seg.mean ≤−0.25, the variant is considered a deletion. All variants with −0.25 < seg.mean < 0.25 are discarded.

Control-FREEC analyzes copy number and BAF profiles. Matched control samples are evaluated to distinguish germline variants from somatic ones. Information on subclonal gains and losses is reported and additionally evaluated if biological truth contains information on clonal composition of the samples. In addition to the standard Control-FREEC pipeline, we applied the additional script “assess_significance.R” [25]. CNV calls with a reported *P* value >0.05 are excluded. We consider both WilcoxonRankSumPvalue (WR) and KolmogorovSmirnovPvalue (KS). The copynumber, considering deletions, duplications, and LOH, reported in column “copy number" is evaluated.

ExomeDepth applies a β-binomial model to a set of exons. Normally, the tool requires multiple samples as input. The idea is that each exome is automatically compared to the exome featuring best correlation. However, because for all samples in our data sets matched controls are available, we assume that the matching control is always the best exome to be used for comparison. The copy number, considering deletions and duplications, reported in column “type" is evaluated.

Additionally, we consider CNV-seq. The tool has not been specifically designed for WES data. However, the general approach is similar to our novel approach CopyDetective: a sliding window is evaluated. The window size is defined by data quality, i.e., in this case coverage. Copy number ratios, as well as confidence values, are determined. However, different from CopyDetective, CNV calling with CNV-seq is solely based on coverage and not on BAFs. To process the raw output, we exclude all calls with missing values in columns “log2" and/or “cnv.size." Regions belonging to the same CNV (identifier in column “cnv") are merged. All merged calls with cnv.p.value >0.05 are excluded. The remaining calls are categorized as deletions if cnv.log2 <−0.25 and as duplications if cnv.log2 >0.25. All the other calls are categorized as LOH.

Details on the precise commands for executing CNV calling with the common approaches are provided in Additional File 1, Section 1.4. It should be noted that we tried to apply several additional tools on our data, e.g., THetA2 (THetA, RRID:SCR_001860) [26] or iCNV [27]. Information on all tools that we tested, and the reasons why they were excluded from further consideration, can be found in Additional File 1, Section 1.5.

## Results

We apply CopyDetective (simulation approach) on 4 sets of real data. Performance is compared to 5 established tools for CNV calling in WES data: ExomeCNV, VarScan2, ExomeDepth, Control-FREEC (WR and KS), and CNV-seq. Two samples from Data Set 2 (BL_03: P3 and R3) were excluded from analysis. Detailed analyses have shown that almost all validated CNVs appear to have been already present in the control sample, being either contamination or germline calls (for details see Supplementary Fig. S5). The results for the remaining 98 samples are summed up in Table 2. It should be noted that LOH was excluded from Data Set 4 because we do not have any information on the frequency of affected cells for these calls. All called CNVs of the type “LOH" were removed from the output of ExomeCNV, ControlFREEC, and CNV-seq. CNV calls reported by CopyDetective that were overlapping regions of validated LOH were equally removed. An analysis of Data Set 4 including LOH can be found in Additional File 1, Section 2.2.

**Table 2:** Performance of CopyDetective (raw, i.e., without optional final filtration, and filter, i.e., with default filtration threshold of 10.76) in comparison to 5 established approaches: ExomeCNV, VarScan2, ExomeDepth, Control-FREEC (WR and KS), and CNV-seq.

Tool and configuration	Data set	TP calls (+ false type)	FP calls	CNVs			Sens	PPV	F1
				Found	Missed	Detectable			
ExomeCNV	1	1,378 (+2,204)	215,865	49	6	55	0.89	0.01	0.01
	2	280 (+508)	13,213	32	15	47	0.68	0.02	0.04
	3	1,017 (+1,904)	66,686	40	8	48	0.83	0.02	0.03
	4	94 (+1)	2,064	24	64	88	0.27	0.04	0.08
VarScan2	1	119 (+126)	11,736	27	22	49	0.55	0.01	0.02
	2	106 (+185)	2,758	26	11	37	0.70	0.04	0.07
	3	65 (+16)	374	21	14	35	0.60	0.15	0.24
	4	30 (+0)	54	23	65	88	0.26	0.36	0.30
ExomeDepth	1	163 (+50)	8,074	13	36	49	0.27	0.02	0.04
	2	275 (+162)	2,042	25	12	37	0.68	0.12	0.20
	3	175 (+33)	2,047	20	15	35	0.57	0.08	0.14
	4	909 (+0)	375	32	56	88	0.36	0.71	0.48
ControlFREEC									
WR	1	7 (+6)	1,568	5	50	55	0.09	<0.01	0.01
	2	6 (+3)	278	3	44	47	0.06	0.02	0.03
	3	7 (+9)	654	6	42	48	0.13	0.01	0.02
	4	5 (+2)	231	5	83	88	0.06	0.02	0.03
KS	1	24 (+38)	7,261	12	43	55	0.22	<0.01	0.01
	2	16 (+11)	1,124	10	38	48	0.21	0.01	0.03
	3	16 (+11)	1,124	10	38	48	0.21	0.01	0.03
	4	32 (+4)	224	17	71	88	0.19	0.09	0.13
CNV-seq	1	25,690 (+27,757)	1,723,974	21	34	55	0.38	0.01	0.03
	2	3,016 (+1,885)	94,461	21	26	47	0.45	0.03	0.06
	3	6,628 (+4,518)	316,311	19	29	48	0.40	0.02	0.04
	4	786 (+1,125)	28,863	15	73	88	0.17	0.03	0.05
CopyDetective									
Raw	1	33 (+22)	729	18	1	19	0.95	0.04	0.08
	2	63* (+43)	176	34	3	37	0.92	0.26	0.41
	3	67 (+21)	212	40	1	41	0.98	0.24	0.39
	4	23 (+23)	399	19	0	19	1.00	0.05	0.10
Filter 10.76	1	25 (+15)	180	18	1	19	0.95	0.12	0.22
	2	50 (+31)	63	34	3	37	0.92	0.44	0.60
	3	60 (+18)	10	40	1	41	0.98	0.86	0.90
	4	22 (+16)	63	19	0	19	1.00	0.26	0.41

The table reports true-positive (TP) calls (in parentheses: reporting the number of additional true-positive calls if CNV type is not evaluated), false-positive (FP) calls, found, missed, and detectable CNVs, sensitivity (sens; only evaluating true-positive calls with correct CNV type), positive predictive value (PPV; only evaluating TP calls with correct CNV type), and the F1 score. * Sixty-four detected CNVs are overlapping true CNVs. However, as 1 called CNV is clearly shorter than the validated one and characterized by a remarkably low quality value, we assume that this overlap is occurring by just coincidence. Therefore, it is counted as “missed."

A CNV call is considered true positive if it shows any overlap with ≥1 validated CNV. If a CNV call is overlapping a true variant but features the “wrong” CNV type (e.g., a deletion is called, while the true CNV is a duplication), it is reported in parentheses, as true-positive call with false type. We evaluate sensitivity (sens), the positive predictive value (PPV), and the F1 score only considering the true-positive calls with correct CNV type (for details on how these statistics are calculated see Additional File 1, Section 1.6). For every variant-calling tool the number of detectable CNVs is defined. For VarScan and ExomeDepth the number of detectable CNVs is decreased because these 2 tools are not able to detect LOH. For CopyDetective the number of detectable CNVs is decreased on the basis of each sample's individual detection threshold for CNV length and CF (see Supplementary Tables S6–S9 for precise detection thresholds). Exemplary variant-calling results for 1 sample are visualized in Supplementary Fig. S7. A detailed overview of all missed and detected CNVs for each tool is provided in Supplementary Tables S10–S13. Detailed variant-calling results for CopyDetective are provided in Additional File 2. Results for CopyDetective using the exact approach can be found in Supplementary Fig. S8 and Table S14.

It can be observed that a majority of common variant-calling tools are characterized by low PPV. For ExomeCNV, ControlFREEC (both configurations, WR and KS), and CNV-seq PPV ranges between <0.01 and 0.09 for all data sets. Only in the cases of VarScan and ExomeDepth—both tools that are unable to detect LOH—can higher PPVs partly be observed. However, performance is highly data dependent (Set 1: PPV_VarScan2_ = 0.01, PPV_ExomeDepth_ = 0.02; Set 4: PPV_VarScan2_ = 0.36, PPV_ExomeDepth_ = 0.71). Considering our novel approach CopyDetective without filtration (configuration “raw"), PPV ranges between 0.04 and 0.26. Over all data sets, performance is comparable to the best common approach ExomeDepth (PPV 0.11 vs 0.12). If we apply filtration with our default threshold, values between 0.12 and 0.86 can be observed (over all data sets: 0.33).

Regarding sensitivity, huge differences among all approaches can be observed. While ControlFREEC and CNV-seq are characterized by low sensitivity (maximum: 0.45), much higher values can be observed for ExomeCNV (up to 0.89; on average 0.61). However, owing to low PPV, the overall performance considering the F1 score is, over all data sets, relatively poor. Similar to PPV, ExomeDepth features highly data-dependent performance with respect to sensitivity (ranging between 0.27 and 0.68). In contrast to this, CopyDetective is characterized by stable sensitivity. For both the raw and the filtered results, sensitivity ranges between 0.92 and 1.00. On average, sens = 0.96, which slightly exceeds our user-defined sensitivity of 0.95 when determining the detection thresholds.

**Figure 4:**
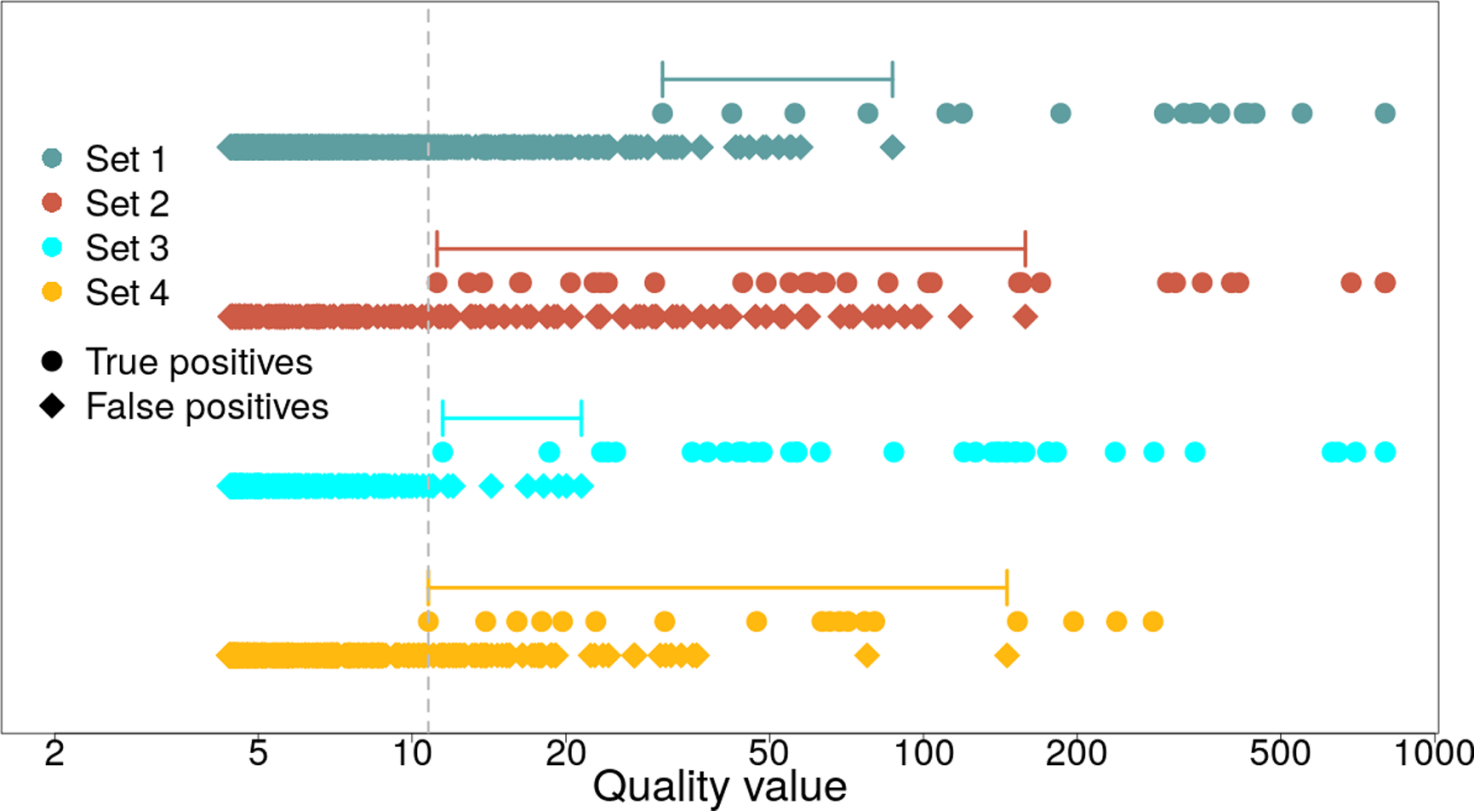
Quality values for true-positive and false-positive CNV calls reported by CopyDetective (evaluating the true CNV call with the highest quality value in case >1 CNV call overlaps a validated CNV). Ranges indicate the possible values of the quality threshold for each data set. Dashed gray line indicates the default filtration threshold of 10.76, excluding no true-positive calls.

Table 2 shows that the performance of CopyDetective is, with respect to PPV, data dependent, also including the influence of filtration. Supplementary Fig. S9 shows the relation between sensitivity and PPV in the context of an increasing quality threshold. A different development can be observed for the different data sets. Optimization of the F1 score would result in different optimal thresholds for each set (1: 56.05; 2: 16.22; 3: 18.52; 4: 36.36). Thus, the optimal quality threshold over all data sets is difficult to define. However, differences between the data sets are less prominent when the true calls’ quality values are considered (see Fig. 4).

For all data sets it can be observed that true CNV calls are characterized by higher quality values compared with false-positive calls. Combining all data sets, no true-positive call with a quality value <10.76 can be observed (see Supplementary Table S15). Three of 4 data sets share a similar threshold (2: 11.19; 3: 11.48; 4: 10.76). Therefore, we decided to select 10.76 as our default threshold for quality filtration applied in Step 4 of our algorithm.

## Discussion

CopyDetective is a novel tool for calling somatic CNVs in matched WES data. It has been developed for but is not limited to the analysis of cancer samples. Different from any other approach, CopyDetective performs initial quality analysis of every sample to estimate the individual detection thresholds, covering the minimum CNV length and the minimum cell fraction. These detection thresholds allow subsequent CNV calling with user-defined sensitivity (default: 0.95).

Considering the performance of our new approach, we observe high sensitivity regarding high- as well as low-coverage data. Over all data sets, CopyDetective outperforms all the other tools we considered, even without optional filtration of low-quality calls. Application of the quality filter results in further improvement of performance, especially with respect to PPV. Data indicate that a threshold of 10.76 can be used safely to exclude false-positive calls. Detailed additional analyses show that the coordinates of the CNVs, determined by CopyDetective, match the coordinates based on validation experiments (see Supplementary Figs S10 and S11 and Additional File 2). Furthermore, CFs estimated by CopyDetective match the cell fractions determined by other methods (e.g., fluorescence *in situ* hybridization; see Supplementary Fig. S12 and Additional File 2). However, it should be noted that the assumed true coordinates and cell fractions may differ from the actual true values. A precise determination of a CNV’s coordinates is usually not possible but can just be estimated. Furthermore, cell fractions that are based on clonal evolution analysis (Sets 1 and 2) may be biased by clustering. A CNV may be present in more or fewer cells compared to the other mutations in its cluster.

Yet, the fact that CopyDetective is able to estimate CFs is an important characteristic, especially with respect to clonal evolution. While allele frequencies of pathogenic mutations can easily be analyzed to determine the subclonal composition of a tumor, this should also be done when analyzing CNVs. However, most tools report only a copy number variant and its CNV value but not the fraction of cells affected by the mutation. To our knowledge, only 2 additional tools are able to estimate tumor purity in NGS data: CNAnorm [28] and THetA2 [26] (in addition, there are ASCAT [29] and ABSOLUTE [30]; however, these tools were designed for SNP array data). While superior performance of THetA2 has been reported by Oesper et al. [26], the tool failed on our data (see Additional File 1, Section 1.5).

It may seem astonishing that CNVs reported by CopyDetective match the validated CNVs, spread all over the genome, with respect to coordinates and CFs so well while just analyzing WES data. However, the main advantage of our approach lies in the analysis of a sliding window and the subsequent merging of windows located in close vicinity. This approach allows us to explore between 99.5% and 99.8% of the human genome (see Supplementary Fig. S6).

However, CopyDetective certainly has some limitations. We need a specific scenario—matching control samples—to evaluate changes in VAF for every polymorphism. CopyDetective's performance is dependent on the accuracy of polymorphism calling in the control sample. However, analyses of robustness have shown that CopyDetective is especially tolerant towards false-negative polymorphism calls (see Supplementary Figs S13 and S14 and Tables S16 and S17). Our approach is currently able to call only simple deletions or duplications. Depending on the cells affected by a CNV, ambiguous results are possible because, e.g., a deletion present in 50% of the cells can also be explained by a duplication present in 100% of the cells. LOH is always reported as a deletion by CopyDetective. However, a coverage indicator is reported. Analyses show that a true deletion is characterized by a negative coverage indicator (sens = 0.88), while LOH is characterized by a coverage indicator overlapping zero (sens = 0.87) (see Supplementary Fig. S15).

Currently, gonosomes are not evaluated by CopyDetective. CNVs on the Y chromosome cannot be detected because all polymorphisms are hemizygous (same is true for small CNVs just covering homozygous polymorphisms). However, our approach is expected to work for women's X chromosomes.

Depending on the quality of the input data provided, CopyDetective may not detect and report any small CNVs like focal CNVs, which are known to play an important role in cancer [31]. However, CopyDetective’s detection thresholds serve to ensure sufficient sensitivity of the CNV-calling results. By reporting the minimum CNV length and the minimum cell fraction, it becomes easy to decide whether the analyzed WES data are sufficient to detect the CNVs of interest or whether additional experiments have to be performed. When manually changing CopyDetective’s automatically determined detection thresholds to higher or lower values, we observe a decline in performance (see Supplementary Table S18). Higher, stricter thresholds decrease the number of detectable CNVs, no longer tapping the full potential of the data. On the contrary, lower thresholds lead to a major increase in false-positive calls.

## Conclusions

CopyDetective unites an established idea—evaluating the change in VAF of polymorphisms to detect CNVs—with a completely new aspect—determining individual detection thresholds for every sample. Thereby, CopyDetective shines a new light on CNV calling in WES data: individual detection thresholds reveal that not every data set is equally apt for CNV calling. The general idea of our algorithm—applying a 2-step procedure—can be combined with any other CNV-calling approach. Initial quality analyses, determining individual detection thresholds, can and should be performed prior to actual variant calling.

## Availability of Source Code and Requirements

Project name: CopyDetective [12]

Project home page:  https://github.com/sandmanns/CopyDetective

Operating system: Platform independent

Programming language: R

Other requirements: None

License: AGPL-3.0

bio.tools ID: biotools:copydetective

RRID:SCR_018909

## Availability of Supporting Data and Materials

Sequencing data are available at the NCBI SRA (PRJNA355124, PRJNA561490, and PRJNA285732), the EMBL-EBI European Nucleotide Archive (PRJEB36436), Array Express (E-MTAB-8763), and the Gene Expression Omnibus (GSE68078). All supporting data and materials are available in the *GigaScience* GigaDB database [32].

## Additional Files

Supplementary Figure S1. Exemplary coverage distribution.

Supplementary Figure S2. Exemplary relation between window size and cell fraction.

Supplementary Figure S3. General idea of CopyDetective.

Supplementary Figure S4. Expected change in VAF of polymorphisms in the presence of CNVs.

Supplementary Figure S5. Beta allele frequency.

Supplementary Figure S6. Relation bewteen the distance between two polymorphisms and the corresponding percentile.

Supplementary Figure S7. Exemplary variant calling output.

Supplementary Figure S8. Relation between detection thresholds (simulation vs exact approach).

Supplementary Figure S9. Relation between sensitivity and PPV.

Supplementary Figure S10. Overlap of CNVs reported by CopyDetective with validated CNVs.

Supplementary Figure S11. Relative deviation of the true start and end position from the called ones.

Supplementary Figure S12. Relation between estimated CF and true CF.

Supplementary Figure S13. Relation between detection thresholds (FN polymorphisms).

Supplementary Figure S14. Relation between detection thresholds (FP polymorphisms).

Supplementary Figure S15. Coverage indicator for true CNVs.

Supplementary Table S1. Sequencing data characteristics of data set 1.

Supplementary Table S2. Sequencing data characteristics of data set 2.

Supplementary Table S3. Sequencing data characteristics of data set 3.

Supplementary Table S4. Sequencing data characteristics of data set 4.

Supplementary Table S5. CNV calling results in data set 4, including LOH.

Supplementary Table S6. Detection thresholds of data set 1.

Supplementary Table S7. Detection thresholds of data set 2.

Supplementary Table S8. Detection thresholds of data set 3.

Supplementary Table S9. Detection thresholds of data set 4.

Supplementary Table S10. Detailed CNV calling results for data set 1.

Supplementary Table S11. Detailed CNV calling results for data set 2.

Supplementary Table S12. Detailed CNV calling results for data set 3.

Supplementary Table S13. Detailed CNV calling results for data set 4.

Supplementary Table S14. Performance of CopyDetective using the exact approach.

Supplementary Table S15. Lowest and highest quality values for TP vs FP CNV calls.

Supplementary Table S16. Performance of CopyDetective simulating FN polymorphisms.

Supplementary Table S17. Performance of CopyDetective simulating FP polymorphisms.

Supplementary Table S18. Performance of CopyDetective changing detection thresholds.

Supplementary Data S1. CNV calling output from CopyDetective, including raw and filtered calls for Data Sets 1–4.

## Abbreviations

BAF: B-allele frequencies; BL: Burkitt lymphoma; bp: base pairs; CF: cell fraction; CNV: copy number variant; EMBL-EBI: European Molecular Biology Laboratory European Bioinformatics Institute; FP: false positive; indel: insertion and deletion; KS: KolmogorovSmirnovPvalue; LOH: loss of heterozygosity; Mb: megabase pairs; MDS: myelodysplastic syndromes; NCBI: National Center for Biotechnology Information; NGS: next-generation sequencing; NMZL: nodal marginal zone lymphoma; PPV: positive predictive value; sens: sensitivity; sd: standard deviations: SNV: single-nucleotide variant; SNP: single-nucleotide polymorphism; SRA: Sequence Read Archive; SV: structural variant; T-LBL: T-lymphoblastic lymphoma; TP: true positive; VAF: variant allele frequency; WES: whole-exome sequencing; WGS: whole-genome sequencing; WR: WilcoxonRankSumPvalue.

## Ethical Approval

All patient material was collected and analyzed in accordance with the relevant ethical guidelines and regulations. Informed consent was obtained from all subjects.

## Competing Interests

The authors declare that they have no competing interests.

## Funding

This work has been supported by the EU grant Horizon2020 MDS-RIGHT (grant No. 634789), the DFG grant TU 298/5-1 (DFG Clinical Research Unit 326 Male Germ Cells: from Genes to Function), a grant from Deutsche Krebshilfe DKH (grant No. 111347), by Löwenkinder–Verein zur Unterstützung krebskranker Kinder e.V., and by Deutsche Kinderkrebsstiftung (support of the NHL-BFM Registry 2012; DKS349 2014.11 A/B).

## Authors' Contributions

S.S. developed the algorithm, performed data analyses, and wrote the manuscript. S.S. and M.W. performed analysis of validation data. A.O.d.G. and J.H.J. collected patient samples and coordinated targeted mutational and WES on the MDS cases. B.B. collected patient samples and coordinated WES on the T-LBL cases. M.D. reviewed development of the algorithm and reviewed the manuscript. All authors read, revised, and approved the final version of the manuscript.

## Supplementary Material

giaa118_GIGA-D-20-00138_Original_Submission

giaa118_GIGA-D-20-00138_Revision_1

giaa118_GIGA-D-20-00138_Revision_2

giaa118_Response_to_Reviewer_Comments_Original_Submission

giaa118_Response_to_Reviewer_Comments_Revision_1

giaa118_Reviewer_1_Report_Original_Submission Sheida Nabavi -- 7/3/2020 Reviewed

giaa118_Reviewer_1_Report_Revision_1 Sheida Nabavi -- 9/9/2020 Reviewed

giaa118_Reviewer_2_Report_Original_Submission Robert Nowak -- 7/5/2020 Reviewed

giaa118_Reviewer_2_Report_Revision_1 Robert Nowak -- 8/18/2020 Reviewed

giaa118_Supplemental_Files
