# Supplementary material for: CopyDetective: Detection threshold–aware copy number variant calling in whole-exome sequencing data
Source: Gigascience. 2020 Nov 2;9(11):giaa118. doi: 10.1093/gigascience/giaa118 (PMC7604644; doi:10.1093/gigascience/giaa118)
Supplement: giaa118_GIGA-D-20-00138_Revision_2 [file giaa118_giga-d-20-00138_revision_2.pdf]

GigaScience

CopyDetective: Detection Threshold Aware CNV Calling in WES Data

--Manuscript Draft--

**Manuscript Number:**	GIGA-D-20-00138R2
**Full Title:**	CopyDetective: Detection Threshold Aware CNV Calling in WES Data
**Article Type:**	Technical Note
**Funding Information:**	Horizon 2020 (634789) — Not applicable<br>Deutsche Forschungsgemeinschaft (TU 298/5-1) — Not applicable<br>Deutsche Krebshilfe (111347) — Prof. Dr. Dr. Birgit Burkhardt<br>Löwenkinder - Verein zur Unterstützung krebskranker Kinder e.V. — Prof. Dr. Dr. Birgit Burkhardt<br>Deutsche Kinderkrebsstiftung (DKS349 2014.11 A/B) — Prof. Dr. Dr. Birgit Burkhardt
**Abstract:**	Background: Copy number variants (CNVs) are known to play an important role in the development and progression of several diseases. However, detection of CNVs with whole-exome sequencing experiments is challenging. Usually, additional experiments have to be performed. Results: We developed a novel algorithm for somatic CNV calling in matched WES data called 'CopyDetective'. Different from other approaches, CNV calling with CopyDetective consists of a 2-step procedure: first, quality analysis is performed, determining individual detection thresholds for every sample. Second, actual CNV calling on the basis of the previously determined thresholds is performed. Our algorithm evaluates the change in variant allele frequency of polymorphisms and reports the fraction of affected cells for every CNV. Analyzing 4 WES data sets (n=100) we observe superior performance of CopyDetective compared to ExomeCNV, VarScan2, ControlFREEC, ExomeDepth and CNV-seq. Conclusions: Individual detection thresholds reveal that not every WES data set is equally apt for CNV calling. Initial quality analyses, determining individual detection thresholds - as it is realized by CopyDetective -, can and should be performed prior to actual variant calling.
**Corresponding Author:**	Sarah Sandmann<br>Westfälische Wilhelms-Universität Münster<br>Münster, GERMANY
**Corresponding Author Secondary Information:**	
**Corresponding Author's Institution:**	Westfälische Wilhelms-Universität Münster
**Corresponding Author's Secondary Institution:**	
**First Author:**	Sarah Sandmann
**First Author Secondary Information:**	
**Order of Authors:**	Sarah Sandmann<br>Marius Wöste<br>Aniek O de Graaf<br>Birgit Burkhardt<br>Joop H Jansen<br>Martin Dugas
**Order of Authors Secondary Information:**	
**Response to Reviewers:**	There were no more reviewer comments to be addressed.
**Additional Information:**	

Powered by Editorial Manager® and ProduXion Manager® from Aries Systems Corporation

Question	Response
Are you submitting this manuscript to a special series or article collection?	No
**Experimental design and statistics**<br><br>Full details of the experimental design and statistical methods used should be given in the Methods section, as detailed in our Minimum Standards Reporting Checklist. Information essential to interpreting the data presented should be made available in the figure legends.<br><br>Have you included all the information requested in your manuscript?	Yes
**Resources**<br><br>A description of all resources used, including antibodies, cell lines, animals and software tools, with enough information to allow them to be uniquely identified, should be included in the Methods section. Authors are strongly encouraged to cite Research Resource Identifiers (RRIDs) for antibodies, model organisms and tools, where possible.<br><br>Have you included the information requested as detailed in our Minimum Standards Reporting Checklist?	Yes
**Availability of data and materials**<br><br>All datasets and code on which the conclusions of the paper rely must be either included in your submission or deposited in publicly available repositories (where available and ethically appropriate), referencing such data using a unique identifier in the references and in the "Availability of Data and Materials" section of your manuscript.	No

Have you have met the above requirement as detailed in our Minimum Standards Reporting Checklist?	
If not, please give reasons for any omissions below.<br><br>as follow-up to "**Availability of data and materials**<br><br>All datasets and code on which the conclusions of the paper rely must be either included in your submission or deposited in publicly available repositories (where available and ethically appropriate), referencing such data using a unique identifier in the references and in the "Availability of Data and Materials" section of your manuscript.<br><br>Have you have met the above requirement as detailed in our Minimum Standards Reporting Checklist?<br><br>"	Sequencing data of data set number 3 (T-LBL) has already been uploaded to the EMBL-EBI European Nucleotide Archive (NGS data) and to Array Express (SNP array data). IDs are already available and referenced in the manuscript (PRJEB36436 and E-MTAB-8763). However, as the manuscript analyzing these data on a medical level is currently under revision, data has not yet been released.<br><br>As requested, we uploaded the medical manuscript as Supplementary Material. We would like to stress the fact that this manuscript has not been published yet and should therefore be treated strictly confidential. Thank you!<br>To share the sequencing data for set 3 (SNP array and WES), we uploaded it to our sftp-server. It is available via:<br>sftp://sftp.uni-muenster.de<br>User: copydetective<br>Pwd: ancient_issue_citrus_immunize_shown_rosy_marmalade_corner<br><br>All other data is publically available or avialable with the Supplementary Information.<br><br>With respect to code: following the Editorial Policies and Reporting Standards, we uploaded our software to github (https://github.com/sandmanns/CopyDetective). Additionally, it was archived using Zenodo (doi: 10.5281/zenodo.3859733).<br>Both, the link to github and the reference to the archived version are available with our manuscript.

Powered by Editorial Manager® and ProduXion Manager® from Aries Systems Corporation

Click here to access/download;LaTeX - Main Document (TeX file);main.tex

OXFORD (GIGA)^n SCIENCE

GigaScience, 2017, 1–10

doi: xx.xxxx/xxxx
Manuscript in Preparation
Technical Note

TECHNICAL NOTE

CopyDetective: Detection Threshold Aware CNV Calling in WES Data

Sarah Sandmann[1,*], Marius Wöste[1], Aniek O de Graaf[2], Birgit Burkhardt[3], Joop H Jansen[2] and Martin Dugas[1]

[1]Institute of Medical Informatics, University of Münster, Münster, 48149, Germany, and [2]Laboratory Hematology, RadboudUMC, Nijmegen, 6525 GA, Netherlands, and [3]Paediatric Hematology & Oncology, University Hospital Münster, Münster, 48149, Germany.

[*]sarah.sandmann@uni-muenster.de; +49 251 83 55270

ORCIDs:
Sarah Sandmann, 0000-0002-5011-0641; Marius Wöste, 0000-0003-4994-8380; Martin Dugas, 0000-0001-9740-0788.

Abstract

Background: Copy number variants (CNVs) are known to play an important role in the development and progression of several diseases. However, detection of CNVs with whole-exome sequencing experiments is challenging. Usually, additional experiments have to be performed. **Results:** We developed a novel algorithm for somatic CNV calling in matched WES data called 'CopyDetective'. Different from other approaches, CNV calling with CopyDetective consists of a 2-step procedure: first, quality analysis is performed, determining individual detection thresholds for every sample. Second, actual CNV calling on the basis of the previously determined thresholds is performed. Our algorithm evaluates the change in variant allele frequency of polymorphisms and reports the fraction of affected cells for every CNV. Analyzing four WES data sets (n=100) we observe superior performance of CopyDetective compared to ExomeCNV, VarScan2, ControlFREEC, ExomeDepth and CNV-seq. **Conclusions:** Individual detection thresholds reveal that not every WES data set is equally apt for CNV calling. Initial quality analyses, determining individual detection thresholds – as it is realized by CopyDetective –, can and should be performed prior to actual variant calling.

Key words: Copy number variant; polymorphism; cell fraction

Background

Over the last years, next-generation sequencing (NGS) has found its way to clinical routine [1]. With the sequencing costs still getting cheaper – currently working on the "$100 genome" [2] – whole-exome sequencing (WES) and whole-genome sequencing (WGS) are performed for an increasing number of patients to improve their diagnosis, prognosis and therapy by the help of personalized medicine [3, 4].

Despite continuously decreasing costs for experiments, it is desirable to keep the number of necessary genetic experiments to a minimum – not least because of limited tumor material [5, 6]. Thus, it would be most practical if there were valid algorithms to determine single-nucleotide variants (SNVs), short insertions and deletions (indels), structural variants (SVs) and copy number variants (CNVs) by just a single NGS-experiment.

Although there still remain challenges to be addressed, relatively short mutations – like SNVs and indels – can already be determined quite reliably [7, 8]. In contrast to this, large mutations like CNVs still impose a major challenge [9].

Numerous algorithms, all following different approaches, exist for calling CNVs in WES data. While some concentrate on normalizing coverage, e.g. VarScan [10], others analyze single-nucleotide polymorphisms (SNPs) and coverage information similar to SNP arrays, e.g. ExomeCNV [11]. Some algorithms require matched control samples, while others do not

Compiled on: September 24, 2020.
Draft manuscript prepared by the author.

Table 1. Overview of the samples analyzed with CopyDetective.

Data set	Disease		Samples	Mean coverage		Coverage ≥1x		Coverage ≥10x		Heterozygous
				Germline	Tumor	Germline	Tumor	Germline	Tumor	SNPs
1	MDS		47	114.90x	119.71x	99.28%	99.07%	97.29%	97.04%	10,543
2	BL	primary	10	44.21x	277.28x	97.73%	98.41%	89.66%	97.38%	11,884
		relapse	5	44.00x	298.79x	97.70%	98.41%	89.44%	97.75%	11,665
3	T-LBL	primary	15	60.25x	190.46x	94.62%	98.41%	84.23%	96.70%	9,659
		relapse	5	57.60x	290.15x	90.98%	98.52%	75.41%	97.97%	7,893
4	NMZL		18	41.63x	41.24x	99.08%	99.10%	78.61%	80.71%	9,341

require any controls. However, all of these algorithms usually suffer from low precision and low recall [9], which raises the question if NGS data from WES experiments is after all suitable to determine valid CNV calls. Or – to specify this question – if every data set is equally apt to determine every kind of CNV, independent of the number of base pairs or fraction of cells affected by the mutation. Especially in the field of cancer research this is highly relevant, specifically regarding cancer cell fractions (CCFs) and clonal evolution.

Considering SNV and indel calling in NGS data, it is obvious that every data set's characteristics define its individual detection thresholds. An essential characteristic is coverage. If data is sequenced with only 10x coverage, it is not apt to detect mutations at allelic frequencies of 5% as only 0.5 reads are expected to carry the mutation. When calling CNVs in NGS data, it is only consistent to assume that comparable detection thresholds exist.

We present a novel algorithm, performing detection threshold aware CNV calling in WES data: CopyDetective [12]. Prior to determining the actual CNVs, CopyDetective addresses data quality of every sample. We consider 1) coverage of the case sample, 2) coverage of the matching control sample, 3) CNV length and 4) CNV value with respect to the fraction of affected cells. For every sample, individual detection thresholds are determined. These thresholds define the minimum cell fraction (CF) and the minimum CNV length still being detectable at a given sensitivity.

Subsequently, CopyDetective analyzes data according to these thresholds. Comparing a case sample to its matching control sample, coverage and SNP information is evaluated to identify regions of significant difference. CopyDetective reports merged and filtered CNVs along with additional information on the calls, e.g. quality values and information on the estimated CF.

Analyzing four real WES data sets (n = 100) the performance of our novel approach is evaluated and compared to five established approaches for CNV calling in WES data: ExomeCNV [11], VarScan2 [10], ExomeDepth [13], Control-FREEC [14] and CNV-seq[15].

Methods

Data sets analyzed

We analyze four data sets, covering real data from n = 100 samples. An overview of the different data sets and the available samples can be found in Table 1.

The first set covers 47 samples from 11 patients with myelodysplastic syndromes (MDS; sequencing data published at the NCBI Sequence Read Archive PRJNA355124). All patients have been sequenced 2 to 8 times. CNV calling results based on CytoScan HD Array (Affymetrix) – containing information on deletions, duplications and loss of heterozygosity (LOH) – have been published. Additionally, information on clonal evolution of all patients has been published by da Silva et al. [16]. Thus, CNV value can be considered with respect to CF. For example: a simple duplication leads to CNV value= 3. However, if the mutation is just present in 60% of the cells, the CNV value over all cells would be 2.6.

The second set covers 15 samples from 10 patients with Burkitt lymphoma (BL; sequencing data published at the NCBI Sequence Read Archive PRJNA561490). Five out of 10 patients have been sequenced twice – at the point of primary and relapse. The remaining 5 patients did not relapse. Only one tumor sample (primary) is available. CNV calling results (deletions, duplications and LOH) are based on SNP arrays (Infinium OmniExpressExome-8v1.3kit; using Illumina GenomeStudio 2.0 (GenomeStudio, RRID:SCR_010973) and cnvPartition v3.2.0 (CNVPartition, RRID:SCR_010925), minimum 100 probes for one call, for analysis) [17]. Additionally, clonal evolution was reconstructed for all patients. Again, CNV value with respect to CF can be considered.

The third set covers 20 samples from 15 patients with T-lymphoblastic lymphoma (T-LBL; sequencing data published at the EMBL-EBI European Nucleotide Archive PRJEB36436). Five out of 15 patients have been sequenced twice – at the point of primary and relapse. As the remaining 10 patients did not relapse, only one tumor sample (primary) is available. CNV calling results (deletions, duplications and LOH) are based on SNP arrays (InfiniumOmni2-5Exome-8; using Illumina GenomeStudio 2.0 (GenomeStudio, RRID:SCR_010973) and cnvPartition v3.2.0 (CNVPartition, RRID:SCR_010925), minimum 100 probes for one call [18]; data published at Array Express E-MTAB-8763; manuscript under review).

The fourth data set covers 18 samples with nodal marginal zone lymphoma (NMZL; sequencing data published at the NCBI Sequence Read Archive PRJNA285732 [19]). CNV data has been deposited in Gene Expression Omnibus (accession number GSE68078; CytoScan HD Array; Affymetrix). Analysis of the CNV data was performed using Rawcopy [20]. Raw calls with a missing allelic imbalance, or an imbalance < 0.2 were removed. The remaining calls were merged if they were located close to each other (<20 Mbp) and characterized by a similar logR-ratio (2^{logR} <0.2). The resulting CNV calls were classified as deletions if CNV value≤1.9, and as duplications if the CNV value≥2.1. The remaining calls were classified as LOH.

For all samples, detailed information on data quality can be found in Additional file 1, section 1.1 (Tables S1-S4).

CopyDetective

CopyDetective is a novel algorithm for calling somatic CNVs in matched WES samples, automatically determining and evaluating individual detection thresholds for every sample. The analysis with CopyDetective can be separated into four major steps: 1) quality analysis, 2) CNV calling, 3) merging, and optional 4) filtration. An overview of the analysis is provided in

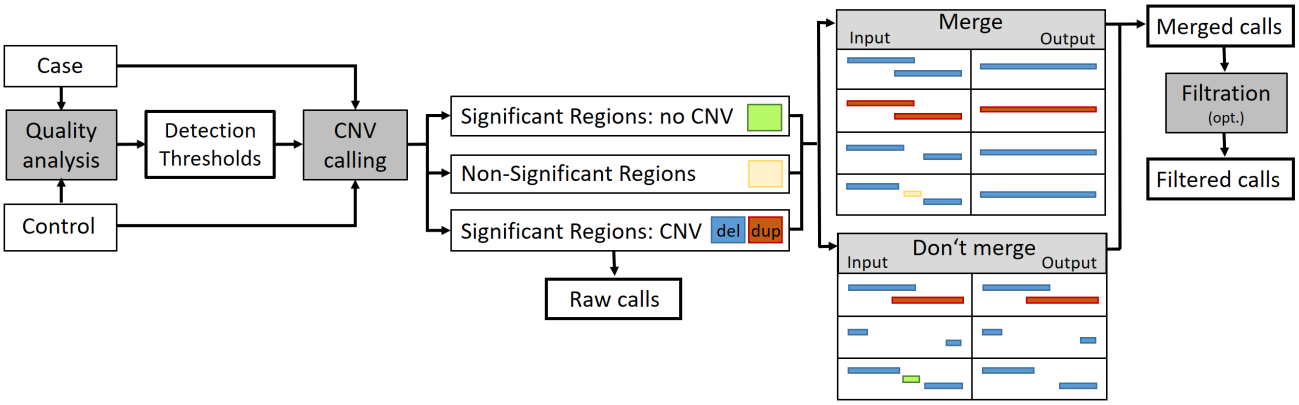

Figure 1. Overview of the analysis performed by CopyDetective. The analysis can be separated into four major steps: 1) Quality analysis: the detection thresholds for CNV calling are estimated. 2) CNV calling: significant regions with and without CNV are determined. 3) Merging of overlapping and adjacent regions with CNV. 4) Optional filtration.

Figure 1.

Quality analysis

Different from other CNV calling algorithms, an initial analysis of data quality is automatically performed by CopyDetective to determine individual detection thresholds for every sample. These thresholds include the minimum CFs for deletions and duplications – CF_{Del} and CF_{Dup} – and the minimum CNV lengths – W_{Del} and W_{Dup}. An overview of CopyDetective's quality analysis, which is performed for both deletions and duplications, is provided in Figure 2.

Quality analysis itself is split into three steps: First, CFs are considered (see Figure 2A). Our analysis is based on the actual coverage of all heterozygous polymorphisms detected in a matching case-control-pair. Analyses show that coverage distribution can be approximated by a log-normal distribution (see exemplary coverage distribution of patient MDS_01 germline in Additional file 1, section 1.2.1, Figure S1). We evaluate all CFs in a user-defined range (default: 5-100% with steps of 5%; smaller step sizes are possible) for deletions and duplications separately. The user can choose between a simulation approach (default) and an exact approach. For the simulation approach, an artificial case-control-pair is considered. To keep run-time low, we do not simulate the actual reads. Instead, just the coverage and variant allele frequency (VAF) of heterozygous polymorphisms are simulated (default: 1 to 100 SNPs). In the control sample, the expected value for VAF is 50%. In the case sample, VAF is dependent on CF: If CF = 100% the expected value for VAF is 0% for deletions and 33% for duplications (100% and 67% are equally valid expected values; for reasons of simplicity we always work with expected VAFs<50% as CopyDetective automatically transforms all VAFs to CFs and performs all calculations on CF-level only).

For the exact approach, all called heterozygous polymorphisms in the control sample are considered. Coverage for both the control and the case sample are given and do not have to be simulated. VAF for the control sample can either be based on the available data or it can be simulated. VAF for the case sample is always simulated based on CF, just like in case of the simulation approach.

To identify CNVs, we apply a weighted t-test. It is investigated whether a significant difference between case and control, evaluating the CFs of an increasing number of simulated polymorphisms, can be observed. By repeating this analysis (default: 500 times; or for all SNPs following the exact approach), we can estimate the lowest number of polymorphisms (min_SNP) that have to be evaluated to reach user-defined sensitivity (default: $sens \geq 0.95$).

In the second quality analysis step, we establish a connection between min_SNP and window size W (see Figure 2B). Polymorphisms are not evenly distributed across the genome. Instead, some regions show a much higher polymorphism density than others. Additionally, CopyDetective evaluates WES and not WGS data. Thus, to evaluate e.g. 3 polymorphisms, it can be sufficient to analyze a very short region, or it might be necessary to consider a much larger one. We base our analysis on all SNPs detected in the control sample. For all relevant numbers of heterozygous SNPs, i.e. all values of min_SNP, we determine the positions and lengths of the corresponding regions. For example, region chr1:906,272-1,007,432 covers 2 heterozygous SNPs. Subsequently, the distribution of the region lengths' is determined and the 95th percentile ($P_{95\%}$) is calculated. If, for example, the 95th percentile for 2 polymorphisms is 2,322,545 bp, we can expect that 95% of all genetic regions of 2,322,545 bp contain at least 2 polymorphisms. The 95th percentile is referred to as window size W within the framework of detection thresholds.

In the third step, the connection between CF and W via min_SNP is summed up and the actual detection thresholds are determined (see Figure 2C). CopyDetective allows the user to force CNV calling with the minimum possible CF or the minimum possible window size. However, by default, we are aiming at optimizing both parameters: we normalize window size (W_{norm}) and minimize the distance between CF and W_{norm} (for details see Additional file 1, section 1.2.2, Figure S2). The cell fraction CF and window size W at minimum distance represent the detection thresholds for subsequent CNV calling.

Note that the quality analysis depicted in Figure 2 is performed for both deletions and duplications. Thus, four detection thresholds are determined: CF_{Del}, W_{Del}, CF_{Dup} and W_{Dup}.

CNV calling

Once the thresholds CF_{Del}, W_{Del}, CF_{Dup} and W_{Dup} have been estimated, as described in the 'quality analysis'-step, actual CNV calling is performed. CNV calling with CopyDetective is based on the analysis of VAFs, comparing heterozygous polymorphisms in matching case-control samples. Coverage is considered by determining 99% confidence intervals ($CI_{0.99}$) for the VAFs.

It can be assumed that a heterozygous polymorphism is present at VAF=50% in control samples and – if not affected by CNV – also in case samples. Deviations from this expected frequency may be observed, resulting from low coverage. However, the $CI_{0.99}$ should cover the true VAF of 50% in 99 out of 100 cases. Therefore, we only evaluate polymorphisms that fulfill this criterion.

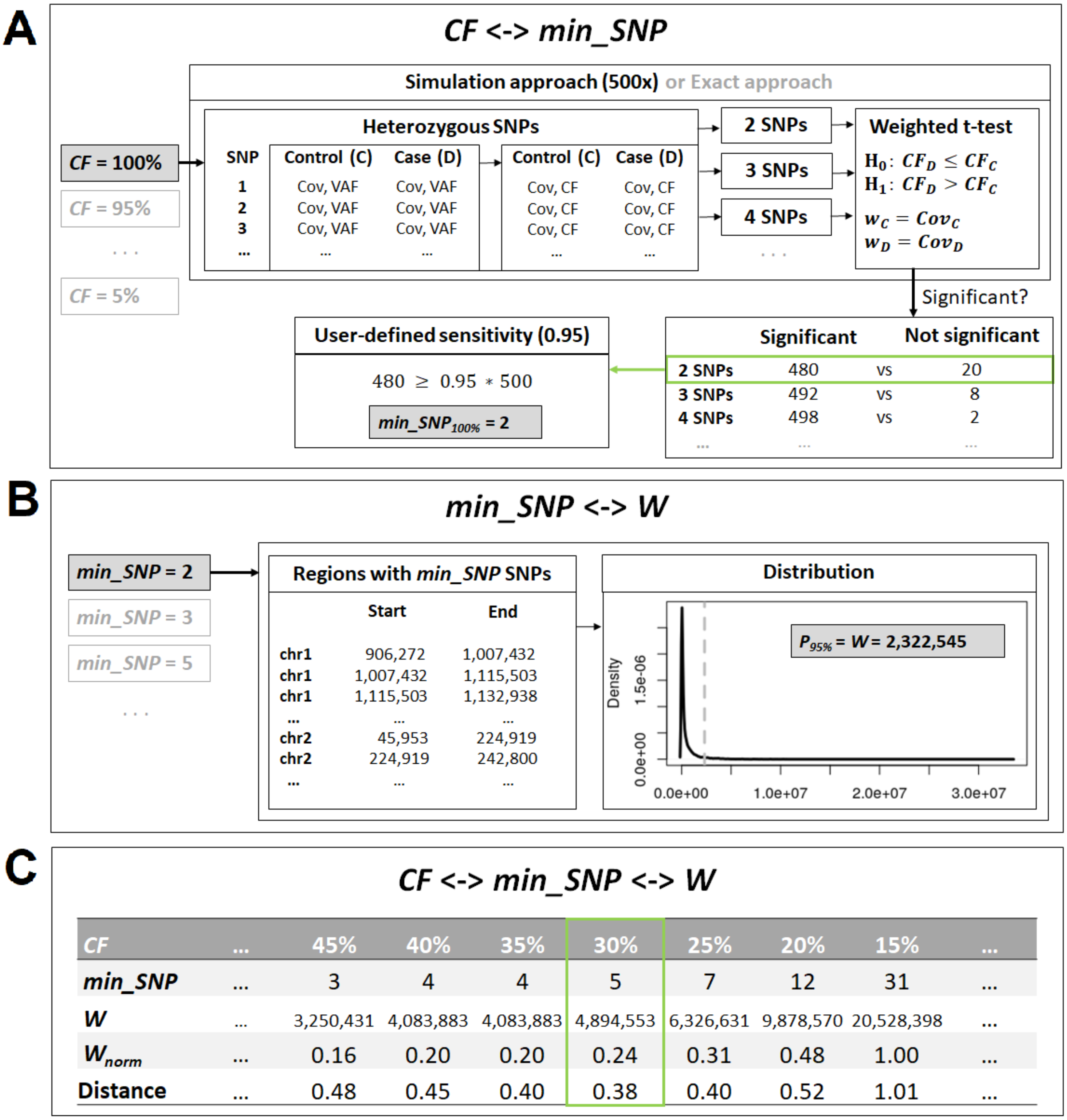

Figure 2. Overview of the quality analysis performed by CopyDetective. A) A connection between *CF* and *min_SNP* is established to reach user-defined sensitivity. Following the simulation approach (default: 500x; alternative: exact approach), heterozygous SNPs are simulated. An increasing number of SNPs is evaluated, applying a weighted t-test. If a significant result is observed in ≥95% of the cases, *min_SNP* has been found. B) For every relevant number of SNPs, the location of regions with *min_SNP* polymorphisms is determined. Based on the locations, the distribution of the region lengths' is determined and $P_{95\%}$, i.e. *W* is calculated. C) A connection between *CF* and *W* has been established. The optimal detection thresholds (by default: optimal trade-off between low CF and small window W) are determined. *CF* – cell fraction; *min_SNP* – minimum number of SNPs; Cov – coverage; VAF – variant allele frequency; $P_{95\%}$ – 95% percentile; *W* – window size; W_{norm} – normalized window size.

In case samples, deviations from the expected VAF of 50% can either – once again – result from low coverage or presence of a CNV. Thus, a polymorphism with VAF=67% can indicate a 1-fold duplication present in 100% of the cells. However, the observed VAF can also be explained by a 1-fold deletion present in 50% of the cells. If the $CI_{0.99}$ of a polymorphism's VAF covers either 33% or 67%, we assume that it can either be explained by a duplication or a deletion. If this is not the case, we only consider deletions. Copy numbers < 1 and > 3 are currently not taken into account.

CopyDetective identifies regions of significant difference comparing one case sample to its matching control. To improve direct interpretability of the results, we decided to work with CFs instead of VAFs. Thus, prior to actual testing, the observed VAFs are transferred to CF-level, considering deletions and duplications. Similarly, the $CI_{0.99}$ are determined for the CFs. Note that the CFs for all heterozygous polymorphisms in the control samples are expected to be zero, but – in reality – show certain variation (for details on the relation between CF and VAF see Additional file 1, section 1.3.1, Figures S3-S4).

The actual test we perform to identify regions of significant difference is a weighted t-test (2-sample, 1-tailed, α adjusted according to Bonferroni correction: $\alpha = 0.05/4 = 0.0125$). For deletions, a sliding window of size W_{Del} with all its covered SNPs is analyzed:

$$\text{Del:} \quad H_0 : CF_{DelD} \leq CF_{DelC} \quad H_1 : CF_{DelD} > CF_{DelC} \tag{1}$$

$$\text{No del:} \quad H_0 : CF_{DelD} \geq CF_{DelC} \quad H_1 : CF_{DelD} < CF_{DelC} \tag{2}$$

CF_{DelD} is defined as the fraction of cells containing a deletion in the case sample ('D' for disease). CF_{DelC} is defined as the fraction of cells containing a deletion in the control sample ('C' for control). If a true deletion is present in the tumor sample, we expect that CF_{DelD} is significantly larger compared to CF_{DelC}.

We expect that $CF_{DelC} = 0$. However, in reality, this is usually not the case. If significantly more cells with a deletion are detected in the control sample compared to the case sample ($CF_{DelD} < CF_{DelC}$), this result indicates that no deletion is likely to be present in the case sample.

Similar to the analysis of deletions, duplications are considered by evaluating a sliding window of size W_{Dup} with all its covered SNPs:

$$\text{Dup:} \quad H_0 : CF_{DupD} \leq CF_{DupC} \quad H_1 : CF_{DupD} > CF_{DupC} \tag{3}$$

$$\text{No dup:} \quad H_0 : CF_{DupD} \geq CF_{DupC} \quad H_1 : CF_{DupD} < CF_{DupC} \tag{4}$$

Instead of an ordinary t-test, we decided to apply a weighted t-test to account for the influence of coverage on the estimated cell fractions: If an evaluated polymorphism is characterized by low coverage – in either one or both, case and control – an observed difference between CF_{DelD} and CF_{DelC} (or between CF_{DupD} and CF_{DupC}) might not result from an actual deletion, but just occur at random. Thus, a decreased weight should be assigned to the low coverage sample(s). In contrast, if an evaluated polymorphism is characterized by high coverage, any observed difference between CF_{DelD} and CF_{DelC} is likely to result from an actual deletion. Thus, an increased weight should be assigned. We define the weights for deletions (case: w_{DelD}; control: w_{DelC}) and duplications (case: w_{DupD}; control: w_{DupC}) as follows:

$$\text{Del:} \quad w_{DelD} = Cov_{DelD} \quad w_{DelC} = Cov_{DelC} \tag{5}$$

$$\text{Dup:} \quad w_{DupD} = Cov_{DupD} \quad w_{DupC} = Cov_{DupC} \tag{6}$$

To determine a list of raw CNV calls for every sample, we exclude non-significant regions as well as significant regions containing no CNV. Furthermore, regions with an estimated CF below the thresholds CF_{Del} and CF_{Dup} (-5% to account for variation of the estimate) are excluded.

It should be noted, that the basis of our approach – the list of polymorphisms – is generated using VarDict [21] (for details on variant calling see Additional file 1, section 1.3.2).

Merging

Raw CNV calls that are reported as being overlapping or located in close vicinity are likely corresponding to one event. Thus, merging of the raw calls is performed. The merging scheme is visualized in Figure 3.

Two CNV calls are merged if the same variant is reported (deletion or duplication), CF is similar ($\lesssim$3 standard deviations – sd) and the calls are overlapping. If two CNV calls are not overlapping, but no significant 'no CNV'-region is located in between and the regions are close (< 20 *Mbp*) they are likewise merged.

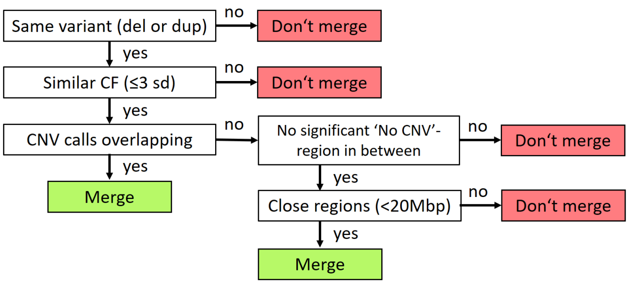

Figure 3. Decision tree for the merging process. Two CNV calls are merged according to the displayed merging scheme. Defining close regions as being separated by < 20 *Mbp* allows for merging of two regions separated by a centromer, which is $\leq$ 7.4 *Mbp*, and for detecting monosomy or trisomy of the smallest chromosomes by only two significant regions.

Note that for estimating the CFs of the merged regions, all significant SNPs are re-evaluated. This can, in some rare cases, lead to a merged region with an overall estimated CF below the actual detection thresholds. However, these regions always contain at least two raw CNV calls with CFs above the detection thresholds.

Filtration (opt.)

Optionally, the merged results can be filtered on the basis of the CNV call quality. Dependent on the analyzed data, it can be useful to consider the merged calls directly. However, we recommend filtration of low quality calls.

Comparison to common approaches

Over the last years, several review articles have been published, considering tools available for CNV calling in NGS data [9, 22, 23]. To evaluate performance of our novel CNV calling algorithm, we compare it to five common CNV calling tools for WES data: ExomeCNV (ExomeCNV, RRID:SCR_010815) [11], VarScan2 (VARSCAN, RRID:SCR_006849) [10], ExomeDepth (ExomeDepth, RRID:SCR_002663) [13], Control-FREEC (Control-FREEC, RRID:SCR_010822) [14] and CNV-seq (CNV-seq, RRID:SCR_013357) [15].

ExomeCNV uses read depth and B-allele frequencies (BAF) from matched WES data to detect deletions, duplications and LOH. It is frequently used for benchmarking [22]. We analyze the CNV calls reported in <Sample>.cnv.txt. The copy number reported in column *copy.number* is evaluated.

VarScan2 analyzes normalized read depth in matched WES samples to detect deletions and duplications. For every region, *num.mark* and *seg.mean* are reported. We exclude all variants with *num.mark* < 10. If *seg.mean* $\geq$ 0.25, the variant is considered a duplication. If *seg.mean* $\leq$ −0.25, the variant is considered a deletion. All variants with −0.25 < *seg.mean* < 0.25 are discarded.

Control-FREEC analyzes copy number- and BAF profiles. Matched control sample are evaluated to distinguish germline variants from somatic ones. Information on subclonal gains and losses is reported and additionally evaluated if biological truth contains information on clonal composition of the samples. In addition to the standard Control-FREEC pipeline, we applied the additional script "assess_significance.R" [24]. CNV calls with a reported p value > 0.05 are excluded. We consider both *WilcoxonRankSumPvalue* (WR) and *KolmogorovSmirnovPvalue* (KS). The copynumber, considering deletions, duplications and LOH, reported in column *copy number* is evaluated.

ExomeDepth applies a beta-binomial model to a set of exons. Normally, the tool requires multiple samples as input. The

idea is that each exome is automatically compared to the exome featuring best correlation. However, as for all samples in our data sets matched controls are available, we assume that the matching control is always the best exome to be used for comparison. The copy number, considering deletions and duplications, reported in column *type* is evaluated.

Additionally, we consider CNV-seq. The tool has not been specifically designed for WES data. However, the general approach is similar to our novel approach CopyDetective: a sliding window is evaluated. The window size is defined by data quality, i.e. in this case coverage. Copy number ratios as well as confidence values are determined. However, different from CopyDetective, CNV calling with CNV-seq is solely based on coverage and not on BAFs. To process the raw output, we exclude all calls with missing values in columns *log2* and/or *cnv.size*. Regions belonging to the same CNV (identifier in column *cnv*) are merged. All merged calls with *cnv.p.value* > 0.05 are excluded. The remaining calls are categorized as deletions if *cnv.log2* < −0.25 and as duplications if *cnv.log2* > 0.25. All the other calls are categorized as LOH.

Details on the precise commands for executing CNV calling with the common approaches are provided in Additional file 1, section 1.4. It should be noted that we tried to apply several additional tools on our data, e.g. THetA2 (THetA, RRID:SCR_001860) [25] or iCNV [26]. Information on all tools we tested, and the reasons why they were excluded from further consideration can be found in Additional file 1, section 1.5.

Results

We apply CopyDetective (simulation approach) on four sets of real data. Performance is compared to five established tools for CNV calling in WES data: ExomeCNV, VarScan2, ExomeDepth, Control-FREEC (WR and KS) and CNV-seq. Two samples from data set 2 (BL_03: P3 and R3) were excluded from analysis. Detailed analyses have shown that almost all validated CNVs appear to have been already present in the control sample – either being contamination or germline calls (for details see Additional file 1, section 2.1, Figure S5). The results for the remaining 98 samples are summed up in Table 2. It should be noted that LOH was excluded from data set 4 as we do not have any information on the frequency of affected cells for these calls. All called CNVs of the type 'LOH' were removed from the output of ExomeCNV, ControlFREEC and CNV-seq. CNV calls reported by CopyDetective that were overlapping regions of validated LOH were equally removed. An analysis of data set 4 including LOH can be found in Additional file 1, section 2.2.

A CNV call is considered true positive if it shows any overlap with at least one validated CNV. If a CNV call is overlapping a true variant, but features the "wrong" CNV type (e.g. a deletion is called, while the true CNV is a duplication), is is reported in brackets, as true positive call with false type. We evaluate sensitivity (sens), the positive predictive value (PPV) and the F1 score only considering the true positive calls with correct CNV type (for details on how these statistics are calculated see Additional file 1, section 1.6). For every variant calling tool the number of detectable CNVs is defined. For VarScan and ExomeDepth the number of detectable CNVs is decreased as these two tools are not able to detect LOH. For CopyDetective the number of detectable CNVs is decreased based on every samples' individual detection thresholds for CNV length and CF (see Additional file 1, section 2.3, Tables S6-S9 for precise detection thresholds). Exemplary variant calling results for one sample are visualized in Additional file 1, section 2.4, Figure S7. A detailed overview of all missed and detected CNVs for each tool is provided in Additional file 1, section 2.4, Tables S10-S13. Detailed variant calling results for CopyDetective are provided in Additional file 2. Results for CopyDetective using the exact approach can be found in Additional file 1, section 2.5, Figure S8 and Table S14.

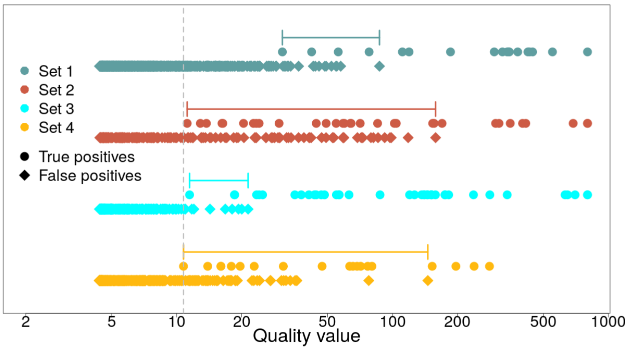

Figure 4. Quality values for true positive and false positive CNV calls reported by CopyDetective (evaluating the true CNV call with the highest quality value in case more than one CNV call overlaps a validated CNV). Ranges indicate the possible values of the quality threshold for each data set. Dashed gray line indicates the default filtration threshold of 10.76, excluding no true positive calls.

It can be observed that a majority of common variant calling tools is characterized by low PPV. For ExomeCNV, Control-FREEC (both configurations, WR and KS) and CNV-seq PPV ranges between <0.01 and 0.09 for all data sets. Just in case of VarScan and ExomeDepth – both tools not being able to detect LOH – higher PPVs can partly be observed. However, performance is highly data dependent (set 1: $PPV_{VarScan2} = 0.01$, $PPV_{ExomeDepth} = 0.02$; set 4: $PPV_{VarScan2} = 0.36$, $PPV_{ExomeDepth} = 0.71$). Considering our novel approach CopyDetective without filtration (config 'raw'), PPV ranges between 0.04 and 0.26. Over all data sets, performance is comparable to the best common approach ExomeDepth (PPV 0.11 vs 0.12). If we apply filtration with our default threshold, values between 0.12 and 0.86 can be observed (over all data sets: 0.33).

Regarding sensitivity, huge differences between all approaches can be observed. While ControlFREEC and CNV-seq are characterized by low sensitivity (maximum: 0.45), much higher values can be observed in case of ExomeCNV (up to 0.89; on average 0.61). However, due to low PPV, the overall performance considering the F1 score is, over all data sets, relatively poor. Similar to PPV, ExomeDepth features highly data dependent performance with respect to sensitivity (ranging between 0.27 and 0.68). In contrast to this, CopyDetective is characterized by stable sensitivity. For both the raw and the filtered results, sensitivity ranges between 0.92 and 1.00. On average, *sens* = 0.96, which slightly exceeds our user-defined sensitivity of 0.95 when determining the detection thresholds.

Table 2 shows that the performance of CopyDetective is, with respect to PPV, data dependent – also including the influence of filtration. Additional file 1, Figure S9 (section 2.6) shows the relation between sensitivity and PPV in the context of an increasing quality threshold. A different development can be observed for the different data sets. Optimization of the F1 score would result in different optimal thresholds for each set (1: 56.05; 2: 16.22; 3: 18.52; 4: 36.36). Thus, the optimal quality threshold over all data sets is difficult to define. However, differences between the data sets are less prominent, when considering the true calls' quality values (see Figure 4).

For all data sets it can be observed that true CNV calls are characterized by higher quality values compared to false positives. Combining all data sets, no true positive call with a quality value < 10.76 can be observed (see Additional file 1, section 2.6, Table S15). Three out of 4 data sets share a most similar threshold (2: 11.19; 3: 11.48; 4: 10.76). Therefore, we decided to select 10.76 as our default threshold for quality filtration ap-

Table 2. Performance of CopyDetective (raw, i.e. without optional final filtration, and filter, i.e. with default filtration threshold of 10.76) in comparison to five established approaches: ExomeCNV, VarScan2, ExomeDepth, Control-FREEC (WR and KS) and CNV-seq. True positive (TP) calls (in brackets: reporting the number of additional true positive calls if CNV type is not evaluated), false positive (FP) calls, found, missed and detectable CNVs, sensitivity (sens; just evaluating true positive calls with correct CNV type), positive predictive value (PPV; just evaluating true positive calls with correct CNV type) and the F1 score.

Tool	Config		Data set	TP calls (+ false type)	FP calls	CNVs found	CNVs missed	CNVs detectable	Sens	PPV	F1
ExomeCNV			1	1378 (+2204)	215865	49	6	55	0.89	0.01	0.01
			2	280 (+508)	13213	32	15	47	0.68	0.02	0.04
			3	1017 (+1904)	66686	40	8	48	0.83	0.02	0.03
			4	94 (+1)	2064	24	64	88	0.27	0.04	0.08
VarScan2			1	119 (+126)	11736	27	22	49	0.55	0.01	0.02
			2	106 (+185)	2758	26	11	37	0.70	0.04	0.07
			3	65 (+16)	374	21	14	35	0.60	0.15	0.24
			4	30 (+0)	54	23	65	88	0.26	0.36	0.30
ExomeDepth			1	163 (+50)	8074	13	36	49	0.27	0.02	0.04
			2	275 (+162)	2042	25	12	37	0.68	0.12	0.20
			3	175 (+33)	2047	20	15	35	0.57	0.08	0.14
			4	909 (+0)	375	32	56	88	0.36	0.71	0.48
ControlFREEC	WR		1	7 (+6)	1568	5	50	55	0.09	<0.01	0.01
			2	6 (+3)	278	3	44	47	0.06	0.02	0.03
			3	7 (+9)	654	6	42	48	0.13	0.01	0.02
			4	5 (+2)	231	5	83	88	0.06	0.02	0.03
ControlFREEC	KS		1	24 (+38)	7261	12	43	55	0.22	<0.01	0.01
			2	16 (+11)	1124	10	38	48	0.21	0.01	0.03
			3	16 (+11)	1124	10	38	48	0.21	0.01	0.03
			4	32 (+4)	224	17	71	88	0.19	0.09	0.13
CNV-seq			1	25690 (+27757)	1723974	21	34	55	0.38	0.01	0.03
			2	3016 (+1885)	94461	21	26	47	0.45	0.03	0.06
			3	6628 (+4518)	316311	19	29	48	0.40	0.02	0.04
			4	786 (+1125)	28863	15	73	88	0.17	0.03	0.05
CopyDetective	raw		1	33 (+22)	729	18	1	19	0.95	0.04	0.08
			2	63* (+43)	176	34	3	37	0.92	0.26	0.41
			3	67 (+21)	212	40	1	41	0.98	0.24	0.39
			4	23 (+23)	399	19	0	19	1.00	0.05	0.10
CopyDetective	filter	10.76	1	25 (+15)	180	18	1	19	0.95	0.12	0.22
		10.76	2	50 (+31)	63	34	3	37	0.92	0.44	0.60
		10.76	3	60 (+18)	10	40	1	41	0.98	0.86	0.90
		10.76	4	22 (+16)	63	19	0	19	1.00	0.26	0.41

*Sixty-four detected CNVs are overlapping true CNVs. However, as one called CNV is clearly shorter than the validated one and characterized by a remarkably low quality value, we assume that this overlap is just occurring at coincidence. Therefore, it is counted as ‘missed’.

plied in step 4 of our algorithm.

Discussion and Conclusion

CopyDetective is a novel tool for calling somatic CNVs in matched WES data. It has been developed for, but is not limited to the analysis of cancer samples. Different from any other approach, CopyDetective performs initial quality analysis of every sample to estimate the individual detection thresholds, covering the minimum CNV length and the minimum cell fraction. These detection thresholds allow subsequent CNV calling with user-defined sensitivity (default: 0.95).

Considering performance of our new approach, we observe high sensitivity regarding high- as well as low-coverage data. Over all data sets, CopyDetective outperforms all the other tools we considered, even without optional filtration of low-quality calls. Application of the quality filter results in further improvement of performance, especially with respect to PPV. Data indicates that a threshold of 10.76 can be used safely to exclude false positive calls. Detailed additional analyses show that the coordinates of the CNVs, determined by CopyDetective, match the coordinates based on validation experiments (see Additional file 1, section 2.7, Figures S10-S11 and Additional file 2). Furthermore, CFs estimated by CopyDetective match the cell fractions determined by other methods (like fluorescent *in situ* hybridization; see Additional file 1, section 2.8, Figure S12 and Additional file 2). However, it should be noted that the assumed true coordinates and cell fractions may differ from the actual true values. A precise determination of a CNV's coordinates is usually not possible, but can just be estimated. Furthermore, cell fractions that are based on clonal evolution analysis (sets 1 and 2) may be biased by clustering. A CNV may be present in more or less cells compared to the other mutations in its cluster.

Yet, the fact that CopyDetective is able to estimate CFs is an important characteristic, especially with respect to clonal evolution. While allele frequencies of pathogenic mutations can easily be analyzed to determine subclonal composition of a tumor, this should also be done when analyzing CNVs. However, most tools do just report a copy number variant and its CNV value, but not the fraction of cells affected by the mutation. To

our knowledge, only two additional tools are able to estimate tumor purity in NGS data: CNAnorm [27] and THetA2 [25] (in addition, there are ASCAT [28] and ABSOLUTE [29], however, these tools were designed for SNP array data). While superior performance of THetA2 has been reported by Oesper et al., the tool failed on our data (see Additional file 1, section 1.5).

It may seem astonishing that CNVs reported by CopyDetective match the validated CNVs, spread all over the genome, with respect to coordinates and CFs so well while just analyzing WES data. However, the main advantage of our approach lies in the analysis of a sliding window and the subsequent merging of windows located in close vicinity. This approach allows us to explore between 99.5 and 99.8% of the human genome (see Additional file 1, section 2.3.1, Figure S6).

However, CopyDetective certainly has some limitations. We need a specific scenario – matching control samples – to evaluate changes in VAF for every polymorphism. Performance of CopyDetective is dependent on the accuracy of polymorphism calling in the control sample. However, analyses of robustness have shown that CopyDetective is especially tolerant towards false negative polymorphism calls (see Additional File 1, section 2.9.1, Figures S13-S14 and Tables S16-S17). Our approach is currently just able to call simple deletions or duplications. Dependent on the cells affected by a CNV, ambiguous results are possible as e.g. a deletion present in 50% of the cells can also be explained by a duplication present in 100% of the cells. LOH is always reported as a deletion by CopyDetective. However, a coverage indicator is reported. Analyses show that a true deletion is characterized by a negative coverage indicator (sens=0.88), while LOH is characterized by a coverage indicator overlapping zero (sens=0.87) (see Additional file 1, section 2.10, Figure S15).

Currently, gonosomes are not evaluated by CopyDetective. CNVs on the Y chromosome cannot be detected, as all polymorphisms are hemizygous (same is true for small CNVs just covering homozygous polymorphisms). However, our approach is expected to work for the X chromosomes in case of women.

Dependent on the quality of the provided input data, CopyDetective may not detect and report any small CNVs like focal CNVs, which are known to play an important role in cancer [30]. However, CopyDetective's detection thresholds serve to ensure sufficient sensitivity of the CNV calling results. By reporting the minimum CNV length and the minimum cell fraction, it can easily be decided on whether the analyzed WES data is sufficient to detect the CNVs of interest or if additional experiments have to be performed. When manually changing CopyDetective's automatically determined detection thresholds to higher or lower values, we observe a decline in performance (see Additional file 1, section 2.9.2, Table S18). Higher, stricter thresholds decrease the number of detectable CNVs, no longer tapping the full potential of the data. On the contrary, lower thresholds lead to a major increase in false positive calls.

Concluding, CopyDetective unites an established idea – evaluating the change in VAF of polymorphisms to detect CNVs – with a completely new aspect – determining individual detection thresholds for every sample. Thereby, CopyDetective shines a new light on CNV calling in WES data: Individual detection thresholds reveal that not every data set is equally apt for CNV calling. The general idea of our algorithm – applying a 2-step procedure – is combinable with any other CNV-calling approach. Initial quality analyses, determining individual detection thresholds, can and should be performed prior to actual variant calling.

Availability of source code and requirements

Project name: CopyDetective [12]
Project home page: https://github.com/sandmanns/CopyDetective
Operating system: Platform independent
Programming language: R
Other requirements: none
License: AGPL-3.0
bio.tools ID: biotools:copydetective
SciCrunch ID: RRID:SCR_018909

Availability of supporting data and materials

Data and materials supporting the results of this article are available in Additional file 1 and 2. Sequencing data are available at the NCBI Sequence Read Archive (PRJNA355124, PRJNA561490 and PRJNA285732), the EMBL-EBI European Nucleotide Archive (PRJEB36436), Array Express (E-MTAB-8763) and the Gene Expression Omnibus (GSE68078). All supporting data and materials are available in the *GigaScience* GigaDB database [31].

Additional files

Additional file 1

SupplementaryInformation.pdf: information on supplementary methods and results.

Additional file 2

SupplementaryData1.xlsx: CNV calling output from CopyDetective, including raw and filtered calls for data sets 1 to 4.

Declarations

List of abbreviations

BAF: B-allele frequencies; BL: Burkitt lymphoma; CCF: cancer cell fraction; CF: cell fraction; CNV: copy number variant; FP: false positive; indel: insertion and deletion; KS: KolmogorovSmirnovPvalue; LOH: loss of heterozygosity; MDS: myelodysplastic syndromes; NGS: next-generation sequencing; NMZL: nodal marginal zone lymphoma; PPV: positive predictive value; sd: standard deviation; sens: sensitivity; SNV: single-nucleotide variant; SNP: single-nucleotide polymorphism; SV: structural variant; T-LBL: T-lymphoblastic lymphoma; TP: true positive; VAF: variant allele frequency; WES: whole-exome sequencing; WGS: whole-genome sequencing; WR: WilcoxonRankSumPvalue

Ethical Approval

All patient material was collected and analyzed in accordance with the relevant ethical guidelines and regulations. Informed consent was obtained from all subjects.

Consent for publication

Not applicable

Competing Interests

The authors declare that they have no competing interests.

Funding

This work has been supported by the EU grant Horizon2020 MDS-RIGHT (grant no. 634789), the DFG grant TU 298/5-1 (DFG Clinical Research Unit 326 Male Germ Cells: from Genes to Function), a grant from Deutsche Krebshilfe DKH (grant no. 111347), by Löwenkinder – Verein zur Unterstützung krebskranker Kinder e.V., and by Deutsche Kinderkrebsstiftung (support of the NHL-BFM Registry 2012; DKS349 2014.11 A/B).

Author's Contributions

S.S. developed the algorithm, performed data analyses and wrote the manuscript. S.S. and M.W. performed analysis of validation data. A.O.d.G and J.H.J. collected patient samples and coordinated targeted mutational and whole exome sequencing on the MDS cases. B.B. collected patient samples and coordinated whole exome sequencing on the T-LBL cases. M.D. reviewed development of the algorithm and reviewed the manuscript. All authors read, revised and approved the final version of the manuscript.

Acknowledgements

Not applicable

References

1. Park JY, Kricka LJ, Fortina P. Next-generation sequencing in the clinic. Nat Biotechnol 2013;31:990–992.
2. Release IP. Illumina Introduces the NovaSeq Series – a New Architecture Designed to Usher in the $100 Genome. San Diego (Business Wire) 2017;p. https://www.illumina.com/company/news-center/press-releases/press-release-details.html?newsid=2236383.
3. Ashley EA. Towards precision medicine. Nat Rev Genet 2016;17:507–522.
4. Dey N, Williams C, Leyland-Jones B, De P. Mutation matters in precision medicine: a future to believe in. Cancer Treat Rev 2017;55:136–149.
5. Luthra R, Chen H, Roy-Chowdhuri S, Singh RR. Next-Generation Sequencing in Clinical Molecular Diagnostics of Cancer: Advantages and Challenges. Cancers (Basel) 2015;7:2023–2036.
6. Cheng ML, Solit DB. Opportunities and Challenges in Genomic Sequencing for Precision Cancer Care. Ann Intern Med 2018;168:221–222.
7. Bao R, Huang L, Andrade J, Tan W, Kibbe WA, Jiang H, et al. Review of current methods, applications, and data management for the bioinformatics analysis of whole exome sequencing. Cancer Inform 2014;13:67–82.
8. Sandmann S, de Graaf AO, Karimi M, van der Reijden BA, Hellström-Lindberg E, Jansen JH, et al. Evaluating variant calling tools for non-matched next-generation sequencing data. Sci Rep 2017;7:43169.
9. Zare F, Dow M, Monteleone N, Hosny A, Nabavi S. An evaluation of copy number variation detection tools for cancer using whole exome sequencing data. BMC Bioinformatics 2017;18:286.
10. Koboldt DC, Zhang Q, Larson DE, Shen D, McLellan MD, Lin L, et al. VarScan 2: Somatic mutation and copy number alteration discovery in cancer by exome sequencing. Gen Res 2012;22:568–576.
11. Sathirapongsasuti JF, Lee H, Horst BA, Brunner G, Cochran AJ, Binder S, et al. Exome sequencing-based copy-number variation and loss of heterozygosity detection: ExomeCNV. Bioinformatics 2011;27:2648–2654.
12. Sandmann S. CopyDetective-v1.1.0 2020;DOI: 10.5281/zenodo.3972092.
13. Plagnol V, Curtis J, Epstein M, Mok KY, Stebbings E, Grigoriadou S, et al. A robust model for read count data in exome sequencing experiments and implications for copy number variant calling. Bioinformatics 2012;28:2747–2754.
14. Boeva V, Popova T, Bleakley K, Chiche P, Cappo J, Schleiermacher G, et al. Control-FREEC: a tool for assessing copy number and allelic content using next-generation sequencing data. Bioinformatics 2012;28:423–425.
15. Xie C, Tammi MT. CNV-seq, a new method to detect copy number variation using high-throughput sequencing. BMC Bioinformatics 2009;10:80.
16. da Silva-Coelho P, Kroeze LI, Yoshida K, Koorenhof-Scheele TN, Knops R, van de Locht LT, et al. Clonal evolution in myelodysplastic syndromes. Nat Commun 2017;8:15099.
17. Reutter K, Sandmann S, Rohde J, Mueller S, Wöste M, Khanam T, et al. Reconstructing Clonal Evolution in Relapsed and Non-Relapsed Burkitt Lymphoma. Leukemia 2020;.
18. DNA Copy Number and Loss of Heterozygosity Analysis Algorithms; https://www.illumina.com/documents/products/technotes/technote_cnv_algorithms.pdf.
19. Spina V, Khiabanian H, Messina M, Monti S, Cascione L, Bruscaggin A, et al. The genetics of nodal marginal zone lymphoma. Blood 2016;128:1362–1373.
20. Mayrhofer M, Viklund B, Isaksson A. Rawcopy: Improved copy number analysis with Affymetrix arrays. Sci Rep 2016;6:36158.
21. Lai Z, Markovets A, Ahdesmaki M, Chapman B, Hofmann O, McEwen R, et al. VarDict: A novel and versatile variant caller for next-generation sequencing in cancer research. Nucleic Acids Res 2016;1:e108.
22. Kadalayil L, Rafiq S, Rose-Zerilli MJ, Pengelly RJ, Parker H, Oscier D, et al. Exome sequence read depth methods for identifying copy number changes. Brief Bioinform 2015;16:380–92.
23. Zhao M, Wang Q, Wang Q, Jia P, Zhao Z. Computational tools for copy number variation (CNV) detection using next-generation sequencing data: features and perspective. BMC Bioinformatics 2013;14:S1.
24. Control-FREEC; https://github.com/BoevaLab/FREEC/blob/master/scripts/assess_significance.R.
25. Oesper L, Mahmoody A, Raphael BJ. Quantifying Tumor Heterogeneity in Whole-Genome and Whole-Exome Sequencing Data. Bioinformatics 2014;30:3532–3540.
26. Zhou Z, Wang W, Wang LS, Zhang NR. Integrative DNA copy number detection and genotyping from sequencing and array-based platforms. Bioinformatics 2018;34:2349–2355.
27. Gusnanto A, Wood HM, Pawitan Y, Rabbitts P, Berri S. Correcting for cancer genome size and tumour cell content enables better estimation of copy number alterations from next-generation sequence data. Bioinformatics 2012;28:40–47.
28. Van Loo P, Nordgard SH, Lingjorde OC, Russnes HG, Rye IH, Sun W, et al. Allele-specific copy number analysis of tumors. Proc Natl Acad Sci USA 2010;107:16910–16915.
29. Carter SL, Cibulskis K, Helman E, McKenna A, Shen H, Zack T, et al. Absolute quantification of somatic DNA alterations in human cancer. Nat Biotechnol 2012;30:413–421.
30. Krijgsman O, Carvalho B, Meijer GA, Steenbergen RDM, Ylstra B. Focal chromosomal copy number aberrations in cancer—Needles in a genome haystack. Biochim Biophys Acta

2014;1843:2698–2704.
31. Sandmann S, Wöste M, de Graaf AO, Burkhardt B, Jansen JH, Dugas M. Supporting data for "CopyDetective: Detection Threshold Aware CNV Calling in WES Data. GigaScience Database 2020; http://doi.org/10.5524/100800.

Figure for Latex file Supplement.tex
Click here to access/download;LaTeX - Other (clo, cls, sty etc.);FigS1.png

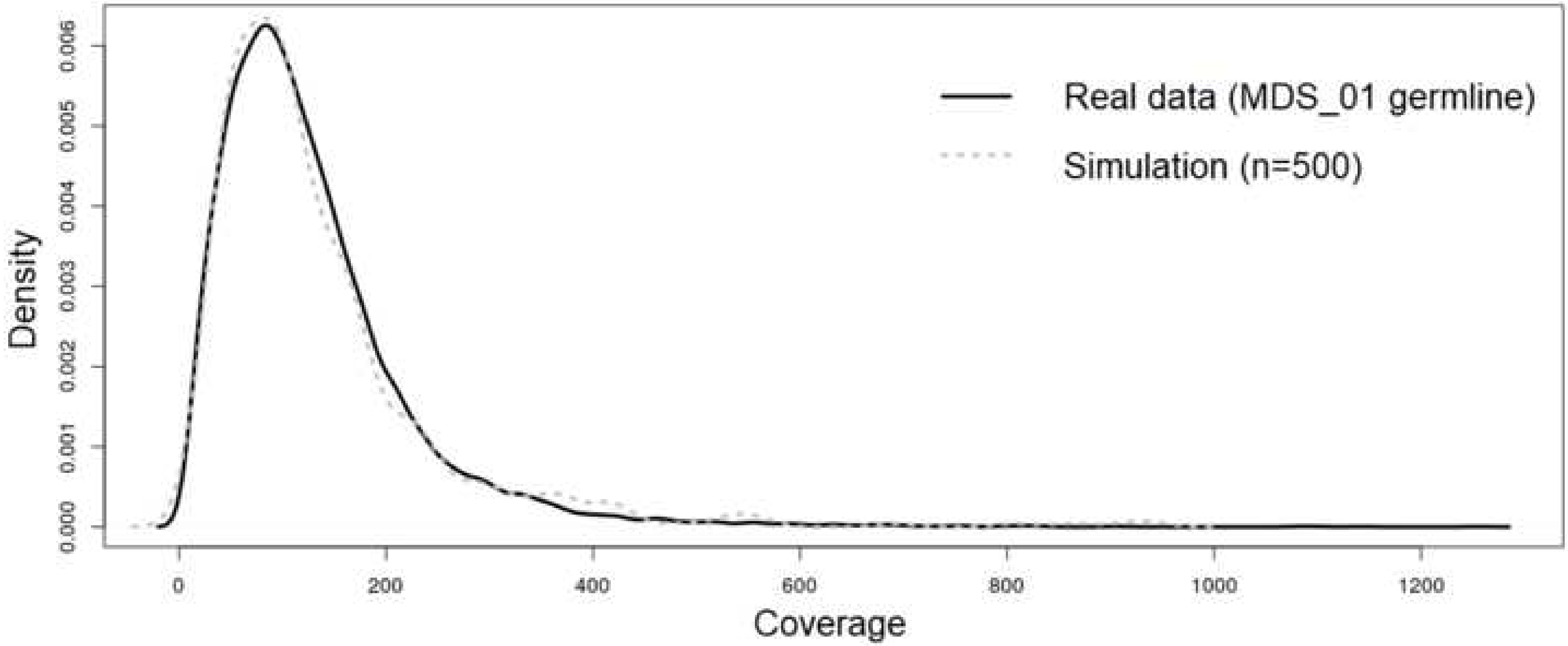

Figure for Latex file Supplement.tex
Click here to access/download;LaTeX - Other (clo, cls, sty etc.);FigS10A.png

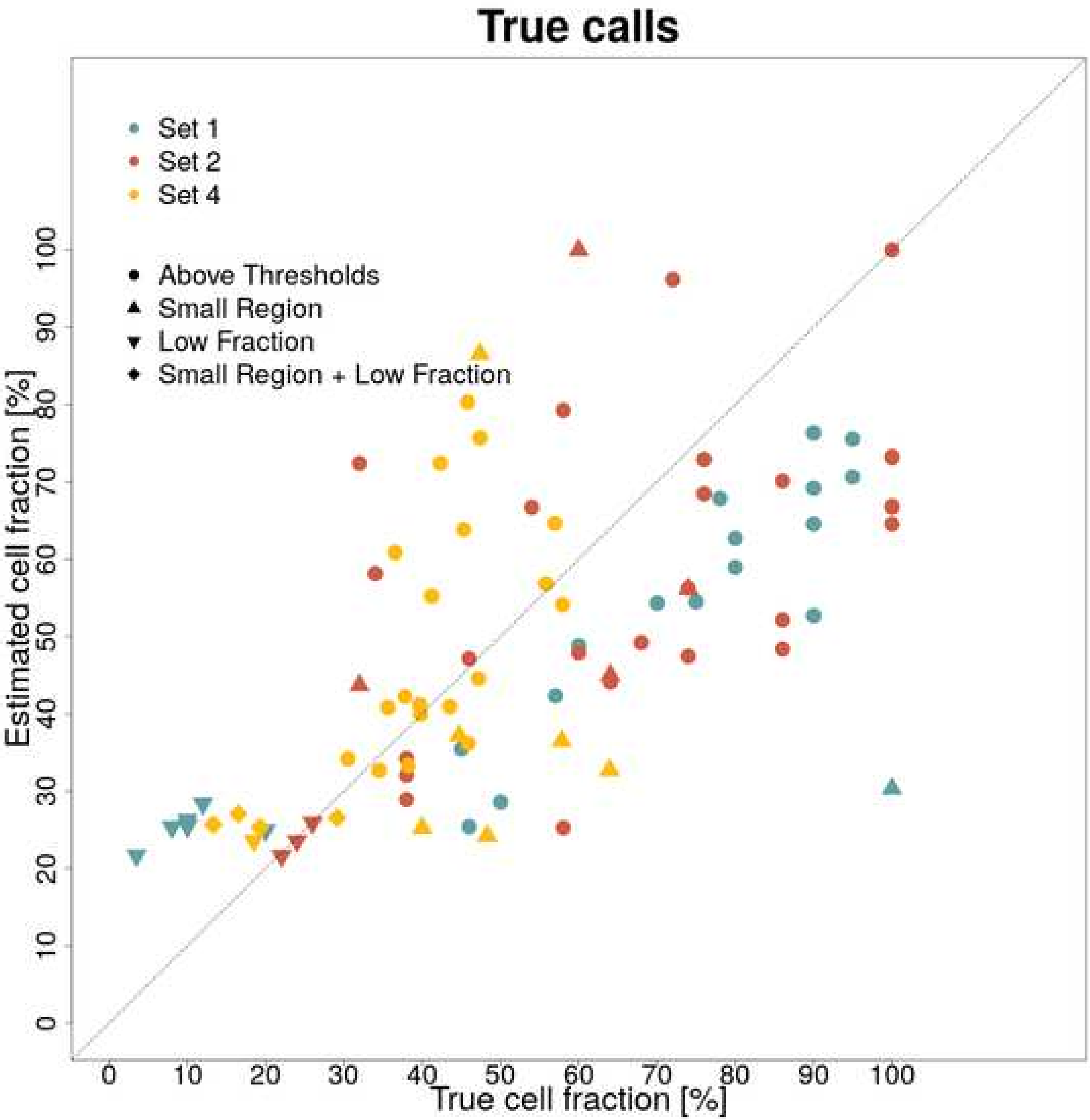

Figure for Latex file Supplement.tex
Click here to access/download;LaTeX - Other (clo, cls, sty etc.);FigS10B.png

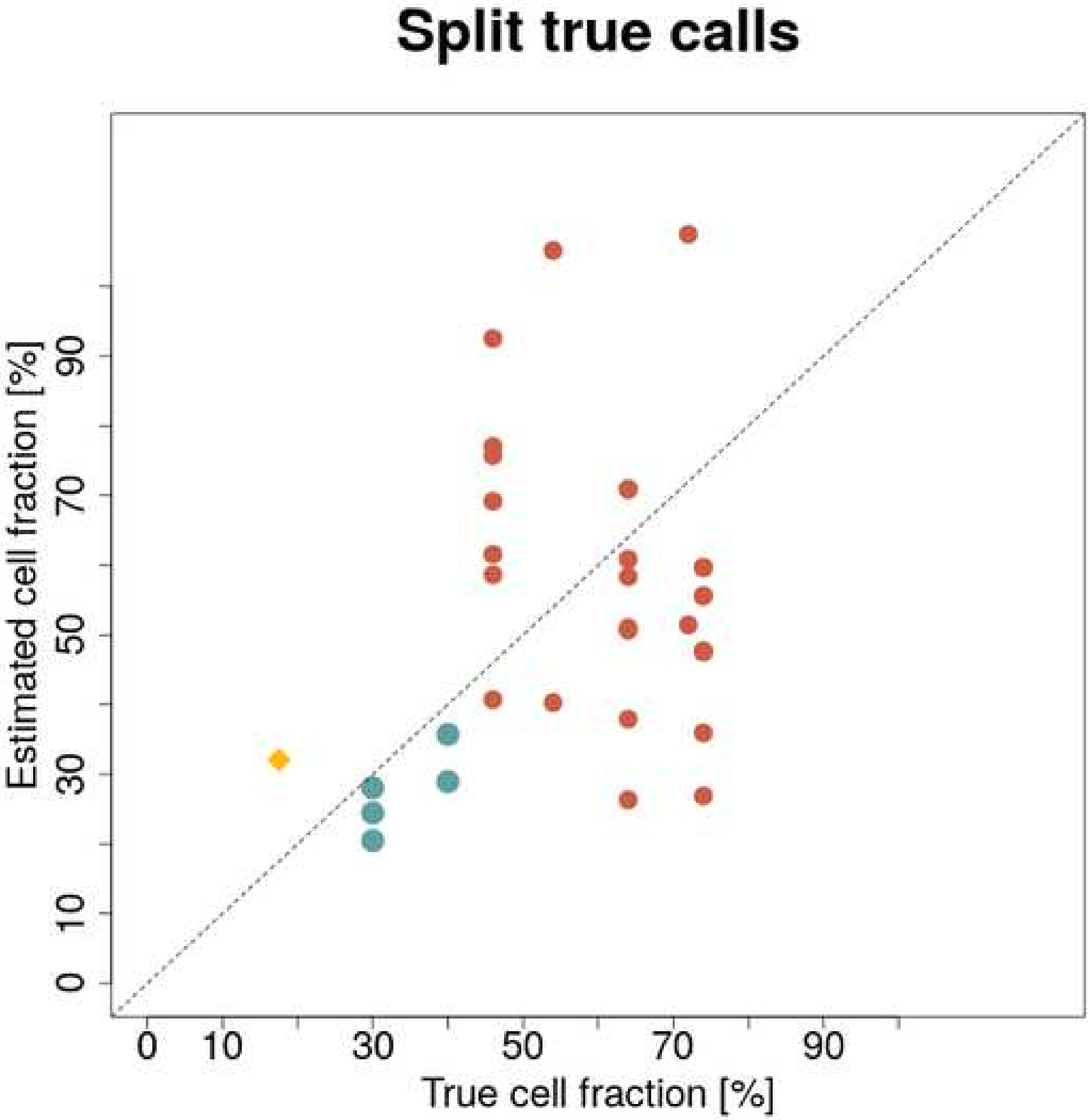

Figure for Latex file Supplement.tex
Click here to access/download;LaTeX - Other (clo, cls, sty etc.);FigS10C.png

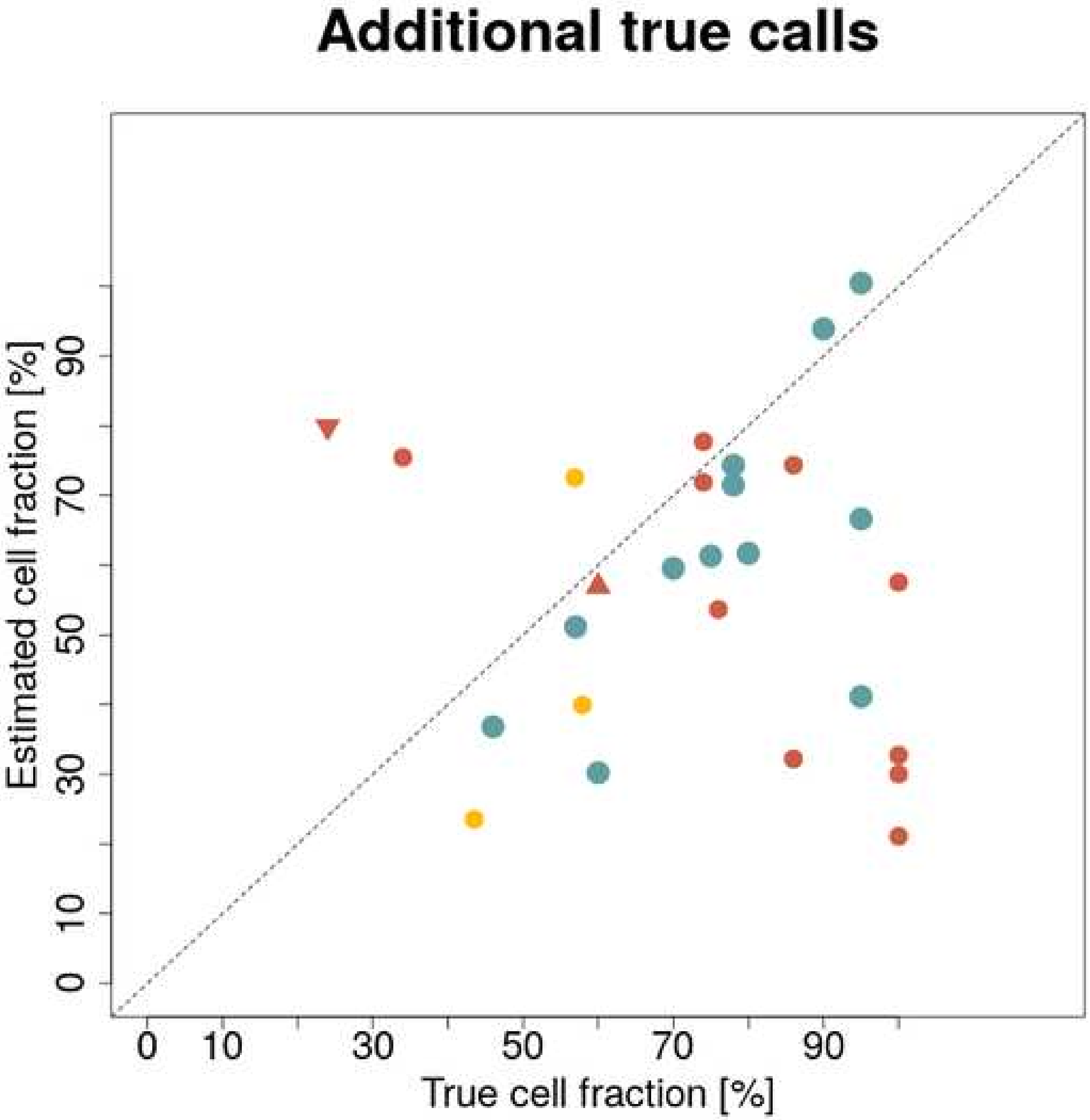

Click here to access/download;LaTeX - Other (clo, cls, sty etc.);FigS11A.png

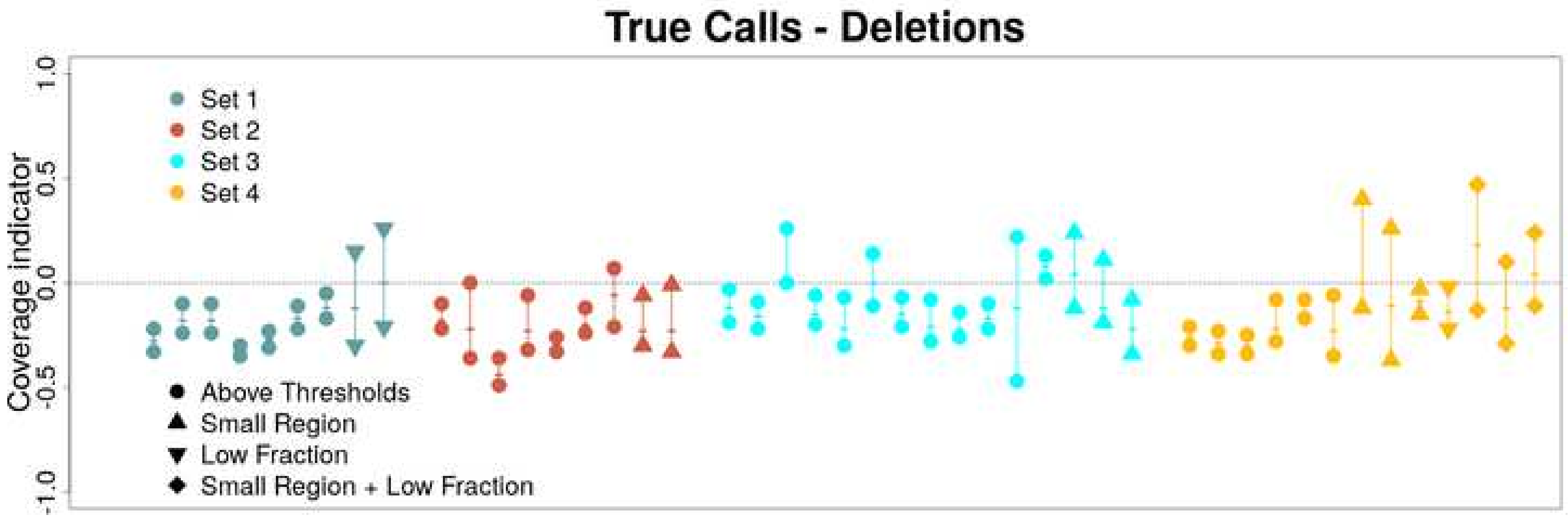

Figure for Latex file Supplement.tex
Click here to access/download;LaTeX - Other (clo, cls, sty etc.);FigS11B.png

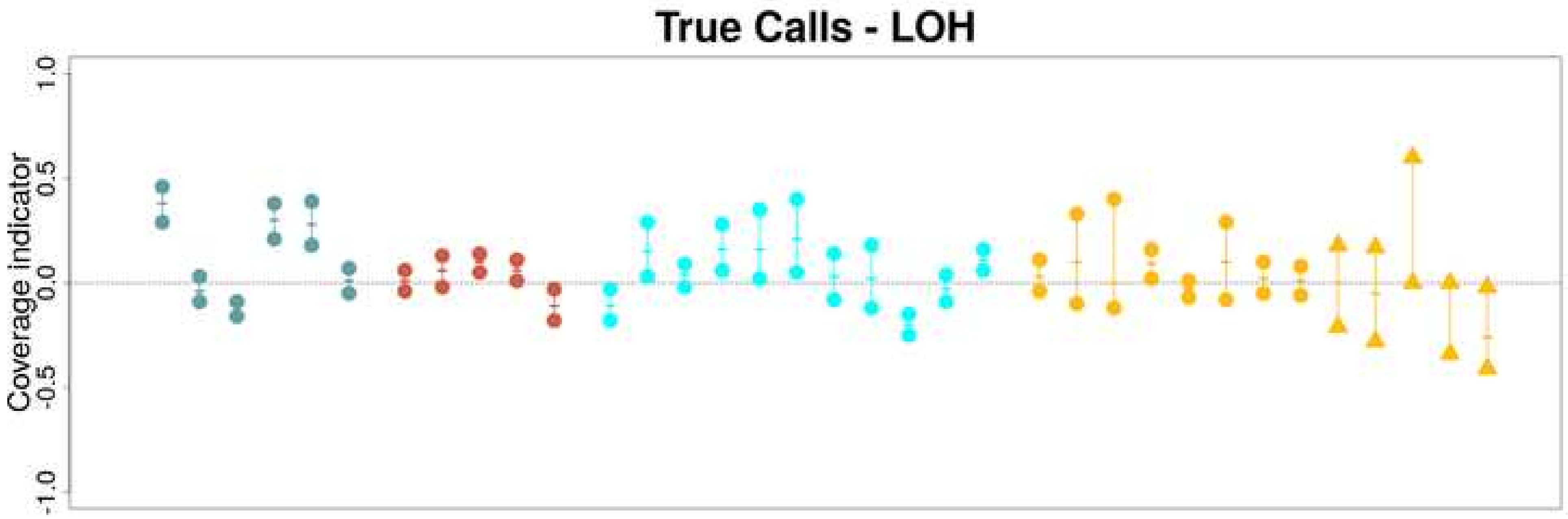

Figure for Latex file Supplement.tex

Click here to access/download;LaTeX - Other (clo, cls, sty etc.);FigS12.png

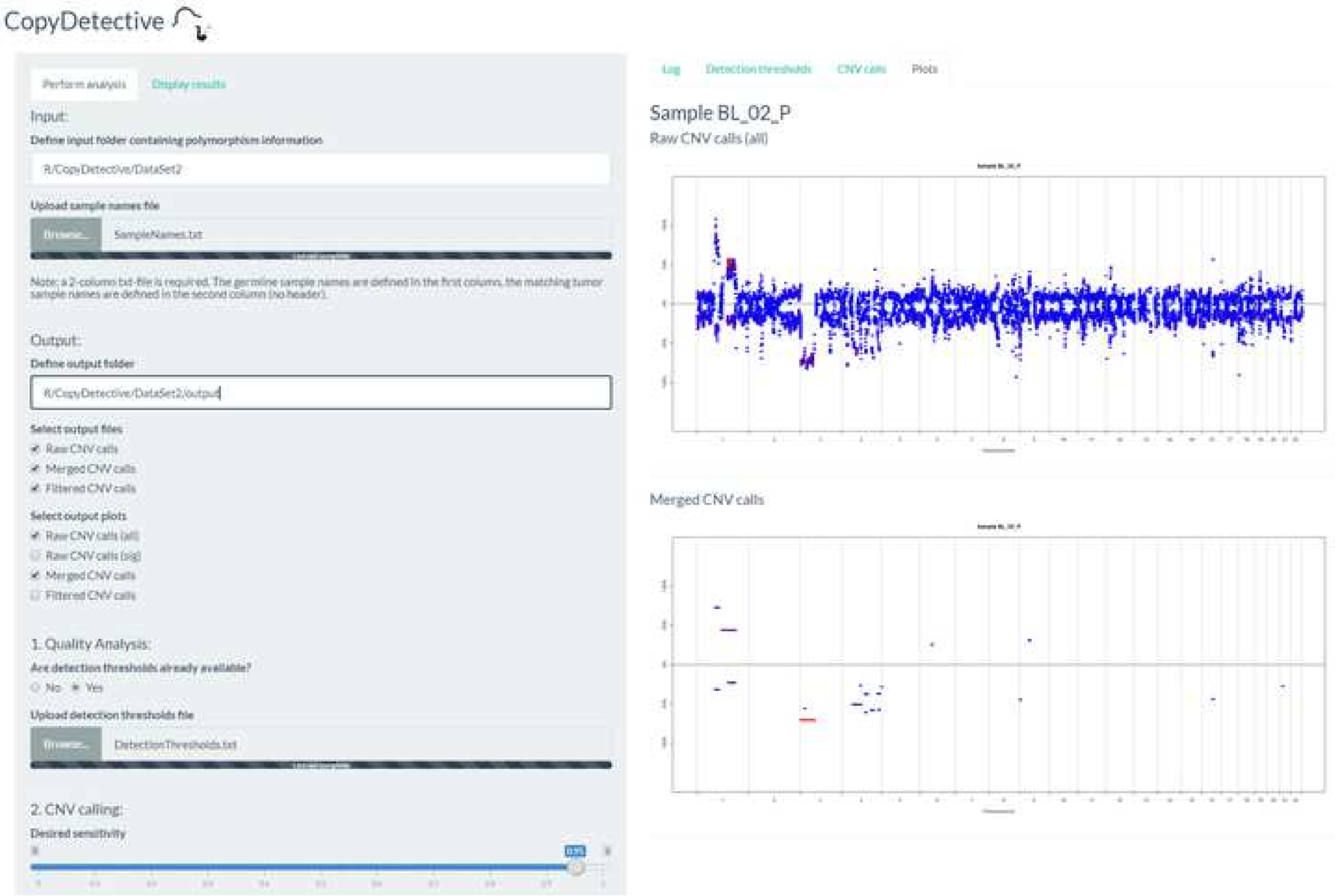

Figure for Latex file Supplement.tex
Click here to access/download;LaTeX - Other (clo, cls, sty etc.);FigS12a.png

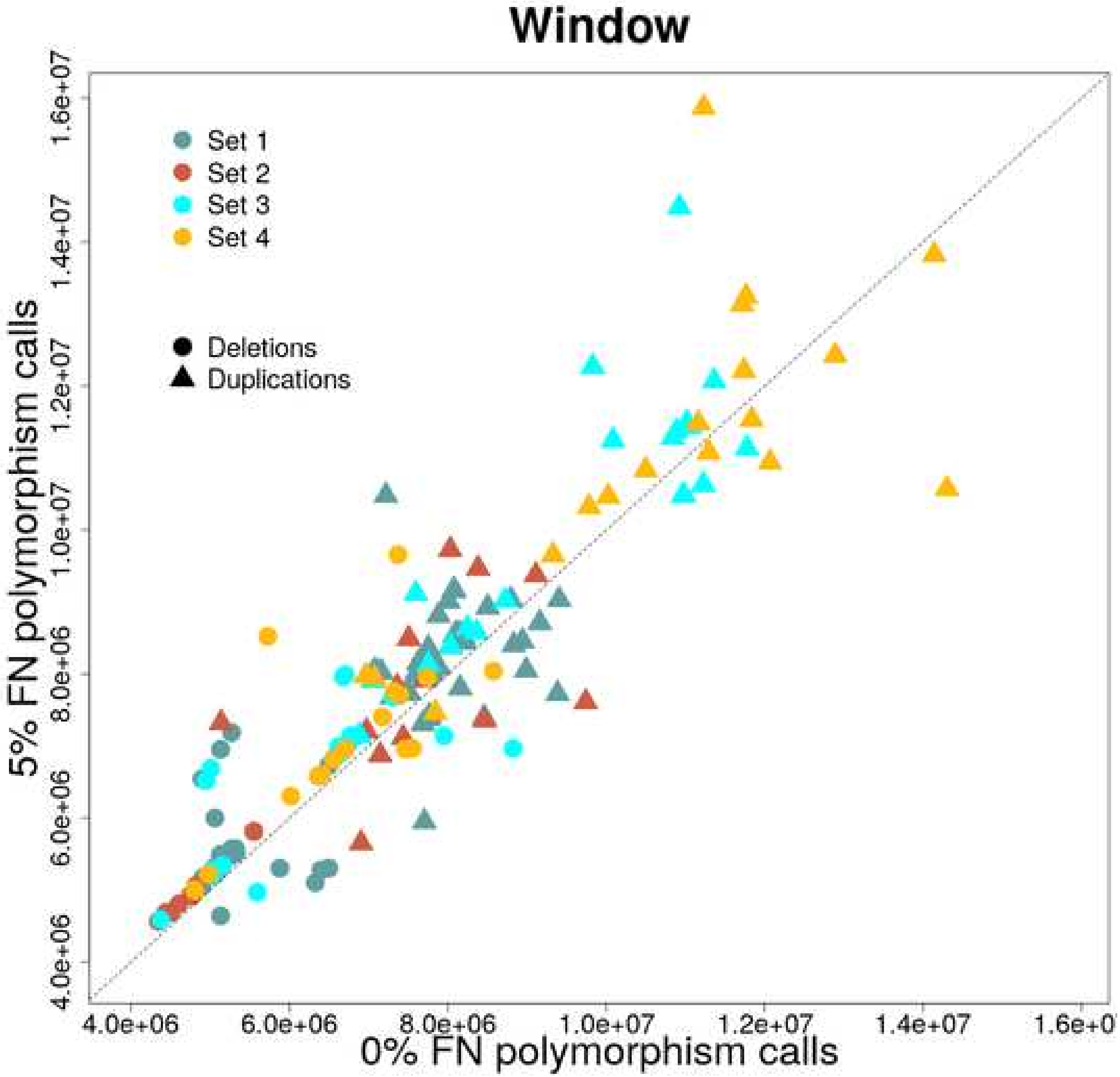

Click here to access/download;LaTeX - Other (clo, cls, sty etc.);FigS12b.png

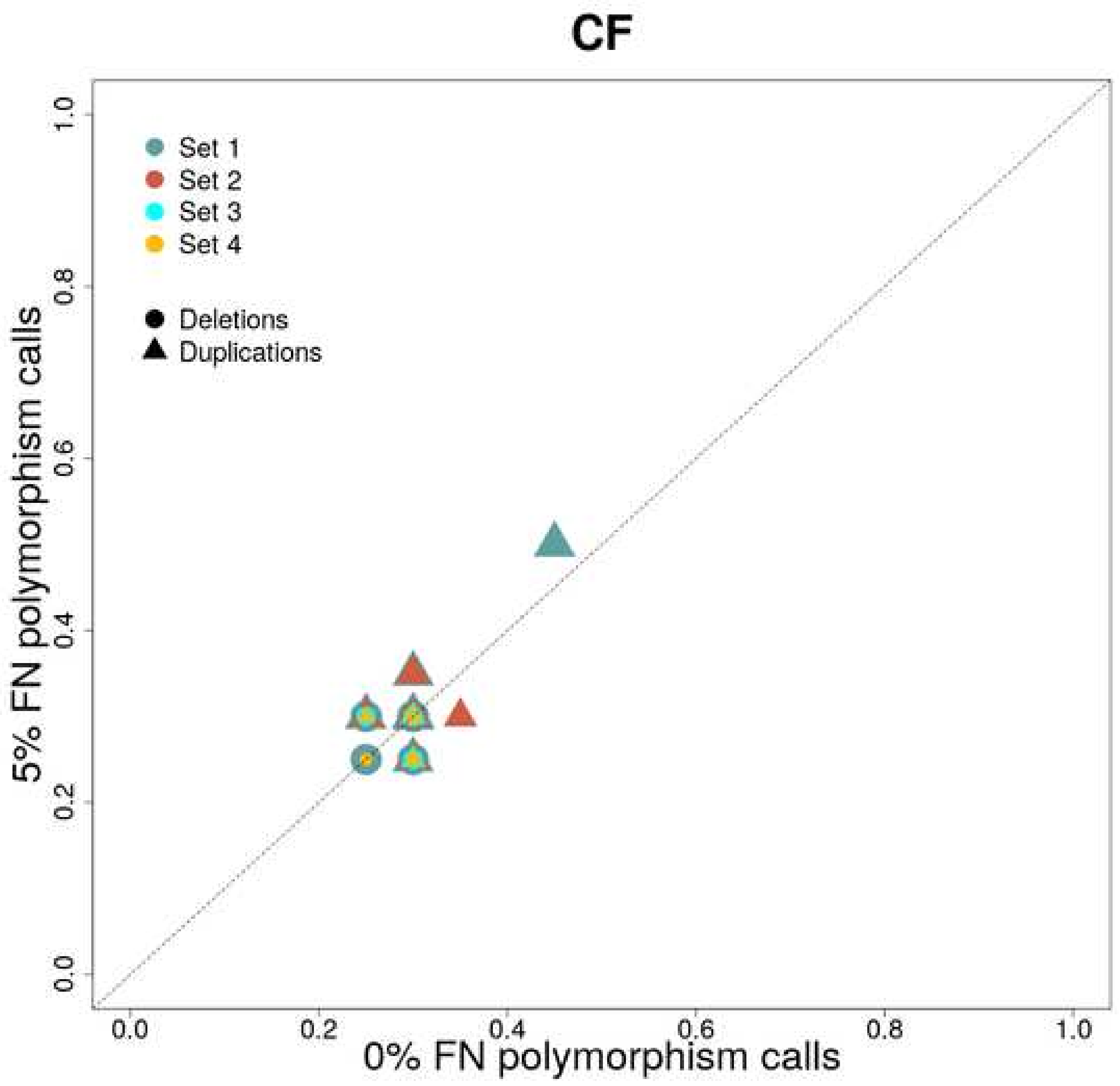

Figure for Latex file Supplement.tex
Click here to access/download;LaTeX - Other (clo, cls, sty etc.);FigS13a.png

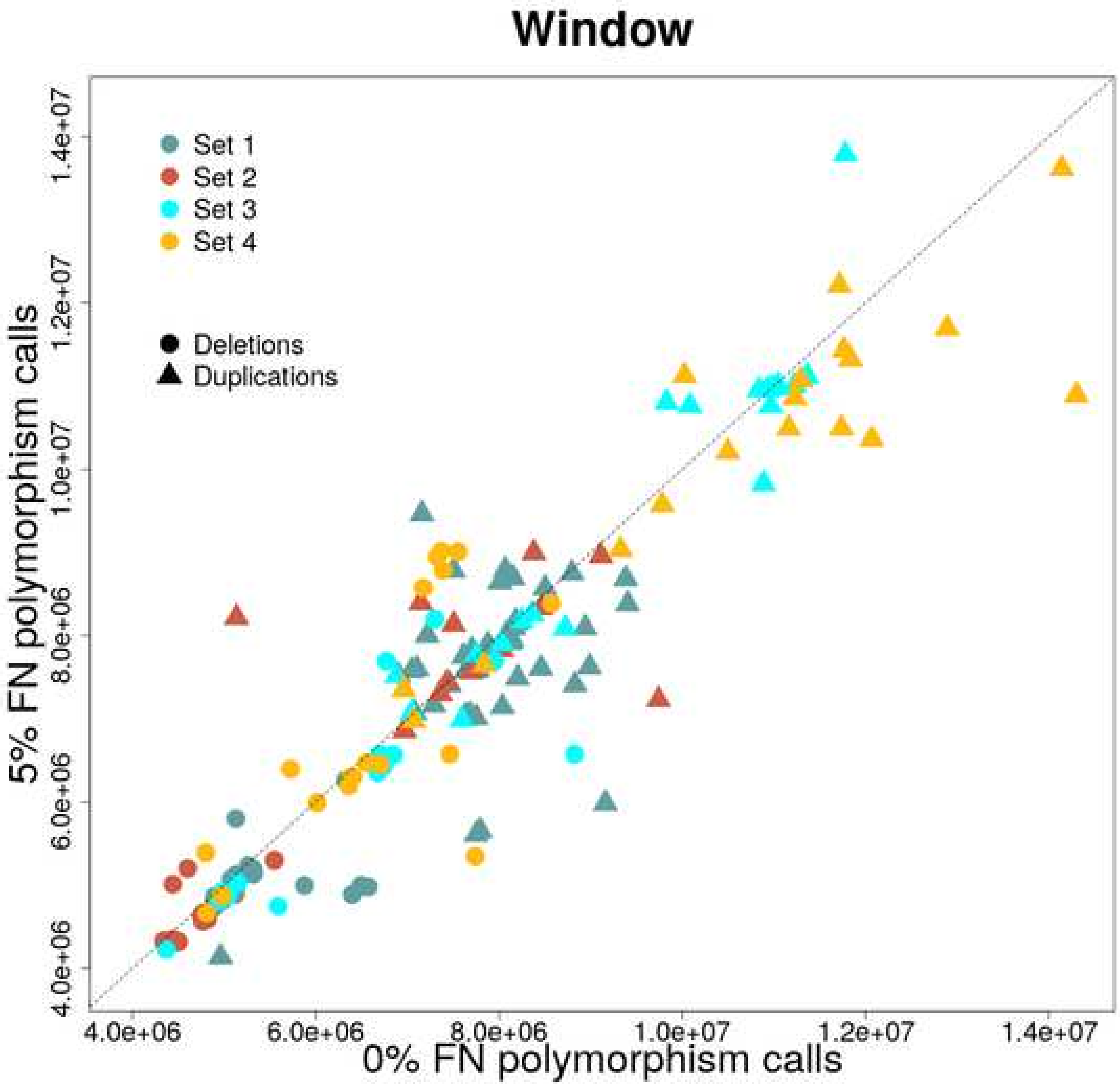

Figure for Latex file Supplement.tex
Click here to access/download;LaTeX - Other (clo, cls, sty etc.);FigS13b.png

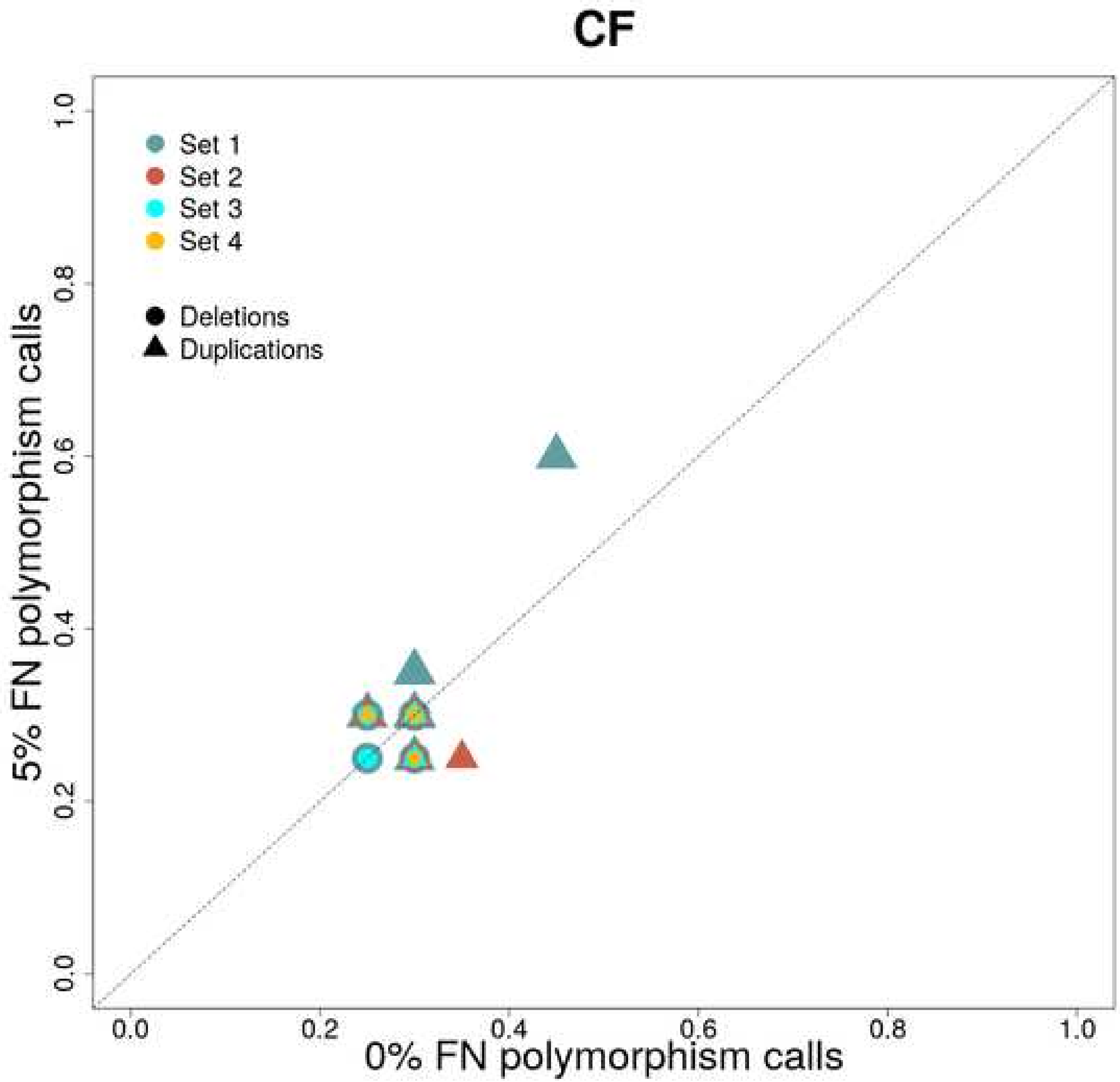

 Click here to access/download;LaTeX - Other (clo, cls, sty etc.);FigS2.png

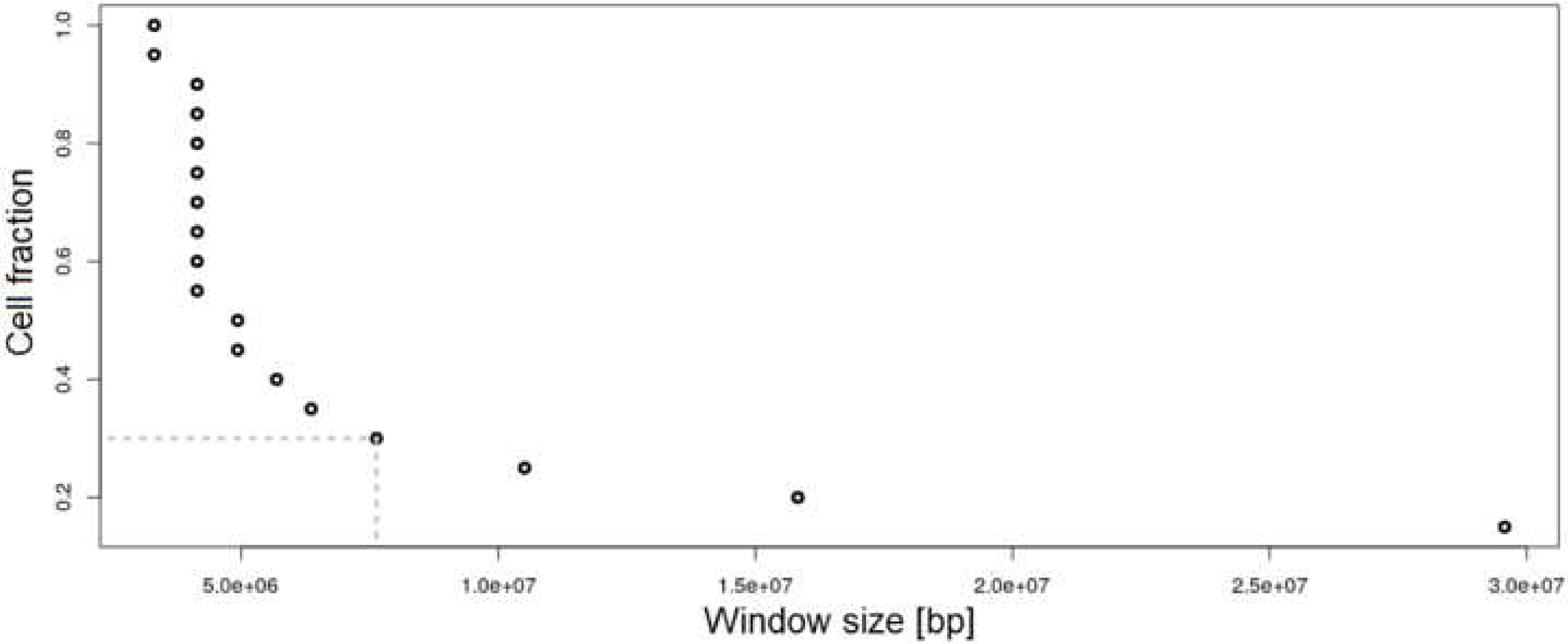

Figure for Latex file Supplement.tex
Click here to access/download;LaTeX - Other (clo, cls, sty etc.);FigS3.jpg

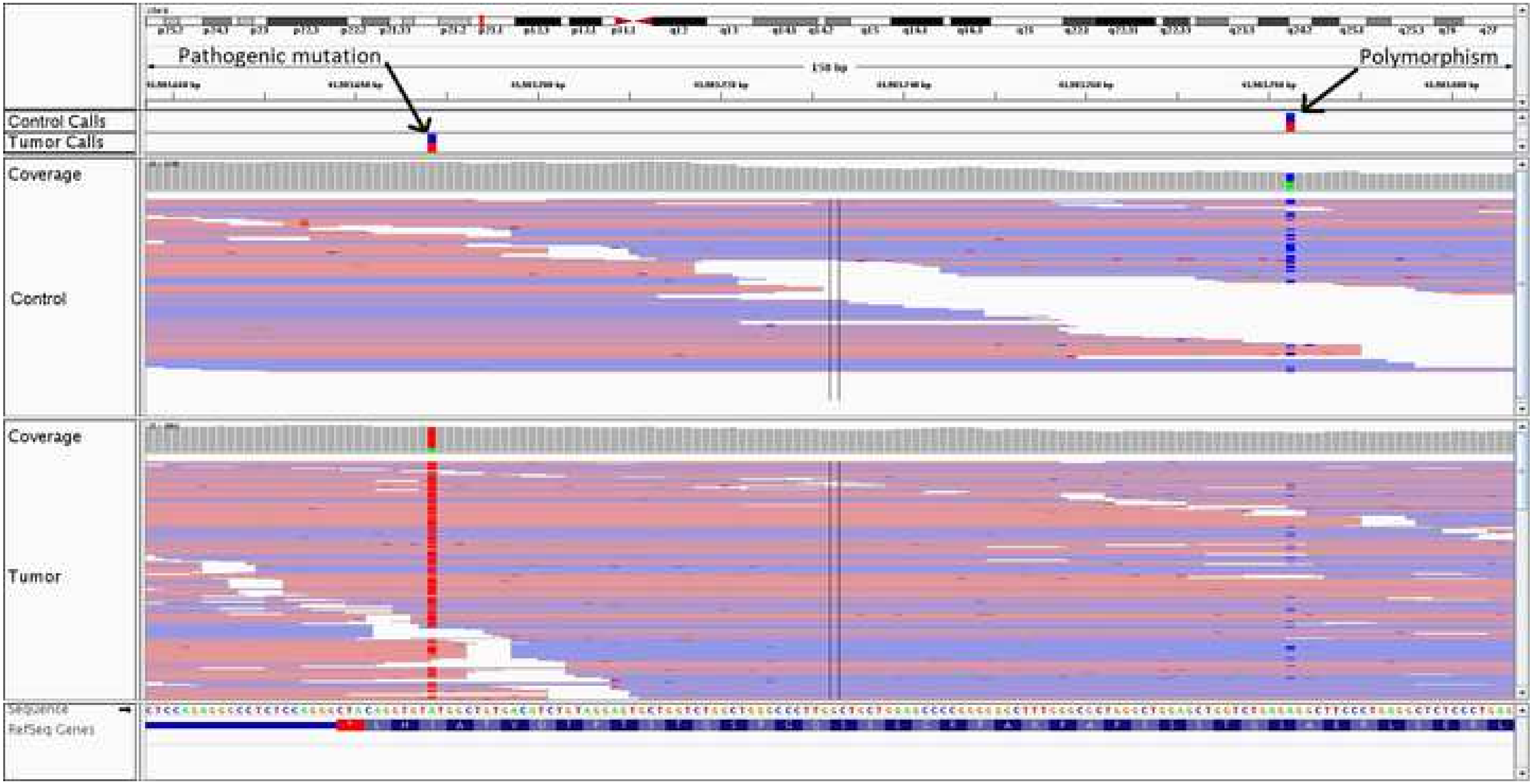

 Click here to access/download;LaTeX - Other (clo, cls, sty etc.);FigS4.jpg

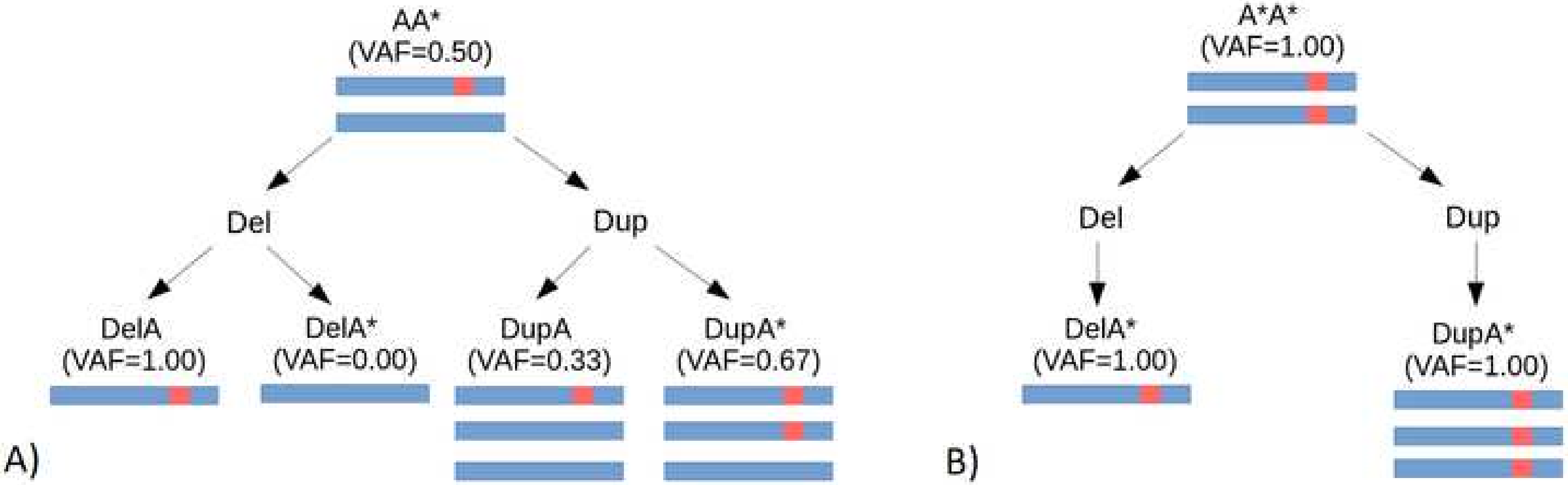

Figure for Latex file Supplement.tex

Click here to access/download;LaTeX - Other (clo, cls, sty etc.);FigS5.png

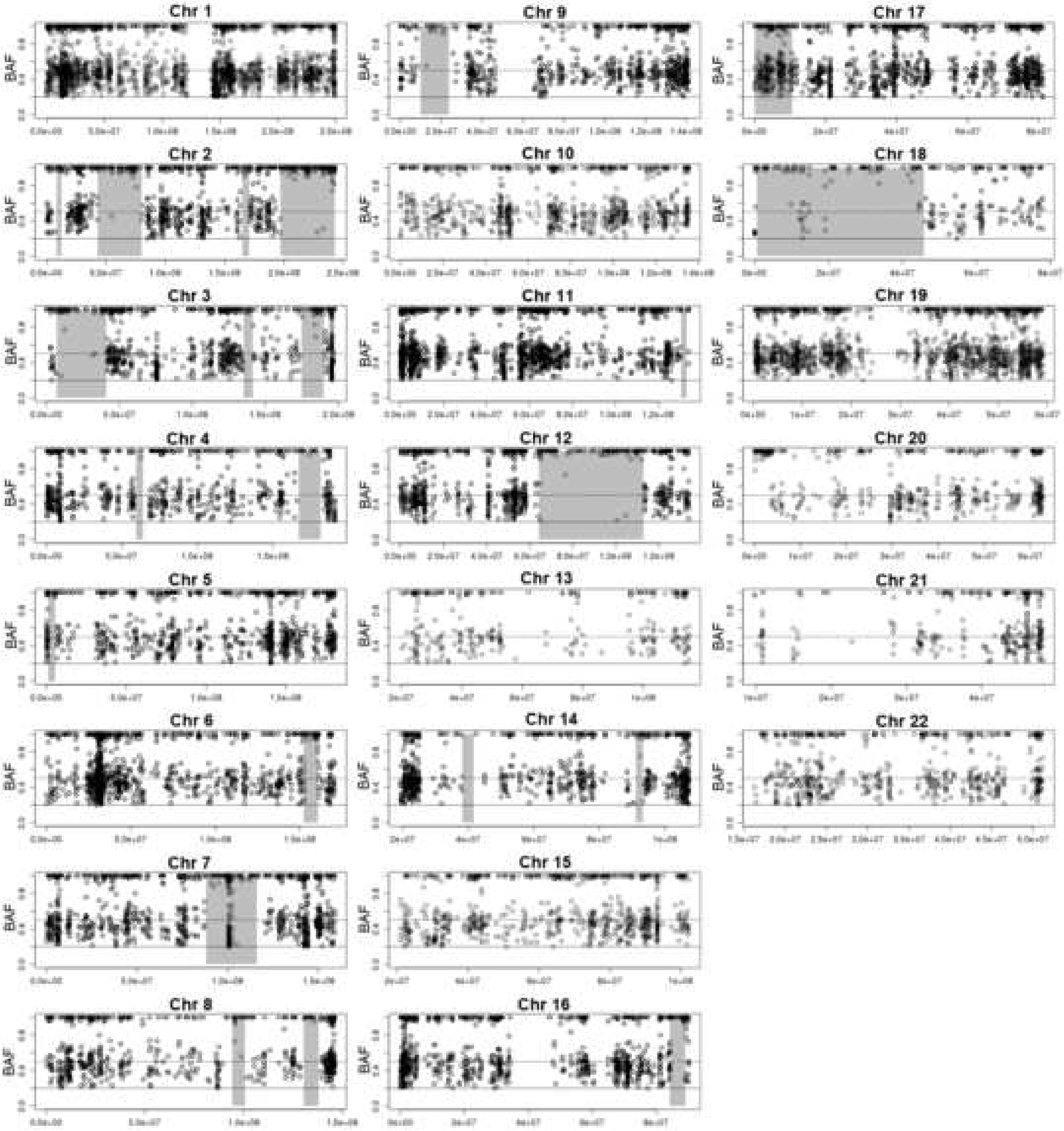

Figure for Latex file Supplement.tex Click here to access/download;LaTeX - Other (clo, cls, sty etc.);FigS5_2a.png

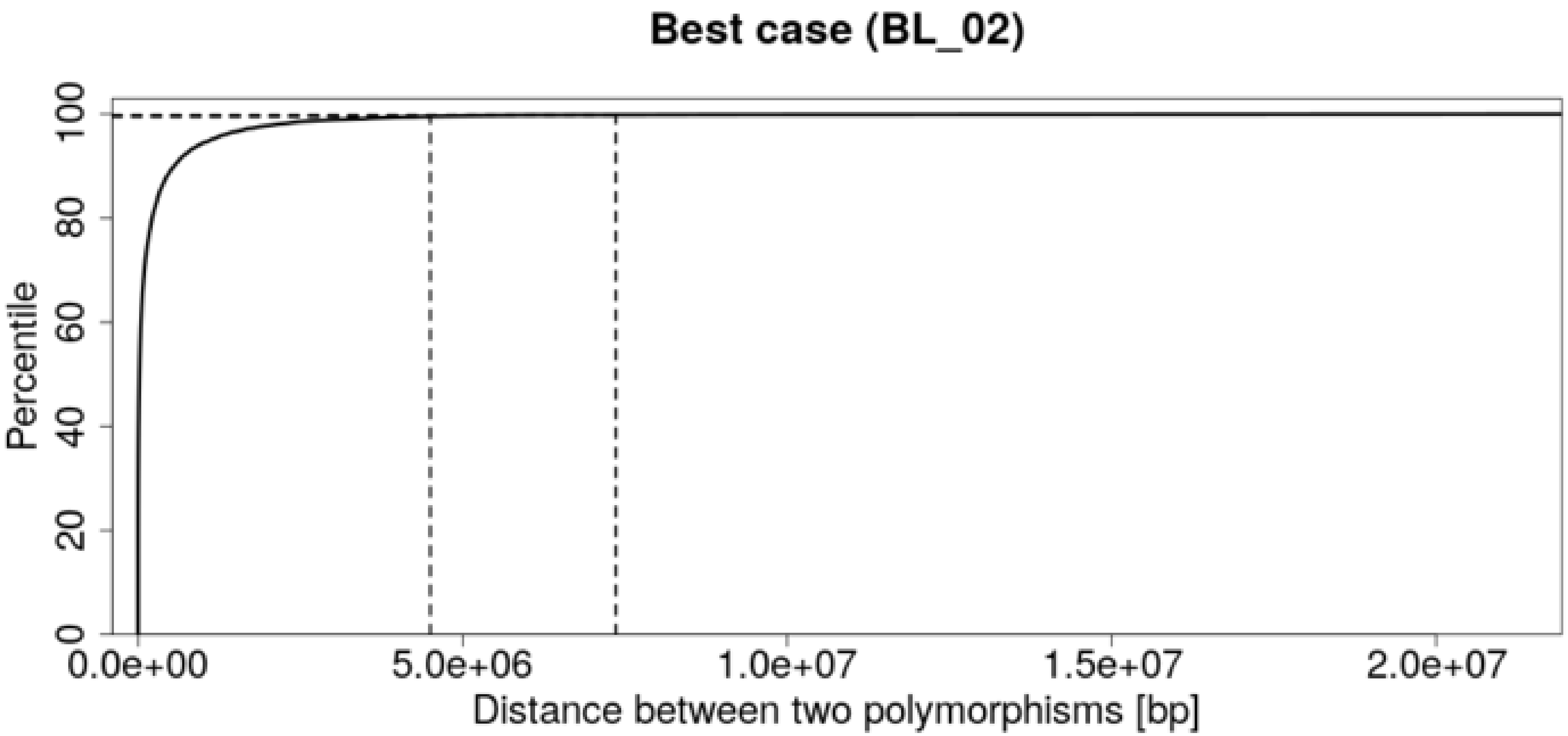

Click here to access/download;LaTeX - Other (clo, cls, sty etc.);FigS5_2b.png

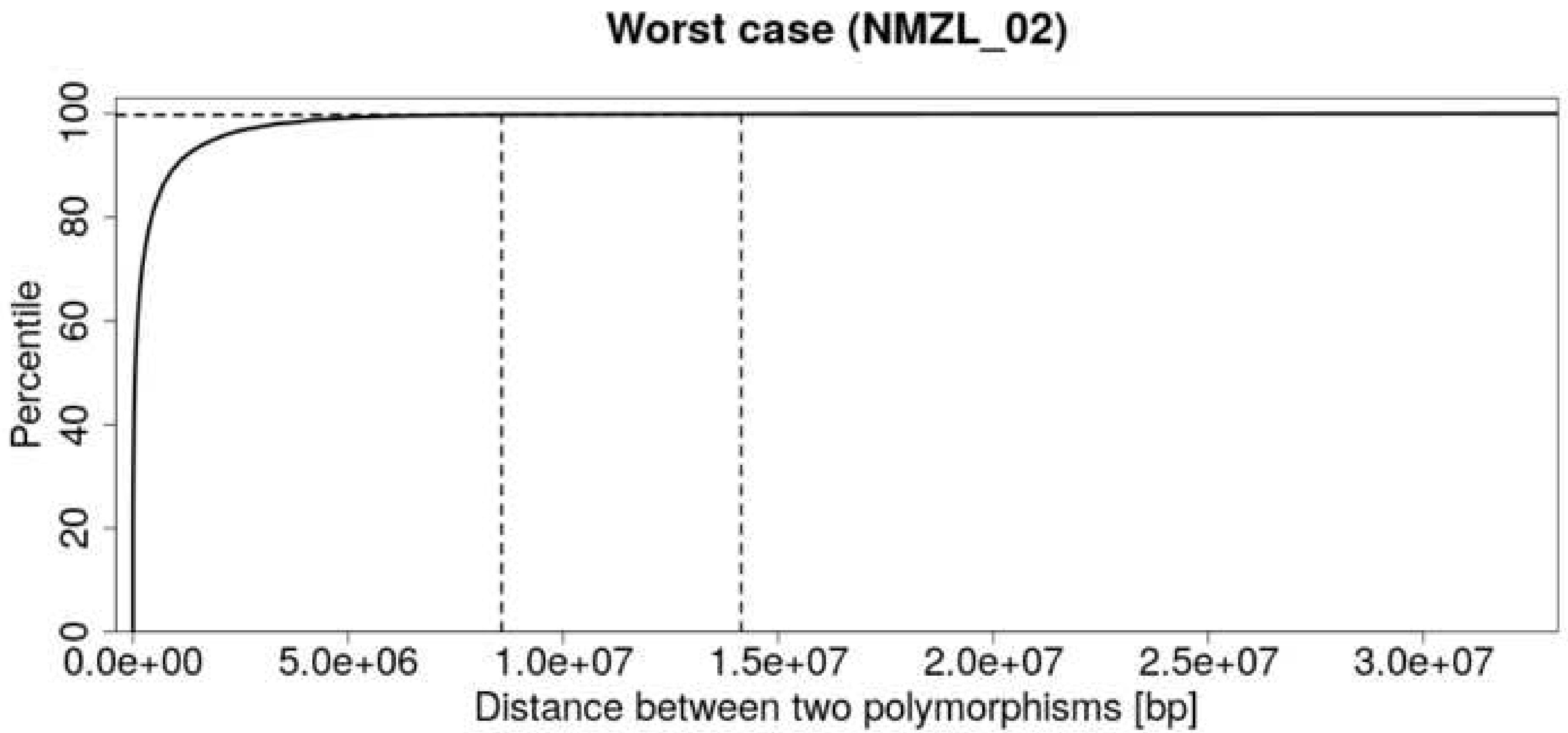

Figure for Latex file Supplement.tex
Click here to access/download;LaTeX - Other (clo, cls, sty etc.);FigS6.png

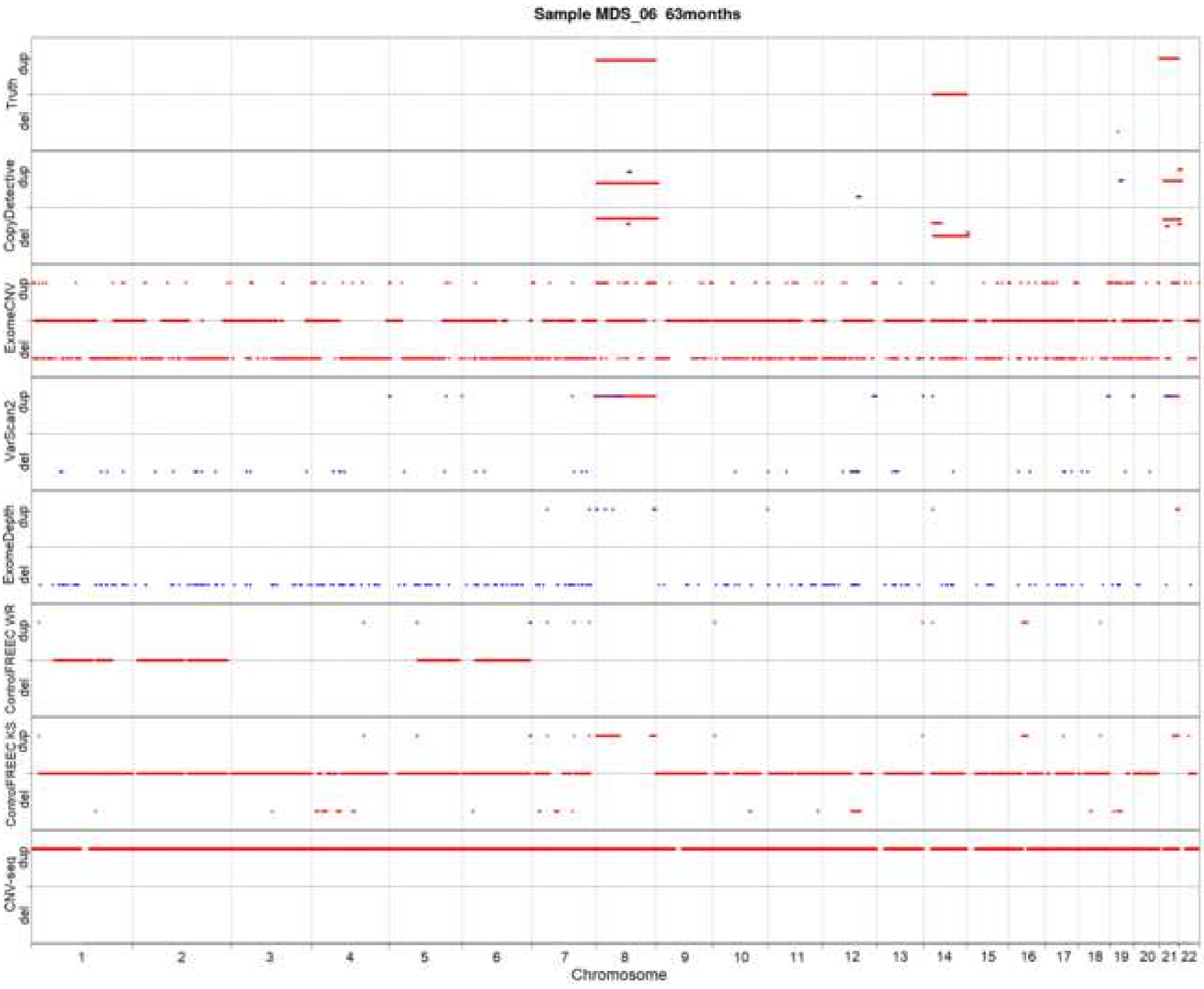

Figure for Latex file Supplement.tex
Click here to access/download;LaTeX - Other (clo, cls, sty etc.);FigS6_2a.png

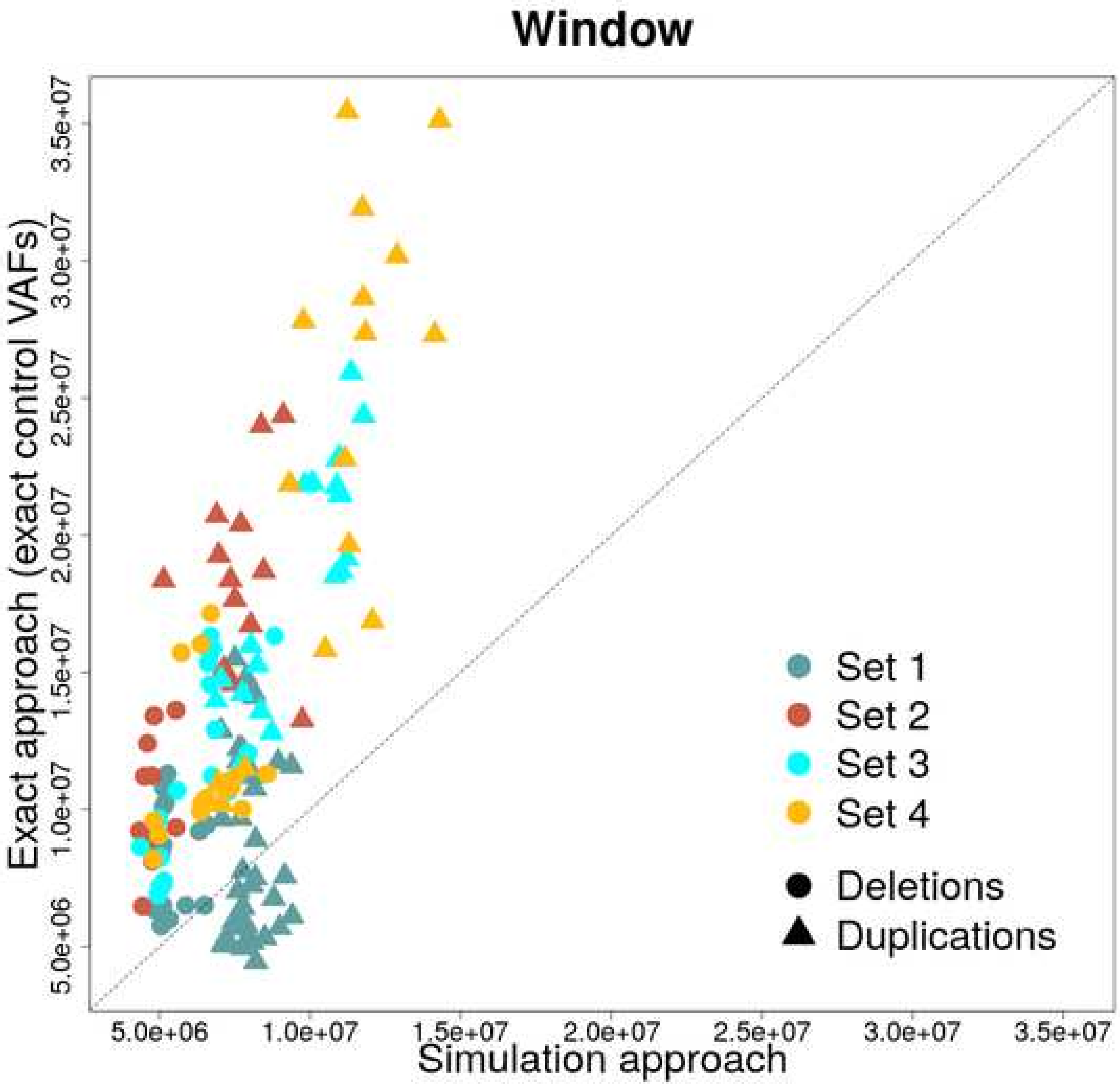

Figure for Latex file Supplement.tex
Click here to access/download;LaTeX - Other (clo, cls, sty etc.);FigS6_2b.png

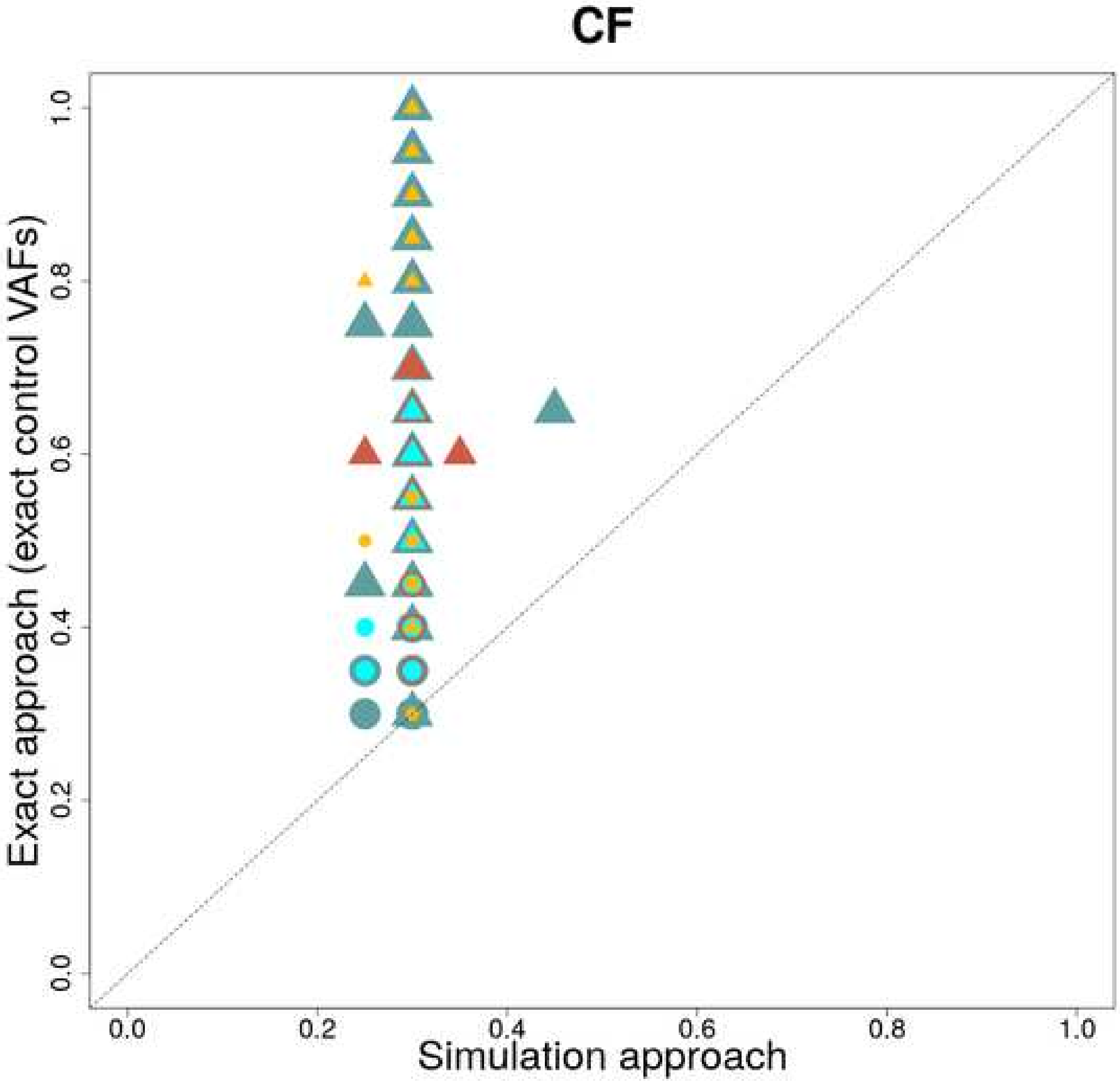

Figure for Latex file Supplement.tex
Click here to access/download;LaTeX - Other (clo, cls, sty etc.);FigS6_2c.png

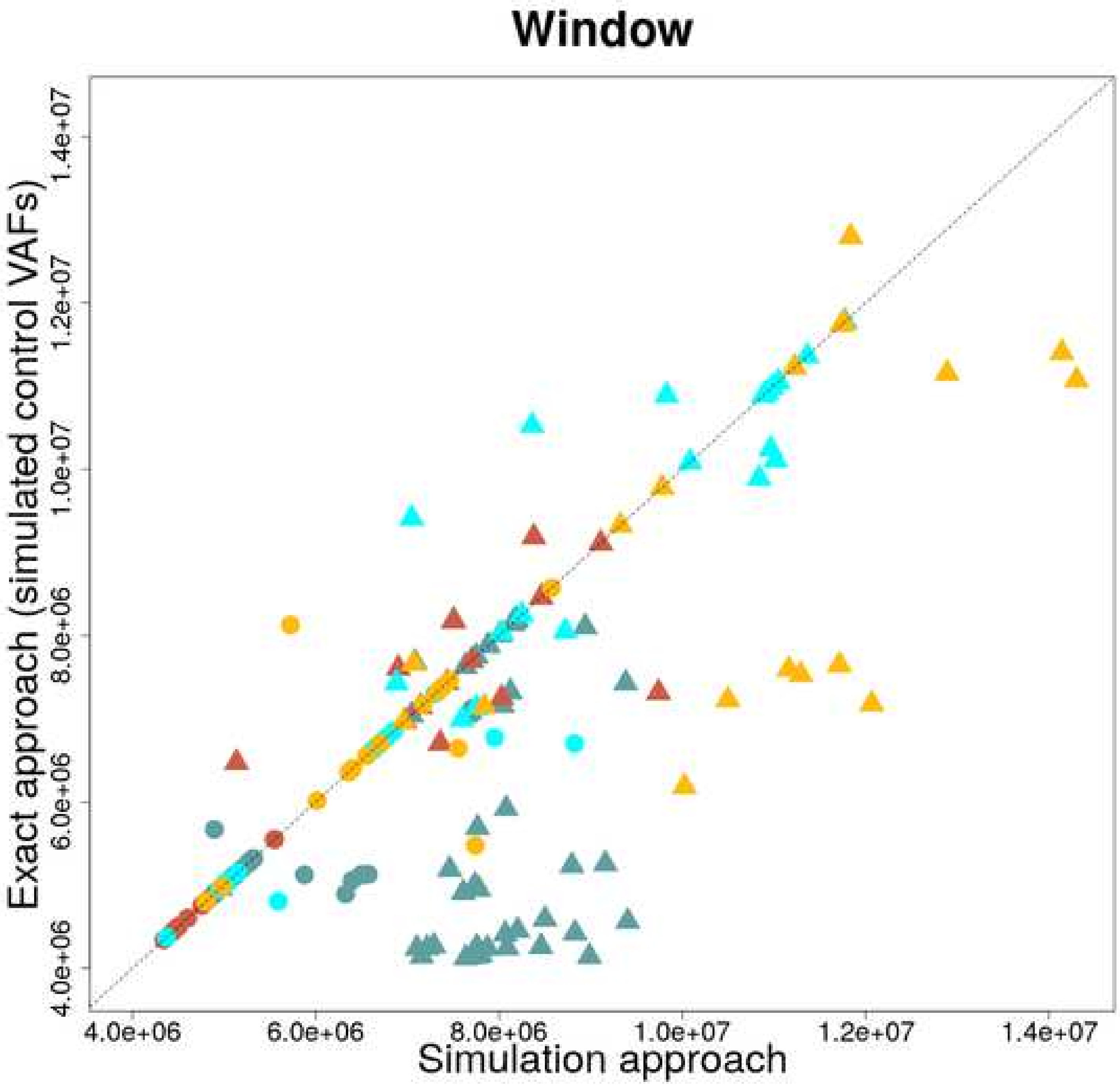

Figure for Latex file Supplement.tex
Click here to access/download;LaTeX - Other (clo, cls, sty etc.);FigS6_2d.png

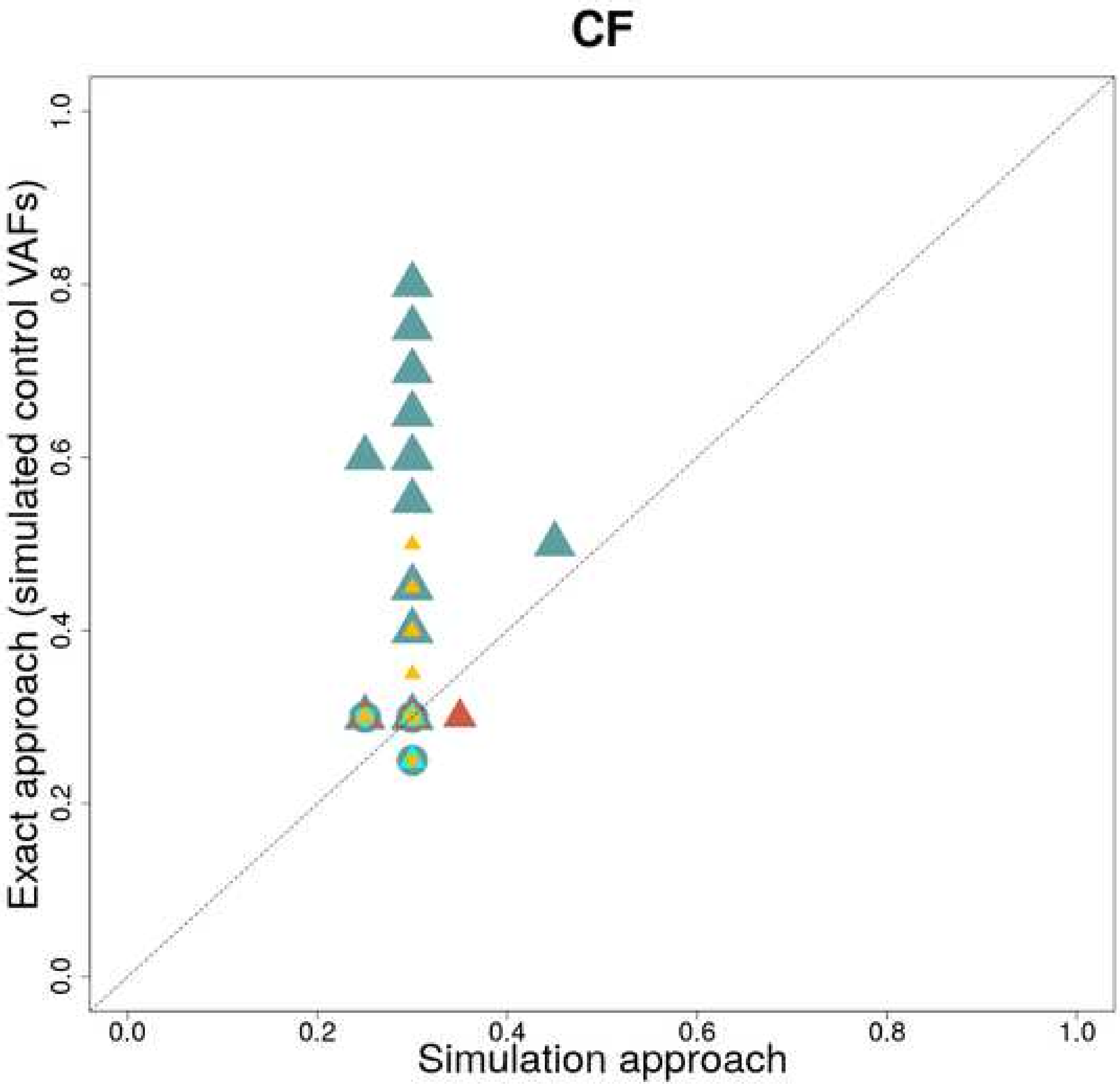

Figure for Latex file Supplement.tex
Click here to access/download;LaTeX - Other (clo, cls, sty etc.);FigS7.png

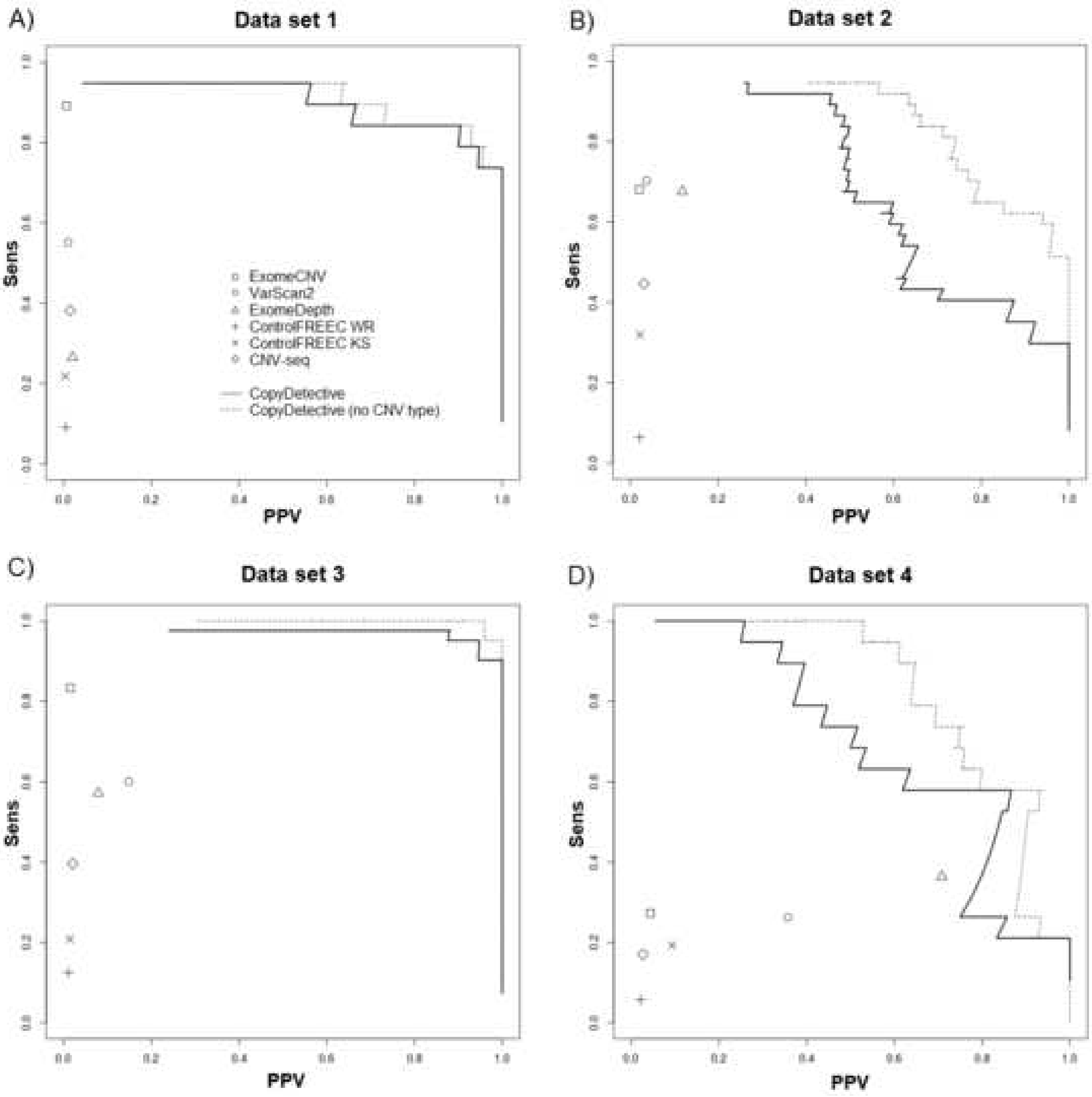

Figure for Latex file Supplement.tex

Click here to access/download;LaTeX - Other (clo, cls, sty etc.);FigS8A.png

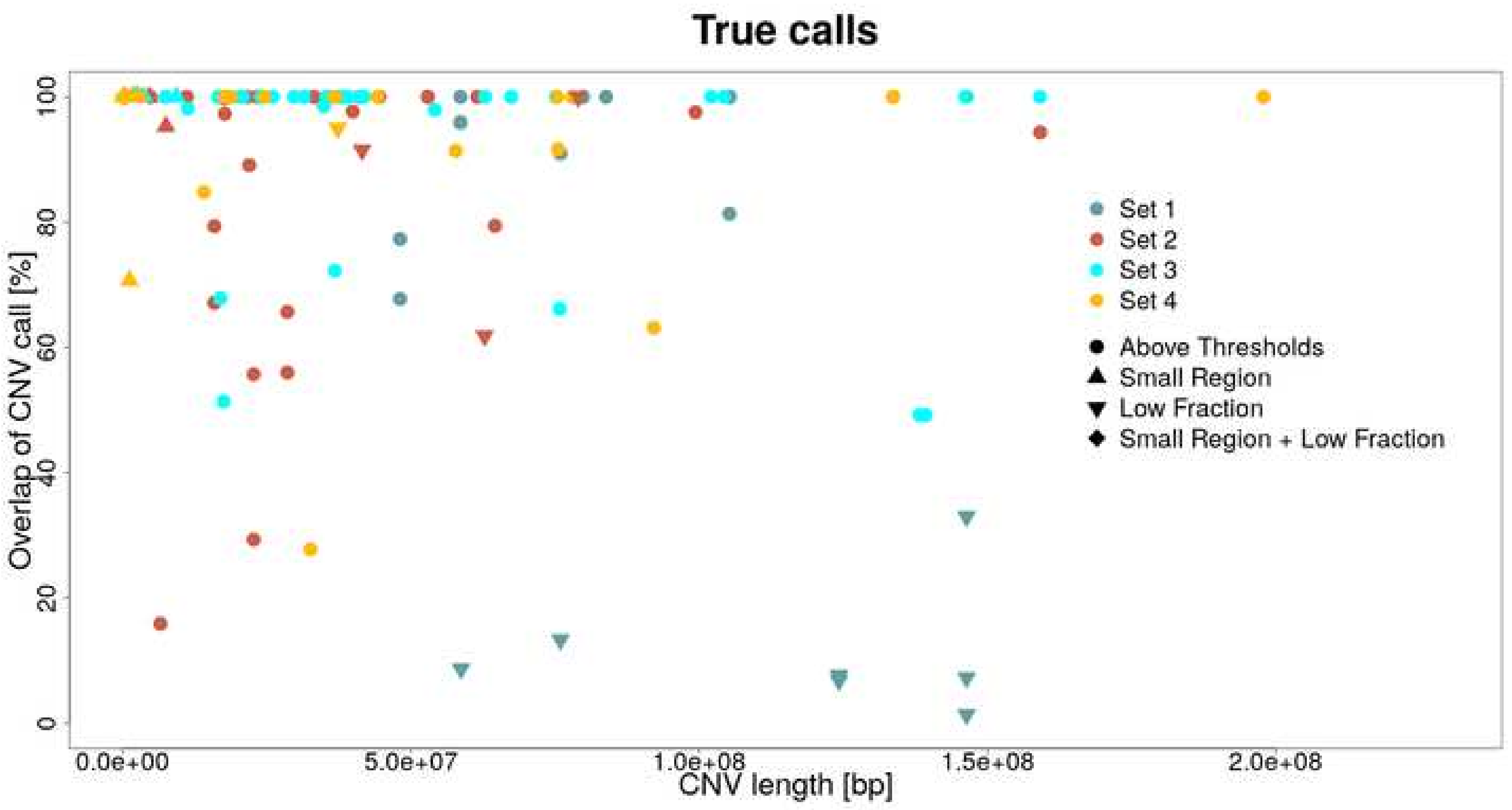

Click here to access/download;LaTeX - Other (clo, cls, sty etc.);FigS8B.png

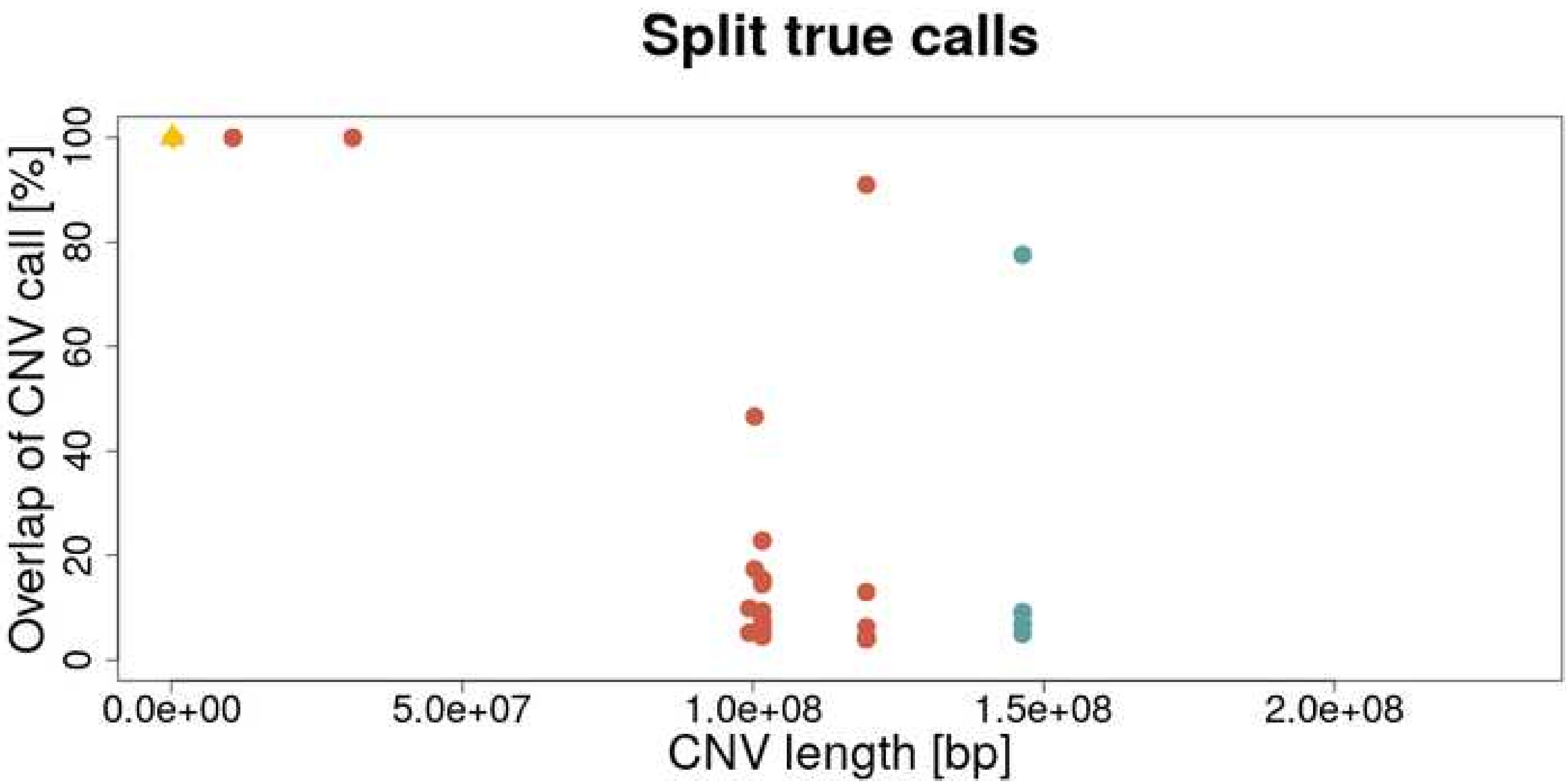

Figure for Latex file Supplement.tex Click here to access/download;LaTeX - Other (clo, cls, sty etc.);FigS8C.png

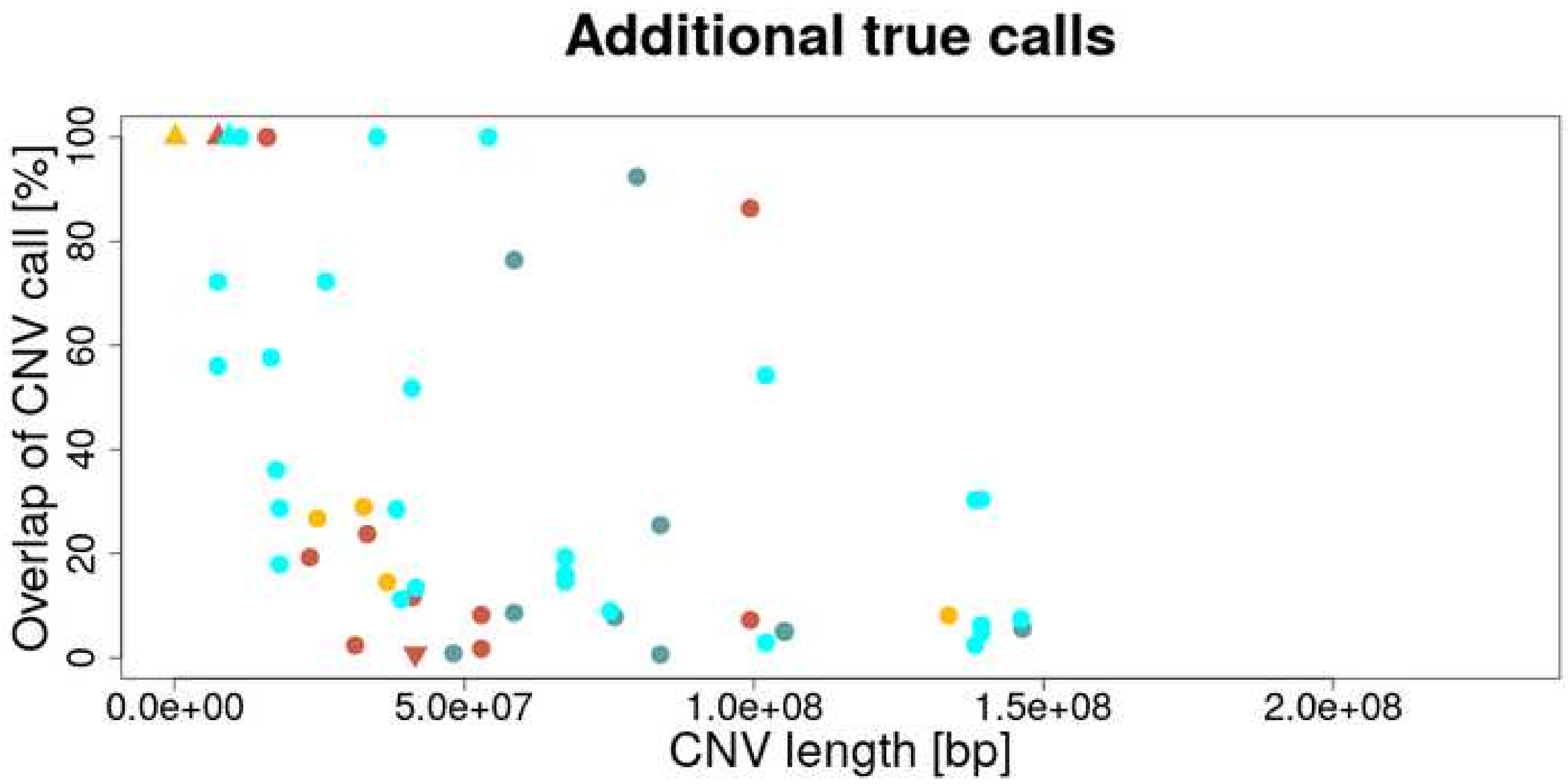

Click here to access/download;LaTeX - Other (clo, cls, sty etc.);FigS9A.png

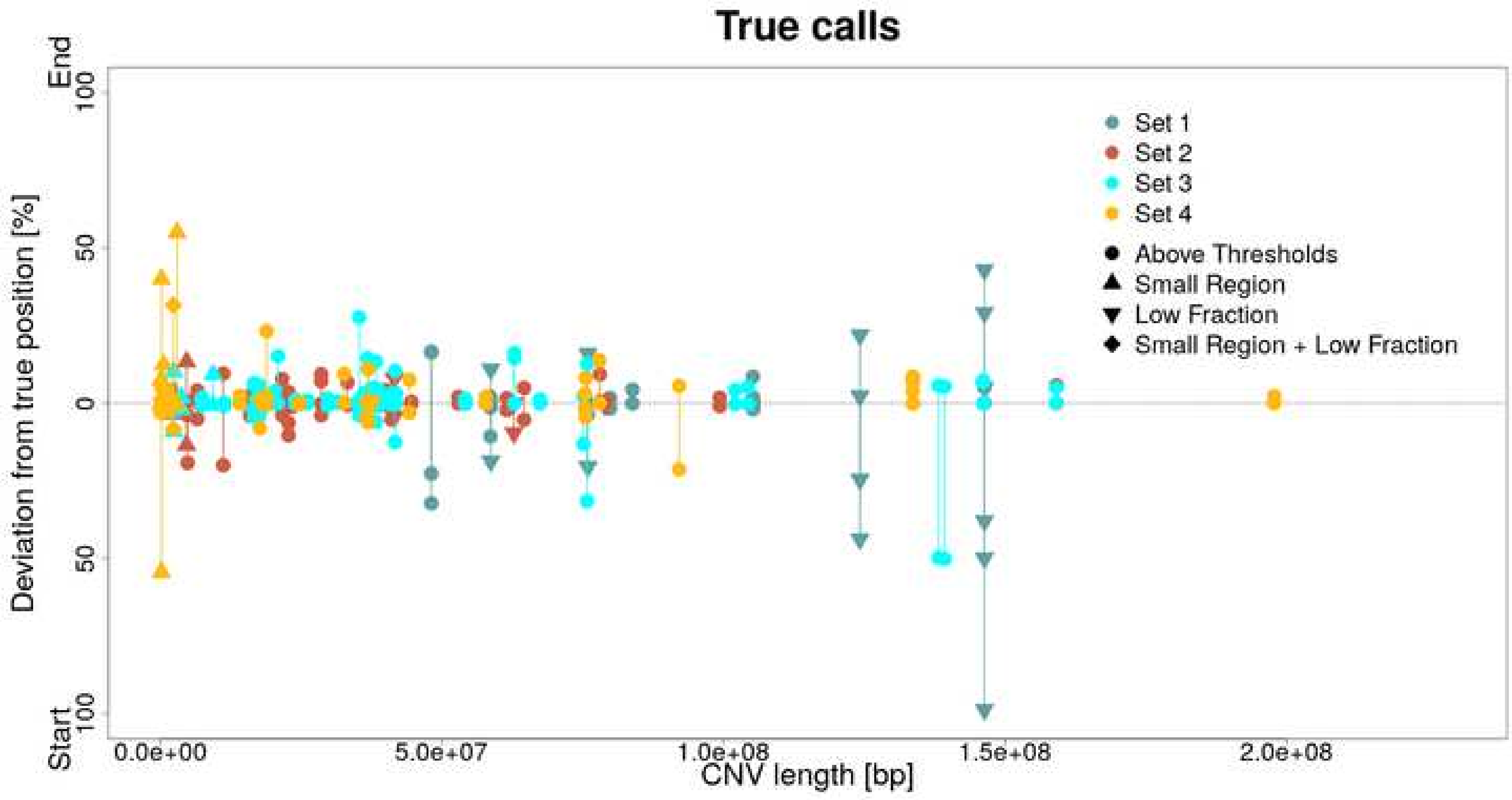

Figure for Latex file Supplement.tex Click here to access/download;LaTeX - Other (clo, cls, sty etc.);FigS9B.png

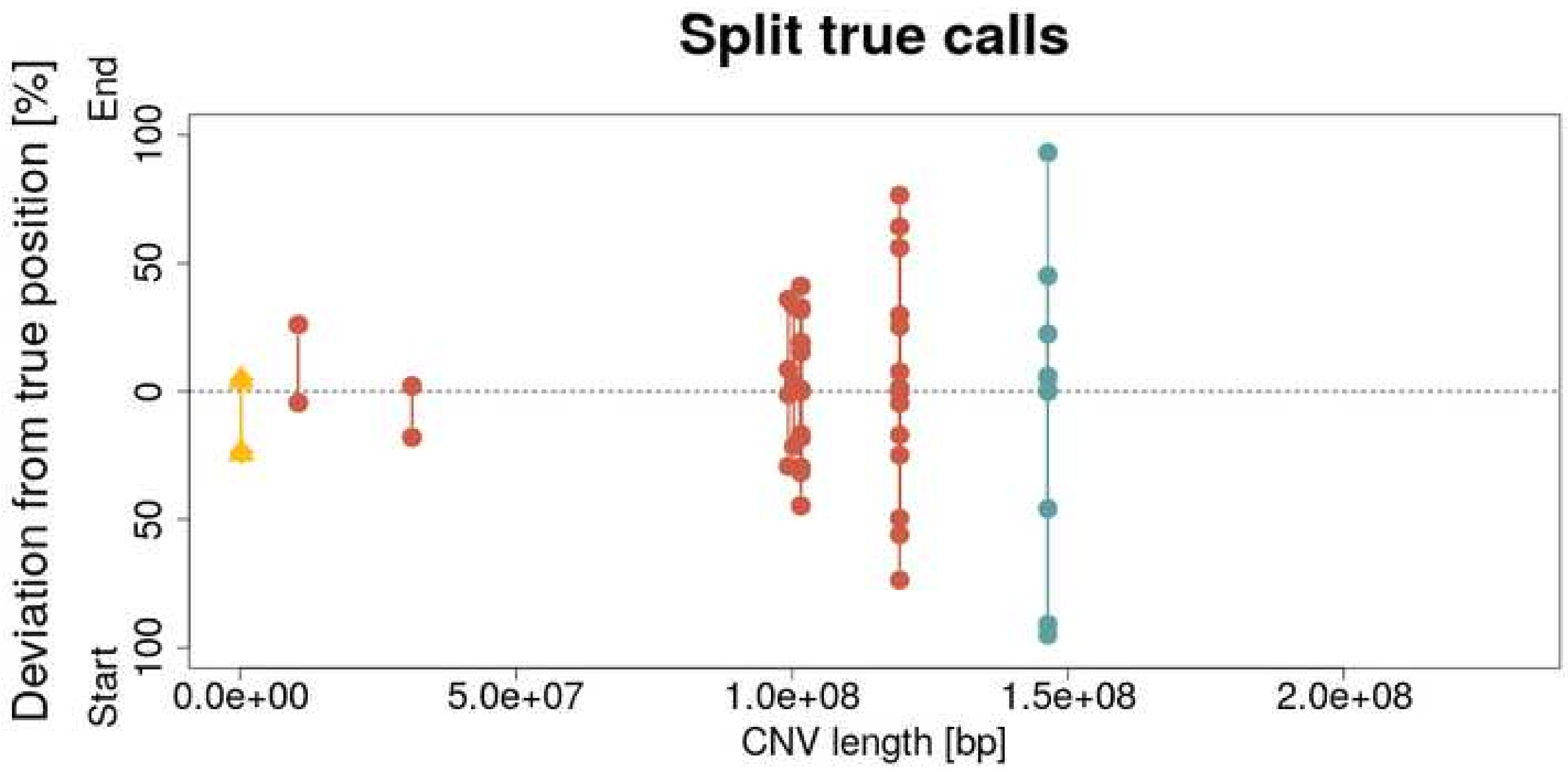

 Click here to access/download;LaTeX - Other (clo, cls, sty etc.);FigS9C.png

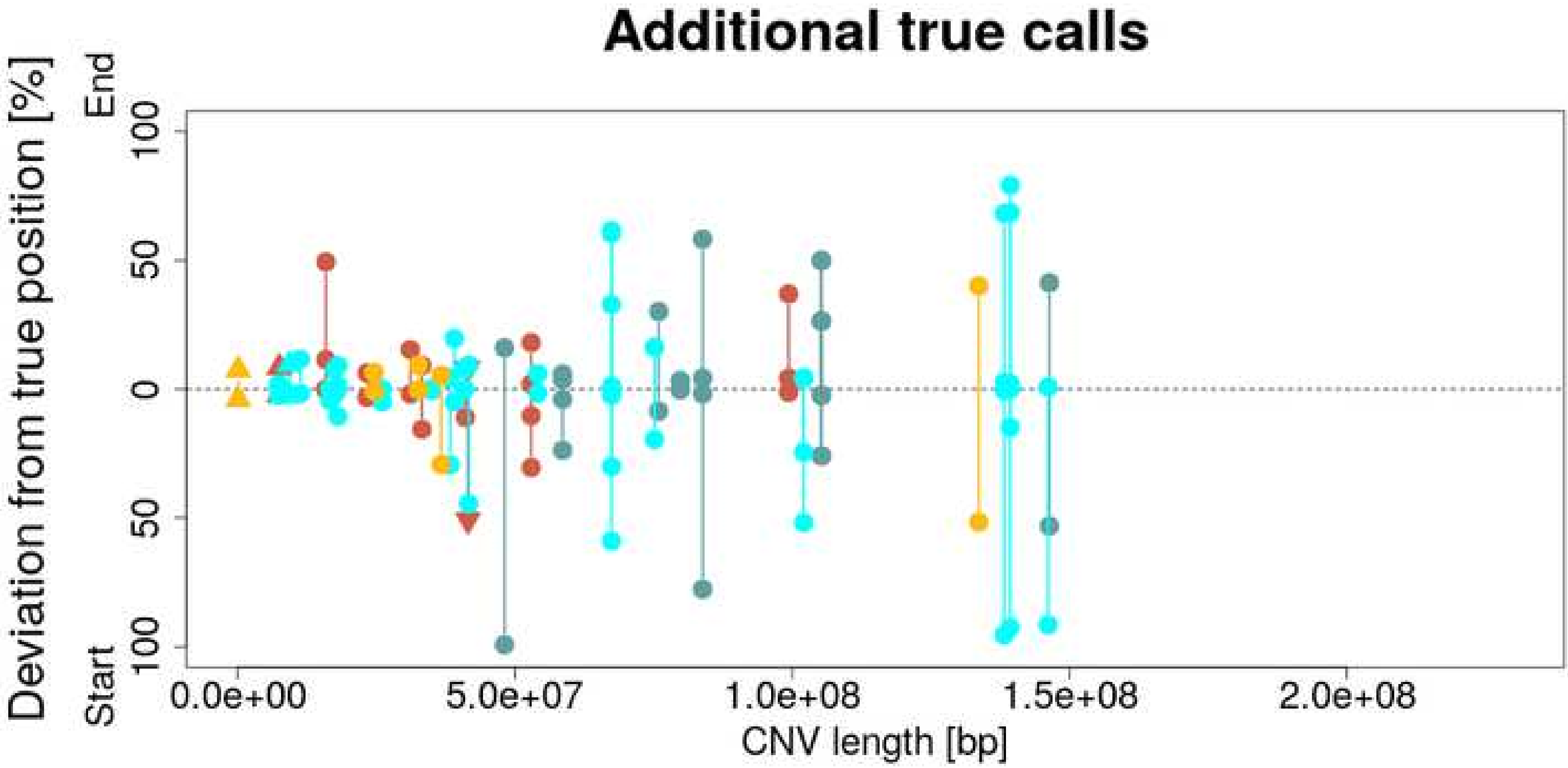

Manuscript
Click here to access/download;Manuscript;CopyDetective_GigScience.pdf

OXFORD
(GIGA)^n SCIENCE

GigaScience, 2017, 1–10

doi: xx.xxxx/xxxx
Manuscript in Preparation
Technical Note

TECHNICAL NOTE

CopyDetective: Detection Threshold Aware CNV Calling in WES Data

Sarah Sandmann[1,*], Marius Wöste[1], Aniek O de Graaf[2], Birgit Burkhardt[3], Joop H Jansen[2] and Martin Dugas[1]

[1]Institute of Medical Informatics, University of Münster, Münster, 48149, Germany, and [2]Laboratory Hematology, RadboudUMC, Nijmegen, 6525 GA, Netherlands, and [3]Paediatric Hematology & Oncology, University Hospital Münster, Münster, 48149, Germany.

*sarah.sandmann@uni-muenster.de; +49 251 83 55270

ORCIDs:
Sarah Sandmann, 0000-0002-5011-0641; Marius Wöste, 0000-0003-4994-8380; Martin Dugas, 0000-0001-9740-0788.

Abstract

Background: Copy number variants (CNVs) are known to play an important role in the development and progression of several diseases. However, detection of CNVs with whole-exome sequencing experiments is challenging. Usually, additional experiments have to be performed. **Results:** We developed a novel algorithm for somatic CNV calling in matched WES data called 'CopyDetective'. Different from other approaches, CNV calling with CopyDetective consists of a 2-step procedure: first, quality analysis is performed, determining individual detection thresholds for every sample. Second, actual CNV calling on the basis of the previously determined thresholds is performed. Our algorithm evaluates the change in variant allele frequency of polymorphisms and reports the fraction of affected cells for every CNV. Analyzing four WES data sets (n=100) we observe superior performance of CopyDetective compared to ExomeCNV, VarScan2, ControlFREEC, ExomeDepth and CNV-seq. **Conclusions:** Individual detection thresholds reveal that not every WES data set is equally apt for CNV calling. Initial quality analyses, determining individual detection thresholds – as it is realized by CopyDetective –, can and should be performed prior to actual variant calling.

Key words: Copy number variant; polymorphism; cell fraction

Background

Over the last years, next-generation sequencing (NGS) has found its way to clinical routine [1]. With the sequencing costs still getting cheaper – currently working on the "$100 genome" [2] – whole-exome sequencing (WES) and whole-genome sequencing (WGS) are performed for an increasing number of patients to improve their diagnosis, prognosis and therapy by the help of personalized medicine [3, 4].

Despite continuously decreasing costs for experiments, it is desirable to keep the number of necessary genetic experiments to a minimum – not least because of limited tumor material [5, 6]. Thus, it would be most practical if there were valid algorithms to determine single-nucleotide variants (SNVs), short insertions and deletions (indels), structural variants (SVs) and copy number variants (CNVs) by just a single NGS-experiment.

Although there still remain challenges to be addressed, relatively short mutations – like SNVs and indels – can already be determined quite reliably [7, 8]. In contrast to this, large mutations like CNVs still impose a major challenge [9].

Numerous algorithms, all following different approaches, exist for calling CNVs in WES data. While some concentrate on normalizing coverage, e.g. VarScan [10], others analyze single-nucleotide polymorphisms (SNPs) and coverage information similar to SNP arrays, e.g. ExomeCNV [11]. Some algorithms require matched control samples, while others do not

Compiled on: September 24, 2020.
Draft manuscript prepared by the author.

Table 1. Overview of the samples analyzed with CopyDetective.

Data set	Disease		Samples	Mean coverage		Coverage ≥1x		Coverage ≥10x		Heterozygous
				Germline	Tumor	Germline	Tumor	Germline	Tumor	SNPs
1	MDS		47	114.90x	119.71x	99.28%	99.07%	97.29%	97.04%	10,543
2	BL	primary	10	44.21x	277.28x	97.73%	98.41%	89.66%	97.38%	11,884
		relapse	5	44.00x	298.79x	97.70%	98.41%	89.44%	97.75%	11,665
3	T-LBL	primary	15	60.25x	190.46x	94.62%	98.41%	84.23%	96.70%	9,659
		relapse	5	57.60x	290.15x	90.98%	98.52%	75.41%	97.97%	7,893
4	NMZL		18	41.63x	41.24x	99.08%	99.10%	78.61%	80.71%	9,341

require any controls. However, all of these algorithms usually suffer from low precision and low recall [9], which raises the question if NGS data from WES experiments is after all suitable to determine valid CNV calls. Or – to specify this question – if every data set is equally apt to determine every kind of CNV, independent of the number of base pairs or fraction of cells affected by the mutation. Especially in the field of cancer research this is highly relevant, specifically regarding cancer cell fractions (CCFs) and clonal evolution.

Considering SNV and indel calling in NGS data, it is obvious that every data set's characteristics define its individual detection thresholds. An essential characteristic is coverage. If data is sequenced with only 10x coverage, it is not apt to detect mutations at allelic frequencies of 5% as only 0.5 reads are expected to carry the mutation. When calling CNVs in NGS data, it is only consistent to assume that comparable detection thresholds exist.

We present a novel algorithm, performing detection threshold aware CNV calling in WES data: CopyDetective [12]. Prior to determining the actual CNVs, CopyDetective addresses data quality of every sample. We consider 1) coverage of the case sample, 2) coverage of the matching control sample, 3) CNV length and 4) CNV value with respect to the fraction of affected cells. For every sample, individual detection thresholds are determined. These thresholds define the minimum cell fraction (CF) and the minimum CNV length still being detectable at a given sensitivity.

Subsequently, CopyDetective analyzes data according to these thresholds. Comparing a case sample to its matching control sample, coverage and SNP information is evaluated to identify regions of significant difference. CopyDetective reports merged and filtered CNVs along with additional information on the calls, e.g. quality values and information on the estimated CF.

Analyzing four real WES data sets (n = 100) the performance of our novel approach is evaluated and compared to five established approaches for CNV calling in WES data: ExomeCNV [11], VarScan2 [10], ExomeDepth [13], Control-FREEC [14] and CNV-seq[15].

Methods

Data sets analyzed

We analyze four data sets, covering real data from n = 100 samples. An overview of the different data sets and the available samples can be found in Table 1.

The first set covers 47 samples from 11 patients with myelodysplastic syndromes (MDS; sequencing data published at the NCBI Sequence Read Archive PRJNA355124). All patients have been sequenced 2 to 8 times. CNV calling results based on CytoScan HD Array (Affymetrix) – containing information on deletions, duplications and loss of heterozygosity (LOH) – have been published. Additionally, information on clonal evolution of all patients has been published by da Silva et al. [16]. Thus, CNV value can be considered with respect to CF. For example: a simple duplication leads to CNV value= 3. However, if the mutation is just present in 60% of the cells, the CNV value over all cells would be 2.6.

The second set covers 15 samples from 10 patients with Burkitt lymphoma (BL; sequencing data published at the NCBI Sequence Read Archive PRJNA561490). Five out of 10 patients have been sequenced twice – at the point of primary and relapse. The remaining 5 patients did not relapse. Only one tumor sample (primary) is available. CNV calling results (deletions, duplications and LOH) are based on SNP arrays (Infinium OmniExpressExome-8v1.3kit; using Illumina GenomeStudio 2.0 (GenomeStudio, RRID:SCR_010973) and cnvPartition v3.2.0 (CNVPartition, RRID:SCR_010925), minimum 100 probes for one call, for analysis) [17]. Additionally, clonal evolution was reconstructed for all patients. Again, CNV value with respect to CF can be considered.

The third set covers 20 samples from 15 patients with T-lymphoblastic lymphoma (T-LBL; sequencing data published at the EMBL-EBI European Nucleotide Archive PRJEB36436). Five out of 15 patients have been sequenced twice – at the point of primary and relapse. As the remaining 10 patients did not relapse, only one tumor sample (primary) is available. CNV calling results (deletions, duplications and LOH) are based on SNP arrays (InfiniumOmni2-5Exome-8; using Illumina GenomeStudio 2.0 (GenomeStudio, RRID:SCR_010973) and cnvPartition v3.2.0 (CNVPartition, RRID:SCR_010925), minimum 100 probes for one call [18]; data published at Array Express E-MTAB-8763; manuscript under review).

The fourth data set covers 18 samples with nodal marginal zone lymphoma (NMZL; sequencing data published at the NCBI Sequence Read Archive PRJNA285732 [19]). CNV data has been deposited in Gene Expression Omnibus (accession number GSE68078; CytoScan HD Array; Affymetrix). Analysis of the CNV data was performed using Rawcopy [20]. Raw calls with a missing allelic imbalance, or an imbalance < 0.2 were removed. The remaining calls were merged if they were located close to each other (<20 Mbp) and characterized by a similar logR-ratio (2^{logR} <0.2). The resulting CNV calls were classified as deletions if CNV value≤1.9, and as duplications if the CNV value≥2.1. The remaining calls were classified as LOH.

For all samples, detailed information on data quality can be found in Additional file 1, section 1.1 (Tables S1-S4).

CopyDetective

CopyDetective is a novel algorithm for calling somatic CNVs in matched WES samples, automatically determining and evaluating individual detection thresholds for every sample. The analysis with CopyDetective can be separated into four major steps: 1) quality analysis, 2) CNV calling, 3) merging, and optional 4) filtration. An overview of the analysis is provided in

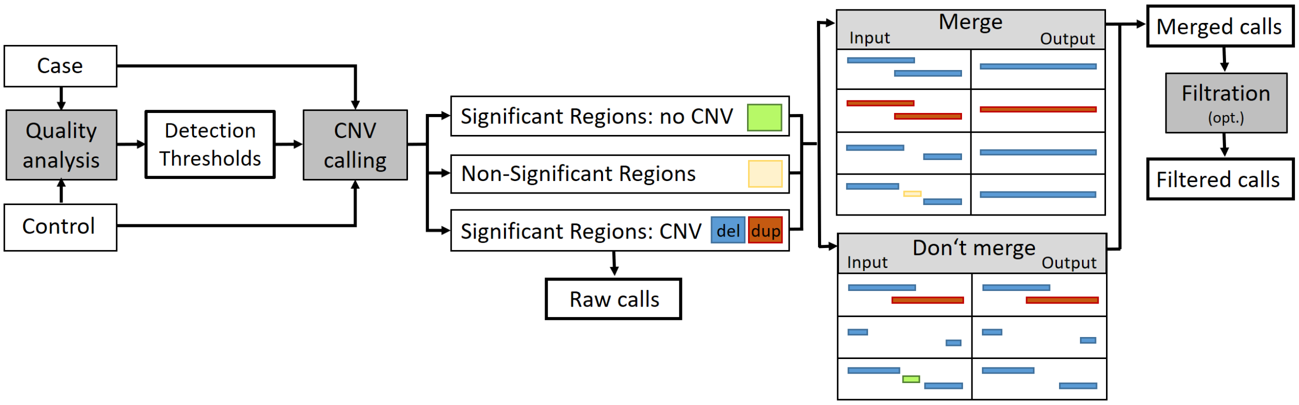

Figure 1. Overview of the analysis performed by CopyDetective. The analysis can be separated into four major steps: 1) Quality analysis: the detection thresholds for CNV calling are estimated. 2) CNV calling: significant regions with and without CNV are determined. 3) Merging of overlapping and adjacent regions with CNV. 4) Optional filtration.

Figure 1.

Quality analysis

Different from other CNV calling algorithms, an initial analysis of data quality is automatically performed by CopyDetective to determine individual detection thresholds for every sample. These thresholds include the minimum CFs for deletions and duplications – CF_{Del} and CF_{Dup} – and the minimum CNV lengths – W_{Del} and W_{Dup}. An overview of CopyDetective's quality analysis, which is performed for both deletions and duplications, is provided in Figure 2.

Quality analysis itself is split into three steps: First, CFs are considered (see Figure 2A). Our analysis is based on the actual coverage of all heterozygous polymorphisms detected in a matching case-control-pair. Analyses show that coverage distribution can be approximated by a log-normal distribution (see exemplary coverage distribution of patient MDS_01 germline in Additional file 1, section 1.2.1, Figure S1). We evaluate all CFs in a user-defined range (default: 5-100% with steps of 5%; smaller step sizes are possible) for deletions and duplications separately. The user can choose between a simulation approach (default) and an exact approach. For the simulation approach, an artificial case-control-pair is considered. To keep run-time low, we do not simulate the actual reads. Instead, just the coverage and variant allele frequency (VAF) of heterozygous polymorphisms are simulated (default: 1 to 100 SNPs). In the control sample, the expected value for VAF is 50%. In the case sample, VAF is dependent on CF: If $CF = 100\%$ the expected value for VAF is 0% for deletions and 33% for duplications (100% and 67% are equally valid expected values; for reasons of simplicity we always work with expected VAFs<50% as CopyDetective automatically transforms all VAFs to CFs and performs all calculations on CF-level only).

For the exact approach, all called heterozygous polymorphisms in the control sample are considered. Coverage for both the control and the case sample are given and do not have to be simulated. VAF for the control sample can either be based on the available data or it can be simulated. VAF for the case sample is always simulated based on CF, just like in case of the simulation approach.

To identify CNVs, we apply a weighted t-test. It is investigated whether a significant difference between case and control, evaluating the CFs of an increasing number of simulated polymorphisms, can be observed. By repeating this analysis (default: 500 times; or for all SNPs following the exact approach), we can estimate the lowest number of polymorphisms (*min_SNP*) that have to be evaluated to reach user-defined sensitivity (default: $sens \geq 0.95$).

In the second quality analysis step, we establish a connection between *min_SNP* and window size W (see Figure 2B). Polymorphisms are not evenly distributed across the genome. Instead, some regions show a much higher polymorphism density than others. Additionally, CopyDetective evaluates WES and not WGS data. Thus, to evaluate e.g. 3 polymorphisms, it can be sufficient to analyze a very short region, or it might be necessary to consider a much larger one. We base our analysis on all SNPs detected in the control sample. For all relevant numbers of heterozygous SNPs, i.e. all values of *min_SNP*, we determine the positions and lengths of the corresponding regions. For example, region chr1:906,272-1,007,432 covers 2 heterozygous SNPs. Subsequently, the distribution of the region lengths' is determined and the 95th percentile ($P_{95\%}$) is calculated. If, for example, the 95th percentile for 2 polymorphisms is 2,322,545 bp, we can expect that 95% of all genetic regions of 2,322,545 bp contain at least 2 polymorphisms. The 95th percentile is referred to as window size W within the framework of detection thresholds.

In the third step, the connection between CF and W via *min_SNP* is summed up and the actual detection thresholds are determined (see Figure 2C). CopyDetective allows the user to force CNV calling with the minimum possible CF or the minimum possible window size. However, by default, we are aiming at optimizing both parameters: we normalize window size (W_{norm}) and minimize the distance between CF and W_{norm} (for details see Additional file 1, section 1.2.2, Figure S2). The cell fraction CF and window size W at minimum distance represent the detection thresholds for subsequent CNV calling.

Note that the quality analysis depicted in Figure 2 is performed for both deletions and duplications. Thus, four detection thresholds are determined: CF_{Del}, W_{Del}, CF_{Dup} and W_{Dup}.

CNV calling

Once the thresholds CF_{Del}, W_{Del}, CF_{Dup} and W_{Dup} have been estimated, as described in the 'quality analysis'-step, actual CNV calling is performed. CNV calling with CopyDetective is based on the analysis of VAFs, comparing heterozygous polymorphisms in matching case-control samples. Coverage is considered by determining 99% confidence intervals ($CI_{0.99}$) for the VAFs.

It can be assumed that a heterozygous polymorphism is present at VAF=50% in control samples and – if not affected by CNV – also in case samples. Deviations from this expected frequency may be observed, resulting from low coverage. However, the $CI_{0.99}$ should cover the true VAF of 50% in 99 out of 100 cases. Therefore, we only evaluate polymorphisms that fulfill this criterion.

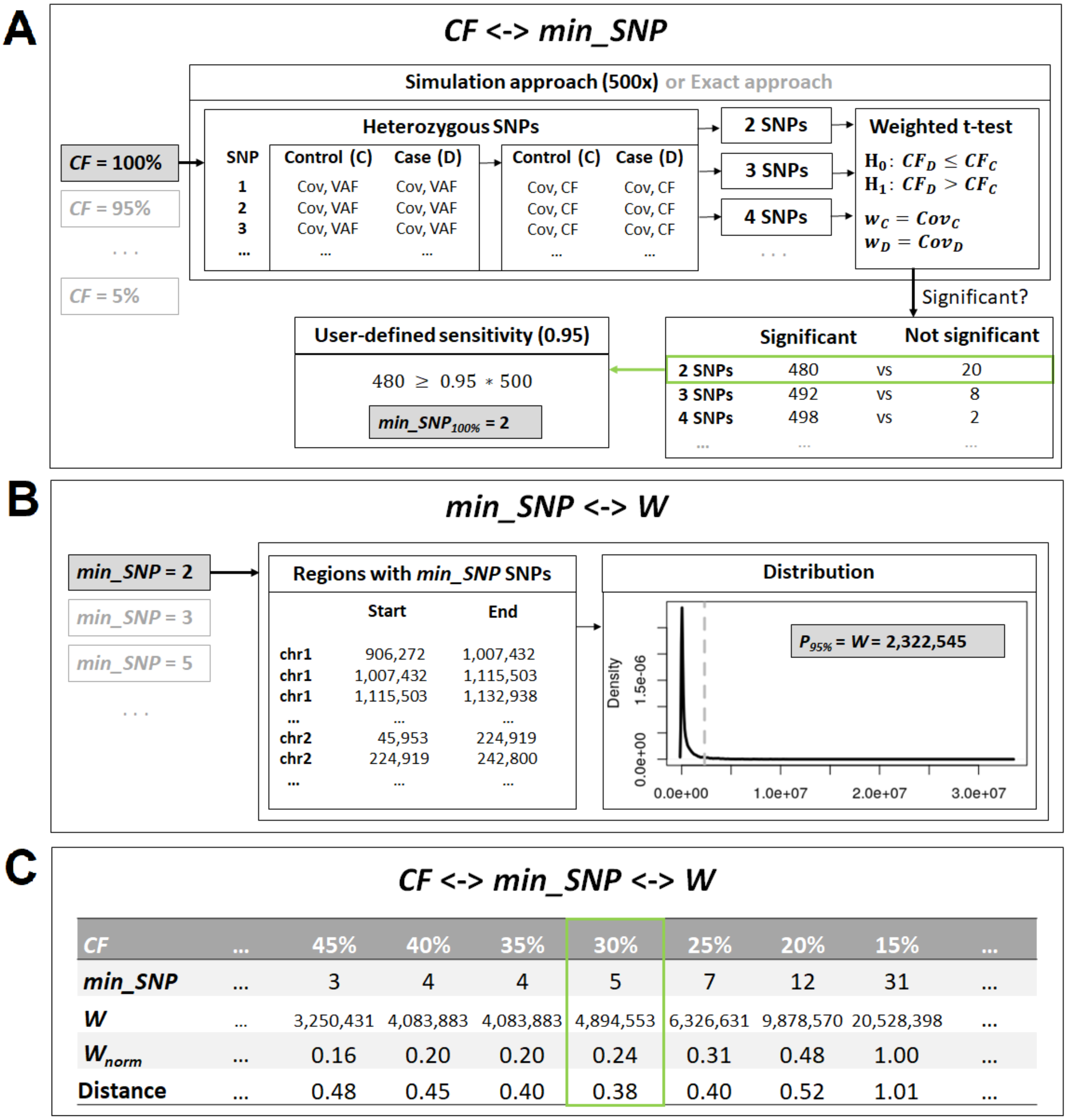

Figure 2. Overview of the quality analysis performed by CopyDetective. A) A connection between *CF* and *min_SNP* is established to reach user-defined sensitivity. Following the simulation approach (default: 500x; alternative: exact approach), heterozygous SNPs are simulated. An increasing number of SNPs is evaluated, applying a weighted t-test. If a significant result is observed in ≥95% of the cases, *min_SNP* has been found. B) For every relevant number of SNPs, the location of regions with *min_SNP* polymorphisms is determined. Based on the locations, the distribution of the region lengths' is determined and $P_{95\%}$, i.e. *W* is calculated. C) A connection between *CF* and *W* has been established. The optimal detection thresholds (by default: optimal trade-off between low CF and small window W) are determined. *CF* – cell fraction; *min_SNP* – minimum number of SNPs; Cov – coverage; VAF – variant allele frequency; $P_{95\%}$ – 95% percentile; *W* – window size; W_{norm} – normalized window size.

In case samples, deviations from the expected VAF of 50% can either – once again – result from low coverage or presence of a CNV. Thus, a polymorphism with VAF=67% can indicate a 1-fold duplication present in 100% of the cells. However, the observed VAF can also be explained by a 1-fold deletion present in 50% of the cells. If the $CI_{0.99}$ of a polymorphism's VAF covers either 33% or 67%, we assume that it can either be explained by a duplication or a deletion. If this is not the case, we only consider deletions. Copy numbers < 1 and > 3 are currently not taken into account.

CopyDetective identifies regions of significant difference comparing one case sample to its matching control. To improve direct interpretability of the results, we decided to work with CFs instead of VAFs. Thus, prior to actual testing, the observed VAFs are transferred to CF-level, considering deletions and duplications. Similarly, the $CI_{0.99}$ are determined for the CFs. Note that the CFs for all heterozygous polymorphisms in the control samples are expected to be zero, but – in reality – show certain variation (for details on the relation between CF and VAF see Additional file 1, section 1.3.1, Figures S3-S4).

The actual test we perform to identify regions of significant difference is a weighted t-test (2-sample, 1-tailed, α adjusted according to Bonferroni correction: $\alpha = 0.05/4 = 0.0125$). For deletions, a sliding window of size W_{Del} with all its covered SNPs is analyzed:

$$\text{Del:} \quad H_0 : CF_{DelD} \leq CF_{DelC} \quad H_1 : CF_{DelD} > CF_{DelC} \tag{1}$$
$$\text{No del:} \quad H_0 : CF_{DelD} \geq CF_{DelC} \quad H_1 : CF_{DelD} < CF_{DelC} \tag{2}$$

CF_{DelD} is defined as the fraction of cells containing a deletion in the case sample ('D' for disease). CF_{DelC} is defined as the fraction of cells containing a deletion in the control sample ('C' for control). If a true deletion is present in the tumor sample, we expect that CF_{DelD} is significantly larger compared to CF_{DelC}.

We expect that $CF_{DelC} = 0$. However, in reality, this is usually not the case. If significantly more cells with a deletion are detected in the control sample compared to the case sample ($CF_{DelD} < CF_{DelC}$), this result indicates that no deletion is likely to be present in the case sample.

Similar to the analysis of deletions, duplications are considered by evaluating a sliding window of size W_{Dup} with all its covered SNPs:

$$\text{Dup:} \quad H_0 : CF_{DupD} \leq CF_{DupC} \quad H_1 : CF_{DupD} > CF_{DupC} \tag{3}$$
$$\text{No dup:} \quad H_0 : CF_{DupD} \geq CF_{DupC} \quad H_1 : CF_{DupD} < CF_{DupC} \tag{4}$$

Instead of an ordinary t-test, we decided to apply a weighted t-test to account for the influence of coverage on the estimated cell fractions: If an evaluated polymorphism is characterized by low coverage – in either one or both, case and control – an observed difference between CF_{DelD} and CF_{DelC} (or between CF_{DupD} and CF_{DupC}) might not result from an actual deletion, but just occur at random. Thus, a decreased weight should be assigned to the low coverage sample(s). In contrast, if an evaluated polymorphism is characterized by high coverage, any observed difference between CF_{DelD} and CF_{DelC} is likely to result from an actual deletion. Thus, an increased weight should be assigned. We define the weights for deletions (case: w_{DelD}; control: w_{DelC}) and duplications (case: w_{DupD}; control: w_{DupC}) as follows:

$$\text{Del:} \quad w_{DelD} = Cov_{DelD} \quad w_{DelC} = Cov_{DelC} \tag{5}$$
$$\text{Dup:} \quad w_{DupD} = Cov_{DupD} \quad w_{DupC} = Cov_{DupC} \tag{6}$$

To determine a list of raw CNV calls for every sample, we exclude non-significant regions as well as significant regions containing no CNV. Furthermore, regions with an estimated CF below the thresholds CF_{Del} and CF_{Dup} (-5% to account for variation of the estimate) are excluded.

It should be noted, that the basis of our approach – the list of polymorphisms – is generated using VarDict [21] (for details on variant calling see Additional file 1, section 1.3.2).

Merging

Raw CNV calls that are reported as being overlapping or located in close vicinity are likely corresponding to one event. Thus, merging of the raw calls is performed. The merging scheme is visualized in Figure 3.

Two CNV calls are merged if the same variant is reported (deletion or duplication), CF is similar ($\lesssim$3 standard deviations – sd) and the calls are overlapping. If two CNV calls are not overlapping, but no significant 'no CNV'-region is located in between and the regions are close (< 20 *Mbp*) they are likewise merged.

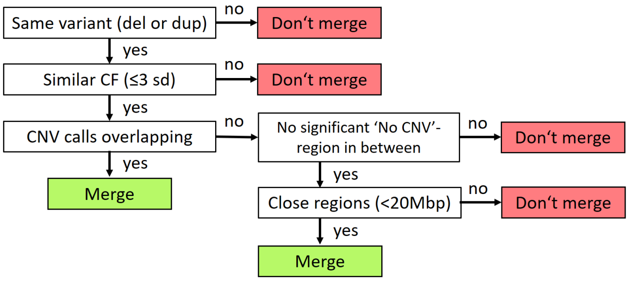

Figure 3. Decision tree for the merging process. Two CNV calls are merged according to the displayed merging scheme. Defining close regions as being separated by < 20 *Mbp* allows for merging of two regions separated by a centromer, which is $\leq$ 7.4 *Mbp*, and for detecting monosomy or trisomy of the smallest chromosomes by only two significant regions.

Note that for estimating the CFs of the merged regions, all significant SNPs are re-evaluated. This can, in some rare cases, lead to a merged region with an overall estimated CF below the actual detection thresholds. However, these regions always contain at least two raw CNV calls with CFs above the detection thresholds.

Filtration (opt.)

Optionally, the merged results can be filtered on the basis of the CNV call quality. Dependent on the analyzed data, it can be useful to consider the merged calls directly. However, we recommend filtration of low quality calls.

Comparison to common approaches

Over the last years, several review articles have been published, considering tools available for CNV calling in NGS data [9, 22, 23]. To evaluate performance of our novel CNV calling algorithm, we compare it to five common CNV calling tools for WES data: ExomeCNV (ExomeCNV, RRID:SCR_010815) [11], VarScan2 (VARSCAN, RRID:SCR_006849) [10], ExomeDepth (ExomeDepth, RRID:SCR_002663) [13], Control-FREEC (Control-FREEC, RRID:SCR_010822) [14] and CNV-seq (CNV-seq, RRID:SCR_013357) [15].

ExomeCNV uses read depth and B-allele frequencies (BAF) from matched WES data to detect deletions, duplications and LOH. It is frequently used for benchmarking [22]. We analyze the CNV calls reported in <Sample>.cnv.txt. The copy number reported in column *copy.number* is evaluated.

VarScan2 analyzes normalized read depth in matched WES samples to detect deletions and duplications. For every region, *num.mark* and *seg.mean* are reported. We exclude all variants with *num.mark* < 10. If *seg.mean* $\geq$ 0.25, the variant is considered a duplication. If *seg.mean* $\leq$ −0.25, the variant is considered a deletion. All variants with −0.25 < *seg.mean* < 0.25 are discarded.

Control-FREEC analyzes copy number- and BAF profiles. Matched control sample are evaluated to distinguish germline variants from somatic ones. Information on subclonal gains and losses is reported and additionally evaluated if biological truth contains information on clonal composition of the samples. In addition to the standard Control-FREEC pipeline, we applied the additional script "assess_significance.R" [24]. CNV calls with a reported p value > 0.05 are excluded. We consider both *WilcoxonRankSumPvalue* (WR) and *KolmogorovSmirnovPvalue* (KS). The copynumber, considering deletions, duplications and LOH, reported in column *copy number* is evaluated.

ExomeDepth applies a beta-binomial model to a set of exons. Normally, the tool requires multiple samples as input. The

idea is that each exome is automatically compared to the exome featuring best correlation. However, as for all samples in our data sets matched controls are available, we assume that the matching control is always the best exome to be used for comparison. The copy number, considering deletions and duplications, reported in column *type* is evaluated.

Additionally, we consider CNV-seq. The tool has not been specifically designed for WES data. However, the general approach is similar to our novel approach CopyDetective: a sliding window is evaluated. The window size is defined by data quality, i.e. in this case coverage. Copy number ratios as well as confidence values are determined. However, different from CopyDetective, CNV calling with CNV-seq is solely based on coverage and not on BAFs. To process the raw output, we exclude all calls with missing values in columns *log2* and/or *cnv.size*. Regions belonging to the same CNV (identifier in column *cnv*) are merged. All merged calls with *cnv.p.value* > 0.05 are excluded. The remaining calls are categorized as deletions if *cnv.log2* < −0.25 and as duplications if *cnv.log2* > 0.25. All the other calls are categorized as LOH.

Details on the precise commands for executing CNV calling with the common approaches are provided in Additional file 1, section 1.4. It should be noted that we tried to apply several additional tools on our data, e.g. THetA2 (THetA, RRID:SCR_001860) [25] or iCNV [26]. Information on all tools we tested, and the reasons why they were excluded from further consideration can be found in Additional file 1, section 1.5.

Results

We apply CopyDetective (simulation approach) on four sets of real data. Performance is compared to five established tools for CNV calling in WES data: ExomeCNV, VarScan2, ExomeDepth, Control-FREEC (WR and KS) and CNV-seq. Two samples from data set 2 (BL_03: P3 and R3) were excluded from analysis. Detailed analyses have shown that almost all validated CNVs appear to have been already present in the control sample – either being contamination or germline calls (for details see Additional file 1, section 2.1, Figure S5). The results for the remaining 98 samples are summed up in Table 2. It should be noted that LOH was excluded from data set 4 as we do not have any information on the frequency of affected cells for these calls. All called CNVs of the type ‘LOH’ were removed from the output of ExomeCNV, ControlFREEC and CNV-seq. CNV calls reported by CopyDetective that were overlapping regions of validated LOH were equally removed. An analysis of data set 4 including LOH can be found in Additional file 1, section 2.2.

A CNV call is considered true positive if it shows any overlap with at least one validated CNV. If a CNV call is overlapping a true variant, but features the “wrong” CNV type (e.g. a deletion is called, while the true CNV is a duplication), is is reported in brackets, as true positive call with false type. We evaluate sensitivity (sens), the positive predictive value (PPV) and the F1 score only considering the true positive calls with correct CNV type (for details on how these statistics are calculated see Additional file 1, section 1.6). For every variant calling tool the number of detectable CNVs is defined. For VarScan and ExomeDepth the number of detectable CNVs is decreased as these two tools are not able to detect LOH. For CopyDetective the number of detectable CNVs is decreased based on every samples’ individual detection thresholds for CNV length and CF (see Additional file 1, section 2.3, Tables S6-S9 for precise detection thresholds). Exemplary variant calling results for one sample are visualized in Additional file 1, section 2.4, Figure S7. A detailed overview of all missed and detected CNVs for each tool is provided in Additional file 1, section 2.4, Tables S10-S13. Detailed variant calling results for CopyDetective are provided in Additional file

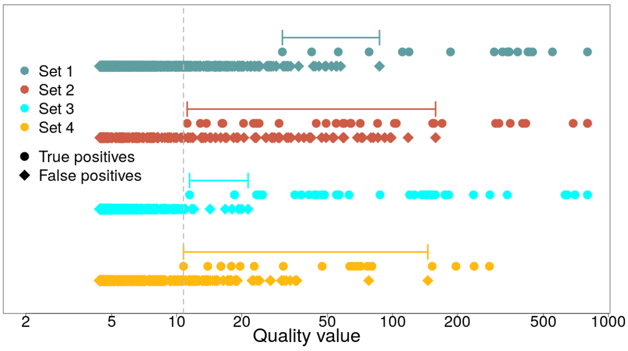

Figure 4. Quality values for true positive and false positive CNV calls reported by CopyDetective (evaluating the true CNV call with the highest quality value in case more than one CNV call overlaps a validated CNV). Ranges indicate the possible values of the quality threshold for each data set. Dashed gray line indicates the default filtration threshold of 10.76, excluding no true positive calls.

2. Results for CopyDetective using the exact approach can be found in Additional file 1, section 2.5, Figure S8 and Table S14.

It can be observed that a majority of common variant calling tools is characterized by low PPV. For ExomeCNV, Control-FREEC (both configurations, WR and KS) and CNV-seq PPV ranges between <0.01 and 0.09 for all data sets. Just in case of VarScan and ExomeDepth – both tools not being able to detect LOH – higher PPVs can partly be observed. However, performance is highly data dependent (set 1: $PPV_{VarScan2} = 0.01$, $PPV_{ExomeDepth} = 0.02$; set 4: $PPV_{VarScan2} = 0.36$, $PPV_{ExomeDepth} = 0.71$). Considering our novel approach CopyDetective without filtration (config ‘raw’), PPV ranges between 0.04 and 0.26. Over all data sets, performance is comparable to the best common approach ExomeDepth (PPV 0.11 vs 0.12). If we apply filtration with our default threshold, values between 0.12 and 0.86 can be observed (over all data sets: 0.33).

Regarding sensitivity, huge differences between all approaches can be observed. While ControlFREEC and CNV-seq are characterized by low sensitivity (maximum: 0.45), much higher values can be observed in case of ExomeCNV (up to 0.89; on average 0.61). However, due to low PPV, the overall performance considering the F1 score is, over all data sets, relatively poor. Similar to PPV, ExomeDepth features highly data dependent performance with respect to sensitivity (ranging between 0.27 and 0.68). In contrast to this, CopyDetective is characterized by stable sensitivity. For both the raw and the filtered results, sensitivity ranges between 0.92 and 1.00. On average, *sens* = 0.96, which slightly exceeds our user-defined sensitivity of 0.95 when determining the detection thresholds.

Table 2 shows that the performance of CopyDetective is, with respect to PPV, data dependent – also including the influence of filtration. Additional file 1, Figure S9 (section 2.6) shows the relation between sensitivity and PPV in the context of an increasing quality threshold. A different development can be observed for the different data sets. Optimization of the F1 score would result in different optimal thresholds for each set (1: 56.05; 2: 16.22; 3: 18.52; 4: 36.36). Thus, the optimal quality threshold over all data sets is difficult to define. However, differences between the data sets are less prominent, when considering the true calls’ quality values (see Figure 4).

For all data sets it can be observed that true CNV calls are characterized by higher quality values compared to false positives. Combining all data sets, no true positive call with a quality value < 10.76 can be observed (see Additional file 1, section 2.6, Table S15). Three out of 4 data sets share a most similar threshold (2: 11.19; 3: 11.48; 4: 10.76). Therefore, we decided to select 10.76 as our default threshold for quality filtration ap-

Table 2. Performance of CopyDetective (raw, i.e. without optional final filtration, and filter, i.e. with default filtration threshold of 10.76) in comparison to five established approaches: ExomeCNV, VarScan2, ExomeDepth, Control-FREEC (WR and KS) and CNV-seq. True positive (TP) calls (in brackets: reporting the number of additional true positive calls if CNV type is not evaluated), false positive (FP) calls, found, missed and detectable CNVs, sensitivity (sens; just evaluating true positive calls with correct CNV type), positive predictive value (PPV; just evaluating true positive calls with correct CNV type) and the F1 score.

Tool	Config		Data set	TP calls (+ false type)	FP calls	CNVs found	CNVs missed	CNVs detectable	Sens	PPV	F1
ExomeCNV			1	1378 (+2204)	215865	49	6	55	0.89	0.01	0.01
			2	280 (+508)	13213	32	15	47	0.68	0.02	0.04
			3	1017 (+1904)	66686	40	8	48	0.83	0.02	0.03
			4	94 (+1)	2064	24	64	88	0.27	0.04	0.08
VarScan2			1	119 (+126)	11736	27	22	49	0.55	0.01	0.02
			2	106 (+185)	2758	26	11	37	0.70	0.04	0.07
			3	65 (+16)	374	21	14	35	0.60	0.15	0.24
			4	30 (+0)	54	23	65	88	0.26	0.36	0.30
ExomeDepth			1	163 (+50)	8074	13	36	49	0.27	0.02	0.04
			2	275 (+162)	2042	25	12	37	0.68	0.12	0.20
			3	175 (+33)	2047	20	15	35	0.57	0.08	0.14
			4	909 (+0)	375	32	56	88	0.36	0.71	0.48
ControlFREEC	WR		1	7 (+6)	1568	5	50	55	0.09	<0.01	0.01
			2	6 (+3)	278	3	44	47	0.06	0.02	0.03
			3	7 (+9)	654	6	42	48	0.13	0.01	0.02
			4	5 (+2)	231	5	83	88	0.06	0.02	0.03
ControlFREEC	KS		1	24 (+38)	7261	12	43	55	0.22	<0.01	0.01
			2	16 (+11)	1124	10	38	48	0.21	0.01	0.03
			3	16 (+11)	1124	10	38	48	0.21	0.01	0.03
			4	32 (+4)	224	17	71	88	0.19	0.09	0.13
CNV-seq			1	25690 (+27757)	1723974	21	34	55	0.38	0.01	0.03
			2	3016 (+1885)	94461	21	26	47	0.45	0.03	0.06
			3	6628 (+4518)	316311	19	29	48	0.40	0.02	0.04
			4	786 (+1125)	28863	15	73	88	0.17	0.03	0.05
CopyDetective	raw		1	33 (+22)	729	18	1	19	0.95	0.04	0.08
			2	63* (+43)	176	34	3	37	0.92	0.26	0.41
			3	67 (+21)	212	40	1	41	0.98	0.24	0.39
			4	23 (+23)	399	19	0	19	1.00	0.05	0.10
CopyDetective	filter	10.76	1	25 (+15)	180	18	1	19	0.95	0.12	0.22
		10.76	2	50 (+31)	63	34	3	37	0.92	0.44	0.60
		10.76	3	60 (+18)	10	40	1	41	0.98	0.86	0.90
		10.76	4	22 (+16)	63	19	0	19	1.00	0.26	0.41

*Sixty-four detected CNVs are overlapping true CNVs. However, as one called CNV is clearly shorter than the validated one and characterized by a remarkably low quality value, we assume that this overlap is just occurring at coincidence. Therefore, it is counted as ‘missed’.

plied in step 4 of our algorithm.

Discussion and Conclusion

CopyDetective is a novel tool for calling somatic CNVs in matched WES data. It has been developed for, but is not limited to the analysis of cancer samples. Different from any other approach, CopyDetective performs initial quality analysis of every sample to estimate the individual detection thresholds, covering the minimum CNV length and the minimum cell fraction. These detection thresholds allow subsequent CNV calling with user-defined sensitivity (default: 0.95).

Considering performance of our new approach, we observe high sensitivity regarding high- as well as low-coverage data. Over all data sets, CopyDetective outperforms all the other tools we considered, even without optional filtration of low-quality calls. Application of the quality filter results in further improvement of performance, especially with respect to PPV. Data indicates that a threshold of 10.76 can be used safely to exclude false positive calls. Detailed additional analyses show that the coordinates of the CNVs, determined by CopyDetective, match the coordinates based on validation experiments (see Additional file 1, section 2.7, Figures S10-S11 and Additional file 2). Furthermore, CFs estimated by CopyDetective match the cell fractions determined by other methods (like fluorescent *in situ* hybridization; see Additional file 1, section 2.8, Figure S12 and Additional file 2). However, it should be noted that the assumed true coordinates and cell fractions may differ from the actual true values. A precise determination of a CNV’s coordinates is usually not possible, but can just be estimated. Furthermore, cell fractions that are based on clonal evolution analysis (sets 1 and 2) may be biased by clustering. A CNV may be present in more or less cells compared to the other mutations in its cluster.

Yet, the fact that CopyDetective is able to estimate CFs is an important characteristic, especially with respect to clonal evolution. While allele frequencies of pathogenic mutations can easily be analyzed to determine subclonal composition of a tumor, this should also be done when analyzing CNVs. However, most tools do just report a copy number variant and its CNV value, but not the fraction of cells affected by the mutation. To

our knowledge, only two additional tools are able to estimate tumor purity in NGS data: CNAnorm [27] and THetA2 [25] (in addition, there are ASCAT [28] and ABSOLUTE [29], however, these tools were designed for SNP array data). While superior performance of THetA2 has been reported by Oesper et al., the tool failed on our data (see Additional file 1, section 1.5).

It may seem astonishing that CNVs reported by CopyDetective match the validated CNVs, spread all over the genome, with respect to coordinates and CFs so well while just analyzing WES data. However, the main advantage of our approach lies in the analysis of a sliding window and the subsequent merging of windows located in close vicinity. This approach allows us to explore between 99.5 and 99.8% of the human genome (see Additional file 1, section 2.3.1, Figure S6).

However, CopyDetective certainly has some limitations. We need a specific scenario – matching control samples – to evaluate changes in VAF for every polymorphism. Performance of CopyDetective is dependent on the accuracy of polymorphism calling in the control sample. However, analyses of robustness have shown that CopyDetective is especially tolerant towards false negative polymorphism calls (see Additional File 1, section 2.9.1, Figures S13-S14 and Tables S16-S17). Our approach is currently just able to call simple deletions or duplications. Dependent on the cells affected by a CNV, ambiguous results are possible as e.g. a deletion present in 50% of the cells can also be explained by a duplication present in 100% of the cells. LOH is always reported as a deletion by CopyDetective. However, a coverage indicator is reported. Analyses show that a true deletion is characterized by a negative coverage indicator (sens=0.88), while LOH is characterized by a coverage indicator overlapping zero (sens=0.87) (see Additional file 1, section 2.10, Figure S15).

Currently, gonosomes are not evaluated by CopyDetective. CNVs on the Y chromosome cannot be detected, as all polymorphisms are hemizygous (same is true for small CNVs just covering homozygous polymorphisms). However, our approach is expected to work for the X chromosomes in case of women.

Dependent on the quality of the provided input data, CopyDetective may not detect and report any small CNVs like focal CNVs, which are known to play an important role in cancer [30]. However, CopyDetective's detection thresholds serve to ensure sufficient sensitivity of the CNV calling results. By reporting the minimum CNV length and the minimum cell fraction, it can easily be decided on whether the analyzed WES data is sufficient to detect the CNVs of interest or if additional experiments have to be performed. When manually changing CopyDetective's automatically determined detection thresholds to higher or lower values, we observe a decline in performance (see Additional file 1, section 2.9.2, Table S18). Higher, stricter thresholds decrease the number of detectable CNVs, no longer tapping the full potential of the data. On the contrary, lower thresholds lead to a major increase in false positive calls.

Concluding, CopyDetective unites an established idea – evaluating the change in VAF of polymorphisms to detect CNVs – with a completely new aspect – determining individual detection thresholds for every sample. Thereby, CopyDetective shines a new light on CNV calling in WES data: Individual detection thresholds reveal that not every data set is equally apt for CNV calling. The general idea of our algorithm – applying a 2-step procedure – is combinable with any other CNV-calling approach. Initial quality analyses, determining individual detection thresholds, can and should be performed prior to actual variant calling.

Availability of source code and requirements

Project name: CopyDetective [12]
Project home page: https://github.com/sandmanns/CopyDetective
Operating system: Platform independent
Programming language: R
Other requirements: none
License: AGPL-3.0
bio.tools ID: biotools:copydetective
SciCrunch ID: RRID:SCR_018909

Availability of supporting data and materials

Data and materials supporting the results of this article are available in Additional file 1 and 2. Sequencing data are available at the NCBI Sequence Read Archive (PRJNA355124, PRJNA561490 and PRJNA285732), the EMBL-EBI European Nucleotide Archive (PRJEB36436), Array Express (E-MTAB-8763) and the Gene Expression Omnibus (GSE68078). All supporting data and materials are available in the *GigaScience* GigaDB database [31].

Additional files

Additional file 1

SupplementaryInformation.pdf: information on supplementary methods and results.

Additional file 2

SupplementaryData1.xlsx: CNV calling output from CopyDetective, including raw and filtered calls for data sets 1 to 4.

Declarations

List of abbreviations

BAF: B-allele frequencies; BL: Burkitt lymphoma; CCF: cancer cell fraction; CF: cell fraction; CNV: copy number variant; FP: false positive; indel: insertion and deletion; KS: KolmogorovSmirnovPvalue; LOH: loss of heterozygosity; MDS: myelodysplastic syndromes; NGS: next-generation sequencing; NMZL: nodal marginal zone lymphoma; PPV: positive predictive value; sd: standard deviation; sens: sensitivity; SNV: single-nucleotide variant; SNP: single-nucleotide polymorphism; SV: structural variant; T-LBL: T-lymphoblastic lymphoma; TP: true positive; VAF: variant allele frequency; WES: whole-exome sequencing; WGS: whole-genome sequencing; WR: WilcoxonRankSumPvalue

Ethical Approval

All patient material was collected and analyzed in accordance with the relevant ethical guidelines and regulations. Informed consent was obtained from all subjects.

Consent for publication

Not applicable

Competing Interests

The authors declare that they have no competing interests.

Funding

This work has been supported by the EU grant Horizon2020 MDS-RIGHT (grant no. 634789), the DFG grant TU 298/5-1 (DFG Clinical Research Unit 326 Male Germ Cells: from Genes to Function), a grant from Deutsche Krebshilfe DKH (grant no. 111347), by Löwenkinder – Verein zur Unterstützung krebskranker Kinder e.V., and by Deutsche Kinderkrebsstiftung (support of the NHL-BFM Registry 2012; DKS349 2014.11 A/B).

Author's Contributions

S.S. developed the algorithm, performed data analyses and wrote the manuscript. S.S. and M.W. performed analysis of validation data. A.O.d.G and J.H.J. collected patient samples and coordinated targeted mutational and whole exome sequencing on the MDS cases. B.B. collected patient samples and coordinated whole exome sequencing on the T-LBL cases. M.D. reviewed development of the algorithm and reviewed the manuscript. All authors read, revised and approved the final version of the manuscript.

Acknowledgements

Not applicable

References

1. Park JY, Kricka LJ, Fortina P. Next-generation sequencing in the clinic. Nat Biotechnol 2013;31:990–992.
2. Release IP. Illumina Introduces the NovaSeq Series – a New Architecture Designed to Usher in the $100 Genome. San Diego (Business Wire) 2017;p. https://www.illumina.com/company/news-center/press-releases/press-release-details.html?newsid=2236383.
3. Ashley EA. Towards precision medicine. Nat Rev Genet 2016;17:507–522.
4. Dey N, Williams C, Leyland-Jones B, De P. Mutation matters in precision medicine: a future to believe in. Cancer Treat Rev 2017;55:136–149.
5. Luthra R, Chen H, Roy-Chowdhuri S, Singh RR. Next-Generation Sequencing in Clinical Molecular Diagnostics of Cancer: Advantages and Challenges. Cancers (Basel) 2015;7:2023–2036.
6. Cheng ML, Solit DB. Opportunities and Challenges in Genomic Sequencing for Precision Cancer Care. Ann Intern Med 2018;168:221–222.
7. Bao R, Huang L, Andrade J, Tan W, Kibbe WA, Jiang H, et al. Review of current methods, applications, and data management for the bioinformatics analysis of whole exome sequencing. Cancer Inform 2014;13:67–82.
8. Sandmann S, de Graaf AO, Karimi M, van der Reijden BA, Hellström-Lindberg E, Jansen JH, et al. Evaluating variant calling tools for non-matched next-generation sequencing data. Sci Rep 2017;7:43169.
9. Zare F, Dow M, Monteleone N, Hosny A, Nabavi S. An evaluation of copy number variation detection tools for cancer using whole exome sequencing data. BMC Bioinformatics 2017;18:286.
10. Koboldt DC, Zhang Q, Larson DE, Shen D, McLellan MD, Lin L, et al. VarScan 2: Somatic mutation and copy number alteration discovery in cancer by exome sequencing. Gen Res 2012;22:568–576.
11. Sathirapongsasuti JF, Lee H, Horst BA, Brunner G, Cochran AJ, Binder S, et al. Exome sequencing-based copy-number variation and loss of heterozygosity detection: ExomeCNV. Bioinformatics 2011;27:2648–2654.
12. Sandmann S. CopyDetective-v1.1.0 2020;DOI: 10.5281/zenodo.3972092.
13. Plagnol V, Curtis J, Epstein M, Mok KY, Stebbings E, Grigoriadou S, et al. A robust model for read count data in exome sequencing experiments and implications for copy number variant calling. Bioinformatics 2012;28:2747–2754.
14. Boeva V, Popova T, Bleakley K, Chiche P, Cappo J, Schleiermacher G, et al. Control-FREEC: a tool for assessing copy number and allelic content using next-generation sequencing data. Bioinformatics 2012;28:423–425.
15. Xie C, Tammi MT. CNV-seq, a new method to detect copy number variation using high-throughput sequencing. BMC Bioinformatics 2009;10:80.
16. da Silva-Coelho P, Kroeze LI, Yoshida K, Koorenhof-Scheele TN, Knops R, van de Locht LT, et al. Clonal evolution in myelodysplastic syndromes. Nat Commun 2017;8:15099.
17. Reutter K, Sandmann S, Rohde J, Mueller S, Wöste M, Khanam T, et al. Reconstructing Clonal Evolution in Relapsed and Non-Relapsed Burkitt Lymphoma. Leukemia 2020;.
18. DNA Copy Number and Loss of Heterozygosity Analysis Algorithms; https://www.illumina.com/documents/products/technotes/technote_cnv_algorithms.pdf.
19. Spina V, Khiabanian H, Messina M, Monti S, Cascione L, Bruscaggin A, et al. The genetics of nodal marginal zone lymphoma. Blood 2016;128:1362–1373.
20. Mayrhofer M, Viklund B, Isaksson A. Rawcopy: Improved copy number analysis with Affymetrix arrays. Sci Rep 2016;6:36158.
21. Lai Z, Markovets A, Ahdesmaki M, Chapman B, Hofmann O, McEwen R, et al. VarDict: A novel and versatile variant caller for next-generation sequencing in cancer research. Nucleic Acids Res 2016;1:e108.
22. Kadalayil L, Rafiq S, Rose-Zerilli MJ, Pengelly RJ, Parker H, Oscier D, et al. Exome sequence read depth methods for identifying copy number changes. Brief Bioinform 2015;16:380–92.
23. Zhao M, Wang Q, Wang Q, Jia P, Zhao Z. Computational tools for copy number variation (CNV) detection using next-generation sequencing data: features and perspective. BMC Bioinformatics 2013;14:S1.
24. Control-FREEC; https://github.com/BoevaLab/FREEC/blob/master/scripts/assess_significance.R.
25. Oesper L, Mahmoody A, Raphael BJ. Quantifying Tumor Heterogeneity in Whole-Genome and Whole-Exome Sequencing Data. Bioinformatics 2014;30:3532–3540.
26. Zhou Z, Wang W, Wang LS, Zhang NR. Integrative DNA copy number detection and genotyping from sequencing and array-based platforms. Bioinformatics 2018;34:2349–2355.
27. Gusnanto A, Wood HM, Pawitan Y, Rabbitts P, Berri S. Correcting for cancer genome size and tumour cell content enables better estimation of copy number alterations from next-generation sequence data. Bioinformatics 2012;28:40–47.
28. Van Loo P, Nordgard SH, Lingjorde OC, Russnes HG, Rye IH, Sun W, et al. Allele-specific copy number analysis of tumors. Proc Natl Acad Sci USA 2010;107:16910–16915.
29. Carter SL, Cibulskis K, Helman E, McKenna A, Shen H, Zack T, et al. Absolute quantification of somatic DNA alterations in human cancer. Nat Biotechnol 2012;30:413–421.
30. Krijgsman O, Carvalho B, Meijer GA, Steenbergen RDM, Ylstra B. Focal chromosomal copy number aberrations in cancer—Needles in a genome haystack. Biochim Biophys Acta

2014;1843:2698–2704.

31. Sandmann S, Wöste M, de Graaf AO, Burkhardt B, Jansen JH, Dugas M. Supporting data for "CopyDetective: Detection Threshold Aware CNV Calling in WES Data. GigaScience Database 2020; http://doi.org/10.5524/100800.

Additional file 1

Click here to access/download
Supplementary Material
SupplementaryInformation.pdf

Click here to access/download
Supplementary Material
latexmkrc

Additional file 2

Click here to access/download
Supplementary Material
SupplementaryData1.xlsx

Personal Cover Click here to access/download;Personal Cover;CoverLetter.pdf

Westfälische Wilhelms-Universität Münster

medizinische fakultät
Westfälische Wilhelms-Universität Münster

Institute of Medizinische InformatikAlbert-Schweitzer-Campus 1 – Building A11 | 48149 Münster

Institute of
Medical Informatics

Albert-Schweitzer-Campus 1
Building A11
48149 Münster

Sarah Sandmann, PhD

Tel. +49 (0) 251 83-5 52 70
Fax +49 (0) 251 83-5 22 59

Sarah.Sandmann@uni-muenster.de
www.imi.uni-muenster.de

Date
17.08.2020

Dear Dr. Goodman,

Please find enclosed the revised version of our manuscript entitled "CopyDetective: Detection Threshold Aware CNV Calling in WES Data" by Sarah Sandmann *et al.*, which we would like to re-submit as a research article (section "technical note") for publication in *GigaScience*. We have thoroughly revised the manuscript according to the reviewers' proposals. In particular, we have introduced the following changes:

- We added an analysis of robustness of the CNV calling results to the supplementary information, including accuracy of polymorphism calling and changing detection thresholds.
- We implemented an additional exact approach for quality analysis, in comparison to the simulation approach, and evaluated its performance. Both the original and the updated version of CopyDetective are available via github (update doi: 10.5281/zenodo.3972092).
- We revised all figures in the main manuscript and supplement.
- We added Additional file 2, containing the CNV calling output from CopyDetective in comparison to biological truth. Additionally, information on the overlap was added.
- We revised the description of our algorithm, especially the quality analysis step.
- We replaced Table 3 by Figure 4.
- We added an analysis on CopyDetective's capability to explore CNVs in the whole genome by analyzing WES data.
- We registered CopyDetective at bio.tools (biotools:copydetective) and SciCrunch (RRID:SCR_018909).

Please find a point-by-point reply to the reviewers' comments in the "Response to Reviewers"-section. We are confident that we have fully addressed the comments from the reviewers as requested. We thank you for giving us the opportunity to improve our work and would be delighted if you could consider our revised manuscript suitable for publication in *GigaScience*.

Please do not hesitate to contact us for any questions.

Yours sincerely,
Sarah Sandmann
