# Supplementary material for: CopyDetective: Detection threshold–aware copy number variant calling in whole-exome sequencing data
Source: Gigascience. 2020 Nov 2;9(11):giaa118. doi: 10.1093/gigascience/giaa118 (PMC7604644; doi:10.1093/gigascience/giaa118)
Supplement: giaa118_Supplemental_Files [file giaa118_supplemental_files.zip › SupplementaryInformation.pdf]

Additional file 1: Supplementary Information

CopyDetective: Detection Threshold Aware CNV Calling in WES Data

Contents

1 Additional methods **3**
1.1 Detailed information on analyzed data sets . . . 3
1.2 CopyDetective – Quality analysis . . . 7
1.2.1 Analyzing coverage distribution . . . 7
1.2.2 Determining the optimum detection thresholds . . . 8
1.3 CopyDetective – CNV calling . . . 9
1.3.1 Deriving the relation between CF and VAF . . . 9
1.3.2 Polymorphism calling . . . 12
1.4 Common algorithms included . . . 13
1.4.1 ExomeCNV . . . 13
1.4.2 VarScan2 . . . 14
1.4.3 ExomeDepth . . . 15
1.4.4 Control-FREEC . . . 16
1.4.5 CNV-seq . . . 17
1.5 Common algorithms excluded . . . 18
1.6 Evaluating performance . . . 19

2 Additional results **20**
2.1 Data set 2 sample 3 . . . 20
2.2 Data set 4 including LOH . . . 21
2.3 Detection thresholds . . . 22
2.3.1 Covering the whole genome with whole-exome data . . . 26
2.4 Detailed variant calling results . . . 27
2.5 CNV calling using the exact approach . . . 33
2.6 Relation between performance and quality filtration . . . 36
2.7 Evaluating coordinates . . . 38
2.8 Evaluating cell fractions . . . 40
2.9 Robustness of the CNV calling results . . . 42
2.9.1 Accuracy of polymorphism calling . . . 42
2.9.2 Changing detection thresholds . . . 45
2.10 Detection of loss of heterozygosity . . . 47

3 Availability of CopyDetective **48**

1 Additional methods

1.1 Detailed information on analyzed data sets

Table S1: Sequencing data characteristics of data set 1: Sample ID used in our study, original sample ID, DNA source (Tumor*: peripheral blood; Tumor**: bone marrow), total reads, mapped reads (absolute and relative), uniquely mapped reads (absolute and relative), Coverage (mean, bases with ≥1x, ≥10x and ≥50x, and called heterozygous SNPs.

Sample	Original ID	DNA source	Total reads	Mapped		Uniquely mapped		Coverage				SNPs
								mean	≥1x	≥10x	≥50x	
MDS_01	UPN01 Normal	Germline	151,107,658	144,312,920	96%	114,237,860	79%	124	99%	98%	81%	11,003
	UPN01 2months	Tumor	238,786,914	228,325,931	96%	151,829,758	66%	164	99%	99%	85%	
	UPN01 19months	Tumor	165,919,542	164,877,976	99%	108,374,805	66%	127	99%	98%	87%	
	UPN01 30months	Tumor	151,120,684	150,174,979	99%	105,255,747	70%	123	99%	98%	86%	
	UPN01 38months	Tumor	159,157,594	158,086,905	99%	116,566,072	74%	134	99%	98%	89%	
	UPN01 50months	Tumor	166,675,288	165,403,973	99%	117,329,347	71%	136	99%	98%	88%	
	UPN01 67months	Tumor	152,100,146	150,962,892	99%	81,687,382	54%	95	99%	97%	77%	
	UPN01 116months	Tumor	114,263,670	101,889,587	89%	84,011,308	82%	104	99%	91%	38%	
	UPN01 119months	Tumor	119,750,354	118,829,426	99%	105,482,041	89%	131	99%	96%	72%	
MDS_02	UPN02 Normal	Germline	117,786,644	113,510,417	96%	95,074,272	84%	105	99%	98%	76%	9,884
	UPN02 baseline	Tumor	113,589,766	112,635,110	99%	98,937,851	88%	121	99%	96%	72%	
	UPN02 8months	Tumor	135,596,638	118,865,906	88%	91,815,866	77%	91	99%	95%	56%	
	UPN02 38months	Tumor	137,659,484	136,620,512	99%	108,807,095	80%	128	99%	98%	86%	
	UPN02 53months	Tumor	177,801,752	176,486,261	99%	112,897,051	64%	134	99%	98%	87%	
	UPN02 60months	Tumor	125,017,414	119,957,162	96%	98,873,261	82%	117	99%	97%	69%	
MDS_03	UPN03 Normal	Germline	120,468,816	114,732,745	95%	90,442,962	79%	100	99%	98%	75%	10,564
	UPN03 baseline	Tumor	141,976,136	134,969,390	95%	121,043,326	90%	140	99%	96%	66%	
	UPN03 93months	Tumor	90,570,998	87,915,039	97%	68,687,210	78%	81	99%	96%	62%	
MDS_04	UPN04 Normal	Germline	110,772,360	107,837,003	97%	90,810,881	84%	109	99%	97%	77%	10,892
	UPN04 baseline	Tumor	110,704,890	108,654,461	98%	88,319,051	81%	101	99%	98%	76%	
	UPN04 72months	Tumor	105,964,368	102,961,349	97%	87,442,574	85%	98	99%	98%	74%	
MDS_05	UPN05 Normal	Germline	160,060,120	156,351,126	98%	126,241,174	81%	168	99%	97%	84%	10,075
	UPN05 baseline	Tumor	121,926,282	112,870,454	93%	89,196,473	79%	95	99%	96%	63%	
	UPN05 79months	Tumor	154,873,436	150,530,007	97%	105,900,454	70%	128	99%	98%	77%	
	UPN05 84months	Tumor*	114,312,534	113,570,863	99%	100,337,164	88%	121	99%	98%	83%	
	UPN05 84months	Tumor**	163,661,150	162,584,793	99%	91,866,982	57%	108	99%	97%	81%	
MDS_06	UPN06 Normal	Germline	124,134,696	106,554,342	86%	75,907,732	71%	73	99%	97%	58%	10,610
	UPN06 baseline	Tumor	218,739,870	208,422,810	95%	186,558,212	90%	217	99%	96%	75%	
	UPN06 63months	Tumor	171,069,434	167,548,427	98%	135,796,133	81%	163	99%	99%	88%	
MDS_07	UPN07 Normal	Germline	155,887,030	146,689,268	94%	113,932,846	78%	116	99%	97%	77%	10,096
	UPN07 baseline	Tumor	129,197,534	120,440,070	93%	104,549,458	87%	113	99%	97%	69%	
	UPN07 4months	Tumor	176,074,140	175,011,495	99%	106,223,597	61%	126	99%	97%	84%	
	UPN07 23months	Tumor	104,475,562	103,598,655	99%	94,756,659	91%	116	99%	95%	67%	
	UPN07 38months	Tumor*	144,050,088	143,140,566	99%	116,049,236	81%	141	99%	99%	88%	
	UPN07 38months	Tumor**	162,356,178	161,323,173	99%	101,674,664	63%	118	99%	97%	83%	
MDS_08	UPN08 Normal	Germline	99,053,768	96,667,953	98%	83,592,443	86%	101	99%	97%	77%	11,132
	UPN08 baseline	Tumor	126,936,024	122,614,285	97%	104,251,488	85%	124	99%	98%	80%	
	UPN08 28months	Tumor	164,358,068	163,373,918	99%	74,035,585	45%	86	99%	97%	72%	
	UPN08 34months	Tumor	103,660,576	103,024,667	99%	74,221,405	72%	86	99%	97%	72%	
	UPN08 45months	Tumor	94,270,644	93,698,872	99%	66,478,312	71%	77	99%	97%	67%	
	UPN08 56months	Tumor	90,756,428	90,197,225	99%	68,677,989	76%	80	99%	97%	70%	
	UPN08 67months	Tumor	121,252,252	120,496,003	99%	110,108,295	91%	130	99%	97%	85%	
	UPN08 112months	Tumor	96,755,164	96,070,284	99%	85,716,485	89%	105	99%	96%	71%	
MDS_9	UPN09 Normal	Germline	161,714,260	151,632,815	94%	114,900,280	76%	137	99%	96%	80%	10,519
	UPN09 baseline	Tumor	109,543,490	108,738,524	99%	89,461,744	82%	107	99%	99%	82%	
	UPN09 8months	Tumor	127,452,440	126,423,990	99%	92,449,043	73%	112	99%	97%	84%	
	UPN09 21months	Tumor	101,212,828	100,671,048	99%	91,473,768	91%	109	99%	97%	80%	
	UPN09 32months	Tumor	103,506,850	102,664,155	99%	78,039,025	76%	95	99%	97%	78%	
	UPN09 53months	Tumor	89,441,872	88,753,449	99%	82,057,129	92%	98	99%	95%	64%	
MDS_10	UPN10 Normal	Germline	145,741,374	140,935,936	97%	113,412,581	80%	143	99%	97%	83%	10,723
	UPN10 3months	Tumor	216,786,456	214,800,216	99%	189,120,172	88%	221	99%	99%	95%	
	UPN10 11months	Tumor	133,372,570	132,302,822	99%	97,981,556	74%	115	99%	98%	85%	
	UPN10 34months	Tumor	105,172,162	104,488,639	99%	92,219,525	88%	111	98%	96%	80%	
	UPN10 49months	Tumor	206,867,442	204,320,613	99%	165,280,580	81%	192	99%	99%	95%	
	UPN10 62months	Tumor*	95,134,686	94,521,159	99%	73,374,730	78%	85	98%	96%	71%	
MDS_11	UPN11 Normal	Germline	198,936,158	182,725,920	92%	97,989,918	54%	86	99%	97%	64%	10,474
	UPN11 baseline	Tumor	112,651,502	109,458,620	97%	85,709,794	78%	101	99%	98%	74%	
	UPN11 29months	Tumor	125,706,708	121,174,220	96%	102,011,796	84%	122	99%	96%	74%	
	Average	Germline	140,514,808	132,904,586	95%	101,503,904	77%	115	99%	97%	76%	10,543
		Tumor	136,004,894	133,073,423	98%	102,190,153	78%	120	99%	97%	77%	

Table S2: Sequencing data characteristics of data set 2: Sample ID used in our study, original sample ID, DNA source, total reads, mapped reads (absolute and relative), uniquely mapped reads (absolute and relative), Coverage (mean, bases with ≥1x, ≥10x and ≥50x, and called heterozygous SNPs.

Sample	Original ID	DNA	Total reads	Mapped		Uniquely mapped		Coverage				SNPs
		source						mean	≥1x	≥10x	≥50x	
BL_01	G1	Germline	33,924,649	33,826,810	100%	32,587,525	96%	51	98%	94%	44%	12,442
	P1	Primary	190,077,305	188,540,410	99%	181,628,464	96%	303	98%	96%	79%	
	R1	Relapse	244,294,627	242,665,199	99%	235,617,268	97%	221	99%	98%	94%	
BL_02	G2	Germline	22,922,803	22,815,965	100%	22,079,977	97%	36	98%	90%	23%	12,912
	P2	Primary	217,765,806	215,924,772	99%	208,381,918	97%	321	98%	98%	96%	
	R2	Relapse	229,448,734	227,292,017	99%	219,074,079	96%	351	98%	98%	95%	
BL_03	G3	Germline	18,877,753	18,828,765	100%	18,314,557	97%	33	97%	84%	22%	11,149
	P3	Primary	135,965,818	135,265,832	99%	131,266,796	97%	232	98%	98%	93%	
	R3	Relapse	152,556,506	151,632,065	99%	146,955,406	97%	263	98%	98%	93%	
BL_04	G4	Germline	34,252,202	34,127,342	100%	33,193,347	97%	61	98%	92%	51%	9,743
	P4	Primary	111,911,873	111,325,704	99%	108,061,669	97%	192	98%	97%	89%	
	R4	Relapse	211,843,837	209,918,389	99%	203,278,075	97%	359	98%	98%	95%	
BL_05	G5	Germline	21,704,291	21,623,884	100%	21,055,811	97%	39	97%	87%	29%	12,079
	P5	Primary	178,135,744	176,750,765	99%	171,540,786	97%	311	98%	96%	87%	
	R5	Relapse	176,011,199	174,469,280	99%	169,175,015	97%	300	98%	98%	94%	
BL_06	G6	Germline	24,751,043	24,658,238	100%	23,818,779	97%	39	98%	91%	28%	12,697
	P6	Primary	204,554,798	203,100,320	99%	196,146,187	97%	310	99%	98%	96%	
BL_07	G7	Germline	20,935,304	20,844,857	100%	20,310,719	97%	37	98%	87%	26%	12,023
	P7	Primary	133,761,957	132,838,692	99%	129,091,841	97%	229	98%	98%	92%	
BL_08	G8	Germline	22,037,001	21,979,126	100%	21,380,791	97%	39	98%	87%	29%	11,928
	P8	Primary	173,541,325	172,472,573	99%	167,206,384	97%	295	99%	98%	94%	
BL_09	G9	Germline	34,187,182	34,057,409	100%	33,178,377	97%	61	98%	93%	52%	11,846
	P9	Primary	155,862,006	154,835,356	99%	150,180,987	97%	271	98%	98%	94%	
BL_10	G10	Germline	26,019,052	25,937,298	100%	25,251,313	97%	46	98%	91%	38%	12,022
	P10	Primary	179,103,411	177,770,296	99%	172,319,617	97%	309	98%	98%	94%	
	Average	Germline	25,961,128	25,869,969	100%	25,117,120	97%	44	98%	90%	34%	11,884
		Primary	168,068,004	166,882,472	99%	161,582,465	97%	277	98%	97%	91%	
		Relapse	202,830,981	201,195,390	99%	194,819,969	97%	299	98%	98%	94%	

Table S3: Sequencing data characteristics of data set 3: Sample ID used in our study, original sample ID, DNA source, total reads, mapped reads (absolute and relative), uniquely mapped reads (absolute and relative), Coverage (mean, bases with $\geq$1x, $\geq$10x and $\geq$50x, and called heterozygous SNPs.

Sample	Original ID	DNA source	Total reads	Mapped		Uniquely mapped		Coverage				SNPs
								mean	$\geq$1x	$\geq$10x	$\geq$50x	
TLBL_01	TG1	Germline	46,502,366	46,225,731	99%	44,428,547	96%	70	98%	93%	51%	12,732
	TP1	Primary	113,564,700	112,011,904	99%	108,107,183	97%	189	98%	95%	79%	
TLBL_02	TG2	Germline	51,235,769	50,952,268	99%	48,942,059	96%	79	98%	94%	57%	10,749
	TP2	Primary	112,660,869	111,077,524	99%	107,121,144	96%	189	98%	96%	80%	
TLBL_03	TG3	Germline	43,402,862	43,134,551	99%	41,310,702	96%	65	98%	92%	47%	11,150
	TP3	Primary	119,433,104	117,884,683	99%	113,705,874	96%	197	98%	96%	83%	
TLBL_04	TG4	Germline	46,839,101	46,594,130	99%	44,679,383	96%	71	98%	93%	51%	10,518
	TP4	Primary	120,048,913	118,378,512	99%	113,972,066	96%	201	98%	96%	82%	
TLBL_05	TG5	Germline	46,670,311	46,420,778	99%	44,698,162	96%	70	98%	94%	52%	10,963
	TP5	Primary	103,161,270	102,245,907	99%	98,907,906	97%	174	98%	95%	74%	
TLBL_06	TG6	Germline	43,799,692	43,564,598	99%	41,718,807	96%	53	98%	91%	39%	11,123
	TP6	Primary	116,376,576	114,676,461	99%	110,517,863	96%	193	98%	96%	82%	
TLBL_07	TG7	Germline	32,270,256	32,091,115	99%	30,840,308	96%	48	98%	93%	37%	11,398
	TP7	Primary	101,468,606	100,011,855	99%	96,207,895	96%	166	98%	96%	78%	
TLBL_08	TG8	Germline	46,125,092	45,878,202	99%	44,122,008	96%	65	98%	93%	49%	11,012
	TP8	Primary	107,998,207	106,507,344	99%	102,558,262	96%	180	98%	96%	80%	
TLBL_09	TG9	Germline	34,481,008	34,256,347	99%	32,716,044	96%	48	89%	71%	32%	7,669
	TP9	Primary	150,088,770	148,331,112	99%	142,644,912	96%	213	98%	98%	93%	
TLBL_10	TG10	Germline	29,591,877	29,416,254	99%	28,197,694	96%	47	90%	72%	32%	8,110
	TP10	Primary	144,964,444	143,258,580	99%	137,864,314	96%	200	98%	98%	93%	
TLBL_11	TG11	Germline	35,931,486	35,658,067	99%	33,829,673	95%	48	89%	71%	32%	7,841
	TP11	Primary	155,044,188	153,136,575	99%	147,272,365	96%	217	99%	98%	94%	
	TR11	Relapse	297,701,295	294,603,215	99%	284,051,829	96%	293	98%	98%	95%	
TLBL_12	TG12	Germline	37,554,934	37,322,330	99%	35,795,124	96%	59	92%	77%	40%	7,856
	TP12	Primary	115,503,832	113,991,750	99%	109,119,429	96%	151	99%	98%	88%	
	TR12	Relapse	283,989,487	282,701,985	100%	273,683,151	97%	277	98%	98%	95%	
TLBL_13	TG13	Germline	37,173,244	36,924,144	99%	35,392,582	96%	59	91%	75%	38%	7,856
	TP13	Primary	146,746,825	144,519,812	98%	138,716,386	96%	191	99%	98%	92%	
	TR13	Relapse	304,473,294	302,859,727	99%	293,757,855	97%	276	99%	98%	95%	
TLBL_14	TG14	Germline	43,080,370	42,823,319	99%	40,775,960	95%	59	92%	77%	40%	7,943
	TP14	Primary	137,628,858	135,654,976	99%	130,078,810	96%	180	99%	98%	90%	
	TR14	Relapse	305,540,841	304,074,713	100%	293,844,463	97%	287	99%	98%	96%	
TLBL_15	TG15	Germline	39,367,277	39,115,982	99%	37,503,082	96%	63	91%	77%	41%	7,968
	TP15	Primary	154,322,864	151,694,045	98%	145,263,182	96%	216	99%	98%	93%	
	TR15	Relapse	306,263,956	304,640,234	99%	294,571,669	97%	317	99%	98%	95%	
	Average	Germline	40,935,043	40,691,854	99%	38,996,676	96%	60	95%	84%	42%	9,659
		Primary	126,600,802	124,892,069	99%	120,137,173	96%	190	98%	97%	85%	
		Relapse	299,593,775	297,775,975	99%	287,981,793	97%	290	99%	98%	95%	

Table S4: Sequencing data characteristics of data set 4: Sample ID used in our study, original sample ID, DNA source, total reads, mapped reads (absolute and relative), uniquely mapped reads (absolute and relative), Coverage (mean, bases with ≥1x, ≥10x and ≥50x, and called heterozygous SNPs.

Sample	Original ID	DNA source	Total reads	Mapped		Uniquely mapped		Coverage				SNPs
								mean	≥1x	≥10x	≥50x	
NMZL_01	1N	Germline	23,902,272	23,164,816	97%	22,712,577	98%	17	99%	71%	3%	8,752
	1T	Tumor	24,025,302	23,865,031	99%	23,392,686	98%	17	99%	72%	2%	
NMZL_02	2N	Germline	19,236,122	18,994,217	99%	18,615,565	98%	14	99%	61%	1%	7,580
	2T	Tumor	18,233,990	18,109,991	99%	17,758,723	98%	13	99%	58%	1%	
NMZL_03	3N	Germline	22,609,478	22,408,794	99%	21,967,300	98%	16	99%	70%	2%	8,482
	3T	Tumor	47,472,837	47,159,134	99%	46,213,896	98%	34	99%	92%	18%	
NMZL_04	4N	Germline	19,641,963	19,519,239	99%	19,151,512	98%	14	99%	64%	1%	7,878
	4T	Tumor	27,241,168	26,970,742	99%	26,467,409	98%	21	99%	79%	5%	
NMZL_05	5N	Germline	26,014,506	25,810,087	99%	25,322,309	98%	19	99%	77%	4%	9,066
	5T	Tumor	18,576,944	18,450,711	99%	18,098,484	98%	13	99%	57%	1%	
NMZL_06	6N	Germline	35,502,144	35,179,275	99%	34,493,282	98%	25	99%	86%	9%	10,268
	6T	Tumor	21,963,205	21,798,698	99%	21,377,774	98%	15	99%	66%	2%	
NMZL_07	7N	Germline	31,247,798	30,745,331	98%	30,141,014	98%	22	99%	82%	6%	9,593
	7T	Tumor	23,235,122	23,036,797	99%	22,574,157	98%	17	99%	70%	2%	
NMZL_08	8N	Germline	22,550,851	22,097,465	98%	21,650,677	98%	16	99%	68%	2%	8,308
	8T	Tumor	26,442,610	26,221,993	99%	25,712,065	98%	19	99%	78%	3%	
NMZL_09	9N	Germline	20,628,781	20,376,449	99%	19,955,592	98%	14	99%	63%	1%	8,054
	9T	Tumor	22,758,132	22,563,215	99%	22,123,669	98%	17	99%	71%	2%	
NMZL_10	10N	Germline	19,779,767	19,609,540	99%	19,232,327	98%	14	99%	62%	1%	7,828
	10T	Tumor	55,805,881	55,328,710	99%	54,222,626	98%	39	99%	95%	25%	
NMZL_11	11N	Germline	19,695,514	19,541,001	99%	19,152,151	98%	14	99%	62%	1%	7,907
	11T	Tumor	27,124,027	26,810,665	99%	26,265,705	98%	19	99%	77%	4%	
NMZL_12	12N	Germline	27,540,276	27,300,028	99%	26,775,632	98%	20	99%	80%	4%	9,532
	12T	Tumor	23,747,770	23,560,829	99%	23,072,394	98%	17	99%	72%	3%	
NMZL_13	13N	Germline	25,372,261	25,109,145	99%	24,579,816	98%	18	99%	74%	3%	9,143
	13T	Tumor	28,687,629	28,466,799	99%	27,908,621	98%	21	99%	81%	5%	
NMZL_14	14N	Germline	25,880,564	25,684,025	99%	25,190,531	98%	18	99%	76%	3%	9,107
	14T	Tumor	21,874,757	21,745,938	99%	21,304,978	98%	15	99%	67%	2%	
NMZL_15	15N	Germline	28,807,692	28,617,705	99%	28,045,066	98%	20	99%	80%	4%	9,707
	15T	Tumor	36,082,161	35,833,751	99%	35,090,589	98%	28	99%	87%	13%	
NMZL_16	16N	Germline	155,924,498	154,487,355	99%	151,537,911	98%	121	100%	99%	86%	11,307
	16T	Tumor	118,643,857	118,482,760	100%	116,407,237	98%	92	100%	98%	75%	
NMZL_17	17N	Germline	153,777,283	153,200,860	100%	150,355,957	98%	116	100%	99%	86%	10,895
	17T	Tumor	121,732,369	121,573,972	100%	119,380,912	98%	95	100%	99%	77%	
NMZL_18	18N	Germline	141,053,529	135,573,325	96%	132,901,324	98%	107	99%	99%	82%	11,255
	18T	Tumor	153,168,364	152,969,171	100%	150,133,316	98%	119	99%	99%	86%	
	Average	Germline	55,699,806	54,809,642	99%	53,751,891	98%	42	99%	79%	23%	9,341
		Tumor	54,585,843	54,374,603	99%	53,350,973	98%	41	99%	81%	24%	

1.2 CopyDetective – Quality analysis

1.2.1 Analyzing coverage distribution

To determine individual detection thresholds for every sample, CopyDetective performs an initial quality analysis. As a basis for this analysis, we use information on the coverage distribution of polymorphisms detected in the actual case- and control samples, assuming a log-normal distribution of coverage. The exemplary coverage distribution for sample MDS_01 germline in comparison to the simulated coverage distribution is visualized in Figure S1.

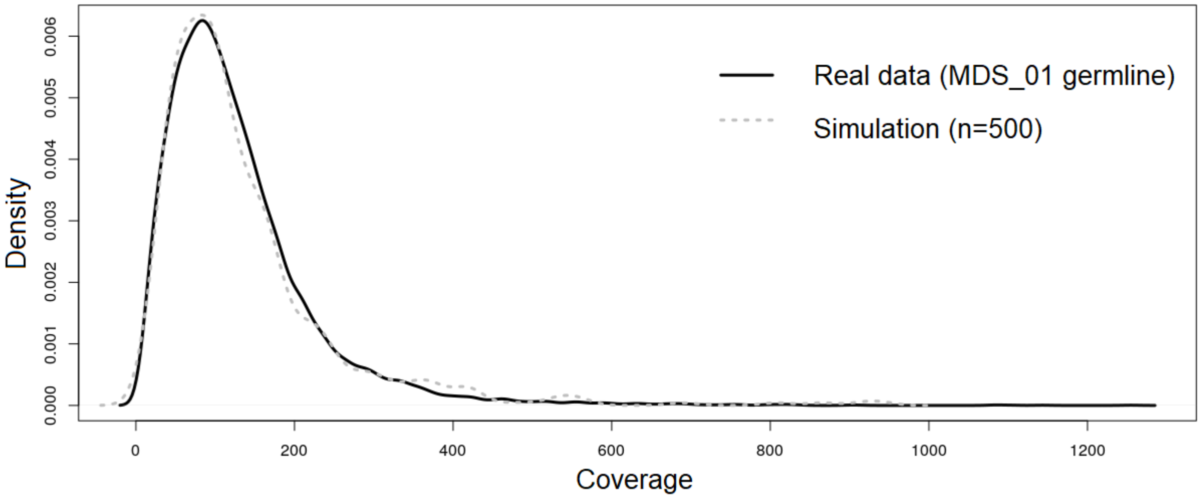

Figure S1: Exemplary coverage distribution for sample MDS_01 germline in comparison to the simulated coverage distribution, assuming a log-normal distribution.

1.2.2 Determining the optimum detection thresholds

To determine the optimum detection thresholds, we minimize the distance between cell fraction and (normalized) window size. In Figure S2, an exemplary relation between window size and cell fraction is displayed (duplications; sample MDS_01 2 months). To detect duplications present in 95% to 100% of the cells with sensitivity≥0.95, a minimum of 3 SNPs has to be evaluated. The average window size to detect 3 SNPs is 3.3 Mbp. The optimum detection thresholds can be observed for a cell fraction of 0.30 and a window size of 7.6 Mbp (evaluation of 9 SNPs).

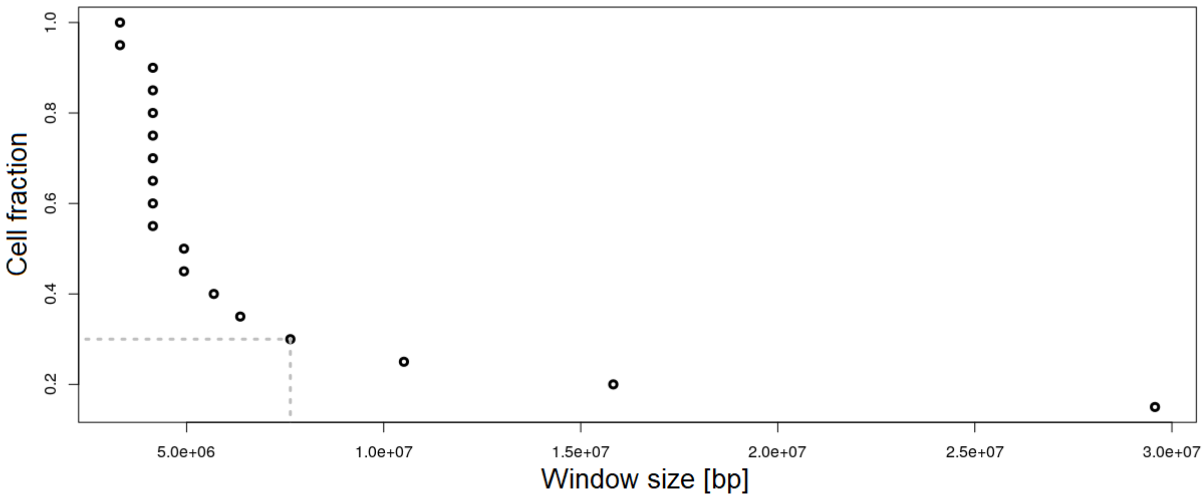

Figure S2: Exemplary relation between window size and cell fraction is displayed (duplications; sample MDS_01 2 months). The optimum detection thresholds can be observed for a cell fraction of 0.30 and a window size of 7,631,549 bp (evaluation of 9 SNPs).

1.3 CopyDetective – CNV calling

1.3.1 Deriving the relation between CF and VAF

CNV calling with CopyDetective is based on the analysis of variant allele frequencies (VAFs) and their relation to the frequency of cells containing a CNV (CF). The algorithm's basic idea is visualized in Figure S3.

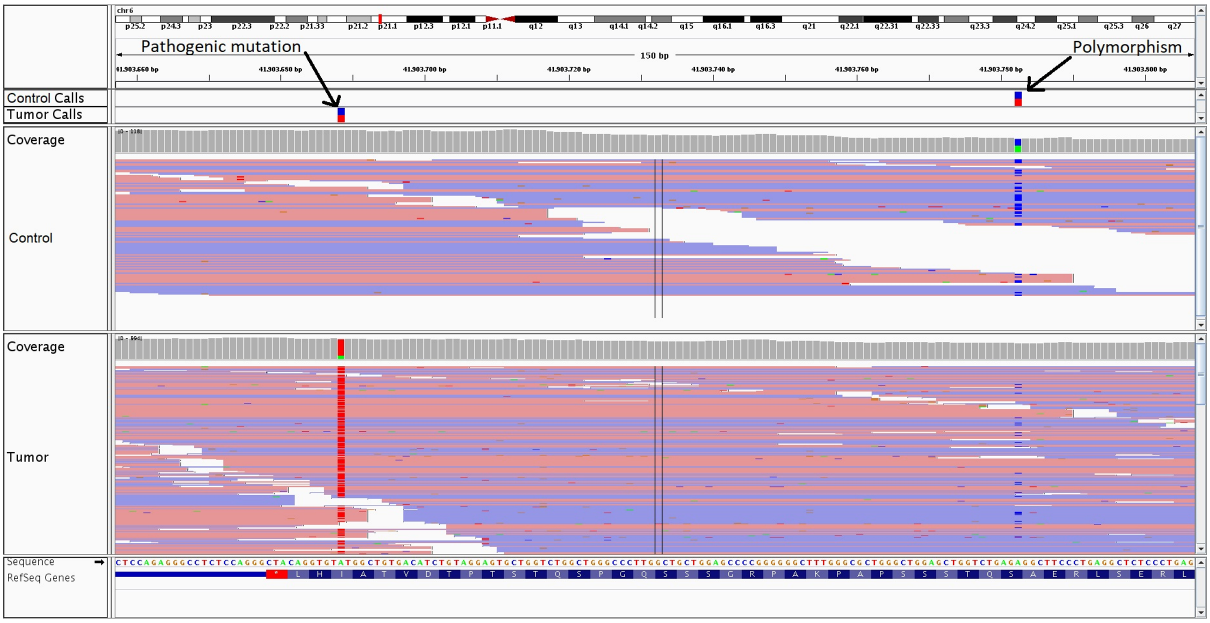

Figure S3: General idea of CopyDetective. A well-known polymorphism (rs1051130) can be observed for both, the control and the case (here: tumor) sample. The VAF of exactly 0.50 in case of the control sample indicates a heterozygous SNP. In the tumor sample, a pathogenic mutation is visible at chr6:41,903,688 with $VAF = 0.81$. The heterozygous SNP shows a decreased frequency of 0.08. This observation indicates a deletion present in a majority of tumor cells.

In both samples, tumor as well as control, a well-known polymorphism (rs1051130; frequency 0.4075 in 1000 Genomes) is visible. The polymorphism's allelic frequency in the control sample (32/64 reads) indicates a heterozygous SNP. In the tumor sample, a pathogenic mutation (COSM144470) is additionally visible at chr6:41,903,688 with $VAF = 0.81$. The heterozygous SNP shows a decreased frequency of 0.08. If we refrain from severe sequencing errors, this observation indicates a deletion present in a majority of tumor cells, affecting the mutated chromosome of our heterozygous polymorphism. Note that – concentrating on deletions and duplications of only one chromosome – a duplication cannot explain the observed changes in allele frequencies as the pathogenic mutation's VAF is > 0.67 (compare Figure S4).

The change in VAF of heterozygous polymorphisms, comparing matching case and control samples, is directly connected to the underlying fraction of cells (CF) featuring either a deletion or a duplication. Calculating this fraction of cells, four cases have to be distinguished:

1) If the VAF of a polymorphism in a case sample is < 0.5, this observation can be explained by a deletion of the mutated allele (DelA*). The fraction of cells featuring a deletion CF_{DelA^*} is calculated with

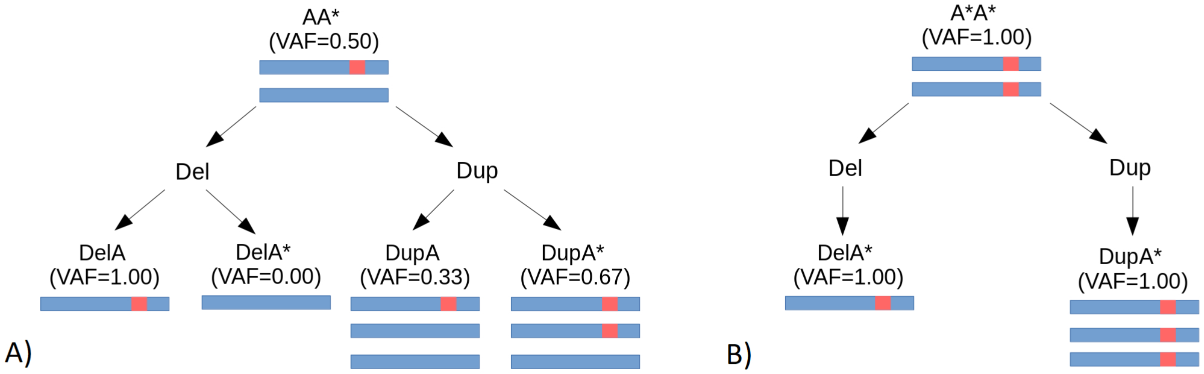

Figure S4: Expected change in VAF of polymorphisms in the presence of CNVs. A) If one allele of a heterozygous polymorphism is affected by a deletion, the frequency of the polymorphism changes to either 1.00 or 0.00 – depending on which allele is deleted. If one allele of a heterozygous polymorphism is affected by a duplication, the frequency changes to either 0.33 or 0.67. B) No change in VAF can be observed if homozygous polymorphisms are affected by deletions or duplications.

$$
\begin{aligned}
VAF &= \frac{ReadsA^*}{ReadsA + ReadsA^*} \\
&= \frac{(\#Cells - \#Cells \cdot CF_{DelA^*})}{\#Cells + (\#Cells - \#Cells \cdot CF_{DelA^*})} \\
&= \frac{1 - CF_{DelA^*}}{1 + 1 - CF_{DelA^*}}
\end{aligned}
$$

$$
CF_{DelA^*} = \frac{2 \cdot VAF - 1}{VAF - 1}. \tag{1}
$$

2) If the VAF of a polymorphism in a case sample is < 0.5, this observation might also be explained by a duplication of the non-mutated allele (DupA). However, as we consider – so far – only onefold duplications, the VAF is not expected to be < 0.33. The fraction of cells featuring a duplication is calculated with

$$
\begin{aligned}
VAF &= \frac{ReadsA^*}{ReadsA + ReadsA^*} \\
&= \frac{\#Cells}{(\#Cells + \#Cells \cdot CF_{DupA}) + \#Cells} \\
&= \frac{1}{1 + CF_{DupA} + 1}
\end{aligned}
$$

$$
CF_{DupA} = \frac{2 \cdot VAF - 1}{-VAF}. \tag{2}
$$

3) If the VAF of a polymorphism in a case sample is > 0.5, this observation can be explained by a deletion of the non-mutated allele (DelA). The fraction of cells featuring a deletion is calculated with

$$
\begin{aligned}
VAF &= \frac{ReadsA^*}{ReadsA + ReadsA^*} \\
&= \frac{\#Cells}{(\#Cells - \#Cells \cdot CF_{DelA}) + \#Cells} \\
&= \frac{1}{1 - CF_{DelA} + 1}
\end{aligned}
$$

$$
CF_{DelA} = \frac{2 \cdot VAF - 1}{VAF}. \tag{3}
$$

4) If the VAF of a polymorphism in a case sample is > 0.5, this observation might also be explainable by a duplication of the mutated allele (DupA*). However, considering only onefold duplciations, the VAF is not expected to be > 0.67. The fraction of cells featuring a duplication is calculated with

$$
\begin{aligned}
VAF &= \frac{ReadsA^*}{ReadsA + ReadsA^*} \\
&= \frac{(\#Cells + \#Cells \cdot CF_{DupA^*})}{\#Cells + (\#Cells + \#Cells \cdot CF_{DupA^*})} \\
&= \frac{1 + CF_{DupA^*}}{1 + 1 + CF_{DupA^*}}
\end{aligned}
$$

$$
CF_{DupA^*} = \frac{2 \cdot VAF - 1}{1 - VAF}. \tag{4}
$$

Transferring the results from 'VAF'-level to 'CF'-level, we calculate the estimated fraction of cells featuring a deletion CF_{DelD} for every polymorphism in the case sample ('D' for disease).

An important aspect to consider, when determining CFs for deletions or duplications, is imprecision in the observed VAFs due to low coverage and general variation in the performed NGS experiments. We assume that higher coverage leads to the estimation of a more precise VAF. The estimated VAF is – on average – closer to the real VAF. To account for the influence of coverage, we determine 95% confidence intervals for all estimated VAFs. We use a binomial distribution to model the VAF and to calculate these intervals: the number of trials n is defined as the coverage at a mutated position, the number of successes k is defined as the number of mutated reads and the probability of success p is defined as the estimated VAF. Subsequently, we also transfer the confidence intervals to 'CF'-level, leading to the lower threshold CI_{DelD_l} and the upper threshold CI_{DelD_u} for cell fractions.

If data allows reasonable explanation of the observed changes in VAF by duplication, i.e. the VAF's confidence interval covers 0.33 or 0.67, for every polymorphism CF_{DupD}, CI_{DupD_l} and CI_{DupD_u} are also calculated.

In the control samples, heterozygous polymorphisms are expected to feature $VAF = 0.50$. Thus, CF_{DelC} and CF_{DupC} would be zero for all polymorphisms. However, deviations from these expected VAFs may occur. Therefore, we also calculate CF_{DelC}, CI_{DelC_l}, CI_{DelC_u}, CF_{DupC}, CI_{DupC_l}, and CI_{DupC_u} for all polymorphisms detected in the control sample.

1.3.2 Polymorphism calling

The approach used to detect the polymorphisms being evaluated by CopyDetective is of minor importance. We decided to use VarDict [1] as this tool performed best when evaluating variant calling tools for non-matched next-generation sequencing data [2] (despite the presence of matched sequencing data, variant calling should solely be performed for the control samples, which equals the analysis of non-matched samples). Subsequently, we decided to filter for synonymous variants present in at least one of of the common polymorphism databases – ESP6500 (http://evs.gs.washington.edu/EVS/), 1000 Genomes [3], dbSNP [4] (build 138 excluding sites after 129), ExAC [5] and ClinVar [6] (common no known medical impact, 03.02.2016) – or non-synonymous or frame-shift variants present in at least two of these databases. All homozygous variants, i.e. the 95% confidence intervals for VAF are not covering 0.5, are excluded.

Any user should consider that sensitive variant calling is important to allow for evaluating a maximum number of variants. Still, the risk of mistaking pathogenic germline mutations or artifacts for polymorphisms should be kept to a minimum.

1.4 Common algorithms included

1.4.1 ExomeCNV

Performing CNV calling with ExomeCNV [7], we stick to the standard workflow. First, GATK is used to determine the DepthOfCoverage:

```
java -jar ./GenomeAnalysisTK.jar -T DepthOfCoverage -omitBaseOutput -omitLocusTable
-R ./Homo_sapiens.GRCh37.67.dna.chromosome.all.fasta -I $dir_bam/$sample.bam
-L $dir/targetRegions/targetRegions.bed -o $dir/exomecnv/$dirsample/$sample.coverage
```

Subsequently, we process the output to prepare it for variant calling with ExomeCNV:

```
input<-read.table(paste(dir,"/",samplename,"/",samplename,".coverage.sample_interval_summary",sep=""),
                  stringsAsFactors=F,header=T)
output<-data.frame(probe=paste("probe",seq(1,length(input[,1])),sep=""),chr=NA,probe_start=NA,
                   probe_end=NA,targeted.base=NA,sequenced.base=NA,coverage=input[,2],
                   average.coverage=input[,3],base.with..15.coverage=input[,9])
temp<-str_split_fixed(input[,1],pattern=":",n=Inf)
output[,2]<-paste("chr",temp[,1],sep="")
temp2<-str_split_fixed(temp[,2],pattern="-",n=Inf)
output[,3]<-as.numeric(temp2[,1])
output[,4]<-as.numeric(temp2[,2])
output[,5]<-output[,6]<-as.numeric(temp2[,2])-as.numeric(temp2[,1])

for(i in c(1:22,"X","Y")){
     write.table(output[output[,2]==paste("chr",i,sep=""),],paste(dirsample,samplename,".chr",i,
                 ".coverage",sep=""),quote=F,row.names=F,sep="\t")
}
```

Finally, CNV calling with ExomeCNV is executed, using the function multi.CNV.analyze():

```
chr.list = c("chr1","chr2","chr3",chr4","chr5","chr6","chr7","chr8","chr9","chr10","chr11","chr12",
             "chr13","chr14","chr15","chr16","chr17","chr18","chr19","chr20","chr21","chr22",
             "chrX","chrY")
temp<-samples[k,1]
temp1<-strsplit(x=temp,split="_")[[1]][1]
temp2<-paste0(temp1,"_normal")
samplename<-temp2
dirsample<-paste(dir,samplename,"/",sep="")
setwd(dirsample)
normal<-read.all.coverage(prefix=paste(samplename,".",sep=""),suffix=".coverage",chr.list=chr.list,
                          header=T)
samplename<-samples[k,1]
dirsample<-paste(dir,samplename,"/",sep="")
system(command = paste0("mkdir -p ",dirsample),intern = F)
setwd(dirsample)
tumor<-read.all.coverage(prefix=paste(samplename,".",sep=""),suffix=".coverage",chr.list=chr.list,
                         header=T)
logR=calculate.logR(normal,tumor)
eCNV=c()
for(i in 1:length(chr.list)){
     idx=(normal$chr==chr.list[i])
     ecnv=classify.eCNV(normal=normal[idx,],tumor=tumor[idx,],logR=logR[idx],min.spec=0.9999,
                        min.sens=0.9999,option="spec",c=0.5,l=70)
     eCNV=rbind(eCNV,ecnv)
}
multicnv<-multi.CNV.analyze(normal=normal,tumor=tumor,logR=logR,all.cnv.ls=list(eCNV),min.spec=0.9999,
                            min.sens=0.9999,option="auc",c=0.5,sdundo=c(2),alpha=c(0.05),
                            coverage.cutoff = 5)
write.output(eCNV,multicnv,samplename)
```

We analyze the CNV calls reported in <Sample>.cnv.txt. The copy number reported in column *copy.number* is evaluated.

1.4.2 VarScan2

Performing CNV calling with VarScan2 [8], we use the docker version of the tool. For every sample, we execute the following command:

```
docker run -v ./data_WES:/data jeltje/varscan2 -c /data/alignment/sampleG.bam
-t /data/alignment/sampleT.bam -q sampleT -i /data/Homo_sapiens.GRCh37.67.dna.chromosome.all.fasta
-b /data/targetRegions/centromeres.bed -w /data/targetRegions/targetRegions.bed
-s /data/varscan2/tempdir > sampleT.cnv
```

For every region, *num.mark* and *seg.mean* are reported in the output file. We exclude all variants with $num.mark < 10$. If $seg.mean \geq 0.25$, the variant is considered a duplication. If $seg.mean \leq -0.25$, the variant is considered a deletion. All variants with $-0.25 < seg.mean < 0.25$ are discarded.

1.4.3 ExomeDepth

CNV calling with the R package 'ExomeDepth' is performed according to the following script:

```
library(ExomeDepth)
data(exons.hg19)

dir<-"/home/"
my.counts<-getBamCounts(bed.file=paste0(dir,"targetRegions/targetRegions_oncocnv.bed"),
                        bam.files=c(paste0(dir,"alignment/tumor.bam"),
                                    paste0(dir,"alignment/germline.bam")),
                        include.chr=F,
                        referenceFasta=paste0(dir,"Homo_sapiens.GRCh37.67.dna.chromosome.all.fasta"))

ExomeCount.dafr<-as(my.counts[,colnames(my.counts)],'data.frame')
ExomeCount.dafr$chromosome<-gsub(as.character(ExomeCount.dafr$space),pattern='chr',replacement='')

my.test<-ExomeCount.dafr$tumor.bam
my.reference.selected<-ExomeCount.dafr$germline.bam
all.exons<-new('ExomeDepth',test=my.test,reference=my.reference.selected,
               formula='cbind(test,reference) ~ 1')
all.exons<-CallCNVs(x=all.exons,chromosome=ExomeCount.dafr$space,start=ExomeCount.dafr$start,
                    end=ExomeCount.dafr$end,name=ExomeCount.dafr$names)
output.file<-"exomeCnv_tumor.csv"
write.csv(file=output.file,x=all.exons@CNV.calls,row.names=F)
```

The copy number, considering deletions and duplications, reported in column *type* is evaluated.

1.4.4 Control-FREEC

CNV calling with Control-FREEC [9] is performed by executing the following command for each sample:

```
freec -conf ./sample/config.txt
```

The config-file is defined as follows:

```
[general]
chrLenFile = /home/controlfreec/hg19.len
window = 0
ploidy = 2
outputDir = /home/controlfreec/
breakPointType=4
chrFiles = /home/reference/
maxThreads=6
breakPointThreshold=1.2
noisyData=TRUE
printNA=FALSE
readCountThreshold=50
BedGraphOutput=TRUE
minimalSubclonePresence = 0.3

[sample]
mateFile = /home/alignment/sampleT.bam
inputFormat = BAM
mateOrientation = FR

[control]
mateFile = /home/alignment/sampleG.bam
inputFormat = BAM
mateOrientation = FR

[BAF]
SNPfile = /home/databases/hg19_snp131.SingleDiNucl.1based.txt
minimalCoveragePerPosition = 5
makePileup=/home/databases/hg19_snp142.SingleDiNucl.1based.txt
fastaFile=/home/Genomes/Homo_sapiens.GRCh37.67/Homo_sapiens.GRCh37.67.dna.chromosome.all.fasta

[target]
captureRegions = /home/targetRegions/targetRegions_Padded.bed
```

Additionally, we apply the script “assess_significance.R” (https://github.com/BoevaLab/FREEC/blob/master/scripts/assess_significance.R) on the output file “sampleT.bam_CNVs” to add two p-values: *WilcoxonRankSumPvalue* (WR) and *KolmogorovSmirnovPvalue* (KS). CNV calls with a reported p value > 0.05 are excluded. The copynumber, considering deletions, duplications and LOH, reported in column *copy number* is evaluated.

1.4.5 CNV-seq

CNV calling with CNV-seq is performed according to the following script:

```
samtools view -F 4 $dir/alignment/$SAMPLENAMET.bam | perl -lane 'print "$F[2]\t$F[3]"'
> $dir/$SAMPLENAMET.hits
samtools view -F 4 $dir/alignment/$SAMPLENAMEG.bam | perl -lane 'print "$F[2]\t$F[3]"'
> $dir/$SAMPLENAMEG.hits
~/R/cnv-seq/cnv-seq.pl --test $dir/$SAMPLENAMET.hits --ref $dir/$SAMPLENAMEG.hits --genome human
```

To process the raw output, we exclude all calls with missing values in columns *log2* and/or *cnv.size*. Regions belonging to the same CNV (identifier in column *cnv*) are merged. All merged calls with $cnv.p.value > 0.05$ are excluded. The remaining calls are categorized as deletions if $cnv.log2 < -0.25$ and as duplications if $cnv.log2 > 0.25$. All the other calls are categorized as LOH.

1.5 Common algorithms excluded

The R package 'saasCNV' [10] performs SNP analysis similar to CopyDetective. VCF files are evaluated, considering coverage and beta allele frequency (BAF) of detected polymorphisms. However, the VCF files we generated based on the VarDict output were not supported by this tool.

The R package 'sequenza' [11] performs an equally comparable SNP analysis. Taking the raw VarScan2 output 'snp' and 'copynumber' as input, analysis is performed. However, these input files, generated on the basis of our data, were not supported.

The R package 'cn.MOPS' [12] (Mixture Of PoissonS for discovering Copy Number variations in next generation sequencing data) models depths of coverage across samples and provides a noise estimate to reduce the false discovery rate. However, when testing the tool on our data, it reports the error: Error in if (all(segMedianT == 0)).

The tool 'CLAMMS' [13] (a scalable algorithm for calling common and rare copy number variants from exome sequencing data) evaluates and normalizes coverage. The tool works without analyzing germline samples. However, when applied on our data it reports to be "not recommended for data from cancer samples", which is why we excluded it from our comparison.

The tool 'iCNV' [14] performs a combined analysis of NGS and SNP array data. We excluded it from our comparison as different SNP arrays were used for the different sets of real data (CytoScan HD Array for data sets 1 and 4, Infinium OmniExpressExome-8v1.3kit for data set 2, InfiniumOmni2-5Exome-8 for data set 3). Furthermore, testing iCNV on data set 2 indicated peculiar results, reporting duplications and deletions for basically every sample and every chromosome [15].

The tool 'THetA2' [16] performs SNP- and coverage analysis, estimating tumor purity and subclonal CNVs similar to our CopyDetective. Applying the 'advanced' mode of THetA2, the tool reports an error (asarray_chkfinite() got an unexpected keyword argument 'dtype'). This error has been reported by several users. However, a solution is not available. Application of THetA2 in the 'easy' mode leads to the warning: This sample isn't a good candidate for THetA analysis. Therefore, we decided to exclude this tool from our comparison.

The approach described by Shen et al. [17], analyzing a designed single nucleotide polymorphism sequencing backbone could not be reproduced as information on the backbone was not available along with the publication and not provided when contacting the corresponding author.

1.6 Evaluating performance

To consider performance of CopyDetective (and other CNV calling tools), we evaluate 3 parameters: sensitivity (sens), positive predictive value (PPV) and F1 score.

Sensitivity is defined as

$$sens = \frac{TP}{TP + FN}$$

with TP being the number of true positives and FN being the number of false negatives. Biological truth is known in case of all data sets by additional experiments. Whenever a reported CNV is overlapping a true CNV, we count this as a true positive call. Not in case of any tool we rate the extent by which the reported and the true CNV are overlapping. A single base pair is – in a most extreme case – enough. If two or more variants are reported to be located within a true CNV, it is just counted as one true positive variant. Similarly, we define a false negative variant as a true CNV for which not a single overlapping CNV is reported.

The positive predictive value is defined as

$$PPV = \frac{TP}{TP + FP}$$

with TP being the number of true positives and FP being the number of false positives. Calculation of PPV is less straightforward compared to sensitivity. Two important aspects have to be considered:

First, some tools, especially ExomeCNV, tend to report a high number of relatively short CNVs. A single copy number variant might be split into more than 100 small variants, overlapping the actual CNV. According to our previous definition, these are true positive calls. However, if we just count them as 1 TP – like in case of sensitivity –, the influence of the false positive calls on PPV would be too high. The positive predictive value would not represent the actual relation between true and false positive calls. Therefore, we decided to count all calls overlapping true CNVs as true positives in case of PPV.

Second, CopyDetective was designed to explain changes in VAF by deletions or duplications present in a certain fraction of cells. However, this approach can lead to ambiguities. If we observe a change in VAF from 0.50 to 0.67, this can either be explained by a duplication affecting 100% of the cells, or by a deletion affecting 50% of the cells. Thus, whenever CopyDetective reports a deletion at the position of a duplication (or the other way round), this is not wrong – it is an alternative explanation. However, it is not a true call either. Therefore, we just report these calls, but do not count them as true or false positives. To treat all approaches equally, we apply the same rules to ExomeCNV, VarScan2, Control-FREEC, ExomeDepth and CNV-seq. As VarScan2 and ExomeDepth are not able to report LOH, these CNVs are excluded from consideration for both tools.

The F1 score allows a combined evaluation of sensitivity and PPV. It is defined as:

$$F1 = 2 \cdot \frac{sens \cdot PPV}{sens + PPV}.$$

2 Additional results

2.1 Data set 2 sample 3

For data set 2, two samples (BL_03: P3 and R3) were excluded. Analyzing the BAF for all polymorphisms detected in the germline sample, we observe that almost all CNVs, validated by SNP array analysis of the tumor samples, appear to be already present in the germline sample – either being contamination or germline calls (see Figure S5).

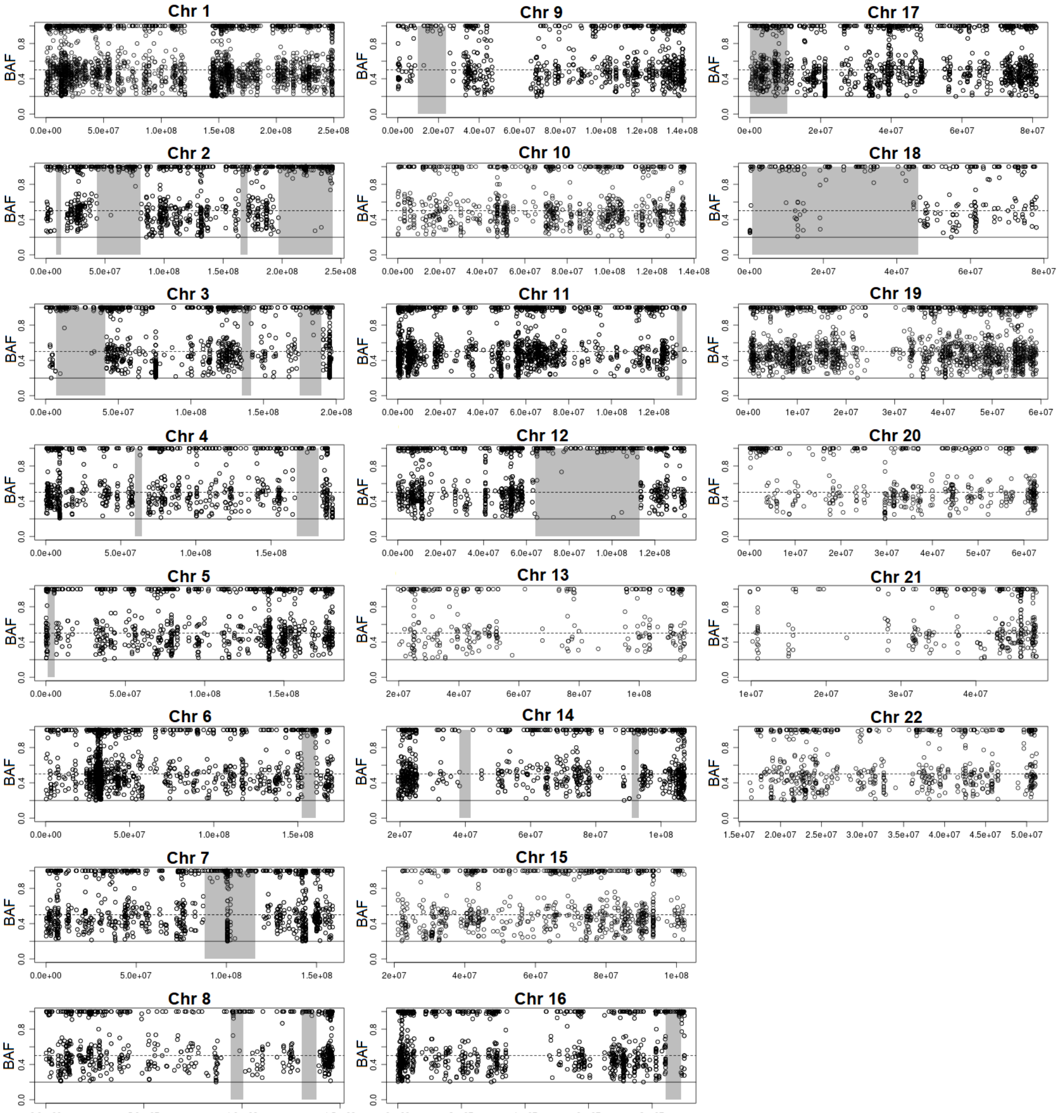

Figure S5: Beta allele frequency for all polymorphisms detected in the germline sample of patient BL_03. Regions of validated CNVs are marked in grey. The black line at 0.2 indicates the applied detection threshold we used for calling polymorphisms.

Polymorphisms detected in the regions marked in grey – except for chromosome 17 – are characterized by VAFs close to 1.0. This observation indicates deletions or LOH being present in a majority of cells. Following CopyDetective's approach for CNV calling, all of these CNVs are categorized as likely homozygous polymorphisms. Thus, no change in their VAF comparing germline and tumor sample would be observable. For this reason, we decided to exclude both tumor samples BL_03 P3 and R3 from analysis.

2.2 Data set 4 including LOH

An overview of the results for data set 4 including LOH can be found in Table S5. As VarScan2 and ExomeDepth do not report any LOH, their results remain unchanged. For CopyDetective, we consider two possible scenarios: worst case (CopyDetective^w) assumes that all validated LOH in data set 4 are present in a fraction of cells above the threshold. Best case (CopyDetective^b) assumes that only validated LOH also detected by CopyDetective are present in a fraction of cells above the threshold.

Table S5: Performance of CopyDetective (raw, i.e. without optional final filtration, and filter, i.e. with default filtration threshold of 10.76) in comparison to five established approaches: ExomeCNV, VarScan2, ExomeDepth, ControlFREEC (WR and KS) and CNV-seq for data set 4 including LOH. Two versions of CopyDetective are considered: worst case CopyDetective^w (all validated LOH are assumed to be present in a fraction of cells above the threshold) and bast case CopyDetective^b (only validated LOH also detected by CopyDetective are assumed to be present in a fraction of cells above the threshold). True positive (TP) calls (in brackets: reporting the number of additional true positive calls if CNV type is not evaluated), false positive (FP) calls, found, missed and detectable CNVs, sensitivity (sens; just evaluating true positive calls with correct CNV type), positive predictive value (PPV; just evaluating true positive calls with correct CNV type) and the F1 score.

Tool	Config		TP calls (+ false type)	FP calls	CNVs found	missed	detectable	Sens	PPV	F1
ExomeCNV			153 (+132)	4748	65	65	130	0.50	0.03	0.06
VarScan2			30 (+0)	54	23	65	88	0.26	0.36	0.30
ExomeDepth			909 (+0)	375	32	56	88	0.36	0.71	0.48
ControlFREEC	WR		13 (+11)	419	12	118	130	0.09	0.03	0.05
ControlFREEC	KS		44 (+37)	877	36	94	130	0.28	0.05	0.08
CNV-seq			786 (+1125)	28863	15	115	130	0.12	0.03	0.04
CopyDetective^w	raw		34 (+33)	399	28	5	33	0.85	0.08	0.10
CopyDetective^w	filter	10.76	30 (+24)	63	26	7	33	0.79	0.31	0.44
CopyDetective^b	raw		34 (+35)	399	28	0	28	1.00	0.08	0.15
CopyDetective^b	filter	10.76	30 (+24)	63	26	2	28	0.93	0.32	0.48

2.3 Detection thresholds

Table S6: Detection thresholds of data set 1: Sample, average coverage (germline and tumor), detection thresholds for deletions (minimum CF, evaluated SNPs and minimum window size [bp]), detection thresholds for duplications (minimum CF, evaluated SNPs and minimum window size [bp]).

Sample	Average coverage		Deletions			Duplications		
	germline	tumor	CF	SNPs	Window	CF	SNPs	Window
MDS_01 2months	124	164	0.30	5	4894553.40	0.30	9	7631549.00
MDS_01 19months	124	127	0.30	5	4894553.40	0.30	9	7755500.40
MDS_01 30months	124	123	0.30	5	4894553.40	0.30	9	7637857.15
MDS_01 38months	124	134	0.30	5	4894553.40	0.30	9	7610767.30
MDS_01 50months	124	136	0.30	5	4894553.40	0.30	9	7703267.70
MDS_01 67months	124	95	0.30	7	4894553.40	0.30	5	7753103.65
MDS_01 116months	124	104	0.25	5	6326631.25	0.45	9	4961936.20
MDS_01 119months	124	131	0.30	5	4894553.40	0.30	9	7780195.55
MDS_02 baseline	105	121	0.30	5	5269054.65	0.30	10	9161183.00
MDS_02 8months	105	91	0.30	5	5269054.65	0.30	8	9404764.50
MDS_02 38months	105	128	0.30	5	5269054.65	0.30	9	7462287.30
MDS_02 53months	105	134	0.30	5	5269054.65	0.30	10	8502070.75
MDS_02 60months	105	117	0.30	5	5269054.65	0.30	10	8941277.90
MDS_03 baseline	100	140	0.30	5	5200219.40	0.30	9	8154680.50
MDS_03 93months	100	81	0.30	5	5200219.40	0.30	9	8161024.25
MDS_04 baseline	109	101	0.30	5	4908478.00	0.30	9	7641325.45
MDS_04 72months	109	98	0.30	5	4908478.00	0.30	8	7046942.15
MDS_05 baseline	168	95	0.30	5	5320874.00	0.30	8	8185008.50
MDS_05 79months	168	128	0.30	5	5320874.00	0.30	9	8193674.40
MDS_05 84months pb	168	121	0.30	5	5320874.00	0.30	9	7505897.25
MDS_05 84months bm	168	108	0.30	5	5320874.00	0.30	9	8204572.00
MDS_06 baseline	73	217	0.25	7	6576212.70	0.30	9	8143222.45
MDS_06 63months	73	163	0.30	5	5129247.10	0.30	9	8124897.05
MDS_07 baseline	116	113	0.30	5	5129210.60	0.30	11	8008222.20
MDS_07 4months	116	126	0.30	5	5129210.60	0.30	9	8829932.20
MDS_07 23months	116	116	0.30	5	5129210.60	0.30	10	8795816.35
MDS_07 38months pb	116	141	0.30	5	5129210.60	0.25	9	9383857.75
MDS_07 38months bm	116	118	0.30	5	5129210.60	0.30	10	8069875.35
MDS_08 baseline	101	124	0.30	5	4981033.35	0.30	9	7752242.05
MDS_08 28months	101	86	0.30	5	4981033.35	0.30	8	7876243.40
MDS_08 34months	101	86	0.30	5	4981033.35	0.30	11	7159303.40
MDS_08 45months	101	77	0.30	5	4981033.35	0.25	9	8987750.90
MDS_08 56months	101	80	0.30	5	4981033.35	0.30	9	7736930.00
MDS_08 67months	101	130	0.30	5	4981033.35	0.30	9	7797372.90
MDS_08 112months	101	105	0.30	5	4981033.35	0.30	9	7765821.00
MDS_09 baseline	137	107	0.25	5	5877445.35	0.30	8	8038192.10
MDS_09 8months	137	112	0.30	5	5124254.55	0.30	9	7291153.50
MDS_09 21months	137	109	0.30	7	5124254.55	0.30	8	8089556.15
MDS_09 32months	137	95	0.25	6	6496792.05	0.30	9	7217267.70
MDS_09 53months	137	98	0.25	7	6496792.05	0.30	9	8078612.45
MDS_10 3months	143	221	0.30	5	5058431.80	0.30	9	7082043.60
MDS_10 11months	143	115	0.30	5	5058431.80	0.30	8	7105228.10
MDS_10 34months	143	111	0.25	7	6397286.10	0.30	10	8458226.40
MDS_10 49months	143	192	0.30	5	5058431.80	0.30	8	7683826.80
MDS_10 62months pb	143	85	0.30	5	5058431.80	0.30	9	7757929.60
MDS_11 baseline	86	101	0.30	5	5077095.00	0.30	9	7881176.50
MDS_11 29months	86	122	0.30	5	5077095.00	0.30	9	7881909.20

Table S7: Detection thresholds of data set 2: Sample, average coverage (germline and tumor), detection thresholds for deletions (minimum CF, evaluated SNPs and minimum window size [bp]), detection thresholds for duplications (minimum CF, evaluated SNPs and minimum window size [bp]).

Sample	Average coverage		Deletions			Duplications		
	germline	tumor	CF	SNPs	Window	CF	SNPs	Window
BL_01 P	51	303	0.30	5	4441485.50	0.25	13	9741577.00
BL_01 R	51	221	0.30	5	4441485.50	0.30	9	8029017.60
BL_02 P	36	321	0.30	5	4500493.00	0.30	9	7144925.50
BL_02 R	36	351	0.30	5	4500493.00	0.30	9	7359096.50
BL_04 P	61	192	0.30	5	5550728.90	0.30	9	9111684.20
BL_04 R	61	359	0.30	5	5550728.90	0.30	8	8381098.80
BL_05 P	39	311	0.30	5	4819648.30	0.30	9	8461853.00
BL_05 R	39	300	0.30	5	4819648.30	0.30	8	7506343.00
BL_06 P	39	310	0.30	5	4343355.75	0.35	6	5136348.00
BL_07 P	37	229	0.30	5	4774395.30	0.30	9	7701854.45
BL_08 P	39	295	0.30	5	4604614.80	0.30	8	6903130.20
BL_09 P	61	271	0.30	5	4756111.70	0.30	9	7435104.80
BL_10 P	46	309	0.30	5	4767261.00	0.30	8	6971859.00

Table S8: Detection thresholds of data set 3: Sample, average coverage (germline and tumor), detection thresholds for deletions (minimum CF, evaluated SNPs and minimum window size [bp]), detection thresholds for duplications (minimum CF, evaluated SNPs and minimum window size [bp]).

Sample	Average coverage		Deletions			Duplications		
	germline	tumor	CF	SNPs	Window	CF	SNPs	Window
TLBL_01 P	70	189	0.30	5	4372493.80	0.30	10	7595773.40
TLBL_02 P	79	189	0.30	5	5035637.20	0.30	9	7755013.00
TLBL_03 P	65	197	0.25	6	5592340.50	0.30	8	7048942.60
TLBL_04 P	71	201	0.30	5	5152054.30	0.30	9	8249386.50
TLBL_05 P	70	174	0.30	5	4982990.00	0.30	10	8725074.05
TLBL_06 P	53	193	0.30	5	5009762.40	0.30	9	8031356.35
TLBL_07 P	48	166	0.30	5	4948463.90	0.30	8	6882114.90
TLBL_08 P	65	180	0.30	5	5044338.90	0.30	9	8362502.00
TLBL_09 P	48	213	0.30	5	7301203.00	0.30	9	11777935.85
TLBL_10 P	47	200	0.30	5	6745618.40	0.30	9	11363220.90
TLBL_11 P	48	217	0.30	5	6849612.75	0.30	9	11048620.85
TLBL_11 R	48	293	0.30	5	6849612.75	0.30	9	11223258.50
TLBL_12 P	59	151	0.25	6	7952835.45	0.30	8	10085069.60
TLBL_12 R	59	277	0.30	5	6772942.85	0.30	9	10964954.80
TLBL_13 P	59	191	0.25	7	8823648.70	0.30	9	10839524.95
TLBL_13 R	59	276	0.30	5	6703395.00	0.30	9	10923909.00
TLBL_14 P	59	180	0.30	5	6623185.40	0.30	9	10887271.80
TLBL_14 R	59	287	0.30	5	6623185.40	0.30	8	9828812.60
TLBL_15 P	63	216	0.30	5	6677651.50	0.30	9	11017907.50
TLBL_15 R	63	317	0.30	5	6677651.50	0.30	9	10982930.70

Table S9: Detection thresholds of data set 4: Sample, average coverage (germline and tumor), detection thresholds for deletions (minimum CF, evaluated SNPs and minimum window size [bp]), detection thresholds for duplications (minimum CF, evaluated SNPs and minimum window size [bp]).

Sample	Average coverage		Deletions			Duplications		
	germline	tumor	CF	SNPs	Window	CF	SNPs	Window
NMZL_01	17	17	0.30	6	6705785.80	0.30	11	11162530.20
NMZL_02	14	13	0.30	7	8573862.00	0.30	13	14141666.85
NMZL_03	16	34	0.30	5	6361801.45	0.25	14	14302486.00
NMZL_04	14	21	0.30	6	7324167.20	0.30	12	12890562.00
NMZL_05	19	13	0.30	7	7464393.20	0.30	12	11714900.60
NMZL_06	25	15	0.30	6	6016057.80	0.30	11	10024902.00
NMZL_07	22	17	0.30	6	6405823.70	0.30	11	10497818.40
NMZL_08	16	19	0.30	6	7174156.10	0.30	11	11737975.45
NMZL_09	14	17	0.30	6	7389632.30	0.30	11	11836621.40
NMZL_10	14	39	0.30	6	7553855.75	0.30	10	11228623.70
NMZL_11	14	19	0.30	6	7370676.70	0.30	11	11764925.30
NMZL_12	20	17	0.30	6	6554950.50	0.30	12	11293482.30
NMZL_13	18	21	0.30	5	5725099.60	0.30	10	9781729.40
NMZL_14	18	15	0.30	6	6375473.90	0.30	13	12067558.25
NMZL_15	20	28	0.25	8	7742308.00	0.30	10	9327749.60
NMZL_16	121	92	0.30	5	4801739.80	0.30	8	6961228.00
NMZL_17	116	95	0.30	5	4978832.80	0.30	9	7840657.65
NMZL_18	107	119	0.30	5	4809261.95	0.30	8	7069992.20

2.3.1 Covering the whole genome with whole-exome data

Despite analyzing whole-exome sequencing data, CopyDetective reports CNVs spanning the whole genome. We evaluate polymorphisms located in a target region of ∼63Gbp (excluding gonosomes), which is roughly 2.2% of the whole genome. Figure S6 visualizes the relation between the distance between two polymorphisms and the corresponding percentile for sample BL_02 (best case as this sample has the highest number of polymorphisms over all data sets) and NMZL_02 (worst case as this sample has the lowest number of polymorphisms over all data sets).

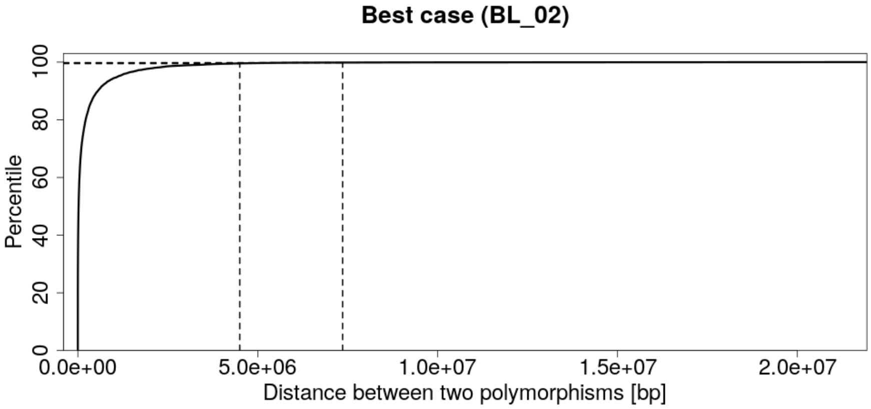

A)

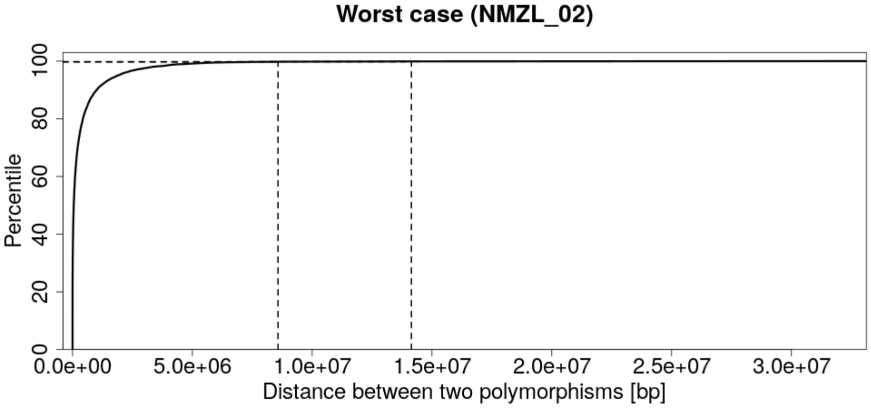

B)

Figure S6: Relation between the distance between two polymorphisms and the corresponding percentile. The percentile indicates the percentage of covered bases in the whole genome. A) Best case sample BL_02. B) Worst case sample NMZL_02. Dashed lines mark the automatically determined thresholds (window) for deletions (lower value) and duplications (higher value).

To detect CNVs, CopyDetective analyzes a sliding window. This window covers in best case sample BL_02 >99.5% (for deletions; >99.7% for duplications) of the genome. For worst case sample NMZL_02 fewer polymorphisms can be evaluated. Therefore, the detection thresholds are generally higher. In this case, >99.7% (for deletions; >99.8% for duplications) of the genome are covered by analyzing a sliding window. Thus, CopyDetective cannot examine any deletions located in 0.3-0.5% of the genome and duplications located in 0.2-0.3% of the genome – despite being characterized by values above the detection thresholds.

2.4 Detailed variant calling results

For every sample, we have a detailed look at variant calling results for every tool. As an example, the results for sample MDS_06 63months are visualized in Figure S7.

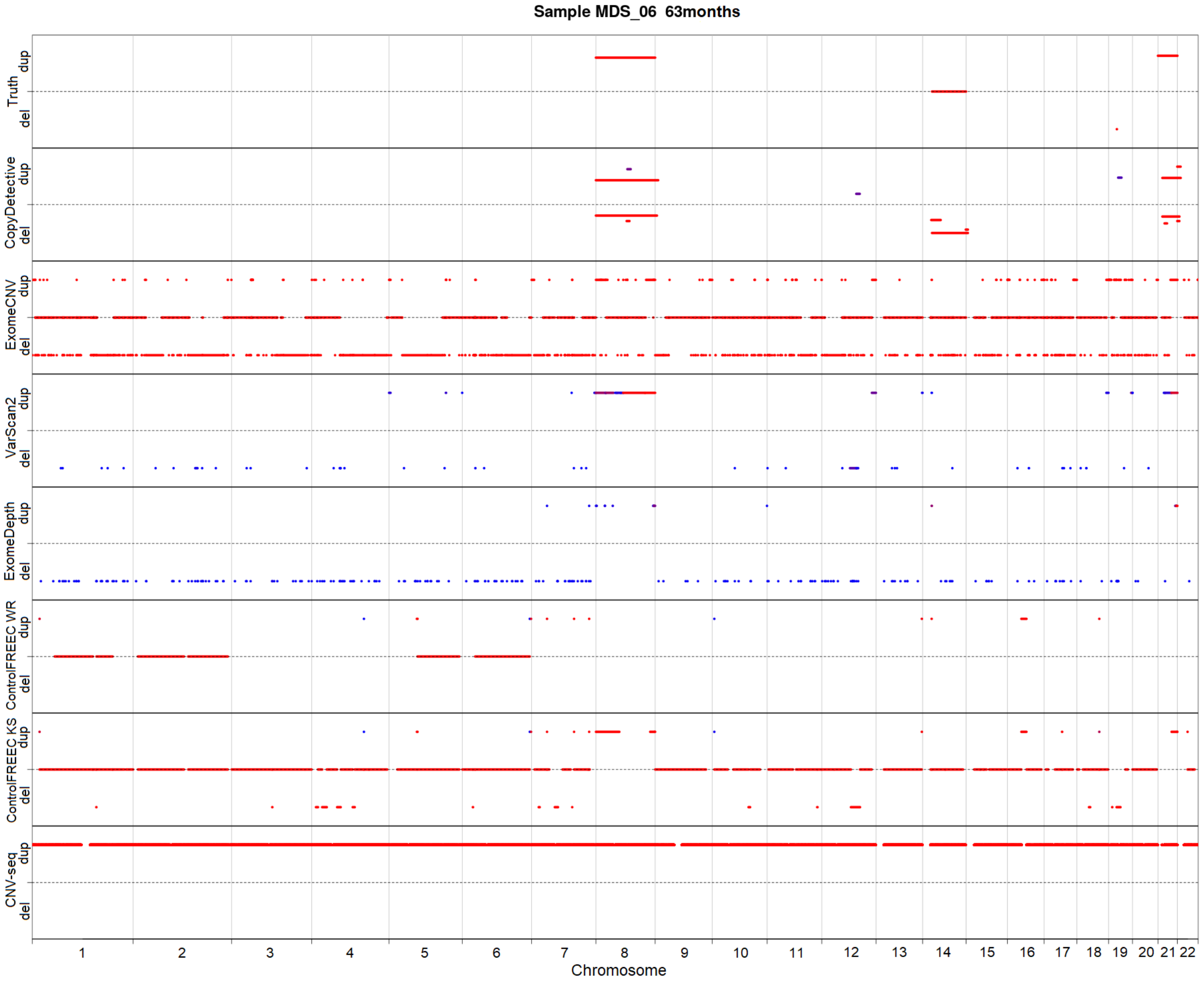

Figure S7: Exemplary variant calling output for sample MDS_06 63months: Truth, CopyDetective (merged results, no optional filtration applied), ExomeCNV, VarScan2, ExomeDepth, ControlFREEC WR (Wilcoxon Rank Sum), ControlFREEC KS (Kolmogorov Smirnov) and CNVSeq. Duplications are plotted above the dashed line, deletions below, LOH on the line. For Truth and CopyDetective, distance from the dashed line indicates the CF for deletions and duplications. Color indicates quality of the called variant (red: high; blue: low).

It can be observed that variant calling results based on CopyDetective match the true CNV calls quite well. Just the small deletion on chromosome 19 is missed. However, this observation is expected as size of the CNV is below the detection threshold (CNV: 122,170 bp; threshold: 5,129,247 bp). The coordinates of the called CNVs as well as the predicted CFs are close to the true values.

LOH is called as deletion by CopyDetective (for details see section 2.10). It can be observed that this is also the case for the LOH on chromosome 14. Coverage indicator for this call is +0.01 [0.07;-0.05], which indicates a true LOH.

For ExomeCNV, ContorlFREEC WR and CNV-seq, data does not indicate presence of true CNVs on chromosome 8, 14 and 21. For VarScan2, the duplications of chromosomes 8 and 21 are detected with high quality. However, as VarScan2 does not detect any LOH, the CNV on

chromosome 14 cannot be called. For ExomeDepth, we observe a high number of false positive deletions. For ControlFREEC KS, we observe a high number of false positive LOH. However, for both tools a minor indicator for true CNVs present on chromosomes 8 and 21 can be observed.

In the following Tables S10-S13, detailed variant calling results for all true CNVs and all variant calling tools we considered are summed up. Variant calling output for CopyDetective – including raw and filtered results – can be found in Additional file 2.

Table S10: Detailed CNV calling results for data set 1. Results for common tools (1: ExomeCNV; 2: VarScan2; 3: ExomeDepth; 4: ControlFREEC WR; 5: ControlFREEC KS; 6: CNV-seq; T: detected; F: missed; NA: not detectable) in comparison to CopyDetective (no (R): region size below detection threshold; no (F): frequency below detection threshold; quality (maximum for split calls); $\widehat{CF}$: CF estimated by CopyDetective, intervals for split calls).

					CNV		Common tools						CopyDetective			
Sample	chr	start	end	type	value	CF	1	2	3	4	5	6	Called	>thresholds	quality	$\widehat{CF}$
MDS_01 2m	5	95804838	154408872	del	1.30	0.70	T	T	T	F	F	F	T	yes	344.53	0.54
MDS_01 19m	5	95804838	154408872	del	1.20	0.80	T	T	T	F	F	F	T	yes	430.49	0.63
MDS_01 30m	5	95804838	154408872	del	1.95	0.05	T	F	F	F	F	F	F	no (F)		
MDS_01 50m	5	95804838	154408872	del	1.95	0.05	T	F	F	F	F	F	F	no (F)		
MDS_01 67m	5	95804838	154408872	del	1.82	0.18	T	F	F	F	F	F	F	no (F)		
MDS_01 116m	5	95804838	154408872	del	1.43	0.57	T	T	F	F	F	F	T	yes	185.60	0.42
MDS_01 119m	5	95804838	154408872	del	1.10	0.90	T	T	F	F	F	F	T	yes	445.25	0.69
MDS_02 b	4	85662390	190921709	LOH	2.00	0.75	T	NA	NA	F	F	F	T	yes	295.93	0.54
MDS_02 8m	4	85662390	190921709	LOH	2.00	0.45	T	NA	NA	F	F	F	T	yes	119.27	0.35
MDS_02 38m	4	85662390	190921709	LOH	2.00	0.78	F	NA	NA	F	F	F	T	yes	347.31	0.68
MDS_02 53m	1	0	121499999	dup	2.10	0.10	T	F	F	F	F	T	F	no (F)		
MDS_02 53m	1	125000000	249250621	dup	2.10	0.10	T	T	F	T	T	T	T	no (F)	4.54	0.25
MDS_02 53m	4	85662390	190921709	LOH	2.00	0.46	F	NA	NA	F	F	F	T	yes	77.89	0.25
MDS_02 60m	1	0	121499999	dup	2.12	0.12	T	T	T	F	F	T	F	no (F)		
MDS_02 60m	1	125000000	249250621	dup	2.12	0.12	T	T	T	T	T	T	T	no (F)	4.45	0.28
MDS_02 60m	4	85662390	190921709	LOH	2.00	0.90	T	NA	NA	F	F	F	T	yes	379.97	0.76
MDS_03 b	13	101700000	110300000	del	1.86	0.14	T	F	F	F	F	F	F	no (F)		
MDS_04 b	8	39195829	39430768	del	1.00	1.00	T	F	F	F	F	F	F	no (R)		
MDS_04 72m	8	39195829	39430768	del	1.00	1.00	F	F	F	F	F	F	F	no (R)		
MDS_05 b	8	0	146364022	dup	2.20	0.20	T	T	T	T	T	T	T	no (F)	10.88	0.25
MDS_05 b	12	11867287	12027012	del	1.10	0.90	T	F	F	F	F	F	F	no (R)		
MDS_05 79m	12	11867287	12027012	del	1.10	0.90	T	F	F	F	F	F	F	no (R)		
MDS_05 84m pb	11	118338477	118354345	dup	2.60	0.60	T	T	F	F	F	T	F	no (R)		
MDS_05 84m pb	12	11867287	12027012	del	1.10	0.90	T	T	F	F	F	F	F	no (R)		
MDS_05 84m bm	11	118338477	118354345	dup	2.60	0.60	T	T	F	F	F	T	F	no (R)		
MDS_05 84m bm	12	11867287	12027012	del	1.10	0.90	T	T	F	F	F	F	F	no (R)		
MDS_06 b	19	20598535	20720705	del	1.00	1.00	T	F	F	F	T	F	F	no (R)		
MDS_06 b	21	0	48129895	dup	2.50	0.50	T	T	F	F	T	T	T	yes	42.20	0.29
MDS_06 63m	8	0	146364022	dup	2.90	0.90	T	T	T	F	T	T	T	yes	339.97	0.65
MDS_06 63m	14	23431738	107285437	LOH	2.00	0.95	T	NA	NA	F	F	F	T	yes	Inf	0.76
MDS_06 63m	19	20598535	20720705	del	1.00	1.00	T	F	F	F	T	F	F	no (R)		
MDS_06 63m	21	0	48129895	dup	2.95	0.95	T	T	T	F	T	T	T	yes	111.10	0.71
MDS_07 b	8	0	146364022	dup	2.10	0.10	T	T	T	F	T	T	T	no (F)	4.71	0.26
MDS_07 4m	8	0	146364022	dup	2.08	0.08	T	T	F	T	T	T	T	no (F)	12.68	0.25
MDS_07 38m pb	8	0	146364022	dup	2.30	0.30	T	T	T	T	T	T	T	yes	30.89	0.20-0.28
MDS_07 38m bm	8	0	146364022	dup	2.40	0.40	T	T	F	F	F	T	T	yes	56.05	0.29-0.36
MDS_08 b	5	82169433	161991376	del	1.10	0.90	T	T	T	F	F	F	T	yes	423.81	0.53
MDS_08 b	12	11208694	11256831	del	1.00	1.00	F	F	F	F	F	F	F	no (R)		
MDS_08 28m	5	82169433	161991376	del	1.20	0.80	T	T	T	F	F	F	T	yes	420.14	0.59
MDS_08 28m	12	11208694	11256831	del	1.00	1.00	T	F	F	F	F	F	F	no (R)		
MDS_08 34m	5	82169433	161991376	del	1.98	0.02	T	F	T	F	F	F	F	no (F)		
MDS_08 34m	12	11208694	11256831	del	1.00	1.00	T	F	F	F	F	F	F	no (R)		
MDS_09 b	5	86890558	162823229	del	1.40	0.60	T	T	F	F	F	F	T	yes	323.01	0.49
MDS_09 b	9	91800000	117700000	del	1.60	0.40	T	F	F	F	F	F	F	yes		
MDS_09 b	18	70720102	72639955	dup	1.00	1.00	T	F	F	F	F	T	F	no (R)		
MDS_09 8m	5	86890558	162823229	del	1.96	0.04	T	F	F	F	F	F	F	no (F)		
MDS_09 8m	18	70720102	72639955	dup	1.00	1.00	T	T	F	F	F	T	F	no (R)		
MDS_09 21m	18	70720102	72639955	dup	1.00	1.00	T	F	F	F	F	T	F	no (R)		
MDS_09 32m	18	70720102	72639955	dup	1.00	1.00	T	F	F	F	F	T	T	no (R)	4.45	0.30
MDS_09 53m	5	86890558	162823229	del	1.97	0.04	T	T	F	F	F	F	T	no (F)	9.13	0.22
MDS_09 53m	18	70720102	72639955	dup	1.00	1.00	T	F	F	F	T	T	F	no (R)		
MDS_10 3m	5	98131505	156829473	del	1.90	0.10	T	T	F	F	F	F	T	no (F)	20.09	0.26
MDS_10 3m	13	32377128	53622642	del	1.90	0.10	T	T	F	F	F	F	F	no (F)		
MDS_11 b	21	39593444	39950442	dup	2.90	0.90	F	F	F	F	F	T	F	no (R)		
MDS_11 29m	21	39593444	39950442	dup	3.00	1.00	F	T	F	F	F	T	F	no (R)		

Table S11: Detailed CNV calling results for data set 2. Results for common tools (1: ExomeCNV; 2: VarScan2; 3: ExomeDepth; 4: ControlFREEC Wilkoxon Rank Sum; 5: ControlFREEC Kolmogorov Smirnov; 6: CNV-seq; T: detected; F: missed; NA: not detectable) in comparison to CopyDetective (no (R): region size below detection threshold; no (F): frequency below detection threshold; quality (maximum for split calls); $\widehat{CF}$: CF estimated by CopyDetective, intervals for split calls).

					CNV		Common tools						CopyDetective			
Sample	chr	start	end	type	value	CF	1	2	3	4	5	6	Called	>thresholds	quality	$\widehat{CF}$
BL_01 P	17	38924	15970682	dup	3.00	1.00	T	T	F	F	F	T	T	yes	20.41	0.65
BL_01 R	1	17270614	117544702	dup	2.72	0.72	T	T	T	F	F	T	T	yes	16.37	0.51-1.07
BL_01 R	1	179748514	244340598	dup	2.72	0.72	T	T	T	T	T	T	T	yes	54.81	0.96
BL_01 R	13	97337247	108507574	del	1.40	0.60	T	T	T	F	F	F	T	yes	59.80	0.48
BL_01 R	17	38924	15970682	dup	3.00	1.00	F	T	F	F	F	T	T	yes	30.88	1.06
BL_02 P	1	87297849	109268615	dup	2.32	0.32	F	F	T	F	F	T	T	yes	22.69	0.72
BL_02 P	1	150250636	154919080	dup	2.32	0.32	F	T	T	F	T	T	T	no (R)	145.01	0.44
BL_02 P	3	8774129	19341221	LOH	2.00	0.64	F	NA	NA	F	F	F	T	yes	414.07	0.71
BL_02 P	3	35554832	66703685	LOH	2.00	0.64	F	NA	NA	F	F	F	T	yes	414.07	0.71
BL_02 P	3	107599953	130947273	del	1.36	0.64	F	F	F	F	F	F	F	yes		
BL_02 P	4	53751933	155356517	del	1.36	0.64	T	T	T	F	F	F	T	yes	63.78	0.26-0.61
BL_02 P	9	214606	2719094	del	1.36	0.64	T	T	T	F	T	F	T	no (R)	22.74	0.45
BL_02 P	16	10995907	11001754	del	1.36	0.64	F	T	T	F	F	F	F	no (R)		
BL_02 P	16	50322149	55146566	del	1.36	0.64	F	T	T	F	T	F	T	yes	16.22	0.44
BL_02 R	3	8774129	19341221	LOH	2.00	0.74	T	NA	NA	F	F	F	T	yes	400.23	0.60
BL_02 R	3	35554832	66703685	LOH	2.00	0.74	T	NA	NA	F	F	F	T	yes	400.23	0.60
BL_02 R	3	107599953	130947273	del	1.26	0.74	T	F	T	F	T	F	T	yes	169.59	0.56
BL_02 R	4	53751933	155356517	del	1.26	0.74	T	T	T	F	F	F	T	yes	58.97	0.27-0.56
BL_02 R	9	214606	2719094	del	1.26	0.74	T	T	T	F	T	F	T	no (R)	36.53	0.56
BL_02 R	16	10995907	11001754	del	1.26	0.74	F	T	F	F	F	F	F	no (R)		
BL_02 R	16	50322149	55146566	del	1.26	0.74	T	T	T	F	T	F	T	yes	23.37	0.47
BL_04 R	4	24651750	32186096	dup	2.60	0.60	F	F	F	F	F	T	T	no (R)	6.62	1.19
BL_04 R	7	1	159138663	dup	2.38	0.38	T	F	F	F	F	T	T	yes	101.99	0.35
BL_04 R	18	1	78077248	dup	2.46	0.46	F	F	F	F	F	T	T	yes	49.26	0.47
BL_05 P	1	149860372	249218992	dup	2.54	0.54	T	T	T	T	F	T	T	yes	29.82	0.40-1.05
BL_05 P	6	108666	53044379	LOH	2.00	1.00	T	NA	NA	T	T	F	T	yes	Inf	0.67
BL_05 P	7	97611196	159119486	dup	2.38	0.38	T	T	T	F	F	T	T	yes	85.28	0.33
BL_05 P	11	106120529	134693504	dup	2.68	0.68	T	T	F	F	T	T	T	yes	12.88	0.54
BL_05 P	13	87132137	109878779	dup	2.54	0.54	F	T	F	F	F	T	F	yes		
BL_05 P	17	8547	17739348	LOH	2.00	1.00	T	NA	NA	F	F	F	T	yes	300.18	0.66
BL_05 R	1	149860372	249218992	LOH	2.00	0.86	T	NA	NA	F	T	F	T	yes	686.47	0.70
BL_05 R	4	71566	44660248	del	1.24	0.76	T	T	T	F	F	F	T	yes	350.92	0.73
BL_05 R	4	148522294	189592930	del	1.24	0.76	T	T	T	F	T	F	T	yes	153.46	0.68
BL_05 R	6	108666	53044379	LOH	2.00	1.00	T	NA	NA	F	T	F	T	yes	Inf	0.74
BL_05 R	9	473379	119979614	del	1.54	0.46	T	T	T	F	F	F	T	yes	155.72	0.41-0.93
BL_05 R	11	106120529	134693504	dup	2.86	0.86	T	T	T	F	T	T	T	yes	24.10	0.53
BL_05 R	13	87132137	109878779	dup	2.86	0.86	T	T	T	F	F	T	T	yes	11.19	0.48
BL_05 R	15	69220912	102461162	dup	2.34	0.34	T	F	T	F	F	T	T	yes	104.07	0.58
BL_05 R	17	8547	17739348	LOH	2.00	1.00	T	NA	NA	F	F	F	T	yes	311.26	0.73
BL_06 P	14	100795140	107349540	del	1.42	0.58	T	T	T	F	T	F	T	yes	13.74	0.25
BL_06 P	17	39646022	81195210	dup	2.24	0.24	F	F	F	F	F	T	T	no (F)	58.36	0.23
BL_07 P	17	59560464	81195210	dup	2.38	0.38	F	F	F	F	F	T	T	yes	46.06	0.29
BL_07 P	22	19710874	25550451	del	1.24	0.76	T	T	T	F	T	F	F	yes		
BL_09 P	1	103343637	107921487	LOH	2.00	0.68	T	NA	NA	F	F	F	F	no (R)		
BL_09 P	1	118583409	181375684	dup	2.22	0.22	T	T	T	F	F	T	T	no (F)	44.59	0.22
BL_10 P	1	144364074	223348765	dup	2.26	0.26	T	F	T	F	F	T	T	no (F)	87.42	0.26
BL_10 P	6	131063724	170980171	dup	2.58	0.58	F	F	F	F	F	T	T	yes	70.85	0.79

Table S12: Detailed CNV calling results for data set 3. Results for common tools (1: ExomeCNV; 2: VarScan2; 3: ExomeDepth; 4: ControlFREEC Wilkoxon Rank Sum; 5: ControlFREEC Kolmogorov Smirnov; 6: CNV-seq; T: detected; F: missed; NA: not detectable) in comparison to CopyDetective (no (R): region size below detection threshold; quality (maximum for split calls); $\widehat{CF}$: CF estimated by CopyDetective, intervals for split calls).

				CNV			Common tools						CopyDetective			
Sample	chr	start	end	type	value	CF	1	2	3	4	5	6	Called	>thresholds	quality	$\widehat{CF}$
TLBL_01 P	8	161532	26250762	LOH	2.00	NA	T	NA	NA	F	F	F	T	yes	213.29	0.72
TLBL_01 P	8	26251468	43789824	dup	3.00	NA	T	T	T	F	F	T	T	yes	11.48	0.67
TLBL_01 P	9	78775	39140153	del	1.00	NA	T	T	T	F	F	F	T	yes	237.13	0.76
TLBL_01 P	9	71219289	107324247	del	0.00	NA	T	T	T	F	F	F	T	yes	175.14	0.47
TLBL_01 P	9	73513811	108817987	dup	3.00	NA	F	F	F	F	F	T	T	yes	62.86	0.26
TLBL_04 P	9	107153	31588249	LOH	2.00	NA	T	NA	NA	T	T	F	T	yes	151.49	0.69
TLBL_05 P	8	29794135	31270546	LOH	2.00	NA	T	NA	NA	F	F	F	F	no (R)		
TLBL_05 P	8	76716547	80600507	LOH	2.00	NA	T	NA	NA	F	F	F	F	no (R)		
TLBL_05 P	9	21056582	32397920	del	1.00	NA	T	T	T	F	T	F	T	yes	24.12	0.62
TLBL_05 P	17	26900862	81052262	LOH	2.00	NA	T	NA	NA	F	T	F	T	yes	Inf	0.76
TLBL_06 P	1	103551918	120521760	del	1.00	NA	T	T	T	F	T	F	T	yes	136.48	0.78
TLBL_06 P	9	21041753	39146898	del	1.00	NA	T	T	T	T	T	F	T	yes	7.71	0.28
TLBL_07 P	1	23175042	25145491	del	1.00	NA	F	F	F	F	T	F	T	no (R)	6.45	0.25
TLBL_07 P	9	7847716	37566978	del	1.00	NA	T	T	T	F	F	F	T	yes	23.46	0.41
TLBL_08 P	7	42899	159124578	dup	3.00	NA	T	T	T	F	F	T	T	yes	339.73	0.70
TLBL_09 P	9	107153	38000660	LOH	2.00	NA	T	NA	NA	F	F	F	T	yes	150.87	0.81
TLBL_10 P	1	83929243	87667937	del	1.00	NA	F	F	F	F	F	F	T	no (R)	8.58	0.45
TLBL_10 P	4	88388799	190450528	dup	3.00	NA	F	F	F	F	F	T	T	yes	40.99	0.75
TLBL_10 P	4	114865091	190023992	del	0.00	NA	T	F	F	F	F	F	T	yes	126.40	0.44
TLBL_10 P	9	18817594	39151736	del	1.00	NA	T	T	T	F	F	F	T	yes	87.51	0.72
TLBL_11 P	22	28984447	31390187	del	1.00	NA	T	T	T	F	F	F	T	no (R)	12.14	0.26
TLBL_11 R	1	208419966	243354174	del	1.00	NA	T	F	T	F	F	F	T	yes	158.02	0.73
TLBL_12 P	9	107153	37591236	LOH	2.00	NA	T	NA	NA	T	T	F	T	yes	54.97	0.66
TLBL_12 R	1	206635152	247586336	del	1.00	NA	T	F	T	F	F	F	T	yes	145.41	0.41
TLBL_12 R	1	208930710	247615669	dup	3.00	NA	F	F	F	F	F	T	T	yes	48.40	0.86
TLBL_12 R	6	66560775	170916485	dup	3.00	NA	T	T	T	F	F	T	T	yes	37.78	0.30
TLBL_12 R	9	107153	21019817	LOH	2.00	NA	T	NA	NA	F	F	F	T	yes	44.28	0.57
TLBL_13 P	7	42899	37885121	dup	3.00	NA	T	T	T	F	F	T	T	yes	46.77	0.49
TLBL_13 P	8	155931	146294106	dup	3.00	NA	T	T	T	F	F	T	T	yes	144.79	0.55
TLBL_13 P	9	107153	41972020	LOH	2.00	NA	T	NA	NA	F	F	F	T	yes	181.99	0.69
TLBL_13 R	8	155931	146294106	dup	3.00	NA	T	T	T	F	F	T	T	yes	151.80	0.56
TLBL_13 R	9	107153	24071176	LOH	2.00	NA	T	NA	NA	T	T	F	T	yes	119.94	0.74
TLBL_13 R	11	127511508	134943190	del	1.00	NA	T	F	T	F	F	F	T	yes	11.53	0.65
TLBL_13 R	17	42753802	81052262	dup	3.00	NA	T	F	T	T	T	T	T	yes	139.92	0.53
TLBL_14 P	7	142496773	159124578	dup	3.00	NA	T	T	F	F	F	T	T	yes	25.01	0.32
TLBL_14 P	9	675257	138881297	LOH	2.00	NA	T	NA	NA	F	F	F	T	yes	646.95	0.81
TLBL_14 P	16	84761	67503379	dup	3.00	NA	T	T	T	F	F	T	T	yes	56.44	0.48
TLBL_14 P	16	84513562	84513588	del	1.00	NA	F	F	F	F	F	F	F	no (R)		
TLBL_14 P	20	63231	62934877	dup	3.00	NA	T	T	F	F	F	T	T	yes	174.97	0.73
TLBL_14 R	7	142496622	159124578	dup	3.00	NA	T	T	F	F	F	T	T	yes	35.28	0.47
TLBL_14 R	9	107153	139400317	LOH	2.00	NA	T	NA	NA	F	F	F	T	yes	629.75	0.81
TLBL_14 R	16	84761	67503379	dup	3.00	NA	T	T	T	F	F	T	T	yes	56.63	0.47
TLBL_14 R	20	63231	62934877	dup	3.00	NA	T	T	F	F	F	T	T	yes	140.38	0.63
TLBL_15 P	17	1542190	77330461	del	0.00	NA	T	T	F	F	F	F	T	yes	700.64	0.62
TLBL_15 P	17	30820176	79356018	dup	3.00	NA	T	F	F	T	T	T	F	yes		
TLBL_15 R	17	28025949	37393835	dup	3.00	NA	F	F	F	F	F	T	T	no (R)	17.12	0.83
TLBL_15 R	17	37403231	79094836	LOH	2.00	NA	T	NA	NA	F	F	F	T	yes	Inf	0.73
TLBL_15 R	22	14500000	51304566	dup	3.00	NA	F	F	F	F	F	T	T	yes	43.50	0.28

Table S13: Detailed CNV calling results for data set 4. Results for common tools (1: ExomeCNV; 2: VarScan2; 3: ExomeDepth; 4: ControlFREEC Wilkoxon Rank Sum; 5: ControlFREEC Kolmogorov Smirnov; 6: CNV-seq; T: detected; F: missed; NA: not detectable) in comparison to CopyDetective (no (R): region size below detection threshold; no (F): frequency below detection threshold; no (R+F): region size and frequency below detection threshold; quality (maximum for split calls); $\widehat{CF}$: CF estimated by CopyDetective, intervals for split calls).

					CNV		Common tools						CopyDetective			
Sample	chr	start	end	type	value	CF	1	2	3	4	5	6	Called	>thresholds	quality	$\widehat{CF}$
NMZL_01	17	44191122	44432028	del	1.78	0.22	F	F	F	F	F	F	F	no (R+F)		
NMZL_02	9	11996146	12129928	del	1.33	0.67	F	F	F	F	F	F	F	no (R)		
NMZL_02	11	134831902	134942654	dup	2.19	0.19	F	F	F	F	F	F	F	no (R+F)		
NMZL_02	12	149960	133778178	dup	2.47	0.47	T	T	T	F	F	T	T	yes	63.31	0.76
NMZL_03	2	3221311	3311490	dup	2.56	0.56	F	F	F	F	F	F	F	no (R)		
NMZL_03	11	50344320	50772878	dup	2.45	0.45	F	F	F	F	F	F	F	no (R)		
NMZL_03	12	149960	133778178	dup	2.47	0.47	T	T	T	F	F	T	T	yes	76.75	0.45
NMZL_03	14	49901878	67870218	LOH	1.99	0.01	T	NA	NA	F	F	F	F	yes		
NMZL_03	14	69381267	106072048	del	1.42	0.58	T	T	T	F	F	F	T	yes	196.61	0.54
NMZL_04	1	1635464	34255911	del	1.57	0.44	T	T	T	F	T	F	T	yes	31.20	0.41
NMZL_04	1	145431957	164182613	dup	2.35	0.35	F	F	T	F	T	T	T	yes	10.76	0.33
NMZL_04	2	202170582	205029513	del	1.45	0.55	F	F	T	F	T	F	F	no (R)		
NMZL_04	2	230676644	231659478	del	1.49	0.52	F	F	T	F	F	F	F	no (R)		
NMZL_04	3	105367460	105708546	del	1.48	0.52	F	F	F	F	F	F	F	no (R)		
NMZL_04	4	101824464	103153050	del	1.49	0.51	F	F	T	F	F	F	F	no (R)		
NMZL_04	5	362540	693499	dup	2.18	0.18	F	F	F	F	F	F	F	no (R+F)		
NMZL_04	5	57341241	180790852	LOH	1.99	0.01	T	NA	NA	F	F	F	T	yes	14.93	0.29
NMZL_04	7	66608428	158745274	dup	2.40	0.40	T	T	T	F	F	T	T	yes	16.03	0.41
NMZL_04	7	158745274	159119732	dup	2.78	0.78	T	T	F	F	T	F	F	no (R)		
NMZL_04	9	65567222	141091406	dup	2.40	0.40	T	T	T	F	F	T	T	yes	17.92	0.40
NMZL_04	10	74096836	74651614	del	1.84	0.16	F	F	F	F	F	F	F	no (R+F)		
NMZL_04	13	64212220	64535353	del	1.56	0.44	F	F	F	F	F	F	F	no (R)		
NMZL_04	16	201049	14318175	del	1.54	0.46	F	T	T	F	F	F	T	yes	68.50	0.36
NMZL_04	17	28014327	28509237	del	1.87	0.13	F	F	F	F	F	F	F	no (R+F)		
NMZL_04	17	44166469	81052368	dup	2.38	0.38	T	F	T	F	F	T	T	yes	16.04	0.42
NMZL_04	19	422265	3405029	dup	2.45	0.45	F	F	F	F	F	F	T	no (R)	14.96	0.37
NMZL_04	19	5524198	8079060	del	1.56	0.44	F	T	T	F	T	F	F	no (R)		
NMZL_05	3	61892	197918154	dup	2.45	0.45	T	T	T	F	F	T	T	yes	71.19	0.64
NMZL_05	12	149960	133778178	dup	2.42	0.42	T	T	T	F	F	T	T	yes	65.56	0.72
NMZL_05	14	40890845	41392587	del	1.32	0.68	F	F	F	F	F	F	F	no (R)		
NMZL_05	17	44164450	44363240	dup	2.58	0.58	F	F	F	F	F	F	T	no (R)	26.22	0.36
NMZL_05	18	31736	78014594	dup	2.46	0.46	T	T	T	F	T	T	T	yes	22.87	0.80
NMZL_05	22	22734859	23240124	del	1.50	0.50	F	F	T	F	F	F	F	no (R)		
NMZL_06	1	111456782	111650173	LOH	1.97	0.03	T	NA	NA	F	F	F	F	no (R)		
NMZL_06	6	31492934	33173042	del	1.52	0.48	F	F	T	F	T	F	T	no (R)	39.50	0.24
NMZL_06	11	48279770	51345043	LOH	1.96	0.04	T	NA	NA	F	T	F	T	no (R)	11.27	0.25
NMZL_06	12	149960	133778178	dup	2.37	0.37	T	T	T	F	F	T	T	yes	80.29	0.61
NMZL_06	17	43655875	44379292	LOH	2.02	0.02	T	NA	NA	T	T	F	F	no (R)		
NMZL_07	1	1029318	8292861	del	1.81	0.19	F	F	T	F	F	F	F	no (F)		
NMZL_07	6	26319194	27174526	LOH	1.95	0.05	T	NA	NA	F	F	F	F	no (R)		
NMZL_07	10	47479902	47704108	del	1.43	0.57	F	F	F	F	F	F	F	no (R)		
NMZL_07	10	129448413	129527366	del	1.42	0.58	F	F	F	F	F	F	F	no (R)		
NMZL_07	10	135242994	135377274	dup	2.47	0.47	F	F	F	F	F	F	T	no (R)	4.91	0.87
NMZL_07	11	57650938	59949802	del	1.84	0.17	F	F	F	F	F	F	T	no (R+F)		
NMZL_07	11	82369024	83718294	del	1.75	0.25	F	F	T	F	F	F	F	no (R+F)		
NMZL_07	13	51075442	57672696	LOH	2.00	0.01	T	NA	NA	F	T	F	F	yes		
NMZL_07	15	62147986	98119101	del	1.76	0.24	T	F	T	F	F	F	F	no (F)		
NMZL_07	16	61576	22452873	LOH	2.02	0.02	T	NA	NA	F	T	F	T	yes	57.13	0.26
NMZL_07	17	44164450	44379292	del	1.74	0.26	F	F	F	F	F	F	T	no (R+F)	15.59	0.26
NMZL_07	22	38842570	39291027	LOH	1.97	0.03	T	NA	NA	F	T	F	F	no (R)		
NMZL_08	1	248795570	249228414	LOH	2.04	0.04	T	NA	NA	T	T	F	F	no (R)		
NMZL_08	3	197575140	197918154	LOH	2.00	0.00	T	NA	NA	T	T	F	F	no (R)		
NMZL_08	7	158737284	159119732	LOH	2.00	0.00	T	NA	NA	F	T	F	T	no (R)	6.00	0.36
NMZL_08	11	54701512	55374020	LOH	1.94	0.06	T	NA	NA	F	T	F	F	no (R)		
NMZL_09	1	145376959	203175268	dup	2.31	0.31	F	F	T	F	F	T	T	yes	13.93	0.34
NMZL_09	6	79038408	171051066	LOH	1.99	0.01	T	NA	NA	F	F	F	T	yes	163.32	0.42
NMZL_09	7	118136222	135819037	del	1.62	0.38	F	F	T	F	T	F	T	yes	19.69	0.33
NMZL_09	11	54701512	55374020	LOH	1.98	0.02	T	NA	NA	F	T	F	T	no (R)	10.42	0.29
NMZL_09	14	106711284	107149151	LOH	2.01	0.01	T	NA	NA	F	F	F	T	no (R)	7.34	0.25
NMZL_10	8	5889697	6100568	del	1.30	0.70	F	F	F	F	F	F	F	no (R)		
NMZL_11	9	12060	16984678	del	1.75	0.25	T	F	F	F	F	F	F	no (F)		
NMZL_11	20	57478138	57653532	LOH	1.97	0.03	T	NA	NA	F	F	F	F	no (R)		
NMZL_12	3	4717379	5530420	dup	2.38	0.38	F	F	F	F	T	T	F	no (R)		
NMZL_12	10	143850	335510	del	1.80	0.20	F	F	F	F	F	F	F	no (R+F)		
NMZL_13	4	92302915	92463686	del	1.32	0.68	F	F	F	F	F	F	F	no (R)		
NMZL_13	15	20016316	102440177	LOH	2.00	0.00	T	NA	NA	F	F	F	T	yes	135.88	0.41
NMZL_14	2	42598312	48732682	del	1.88	0.12	F	F	F	F	F	F	F	no (F)		
NMZL_14	2	122107879	136694748	LOH	1.93	0.07	T	NA	NA	F	F	F	T	yes	10.18	0.27
NMZL_14	2	201239460	201455939	del	1.85	0.15	F	F	F	F	F	F	F	no (R+F)		
NMZL_14	3	129806265	139162961	LOH	1.99	0.01	T	NA	NA	F	F	F	T	yes	8.65	0.33
NMZL_14	3	135109308	135666466	del	1.36	0.64	F	F	F	F	F	F	T	no (R)	8.65	0.33
NMZL_14	4	10236942	10395844	dup	2.15	0.15	F	F	F	F	F	F	F	no (R+F)		
NMZL_14	5	10554462	14279842	del	1.84	0.16	F	F	F	F	F	F	F	no (R+F)		
NMZL_14	5	38934075	50074947	del	1.86	0.14	F	F	F	F	F	F	F	no (F)		
NMZL_14	6	144909812	145156456	del	1.89	0.11	F	F	F	F	F	F	F	no (R+F)		
NMZL_14	7	158404952	158694096	LOH	1.97	0.03	T	NA	NA	F	F	F	F	no (R)		
NMZL_14	8	17757395	17899168	del	1.87	0.13	F	F	F	F	F	F	T	no (R+F)	7.22	0.26
NMZL_14	8	47352711	68416320	LOH	2.00	0.00	T	NA	NA	T	F	F	F	yes		
NMZL_14	8	91875344	92152064	del	1.89	0.11	F	F	F	F	F	F	F	no (R+F)		

NMZL_14	10	64281177	65407432	del	1.53	0.47	F	F	F	F	F	F	F	no (R)		
NMZL_14	10	91449820	94764685	del	1.88	0.12	F	F	F	F	F	F	F	no (R+F)		
NMZL_14	11	28013832	49554770	LOH	2.00	0.00	T	NA	NA	T	T	F	F	yes		
NMZL_14	12	9795227	9933259	del	1.75	0.25	F	F	F	F	F	F	F	no (R+F)		
NMZL_14	13	91923467	92069166	del	1.90	0.10	F	F	F	F	F	F	F	no (R+F)		
NMZL_14	14	39492746	39904192	del	1.88	0.12	F	F	F	F	F	F	F	no (R+F)		
NMZL_14	14	59986165	60361272	LOH	2.05	0.05	T	NA	NA	F	F	F	F	no (R)		
NMZL_14	14	106324327	107086497	del	1.54	0.46	T	T	T	F	F	F	F	no (R)		
NMZL_14	15	85830322	86269499	del	1.79	0.21	F	F	F	F	F	F	F	no (R+F)		
NMZL_14	16	61576	28045978	LOH	2.08	0.08	T	NA	NA	F	F	F	T	yes	134.32	0.39
NMZL_14	17	37906626	44363240	LOH	1.97	0.03	T	NA	NA	T	T	F	F	yes		
NMZL_14	20	29420340	30121589	LOH	2.07	0.07	T	NA	NA	F	F	F	F	no (R)		
NMZL_14	22	34178178	34220626	LOH	2.07	0.07	T	NA	NA	F	F	F	F	no (R)		
NMZL_15	2	107984237	145329210	del	1.82	0.19	F	T	T	F	T	F	T	no (F)	16.74	0.24
NMZL_15	3	105417429	125070822	LOH	1.92	0.08	F	NA	NA	F	F	F	F	yes		
NMZL_15	5	102065072	102555349	del	1.87	0.13	F	F	F	F	F	F	F	no (R+F)		
NMZL_15	7	8486136	8633323	dup	2.12	0.12	F	F	F	F	F	F	F	no (R+F)		
NMZL_15	8	46839736	47831854	dup	2.11	0.11	F	F	F	F	F	T	F	no (R+F)		
NMZL_15	9	3246799	7151558	del	1.82	0.18	F	F	F	T	T	F	F	no (R+F)		
NMZL_15	10	63701462	64037914	del	1.79	0.21	F	F	F	F	F	F	F	no (R+F)		
NMZL_15	10	104992491	106149286	LOH	1.97	0.03	T	NA	NA	F	T	F	F	no (R)		
NMZL_15	11	54701512	55374020	LOH	1.97	0.03	T	NA	NA	F	F	F	T	no (R)	13.58	0.21
NMZL_15	11	83004760	84051472	LOH	2.00	0.00	T	NA	NA	F	F	F	F	no (R)		
NMZL_15	11	106592418	106785747	LOH	1.97	0.03	T	NA	NA	F	F	F	F	no (R)		
NMZL_15	12	632494	697354	dup	2.19	0.19	F	F	F	F	F	F	F	no (R+F)		
NMZL_15	12	111825717	112926665	LOH	1.90	0.10	T	NA	NA	F	T	F	F	no (R)		
NMZL_15	13	73205530	73575625	LOH	1.92	0.08	T	NA	NA	F	F	F	F	no (R)		
NMZL_15	15	62128324	62365340	del	1.81	0.20	F	F	F	F	F	F	F	no (R+F)		
NMZL_15	18	61828344	62113371	dup	2.18	0.18	T	T	F	F	F	F	T	no (R+F)	9.70	0.32
NMZL_15	18	62113371	62271897	dup	3.44	1.44	T	T	F	F	F	F	T	no (R)	9.70	0.32
NMZL_15	19	29909402	30290984	dup	2.19	0.19	F	F	F	F	F	F	F	no (R+F)		
NMZL_15	20	25561996	26305590	LOH	1.91	0.09	T	NA	NA	F	T	F	F	no (R)		
NMZL_16	1	101680512	120541024	del	1.44	0.56	T	T	T	T	T	F	T	yes	281.23	0.57
NMZL_16	1	151396729	176028862	del	1.43	0.57	T	T	T	T	T	F	T	yes	238.69	0.65
NMZL_16	3	122296914	197707662	dup	2.41	0.41	T	T	T	T	T	T	T	yes	152.66	0.55
NMZL_16	6	14703716	15285855	LOH	1.93	0.07	T	NA	NA	F	T	F	F	no (R)		
NMZL_16	8	98700370	98980811	del	1.85	0.15	F	F	F	F	F	F	F	no (R+F)		
NMZL_16	9	28614442	28731517	del	1.30	0.70	F	F	F	F	F	F	F	no (R)		
NMZL_16	10	22687001	23018572	LOH	2.06	0.06	T	NA	NA	F	F	F	F	no (R)		
NMZL_16	11	54965523	55374020	del	1.85	0.15	T	F	F	F	F	F	F	no (R+F)		
NMZL_16	15	102151430	102348396	dup	2.17	0.17	F	F	F	F	F	F	F	no (R+F)		
NMZL_17	7	11237034	11361690	del	1.33	0.67	F	F	F	F	F	F	F	no (R)		
NMZL_17	7	57368564	58025898	LOH	1.97	0.03	T	NA	NA	F	T	F	F	no (R)		
NMZL_17	8	6797235	7063586	LOH	1.99	0.01	T	NA	NA	F	F	F	F	no (R)		
NMZL_17	10	47408545	47704612	dup	2.53	0.53	F	F	F	F	F	F	F	no (R)		
NMZL_17	19	34724907	58949508	LOH	1.97	0.03	T	NA	NA	T	T	F	T	yes	368.52	0.27
NMZL_18	6	137720998	138962606	del	1.60	0.40	T	T	T	F	T	F	T	no (R)	7.07	0.25
NMZL_18	14	106569247	107145419	del	1.71	0.29	T	T	T	F	T	F	T	no (R+F)	10.63	0.27
NMZL_18	14	107184263	107285449	dup	2.19	0.19	F	F	F	F	F	F	T	no (R+F)	5.29	0.25
NMZL_18	18	33769548	78014594	dup	2.36	0.36	T	T	T	T	T	T	T	yes	47.19	0.41
NMZL_18	22	24204664	24278979	LOH	2.07	0.07	T	NA	NA	F	T	F	F	no (R)		

2.5 CNV calling using the exact approach

Prior to actual CNV calling, CopyDetective performs quality analysis of every sample. Two different approaches are available for this step: a simulation approach and an exact approach. While the simulation approach simulates coverage and VAF of heterozygous polymorphisms (100 SNPs, simulation: 500 times), all detected heterozygous polymorphisms are evaluated in the framework of the exact approach. A comparison of the detection thresholds can be found in Figure S8.

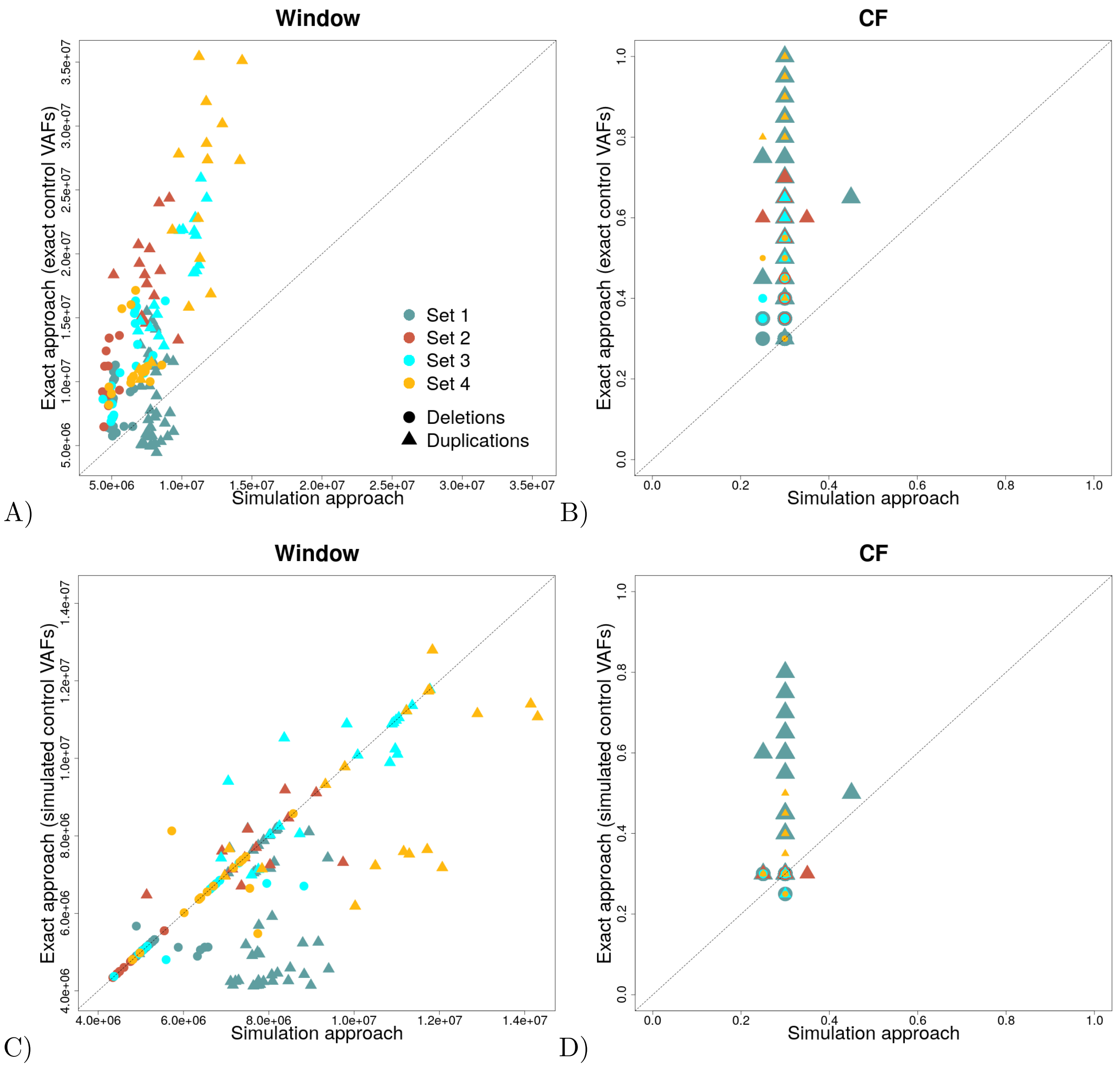

Figure S8: Relation between detection thresholds comparing the simulation approach to the exact approach. A) Window size (exact VAFs for the control sample). B) CF (exact VAFs for the control sample). C) Window (simulated VAFs for the control sample). D) CF (simulated VAFs for the control sample).

It can be observed that data sets 2, 3 and 4 have higher thresholds for window size and CF (see Figure S8A and B). Data set 1 shows a tendency towards higher thresholds for window size and higher thresholds for CF. This overall shift to higher values is unexpected. Instead, we expected results comparable to the simulation approach.

Detailed analyses show that the reason for this observation lies in the VAFs of heterozygous

polymorphisms detected in the control samples. The expected value is 0.5. In our data, however, the average is <0.5 (range: [0.4676-0.4900]). A VAF of 0.4676 corresponds to $CF_{Del} = 0.1217$ and to $CF_{Dup} = 0.1386$. As all control samples are affected by average VAFs below 0.5, it is unlikely that this observation results from actual CNVs present in all control samples. Instead, it is more likely that our polymorphism calling approach filters high-frequency SNPs more strictly compared to low-frequency SNPs. The decreased average VAF of polymorphisms in our control samples is likely to lead to higher detection thresholds as can be observed in Figure S8A and B.

If our assumption is correct, we should observe results comparable to the simulation approach if the VAFs of heterozygous polymorphisms in the control samples are simulated with an expected value of 0.5. Figure S8C and D visualize the detection thresholds. It can be observed that the results are indeed comparable. Although differences can be observed, these are no longer systematic. For data set 1 we observed lower detection thresholds for window size, but higher values for CF. Thus, we do not expect a major difference in the CNV calling results. For both exact and simulated VAFs for the control sample, the CNV calling results are summed up in Table S14.

Table S14: Performance of CopyDetective (raw, i.e. without optional final filtration, and filter, i.e. with default filtration threshold of 10.76) using the exact approach for quality analysis. For VAFs of heterozygous polymorphisms in the control samples two options are considered: exact and simulated. True positive (TP) calls (in brackets: reporting the number of additional true positive calls if CNV type is not evaluated), false positive (FP) calls, found, missed and detectable CNVs, sensitivity (sens; just evaluating true positive calls with correct CNV type), positive predictive value (PPV; just evaluating true positive calls with correct CNV type) and the F1 score.

Config		Control VAF	Data set	TP calls (+ false type)	FP calls	CNVs found	missed	detectable	Sens	PPV	F1
raw		exact	1	28 (+13)	182	16	1	17	0.94	0.13	0.23
			2	43 (+27)	24	18	2	20	0.90	0.64	0.75
			3	54 (+15)	39	35	3	38	0.92	0.58	0.71
			4	7 (+4)	20	4	0	4	1.00	0.26	0.41
		simulated	1	27 (+21)	558	16	1	17	0.94	0.05	0.09
			2	63* (+42)	181	34	3	37	0.92	0.26	0.41
			3	68 (+22)	216	40	1	41	0.98	0.24	0.38
			4	20 (+23)	394	17	0	17	1.00	0.05	0.09
filter	10.76	exact	1	25 (+12)	40	15	2	17	0.88	0.38	0.54
			2	41 (+22)	7	18	2	20	0.90	0.85	0.88
			3	52 (+15)	6	35	3	38	0.92	0.92	0.92
			4	7 (+3)	7	4	0	4	1.00	0.50	0.67
		simulated	1	21 (+15)	125	16	1	17	0.94	0.15	0.26
			2	52 (+31)	65	34	3	37	0.92	0.44	0.60
			3	60 (+18)	11	40	1	41	0.98	0.85	0.91
			4	20 (+16)	45	17	0	17	1.00	0.31	0.47

*One additional CNV is overlapping a true CNV. However as the called CNV is clearly shorter than the validated one and characterized by a remarkably low quality value, we assume that this overlap is just occurring at coincidence. Therefore, it is counted as 'missed'.

It can be observed that a lower number of CNVs is categorized as 'detectable' when using the exact approach for quality analysis, combined with the exact VAFs for heterozygous polymorphisms in the control samples. Only 79 CNVs are detectable compared to 116 CNVs in case of the simulation approach. This decrease in detectable CNVs results from the high detection thresholds. Sensitivity only shows a minor decrease and is still close to 0.95. This observation is expected as quality analysis aims at realizing user-defined sensitivity of 0.95. PPV shows an increase resulting from stricter detection thresholds.

When using the exact approach for quality analysis, combined with simulated VAFs for

heterozygous polymorphisms in the control samples, a considerable increase in the number of detectable CNVs can be observed. One hundred and twelve CNVs are categorized as ‘detectable’, sensitivity is 0.96 over all data sets. These results are highly comparable to the simulation approach. Same is true for the number of false positive calls and PPV.

2.6 Relation between performance and quality filtration

Optional step 4 of CopyDetective – the 'Filtration' – provides an option for excluding low-quality calls. To investigate the effect of this option on CopyDetective's performance, we analyze the relation between sensitivity and PPV in the context of an increasing quality threshold applied to the list of merged calls for every data set. The results, also considering the common approaches ExomeCNV, VarScan2, ExomeDepth, ControlFREEC (WR and KS) and CNV-seq, are visualized in Figure S9. Note that different from ROC curves, we plot sensitivity vs PPV (and not sensitivity vs 1-specificity=1-TN/(FP+TN)). Thus, if a true positive call is excluded by filtration, i.e. sensitivity decreases, a (minor) decrease in PPV is expected as it is defined as TP/(TP+FP).

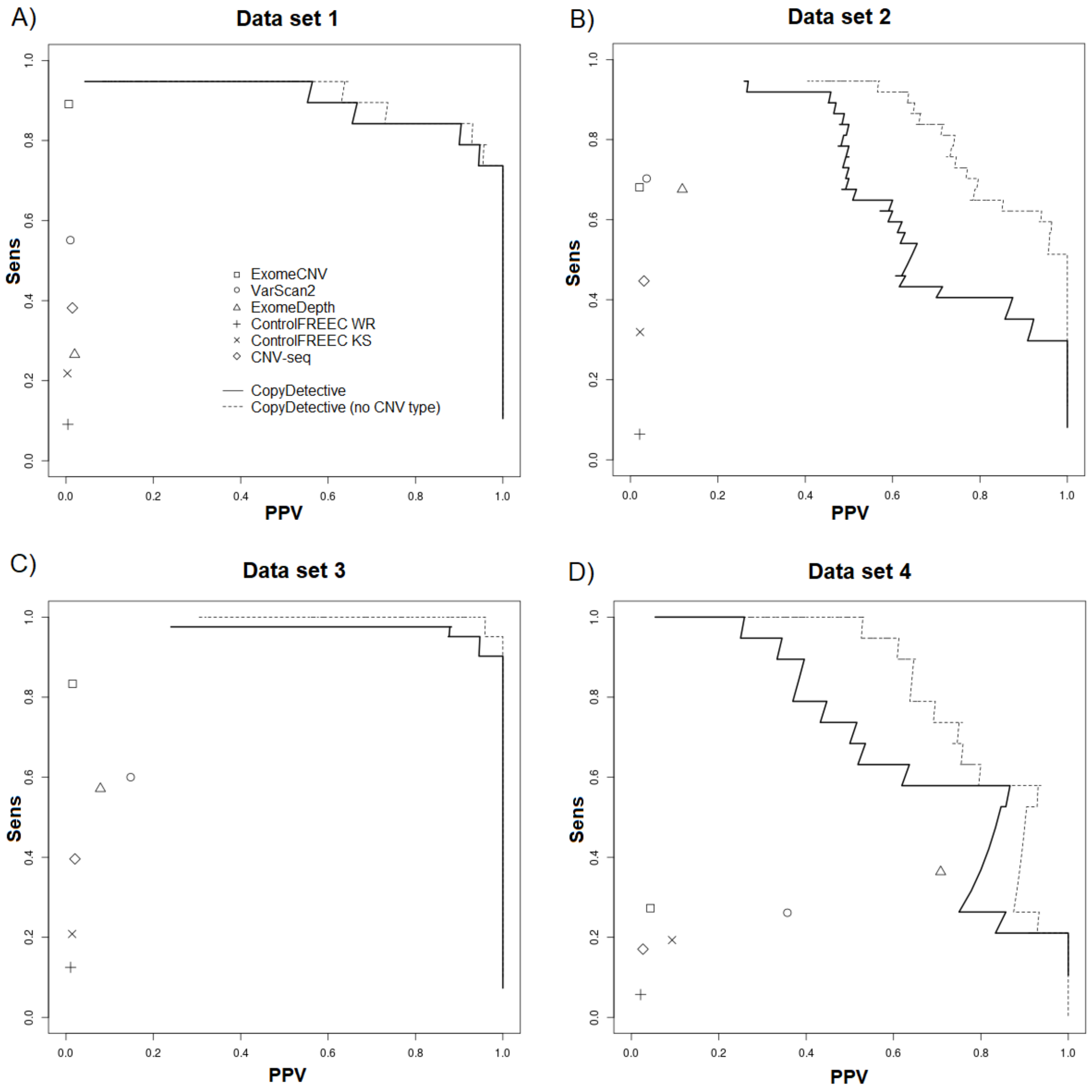

Figure S9: Relation between sensitivity (sens) and positive predictive value (PPV) in case of ExomeCNV, VarScan2, ExomeDepth, ControlFREEC (WR and KS), CNV-seq and CopyDetective (with and without evaluation of the CNV type). For CopyDetective, performance is considered in the context of an increasing quality threshold for CNV calls. A) Data set 1. B) Data set 2. C) Data set 3. D) Data set 4.

It can be observed that filtering low-quality calls has a positive effect on PPV. Regarding sensitivity, all data sets share an initial phase of unchanged sensitivity in the presence of an increasing quality threshold. While this phase is relatively short for data set 2, it is especially long for data set 3.

In Figure 4 of the main manuscript the quality values for true positive and false positive CNV calls reported by CopyDetective are visualized. The lowest and highest quality values for true positive vs false positive calls are summed up in Table S15.

Table S15: Lowest and highest quality values for true positive vs false positive CNV calls reported by CopyDetective (evaluating the true CNV call with the highest quality value in case more than one CNV call overlaps a validated CNV).

Data set	TP calls quality values		FP calls quality values	
	lowest	highest	lowest	highest
1	30.89	550.51	4.39	87.01
2	11.19	686.47	4.39	158.29
3	11.48	700.64	4.39	21.43
4	10.76	312.40	4.38	145.71

It can be observed that the quality values for true and false positive calls overlap in case of every data set. Thus, we can just define a range for the optimum filtration threshold for every data set, e.g. 30.89-87.01 for data set 1. Comparing the different ranges for the four data sets, we identify 10.76 as our default threshold, filtering not a single true positive call in case of any data set, while still filtering false positive calls.

2.7 Evaluating coordinates

The coordinates of the CNVs reported by CopyDetective resemble the coordinates based on validation experiments quite well. Figure S10 shows the overlap of the validated CNVs with the CNVs called by CopyDetective.

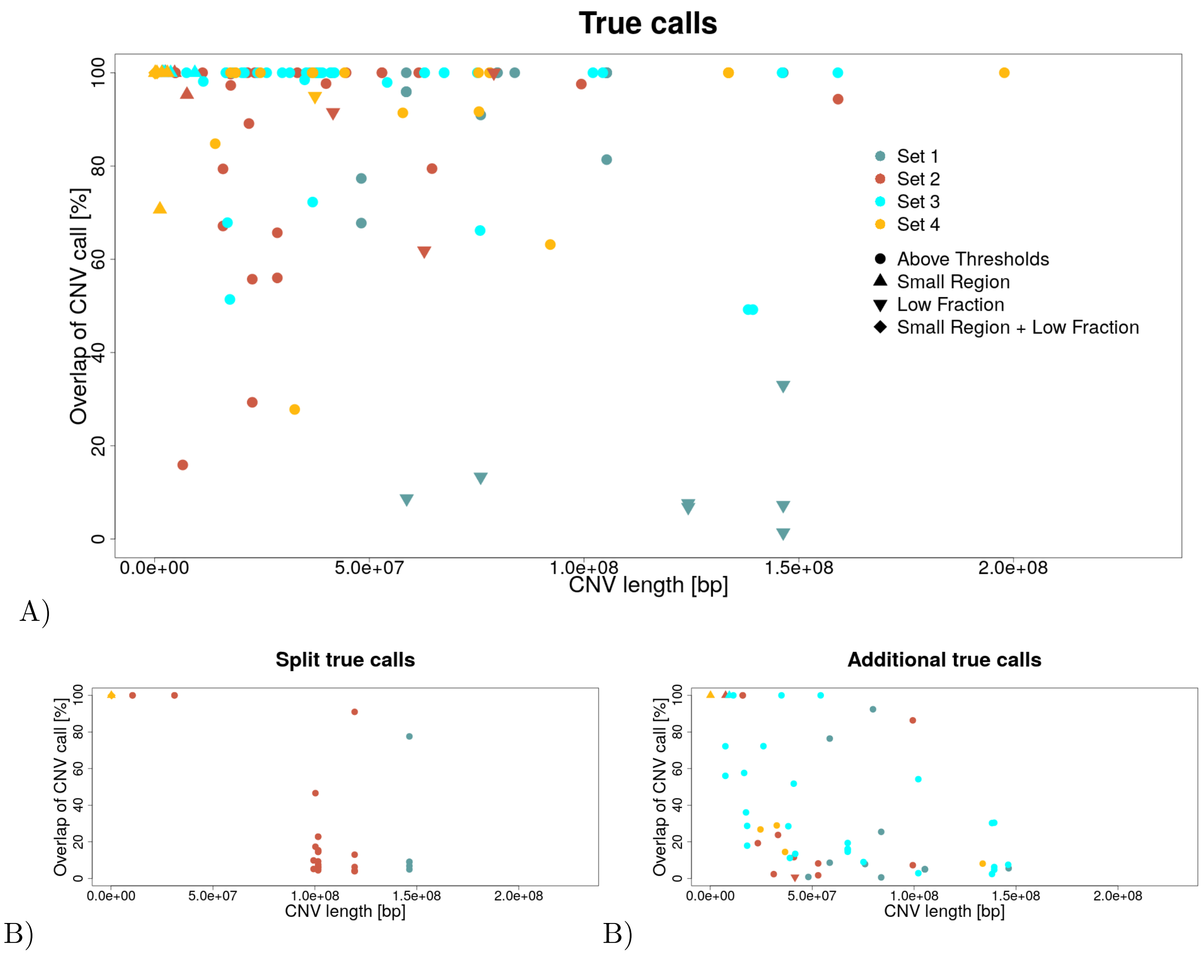

Figure S10: Overlap of the CNVs reported by CopyDetective with the validated CNVs. A) True calls, i.e. best matching calls. B) Split true calls. C) Additional true calls, i.e. true but not the best matching calls.

It can be observed that for a majority of true calls, i.e. the best matching call reported by CopyDetective, overlap is 100% or close to 100% (Figure S10A). A decreased overlap usually goes along with a low fraction of cells being affected by a CNV. This observation is expected. A CNV affecting a certain fraction of cells below the actual detection threshold can only be detected if – due to regional variation – part of the CNV is characterized by a fraction of cells above the threshold. Thus, CopyDetective is only able to detect and report this small part of the CNV.

For split true calls (Figure S10B) it can be observed that the overlap is usually, as expected, <100%. It is distributed over the different CNVs reported by CopyDetective. Detailed evaluation of the results in comparison to the SNP array output used for validation shows, that the split CNVs reported by CopyDetective may actually be correct. One CNV in data set 2 and one in data set 4 is an exception from this observation. In these two cases, the validated CNV is “split”, but just reported as one by CopyDetective. Therefore, two relatively small CNVs with 100% overlap can be observed.

Additional true calls are defined as true CNV calls, i.e. they are overlapping the validated CNV, but they are not the best matching calls reported by CopyDetective. As expected, their overlap is usually <100%.

In addition to the overlap, we investigate the deviation of the validated start and end positions from the start and end positions reported by CopyDetective. The relative deviation based on chromosome lengths is investigated and visualized in Figure S11.

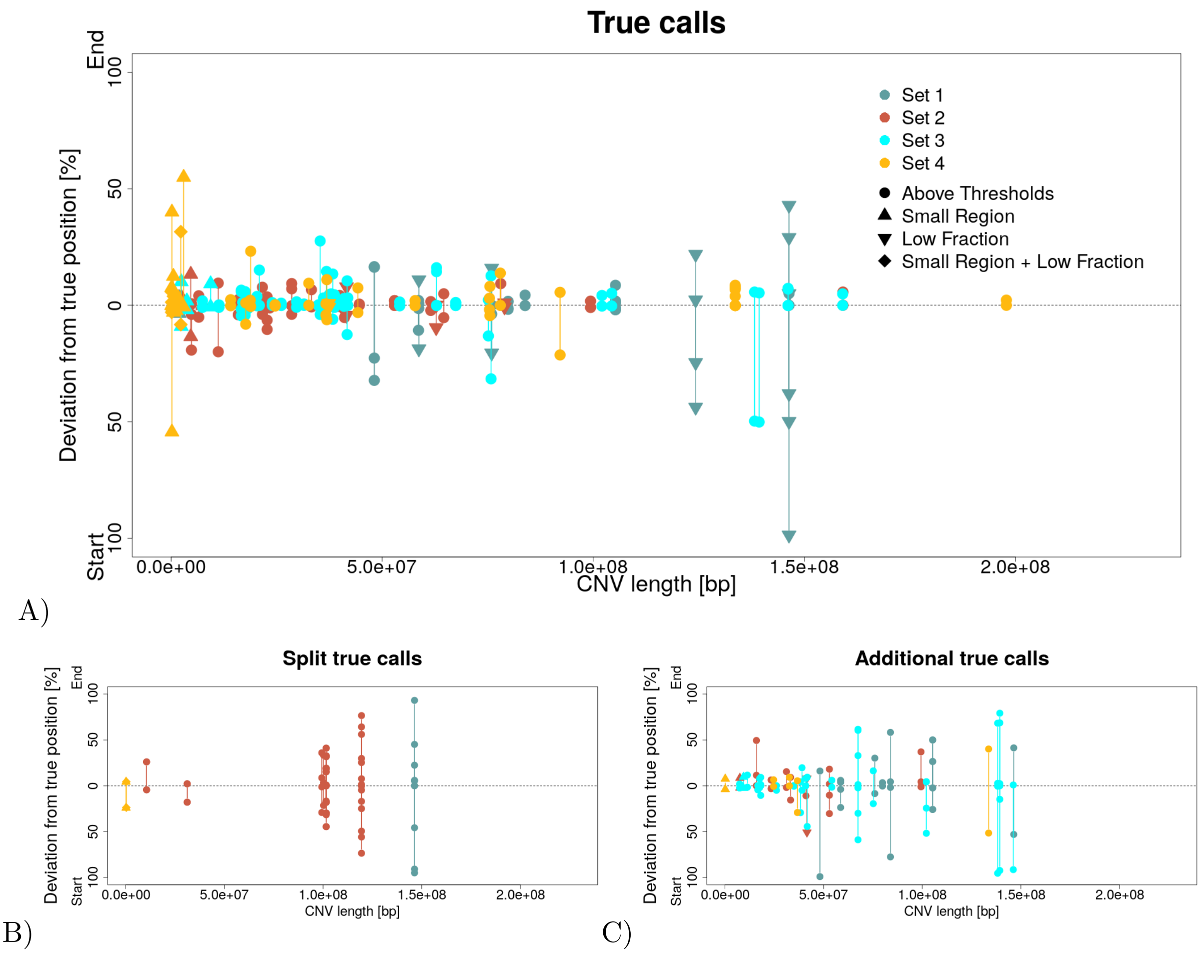

Figure S11: Relative deviation of the true start and end positions from the start and end positions reported by CopyDetective. A) True calls, i.e. best matching calls. B) Split true calls. C) Additional true calls, i.e. true but not the best matching calls.

It can be observed that a majority of calls has a start and end position close to the positions based on validation experiments. Deviations can in most cases only be observed for CNVs with a fraction of cells below the actual detection threshold. As these calls are also characterized by a low overlap with the true CNV (see Figure S10A), the huge deviation observed in Figure S11A is expected.

For split true calls (Figure S11B) it can be observed that every split call is characterized by at least one start and one end position close to the true start/end position. These results indicate that the split CNV actually covers the true CNV.

Additional true calls (Figure S11C) are partly characterized by start and/or end positions close to the true ones, and partly by huge deviations from the true coordinates.

2.8 Evaluating cell fractions

Evaluating CopyDetective's accuracy in estimating cell fractions, we consider the estimated cell fractions for data sets 1, 2 and 4 in comparison to the true cell fractions (see Figure S12).

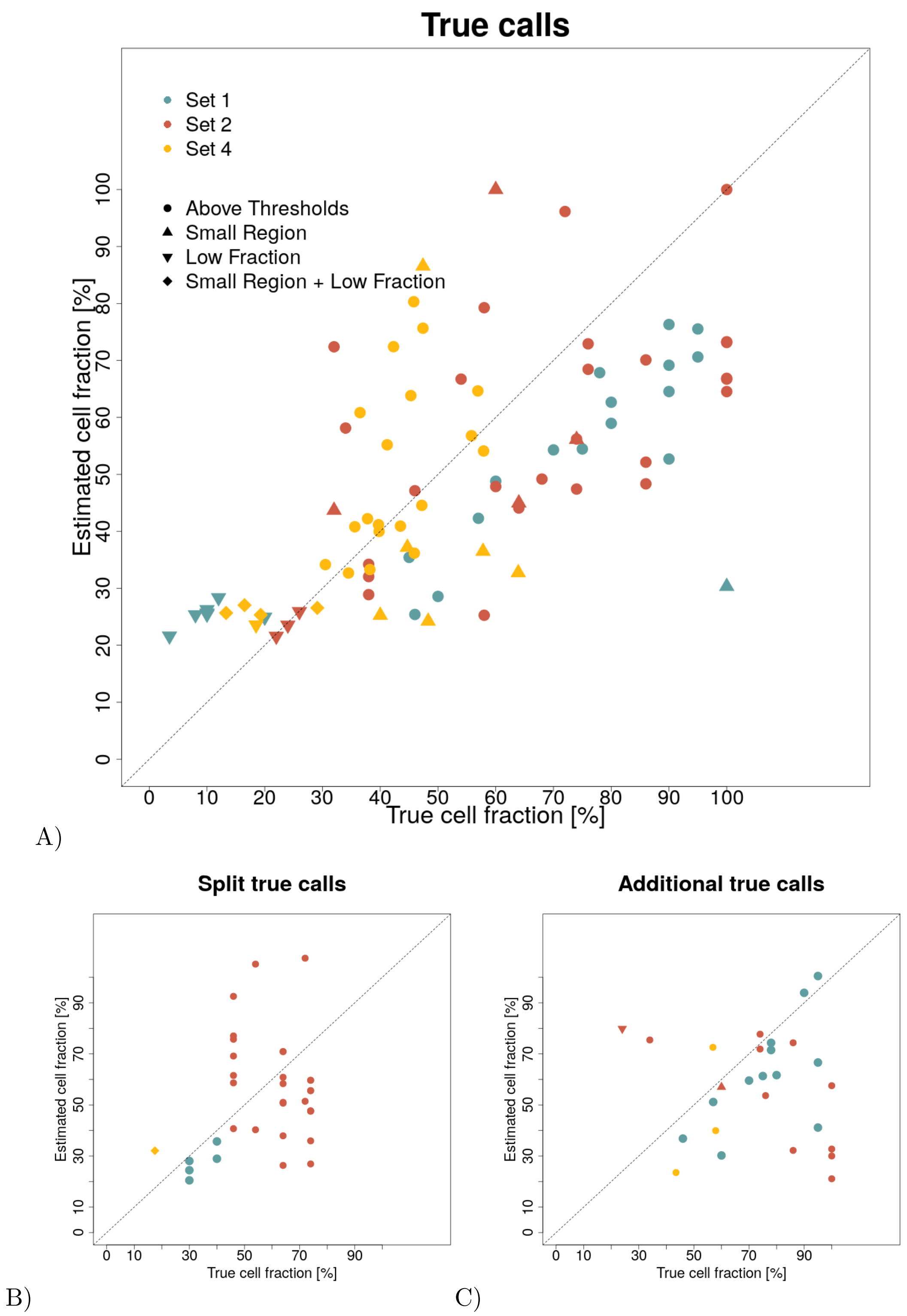

Figure S12: Relation between estimated cell fractions reported by CopyDetective and the true cell fractions. A) True calls, i.e. best matching calls. B) Split true calls. C) Additional true calls, i.e. true but not the best matching calls.

For the true, best matching calls it can be observed that the estimated cell fractions reported by CopyDetective generally match the true cell fractions. This is the case for data sets 1 and 2, with the true cell fractions being based on clonal evolution analysis, as well as data set 4, with the true cell fractions being solely based on the reported logR values and thus, excluding LOH.

For CNVs present in a low fraction of cells it can be observed that the estimated CFs are correct for data set 2. For data sets 1 and 4, it is overestimated slightly. This observation is expected as only small regions with locally increased cell fractions can be detected and reported by CopyDetective.

For CNVs affecting a small region it can be observed that the estimated CFs usually show greater deviation from the true CFs for all data sets.

Estimated CFs of split true calls (Figure S12B) show greater deviation, over- and underestimating the true CFs. However, if a weighted mean was calculated for the split calls belonging to one true CNV, data indicates that the resulting cell fraction would be close to the true one.

As expected, additional true calls (Figure S12C) are mostly characterized by great deviation of the estimated cell fractions from the true ones.

2.9 Robustness of the CNV calling results

2.9.1 Accuracy of polymorphism calling

Polymorphism calling is performed using VarDict. Subsequently, we filter the raw results for being synonymous variants and/or being present in common polymorphism databases.

We expect that both, false positive and false negative polymorphism calls, can possibly harm CNV calling results with CopyDetective. To explore the extent by which errors in polymorphism calling affect CNV calling with our novel approach, we consider two scenarios. First, false negative calls are simulated. We randomly exclude 5% of the polymorphism calls for every sample. The relation between detection thresholds, comparing 0% vs 5% false negative polymorphism calls is visualized in Figure S13.

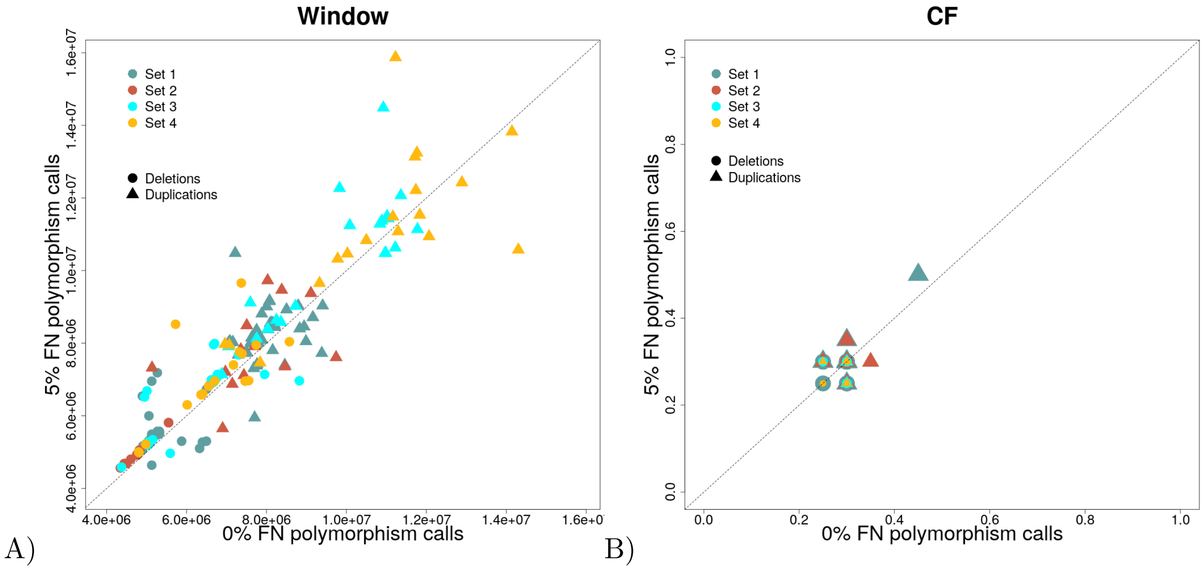

Figure S13: Relation between detection thresholds comparing 0% vs 5% false negative polymorphism calls. A) Window size. B) CF.

It can be observed that the detection thresholds, especially the window size differs in most of the cases. However, a systematic increase, due to fewer SNPs being analyzed, cannot be observed. CNV calling results are summed up in Table S16.

Although the CNV calling results show some differences compared to Table 2 in the main manuscript, the general performance of CopyDetective hardly changes.

In addition to false negative polymorphism calls, we also consider false positive polymorphisms. We randomly add novel variant calls (5% of the original number of polymorphisms called per sample) to our data. We assume a uniform distribution to simulate the position in the target region (whole exome), a log-normal distribution to simulate depth and a normal distribution to simulate VAF in the control sample. Mean and standard deviation are estimated on the basis of the real data. For the case sample, we use a log-normal distribution to simulate depth, and a uniform distribution to simulate VAF, as all values between 0 and 1 are assumed to be equally likely. The relation between detection thresholds, comparing 0% vs 5% false positive polymorphism calls is visualized in Figure S14.

Similar to the analysis of false negative SNPs, we also observe differences in the detection thresholds when simulating false positive SNPs. However, both higher as well as lower detection thresholds can be observed. For window size a minor change to smaller values can be observed. CNV calling results are summed up in Table S16.

In all data sets, it can be observed that the number of false positive CNV calls increases. A

Table S16: Performance of CopyDetective (raw, i.e. without optional final filtration, and filter, i.e. with default filtration threshold of 10.76) simulating 5% false negative polymorphism calls. True positive (TP) calls (in brackets: reporting the number of additional true positive calls if CNV type is not evaluated), false positive (FP) calls, found, missed and detectable CNVs, sensitivity (sens; just evaluating true positive calls with correct CNV type), positive predictive value (PPV; just evaluating true positive calls with correct CNV type) and the F1 score.

Tool	Config		Data set	TP calls (+ false type)	FP calls	CNVs found	CNVs missed	CNVs detectable	Sens	PPV	F1
CopyDetective	raw		1	31 (+18)	755	17	1	18	0.94	0.04	0.08
			2	66* (+44)	170	34	3	37	0.92	0.28	0.43
			3	65 (+27)	206	39	1	40	0.98	0.24	0.38
			4	23 (+26)	390	19	0	19	1.00	0.06	0.11
CopyDetective	filter	10.76	1	29 (+15)	170	17	1	18	0.94	0.15	0.25
		10.76	2	53 (+31)	53	33	4	37	0.89	0.50	0.64
		10.76	3	57 (+22)	10	38	2	40	0.95	0.85	0.90
		10.76	4	22 (+16)	59	18	1	19	0.95	0.27	0.42

*One additional CNV is overlapping a true CNV. However as the called CNV is clearly shorter than the validated one and characterized by a remarkably low quality value, we assume that this overlap is just occurring at coincidence. Therefore, it is counted as 'missed'.

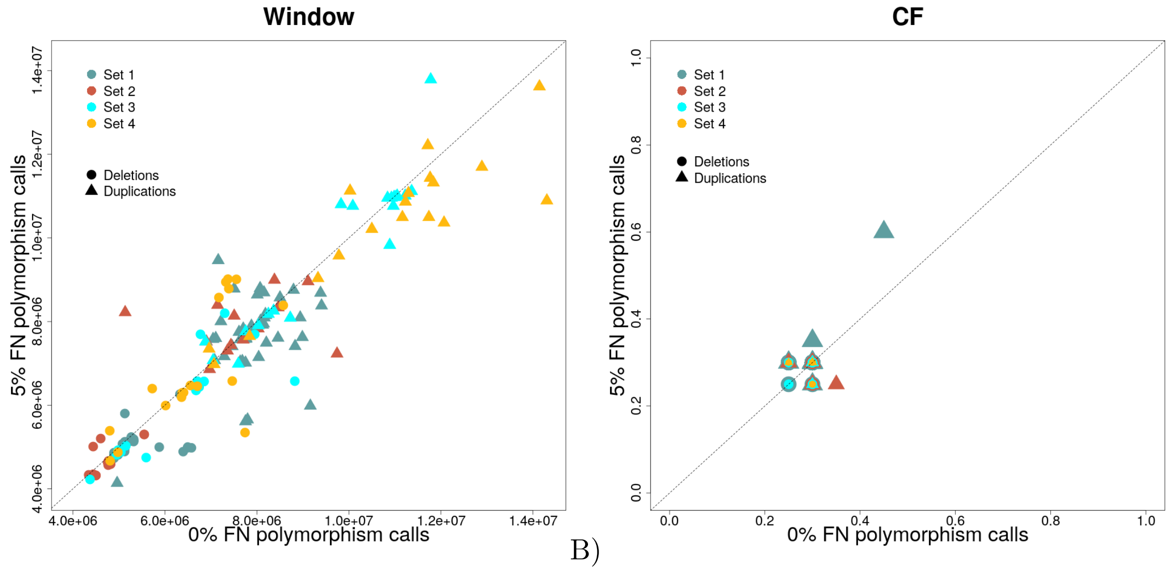

Figure S14: Relation between detection thresholds comparing 0% vs 5% false positive polymorphism calls. A) Window size. B) CF.

majority of false positives can be filtered when applying default quality filtration. However, PPV is still smaller compared to Table 2, main manuscript. Furthermore, data set 3 is characterized by an increased number of missed CNVs.

From our results on the accuracy of polymorphism calling we conclude that CopyDetective is robust towards false negative polymorphism calls. However, false positive polymorphisms can lead to a decrease in PPV. Thus, it appears advisable to apply rather strict filtration when performing polymorphism calling.

Table S17: Performance of CopyDetective (raw, i.e. without optional final filtration, and filter, i.e. with default filtration threshold of 10.76) simulating 5% false positive polymorphism calls. True positive (TP) calls (in brackets: reporting the number of additional true positive calls if CNV type is not evaluated), false positive (FP) calls, found, missed and detectable CNVs, sensitivity (sens; just evaluating true positive calls with correct CNV type), positive predictive value (PPV; just evaluating true positive calls with correct CNV type) and the F1 score.

Tool	Config		Data set	TP calls (+ false type)	FP calls	CNVs found	CNVs missed	CNVs detectable	Sens	PPV	F1
CopyDetective	raw		1	26 (+24)	1896	17	1	18	0.94	0.01	0.03
			2	70* (+45)	373	34	3	37	0.92	0.16	0.27
			3	58 (+22)	522	33	8	41	0.80	0.10	0.18
			4	24 (+26)	641	19	0	19	1.00	0.04	0.07
CopyDetective	filter	10.76	1	25 (+16)	475	17	1	18	0.94	0.05	0.09
		10.76	2	56 (+34)	138	33	4	37	0.89	0.29	0.44
		10.76	3	49 (+18)	83	32	9	41	0.78	0.37	0.50
		10.76	4	22 (+21)	120	18	1	19	0.94	0.15	0.27

*One additional CNV is overlapping a true CNV. However as the called CNV is clearly shorter than the validated one and characterized by a remarkably low quality value, we assume that this overlap is just occurring at coincidence. Therefore, it is counted as 'missed'.

2.9.2 Changing detection thresholds

Prior to actual CNV calling, CopyDetective analyzes every sample's quality and determines detection thresholds for the minimum CNV length and the minimum cell fraction. These thresholds are supposed to ensure that the CNV calling results are characterized by user-defined sensitivity. To analyze the effect of (manual) changes in the thresholds, we consider four cases: 1) all thresholds for minimum CNV length are doubled, 2) all thresholds for minimum cell fraction are doubled, 3) all thresholds for minimum CNV length are halved, 4) all thresholds for minimum cell fraction are halved. The results for CNV calling with CopyDetective – with and without filtration of the merged calls – are summed up in Table S18.

Increasing the window threshold, i.e. the minimum CNV length, it can be observed that the number of detectable CNVs decreases (halved: 124; original: 116; doubled: 105). Similar observations can be made if CF is increased (halved: 125; original: 116; doubled: 82). This observation is expected as the detection thresholds define the number of detectable CNVs.

Regarding missed CNVs, we expect that stricter thresholds will usually not lead to an increase. With two exceptions, data confirm these expectations. One CNV in data set 4 is additionally missed (NMZL_04; chr1:1,635,464-34,255,911 del). The CNV reported by CopyDetective (using original thresholds) is shorter compared to the validated CNV (chr1:1,650,808-10,719,207). By doubling the threshold for window size we evaluate regions of 14,648,334 bp for sample NMZL_04. It appears likely that the bigger evaluated region contains one or more polymorphisms towards its end that do not indicate presence of a CNV. Therefore, the applied test is no longer significant and the CNV is no longer reported.

A second exception is data set 3 with respect to changes in the CF threshold. No information on the precise CF is available for this data set. By increasing the detection thresholds, CopyDetective excludes all CNVs with estimated cell fractions below. It appears likely that the 4 additionally missed CNVs are actually characterized by CFs below the thresholds and should have been excluded from the list of detectable CNVs.

Applying lower thresholds for CNV calling, we expect to observe an increase in the number of missed CNVs and a decrease in sensitivity. Data confirm these expectations. Yet, the observable decrease in sensitivity is only small. The major problem resulting from lower thresholds is the increase in the number of false positive calls and thus, the decrease in PPV. We observe an increase of false positive calls between 45% (halved window; data set 2) and 299% (halved CF; data set 1). Filtration using the default quality filter of 10.76 only removes part of the false positive calls.

Concluding, we observe that the automatically determined thresholds lead to the overall best results. Stricter thresholds may preclude some CNVs from being called. Furthermore, the number of detectable CNVs is decreased. The full potential of the data is not tapped. On the contrary, lower thresholds lead to a major increase in false positive calls.

Table S18: Performance of CopyDetective (raw, i.e. without optional final filtration, and filter, i.e. with default filtration threshold of 10.76) considering automatically calculated thresholds (original) and four additional cases: 1) all thresholds for minimum CNV length are doubled (doubled window), 2) all thresholds for minimum cell fraction are doubled (doubled CF), 3) all thresholds for minimum CNV length are halved (halved window), 4) all thresholds for minimum cell fraction are halved (halved CF). True positive (TP) calls (in brackets: reporting the number of additional true positive calls if CNV type is not evaluated), false positive (FP) calls, found, missed and detectable CNVs, sensitivity (sens; just evaluating true positive calls with correct CNV type), positive predictive value (PPV; just evaluating true positive calls with correct CNV type) and the F1 score.

Config	Thresholds	Data set	TP calls (+ false type)	FP calls	CNVs found	CNVs missed	CNVs detectable	Sens	PPV	F1
raw	original	1	33 (+22)	729	18	1	19	0.95	0.04	0.08
		2	63* (+43)	176	34	3	37	0.92	0.26	0.41
		3	67 (+21)	212	40	1	41	0.98	0.24	0.39
		4	23 (+23)	399	19	0	19	1.00	0.05	0.10
	doubled window	1	32 (+20)	407	18	1	19	0.95	0.07	0.14
		2	60* (+35)	91	29	2	31	0.94	0.40	0.36
		3	62 (+20)	112	37	1	38	0.97	0.36	0.52
		4	23 (+24)	253	16	1	17	0.94	0.08	0.15
	doubled CF	1	20 (+9)	68	14	1	15	0.93	0.23	0.37
		2	35 (+18)	38	23	3	26	0.88	0.48	0.62
		3	52 (+3)	33	36	5	41	0.88	0.61	0.72
		4	0 (+0)	109	0	0	0	/	/	/
	halved window	1	41 (+22)	1195	18	1	19	0.95	0.03	0.06
		2	83 (+49)	256	38	4	42	0.90	0.24	0.62
		3	91 (+28)	344	42	2	44	0.95	0.21	0.34
		4	28 (+32)	604	19	0	19	1.00	0.04	0.08
	halved CF	1	40 (+29)	2906	20	1	21	0.95	0.01	0.03
		2	76* (+49)	301	36	4	40	0.90	0.20	0.33
		3	69 (+33)	596	40	1	41	0.98	0.10	0.19
		4	32 (+22)	841	23	0	23	1.00	0.04	0.07
filter 10.76	original	1	25 (+15)	180	18	1	19	0.95	0.12	0.22
		2	50 (+31)	63	34	3	37	0.92	0.44	0.60
		3	60 (+18)	10	40	1	41	0.98	0.86	0.90
		4	22 (+16)	63	19	0	19	1.00	0.26	0.41
	doubled window	1	30 (+16)	131	18	1	19	0.95	0.19	0.31
		2	54 (+31)	47	29	2	31	0.94	0.53	0.68
		3	58 (+19)	10	35	3	38	0.92	0.85	0.89
		4	23 (+17)	66	16	1	17	0.94	0.26	0.41
	doubled CF	1	15 (+9)	10	13	2	15	0.87	0.60	0.71
		2	30 (+13)	11	23	3	26	0.88	0.73	0.80
		3	47 (+1)	3	35	6	41	0.85	0.94	0.89
		4	0 (+0)	27	0	0	0	/	/	/
	halved window	1	27 (+14)	250	18	1	19	0.95	0.10	0.18
		2	64 (+34)	95	38	4	42	0.90	0.40	0.56
		3	69 (+21)	19	42	2	44	0.95	0.78	0.86
		4	24 (+22)	62	18	1	19	0.95	0.28	0.43
	halved CF	1	31 (+24)	883	20	1	21	0.95	0.03	0.07
		2	58 (+38)	94	36	4	40	0.90	0.38	0.54
		3	61 (+25)	42	40	1	41	0.98	0.59	0.74
		4	27 (+19)	165	21	2	31	0.91	0.14	0.24

*One additional CNV is overlapping a true CNV. However as the called CNV is clearly shorter than the validated one and characterized by a remarkably low quality value, we assume that this overlap is just occurring at coincidence. Therefore, it is counted as ‘missed’.

2.10 Detection of loss of heterozygosity

Loss of heterozygosity (LOH) describes the deletion of the non-mutated allele of a heterozygous variant. The resulting variant is, thus, hemizygous. Subsequently, a duplication event can take place, resulting in a copy-neutral LOH.

Some tools detecting CNVs are able to detect even copy-neutral LOHs, while others are not. As CopyDetective analyzes the change in VAF of heterozygous polymorphisms, the deletion event happening in case of every LOH is detected by CopyDetective. However, as a VAF of 100% can be observed for the hemizygous polymorphisms, any subsequent duplication cannot be detected. Consequently, CopyDetective calls every LOH as a deletion. Whether the actual copy number is 1 – due to the deletion – or 2 – due to subsequent duplication – cannot be determined from the change in VAF. Therefore, we added an indicator, evaluating coverage: for every polymorphism it is evaluated, if the coverage is above or below the average coverage over all polymorphisms in that sample. This is done for both the case and the control sample. Subsequently, it is tested for the regions of significant difference whether the number of polymorphisms with coverage below the average is higher or lower compared to the matching control sample (binomial test). The estimate and the 95%-confidence interval are reported. A positive estimate indicates an increase in coverage, while a negative estimate indicates a decrease. Comparing deletions to LOH, we observe the results summed up in Figure S15.

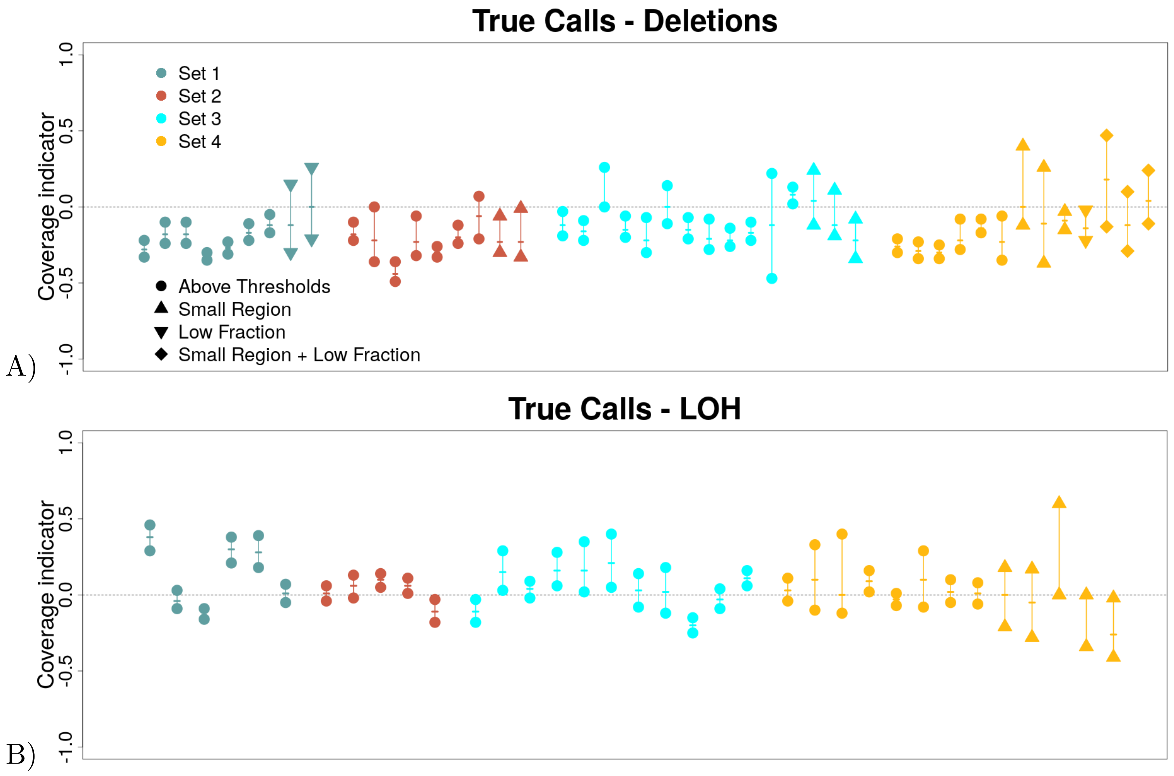

Figure S15: Coverage indicator for true calls detected in our data sets. A) Deletions. B) LOH (for data set 4, CF was not evaluated as it was not available from validation data).

It can be observed that true deletions are characterized by a negative coverage indicator. For true CNVs above the thresholds, even the confidence intervals are ≤ 0 in 28 out of 32 cases. In contrary, true LOH that are also reported as 'deletions' by CopyDetective are mostly characterized by a positive coverage indicator, or a coverage indicator overlapping zero (26 out of 30 cases).

3 Availability of CopyDetective

CopyDetective is freely available at https://github.com/sandmanns/CopyDetective. We used R 3.6.0 [18] for programming. A graphical user interface (using R shiny) is available. A screenshot is displayed in Figure S16.

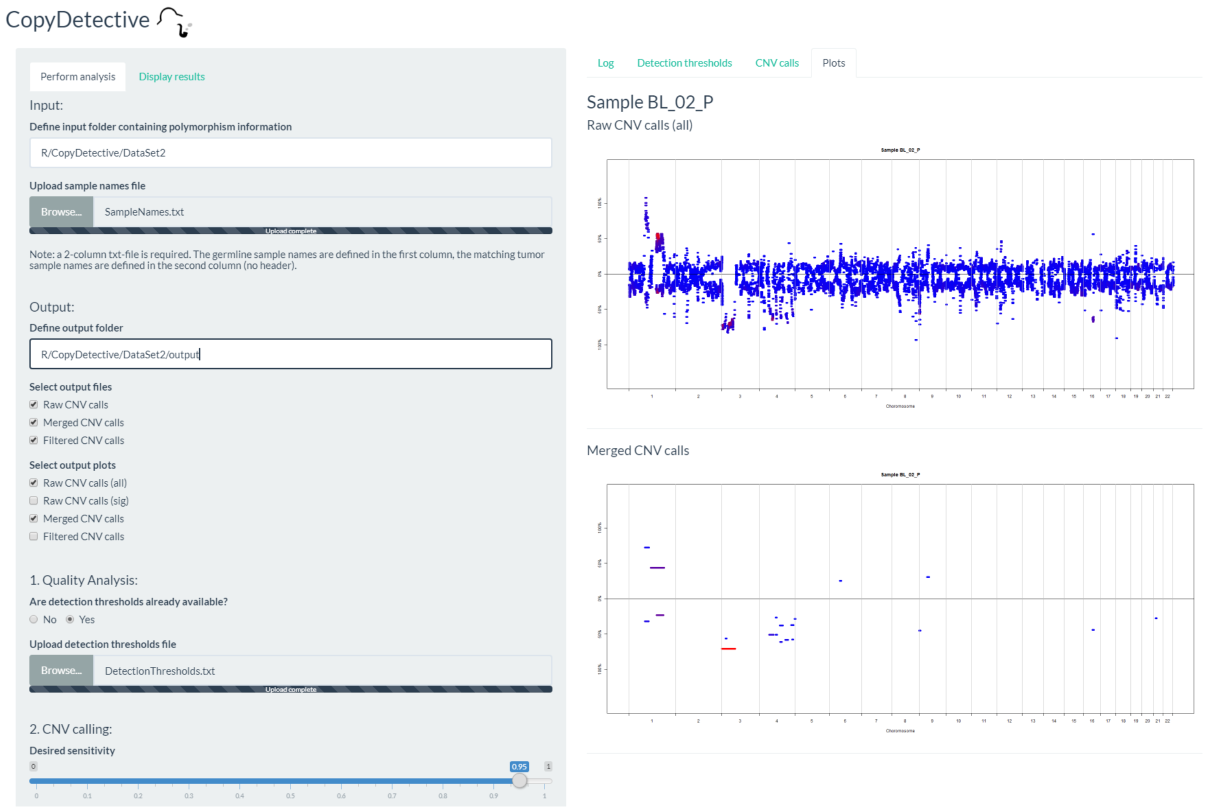

Figure S16: Screenshot of CopyDetective.

An input folder containing information on the heterozygous polymorphisms called for every sample has to be provided. Furthermore, a sample file has to be uploaded. Output options include the output folder and a selection of output files to be generated.

First, quality analysis is performed. If detection thresholds are already available, these can be uploaded. CopyDetective will skip the quality step and directly start with step 2) CNV calling. If no detection thresholds are available, CopyDetective will estimate them. Two options are available: simulation and exact. If simulation is chosen, the number of simulations (default: 500) can be defined. If exact is chosen, the method for the VAF of polymorphisms in the control sample can be selected: exact (variant calling results) or simulated (expected value 0.5). The following additional parameters can be defined: the cell fractions to consider (default: 5 to 100% in steps of 5%; values between 1 and 100% possible, step sizes between 1 and 100% possible), the percentile for window selection (default: 95%), and the strategy for detection threshold optimization (default: compromise; alternative: force (frequency) or force (window size)).

As a second step, CNV calling is performed. Raw CNV calls are merged. The maximum distance separating two raw calls can be defined (default: 20 Mbp). Optionally, filtration can be performed, excluding low-quality CNV calls (default threshold: 10.76).

CopyDetective generates various output. The detection thresholds are displayed for all analyzed patients. Furthermore, the CNV calls and plots generated for the last analyzed sample are displayed. However, the tab ‘Display results’ allows the user to select the output of any analyzed patient for display. Additionally, all output files are saved in the defined output folder.

References

[1] Lai Z, Markovets A, Ahdesmaki M, Chapman B, Hofmann O, McEwen R, et al. VarDict: A novel and versatile variant caller for next-generation sequencing in cancer research. Nucleic Acids Res. 2016;1:e108.

[2] Sandmann S, de Graaf AO, Karimi M, van der Reijden BA, Hellström-Lindberg E, Jansen JH, et al. Evaluating variant calling tools for non-matched next-generation sequencing data. Sci Rep. 2017;7:43169.

[3] Consortium TGP. A global reference for human genetic variation. Nature. 2015;526:68–74.

[4] Sherry ST, Ward MH, Kholodov M, Baker J, Phan L, Smigielski EM, et al. DbSNP: the NCBI database of genetic variation. Nucleic Acids Res. 2001;29:308–311.

[5] Lek M, Karczewski KJ, Minikel EV, Samocha KE, Banks E, Fennell T, et al. Analysis of protein-coding genetic variation in 60,706 humans. Nature. 2016;536:285–291.

[6] Landrum MJ, M L, Benson M, Brown G, Chao C, Chitipiralla S, et al. ClinVar: public archive of interpretations of clinically relevant variants. Nucleic Acids Res. 2016;44:D862–D868.

[7] Sathirapongsasuti JF, Lee H, Horst BA, Brunner G, Cochran AJ, Binder S, et al. Exome sequencing-based copy-number variation and loss of heterozygosity detection: ExomeCNV. Bioinformatics. 2011;27:2648–2654.

[8] Koboldt DC, Zhang Q, Larson DE, Shen D, McLellan MD, Lin L, et al. VarScan 2: Somatic mutation and copy number alteration discovery in cancer by exome sequencing. Gen Res. 2012;22:568–576.

[9] Boeva V, Popova T, Bleakley K, Chiche P, Cappo J, Schleiermacher G, et al. Control-FREEC: a tool for assessing copy number and allelic content using next-generation sequencing data. Bioinformatics. 2012;28:423–425.

[10] Zhang Z, Hao K. SAAS-CNV: A Joint Segmentation Approach on Aggregated and Allele Specific Signals for the Identification of Somatic Copy Number Alterations with Next-Generation Sequencing Data. PLoS Comput Biol. 2015;11:e1004618.

[11] Favero F, Joshi T, Marquard AM, Birkbak NJ, Krzystanek M, Li Q, et al. Sequenza: allele-specific copy number and mutation profiles from tumor sequencing data. Ann Oncol. 2015;26:64–70.

[12] Klambauer G, Schwarzbauer K, Mayr A, Clevert DA, Mitterecker A, Bodenhofer U, et al. cn.MOPS: mixture of Poissons for discovering copy number variations in next generation sequencing data with a low false discovery rate. Nucleic Acids Res. 2012;40:e69.

[13] Packer JS, Maxwell EK, O'Dushlaine C, Lopez AE, Dewey FE, Chernomorsky R, et al. CLAMMS: a scalable algorithm for calling common and rare copy number variants from exome sequencing data. Bioinformatics. 2016;32:133–135.

[14] Zhou Z, Wang W, Wang LS, Zhang NR. Integrative DNA copy number detection and genotyping from sequencing and array-based platforms. Bioinformatics. 2018;34:2349–2355.

[15] Sandmann S, Khanam T, Reutter K, Burkhardt B, Dugas M. A Novel Algorithm for CNV Calling in Matched WES Data. F1000Research. 2018;7:1532 (poster).

[16] Oesper L, Mahmoody A, Raphael BJ. Quantifying Tumor Heterogeneity in Whole-Genome and Whole-Exome Sequencing Data. Bioinformatics. 2014;30:3532–3540.

[17] Shen W, Paxton CN, Szankasi P, Longhurst M, Schumacher JA, Frizzell KA, et al. Detection of genome-wide copy number variants in myeloid malignancies using next-generation sequencing. J Clin Pathol. 2018;71:372–378.

[18] Team RC. R: A Language and Environment for Statistical Computing. R Foundation for Statistical Computing Vienna, Austria, http://wwwR-projectorg/. 2013;.
