# Supplementary figures and images for: CopyDetective: Detection threshold–aware copy number variant calling in whole-exome sequencing data
Source: Gigascience. 2020 Nov 2;9(11):giaa118. doi: 10.1093/gigascience/giaa118 (PMC7604644; doi:10.1093/gigascience/giaa118)

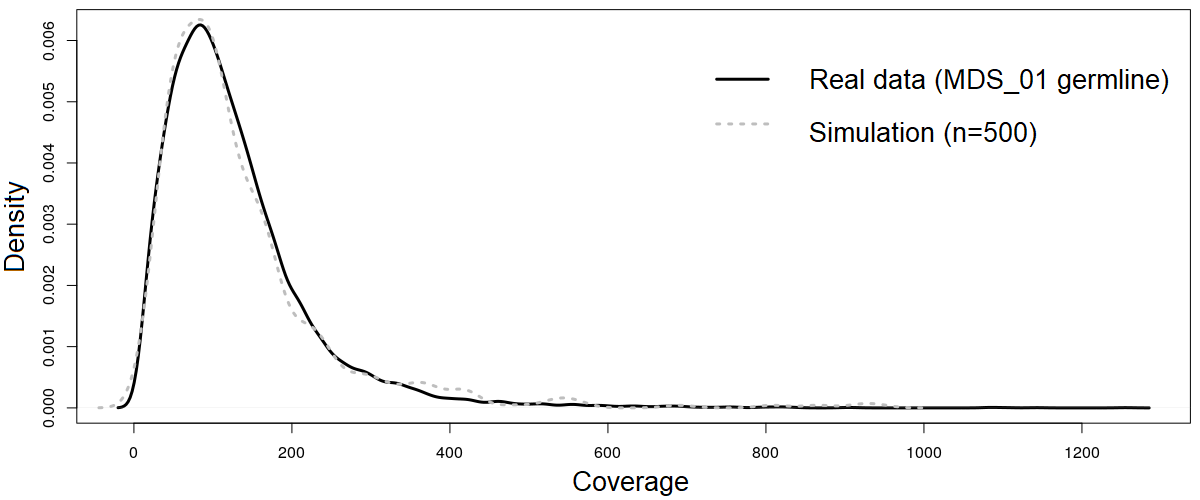

Supplement: giaa118_Supplemental_Files [file giaa118_supplemental_files.zip › FigS1.png]

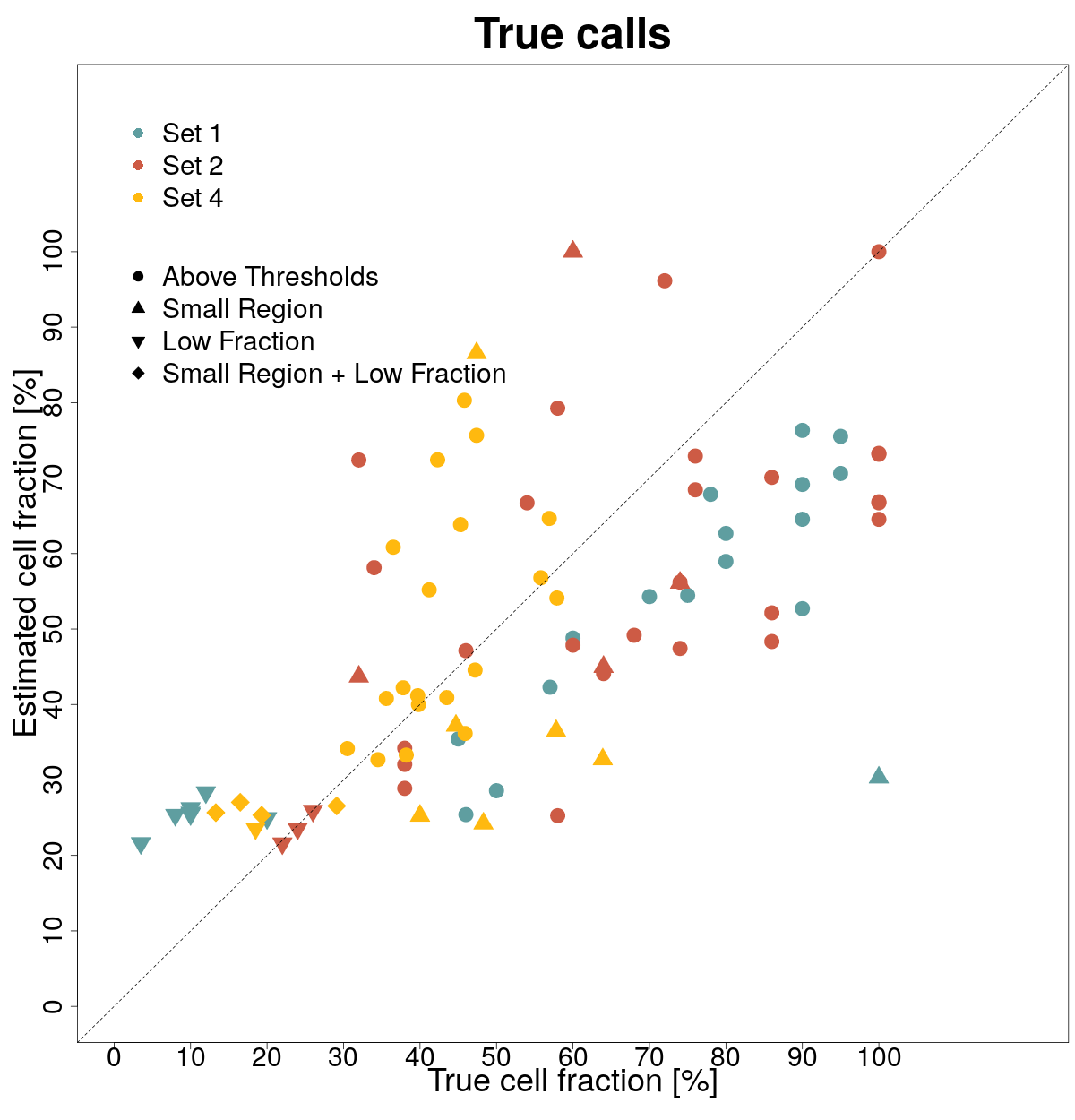

Supplement: giaa118_Supplemental_Files [file giaa118_supplemental_files.zip › FigS10A.png]

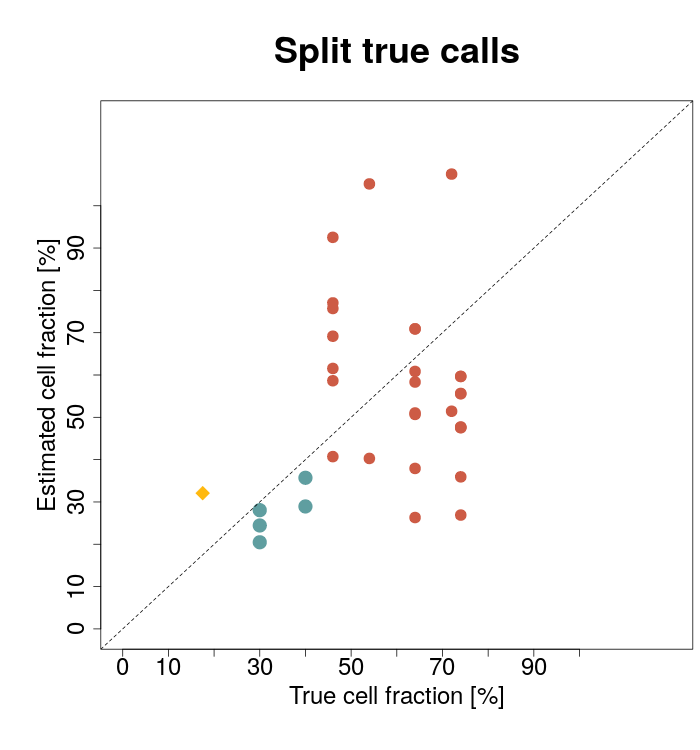

Supplement: giaa118_Supplemental_Files [file giaa118_supplemental_files.zip › FigS10B.png]

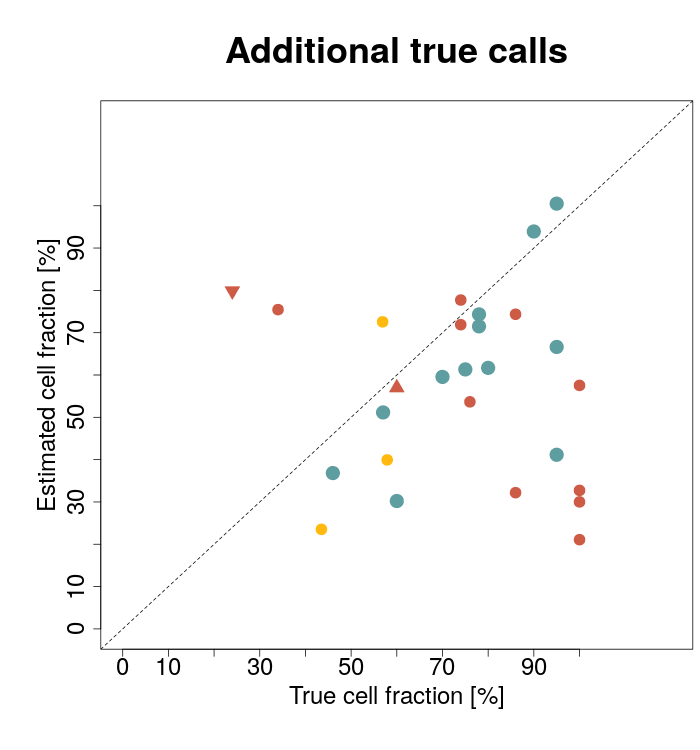

Supplement: giaa118_Supplemental_Files [file giaa118_supplemental_files.zip › FigS10C.png]

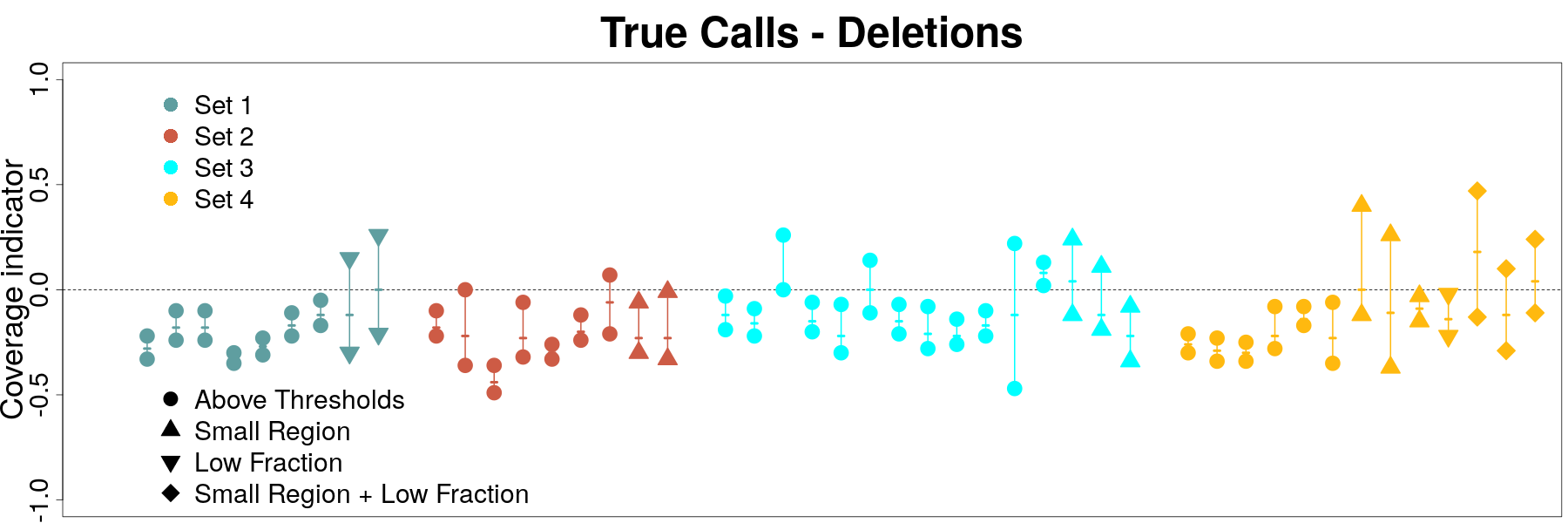

Supplement: giaa118_Supplemental_Files [file giaa118_supplemental_files.zip › FigS11A.png]

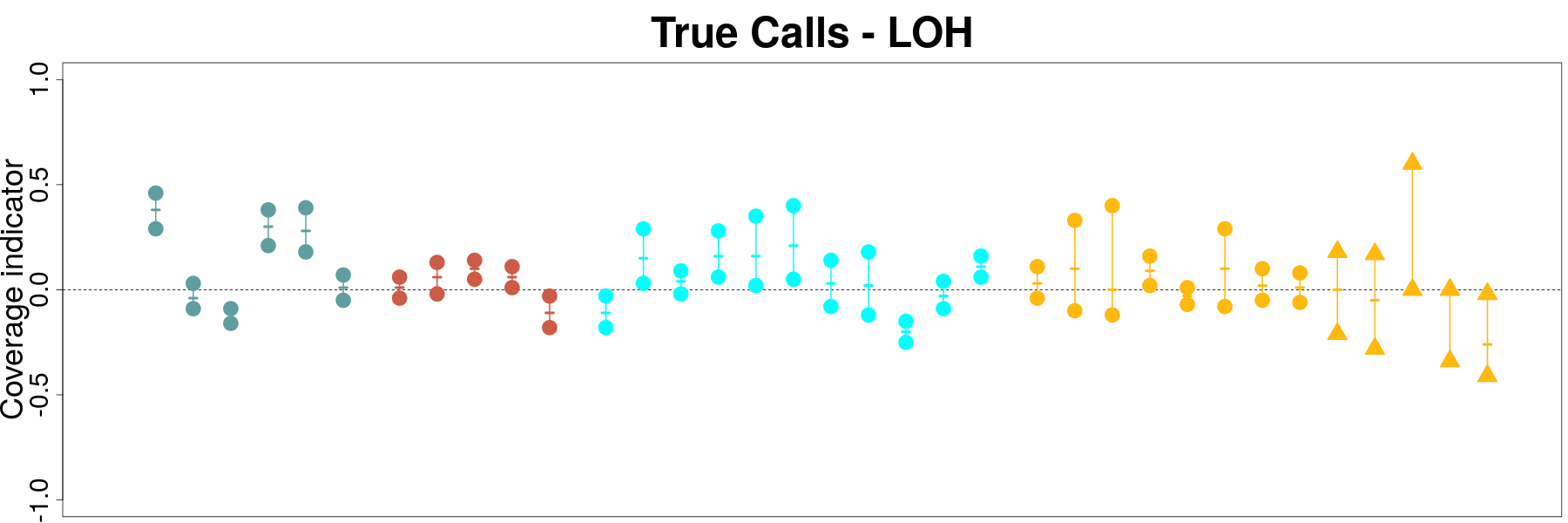

Supplement: giaa118_Supplemental_Files [file giaa118_supplemental_files.zip › FigS11B.png]

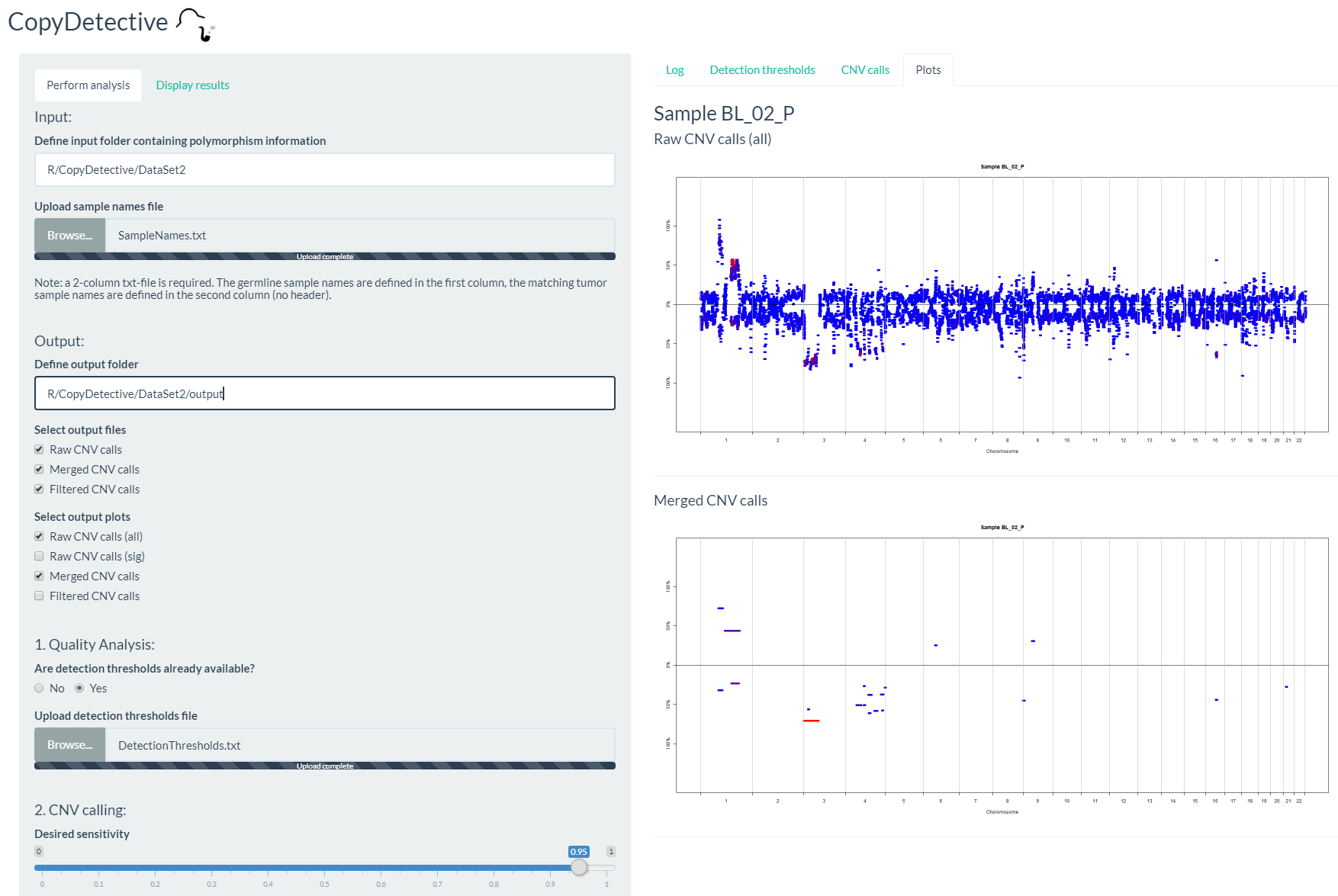

Supplement: giaa118_Supplemental_Files [file giaa118_supplemental_files.zip › FigS12.png]

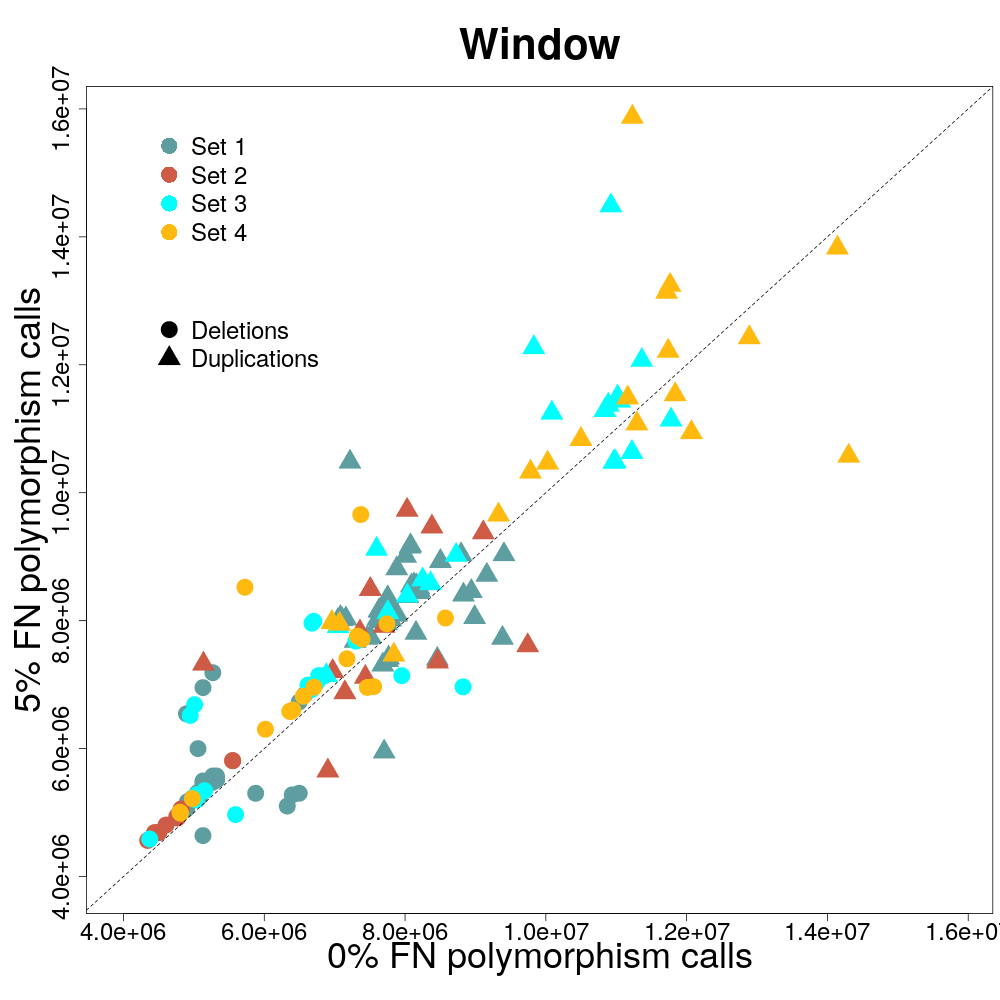

Supplement: giaa118_Supplemental_Files [file giaa118_supplemental_files.zip › FigS12a.png]

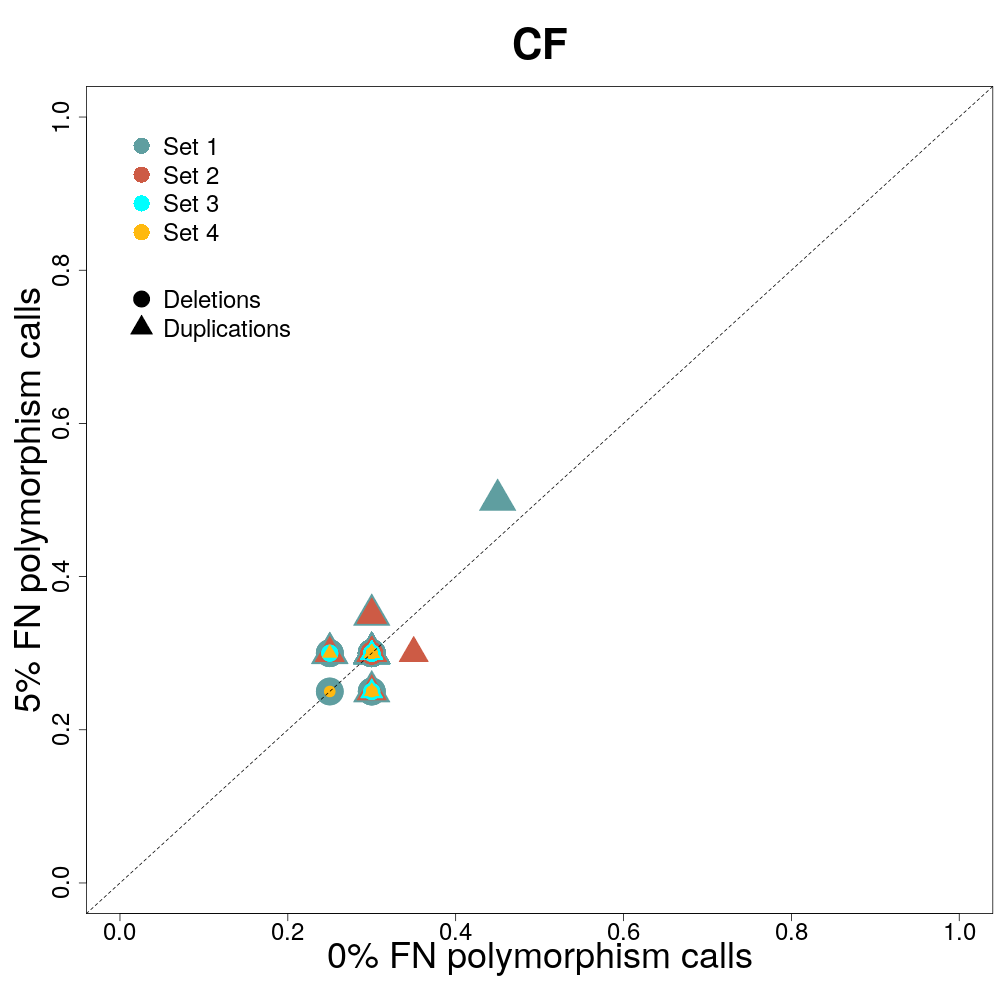

Supplement: giaa118_Supplemental_Files [file giaa118_supplemental_files.zip › FigS12b.png]

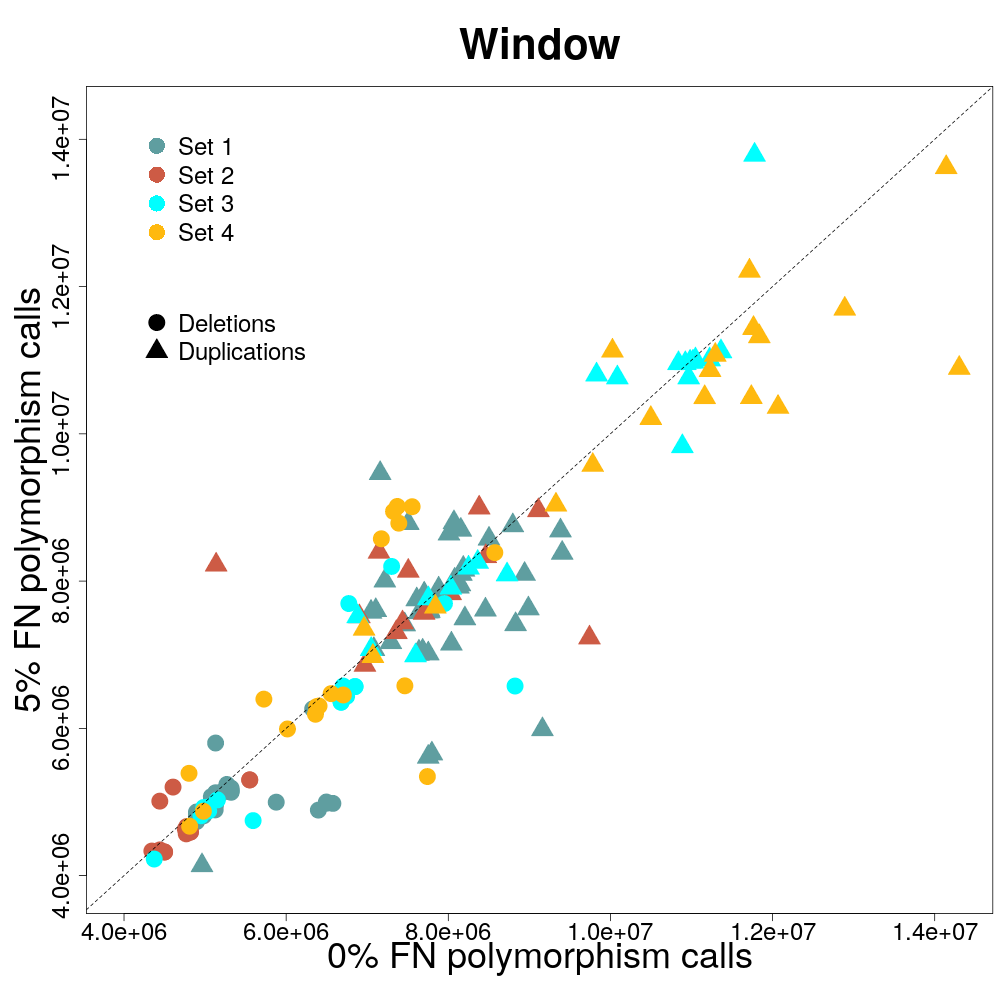

Supplement: giaa118_Supplemental_Files [file giaa118_supplemental_files.zip › FigS13a.png]

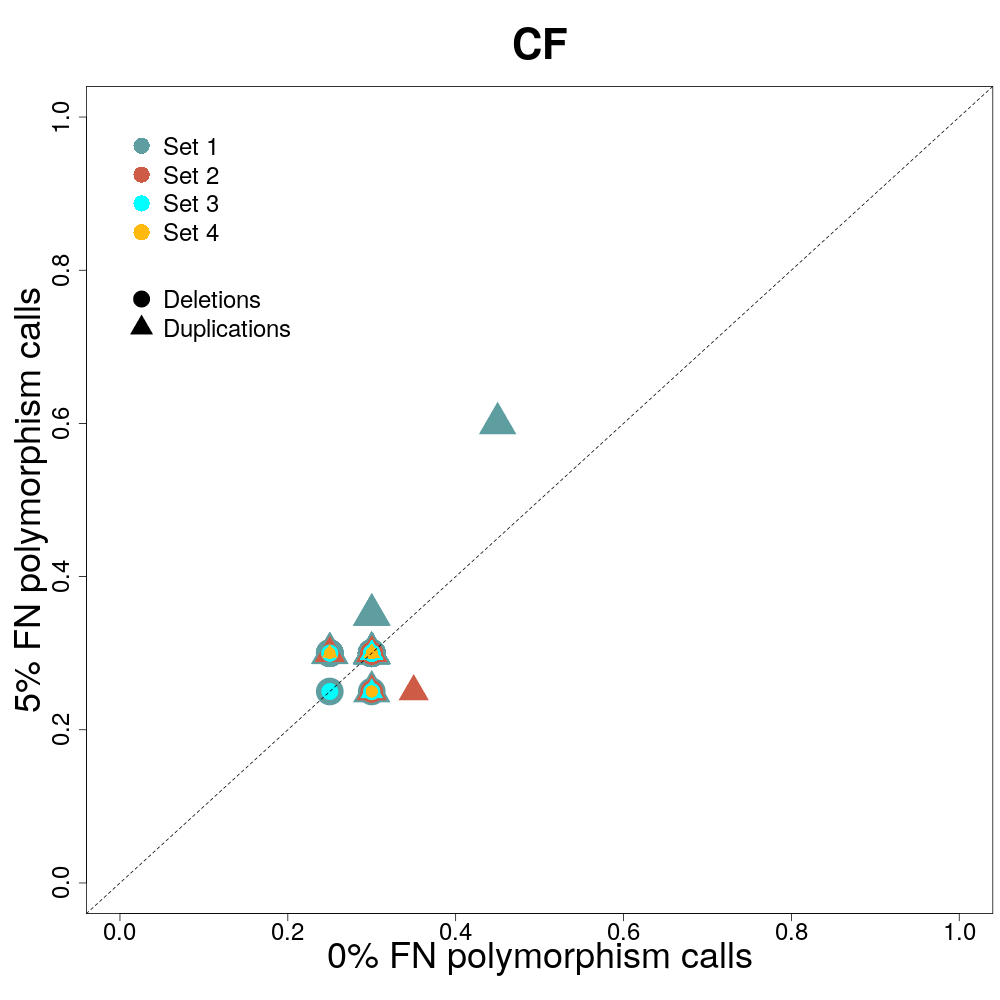

Supplement: giaa118_Supplemental_Files [file giaa118_supplemental_files.zip › FigS13b.png]

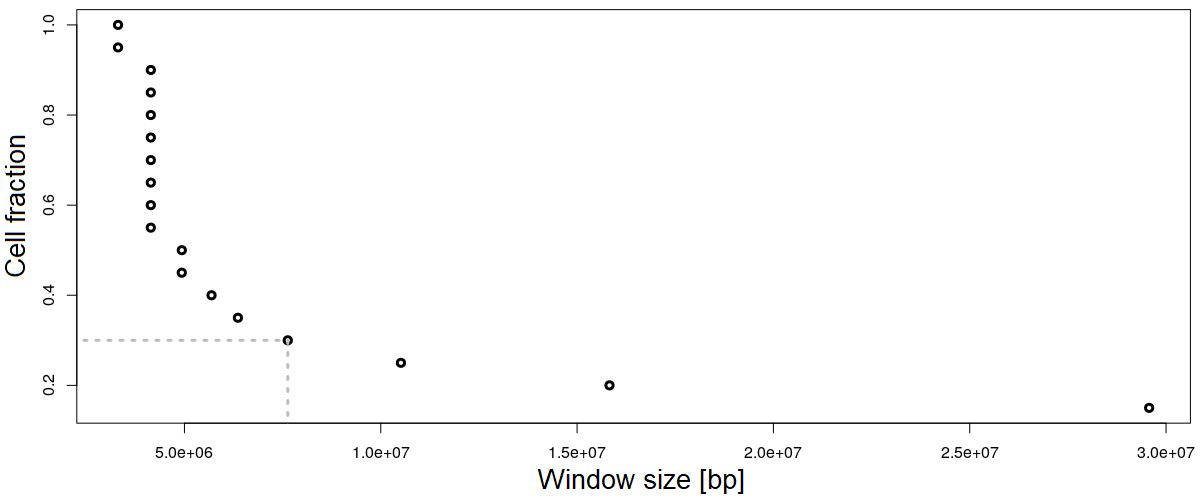

Supplement: giaa118_Supplemental_Files [file giaa118_supplemental_files.zip › FigS2.png]

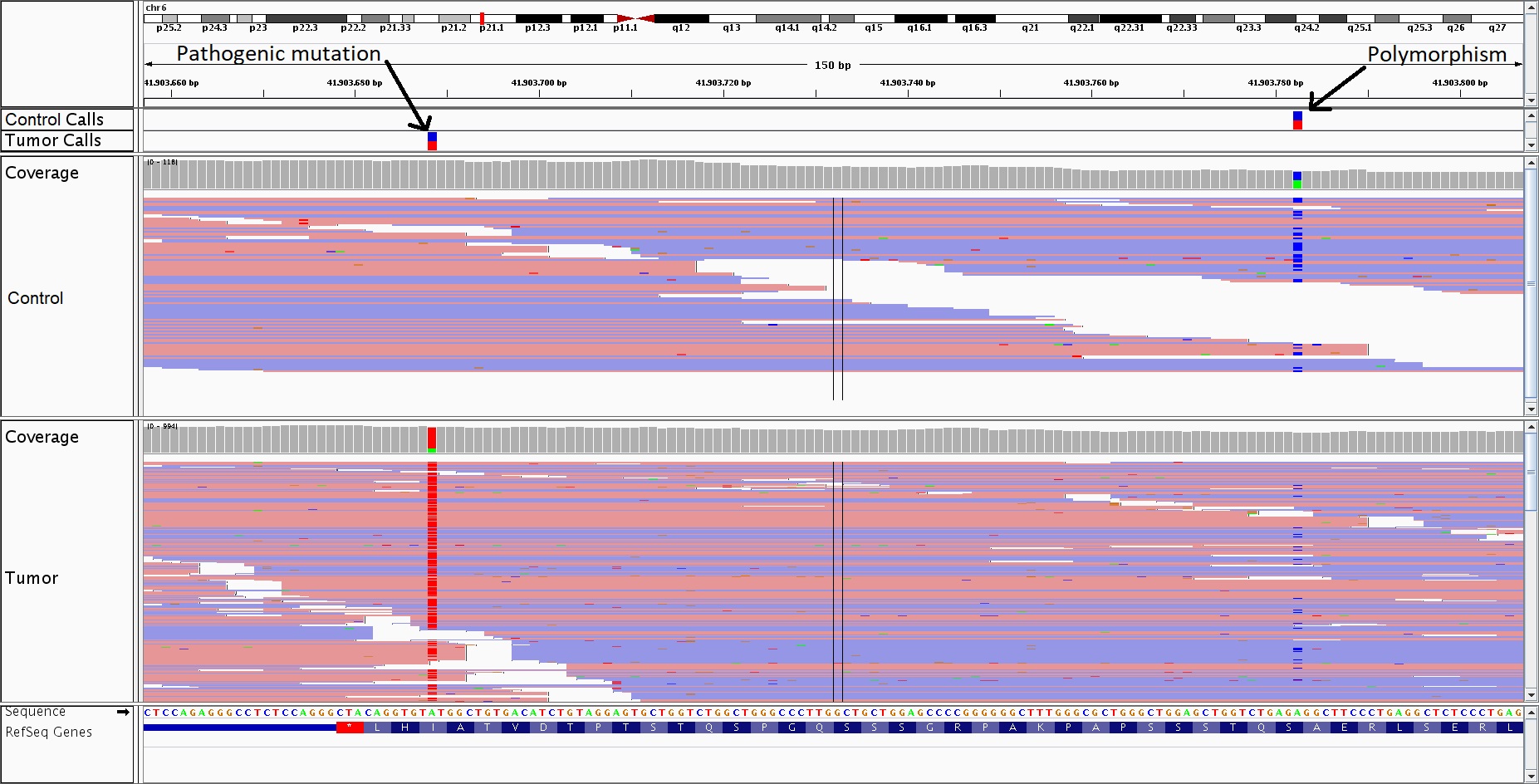

Supplement: giaa118_Supplemental_Files [file giaa118_supplemental_files.zip › FigS3.jpg]

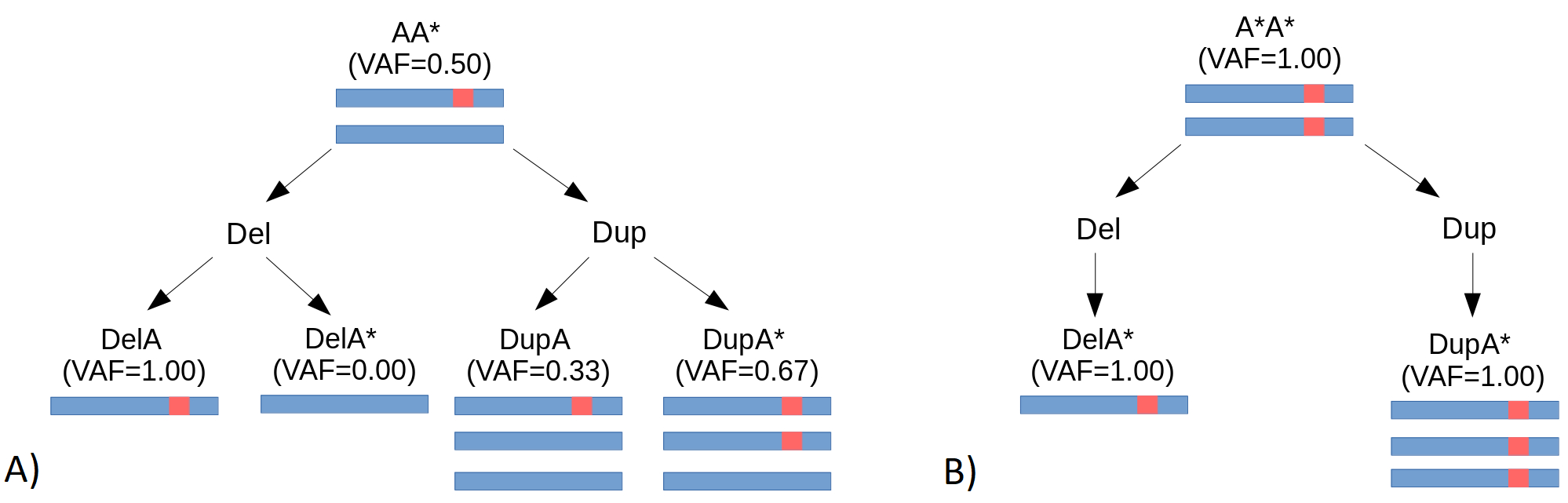

Supplement: giaa118_Supplemental_Files [file giaa118_supplemental_files.zip › FigS4.jpg]

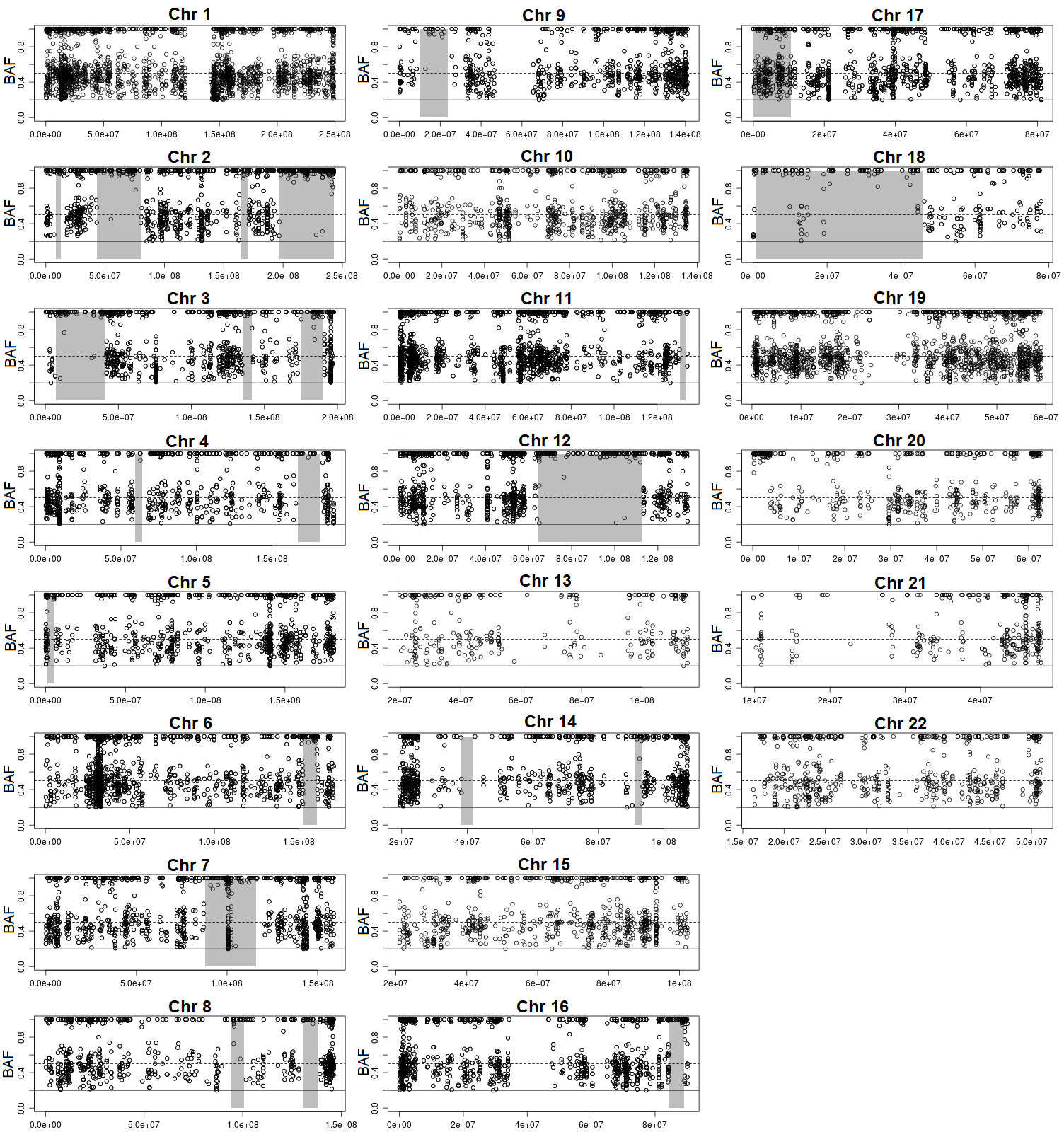

Supplement: giaa118_Supplemental_Files [file giaa118_supplemental_files.zip › FigS5.png]

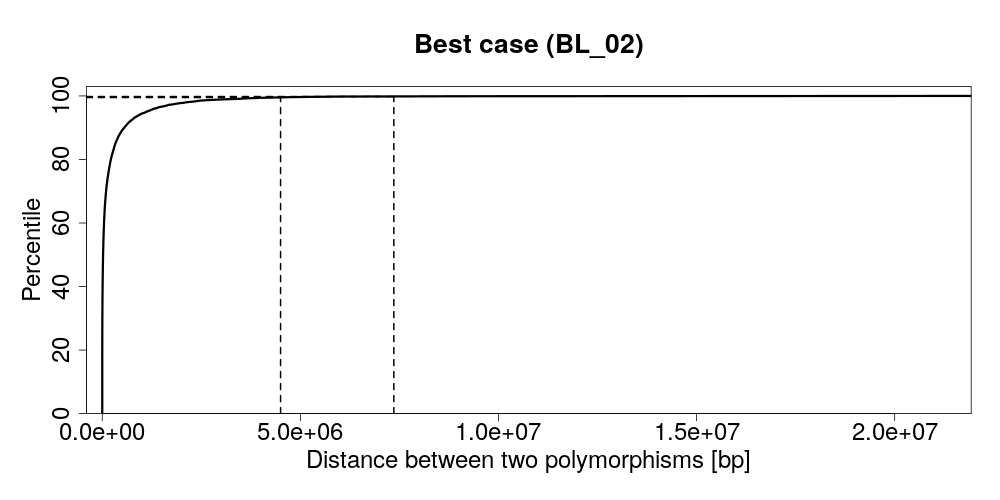

Supplement: giaa118_Supplemental_Files [file giaa118_supplemental_files.zip › FigS5_2a.png]

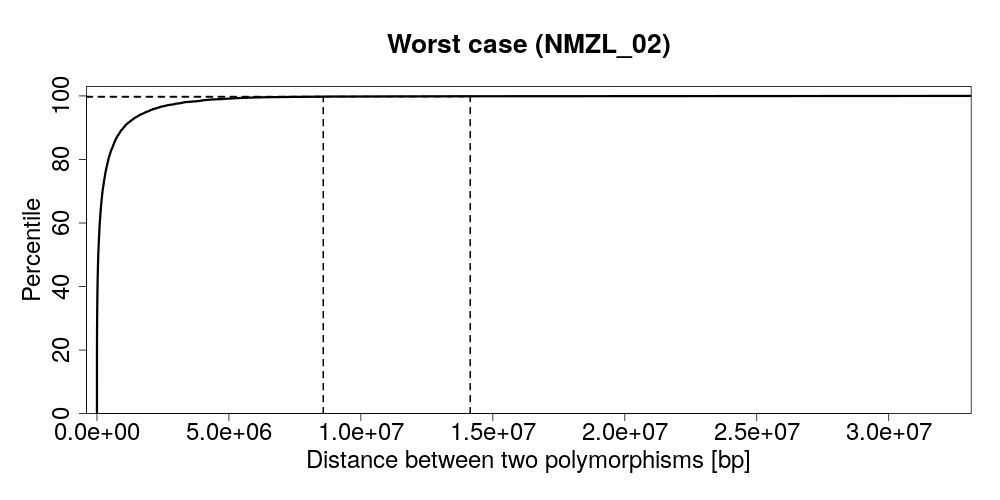

Supplement: giaa118_Supplemental_Files [file giaa118_supplemental_files.zip › FigS5_2b.png]

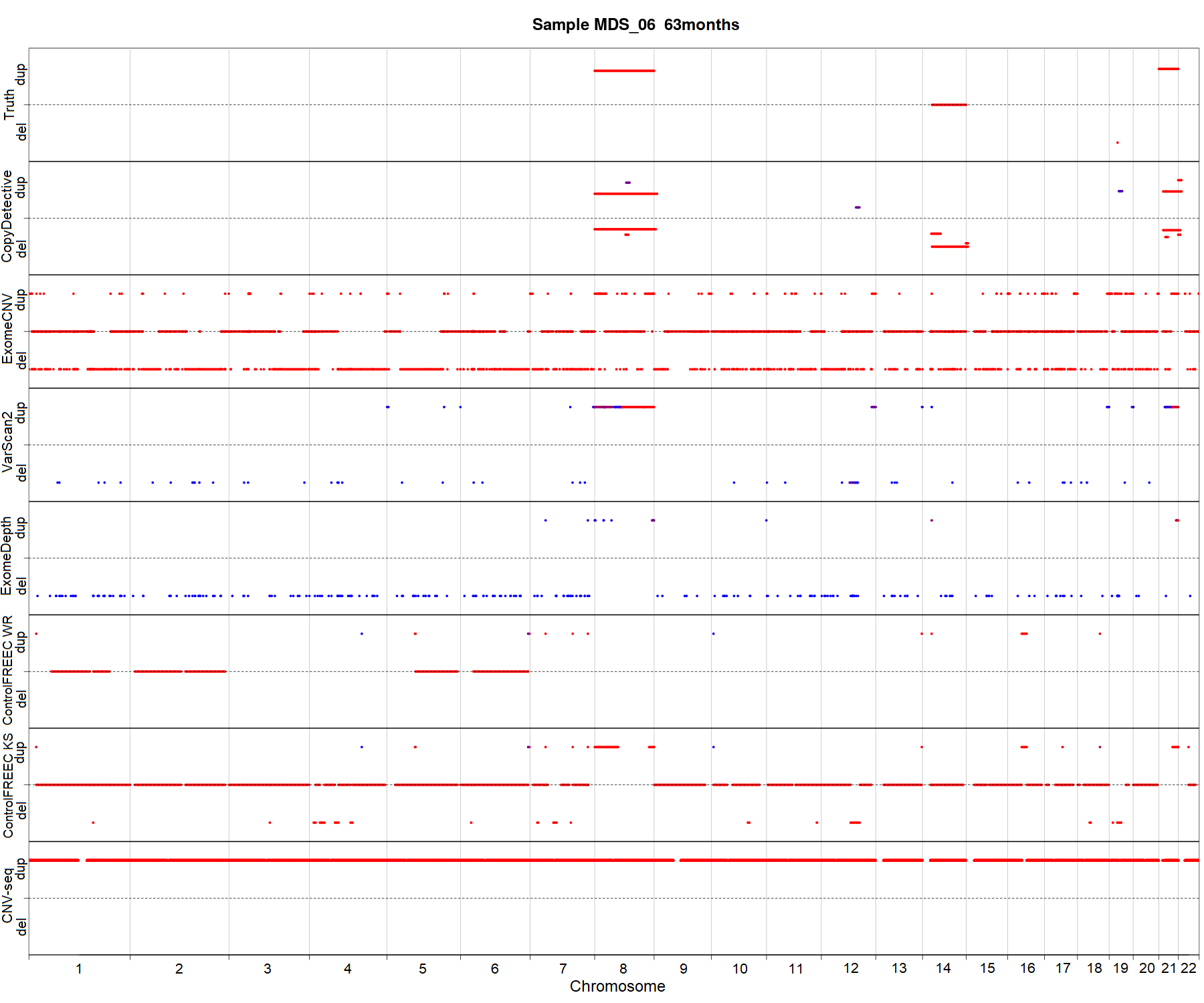

Supplement: giaa118_Supplemental_Files [file giaa118_supplemental_files.zip › FigS6.png]

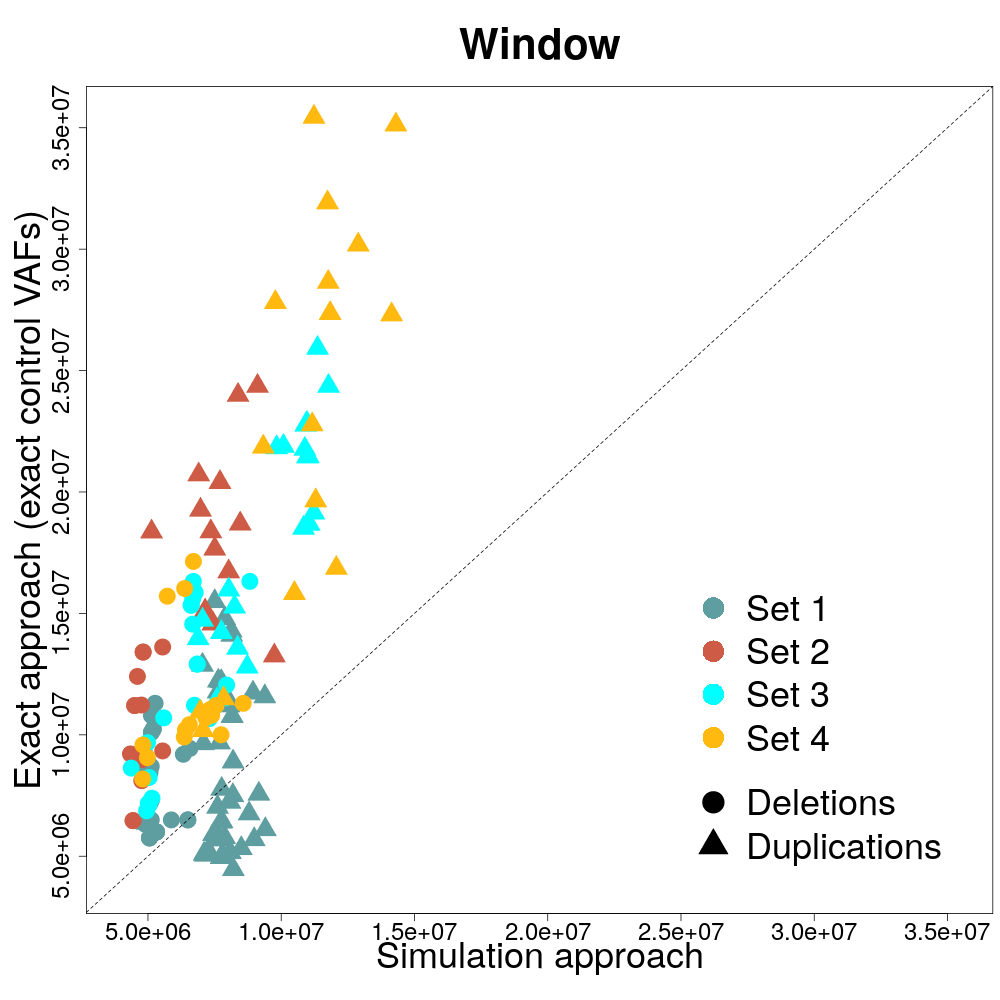

Supplement: giaa118_Supplemental_Files [file giaa118_supplemental_files.zip › FigS6_2a.png]

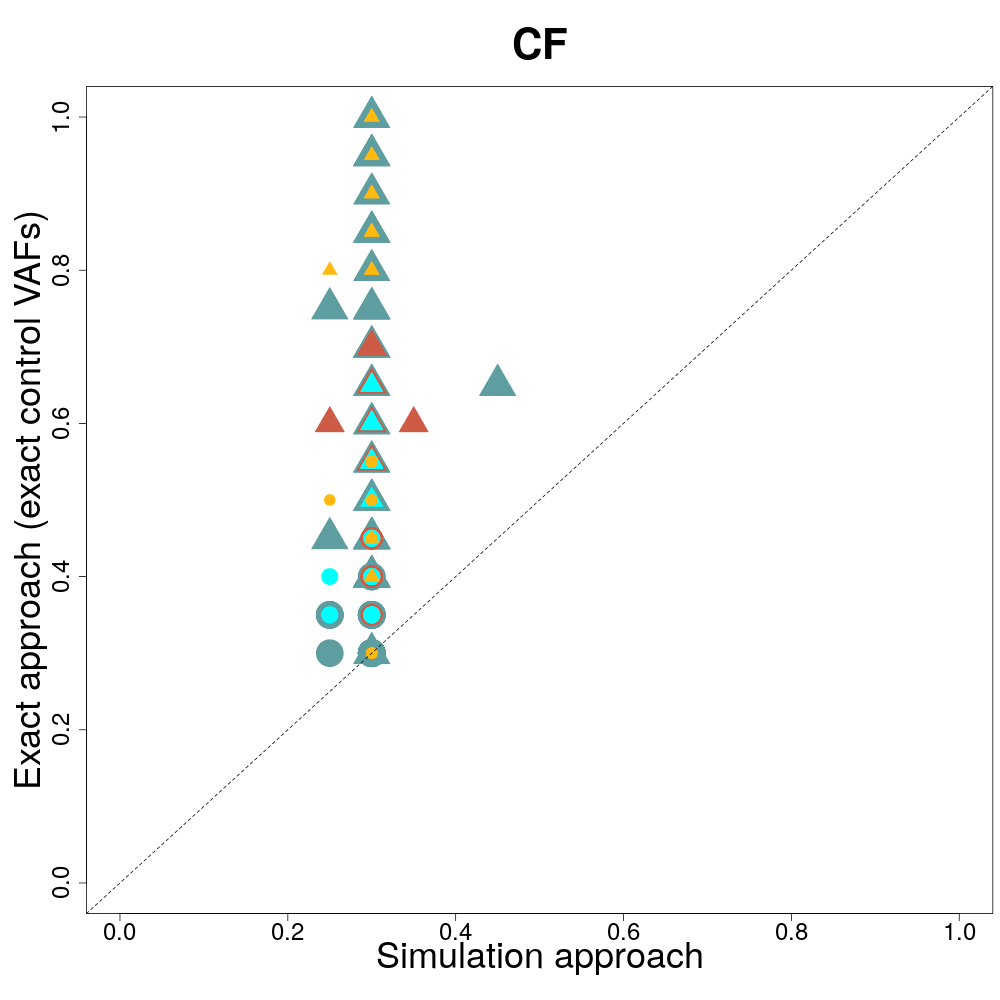

Supplement: giaa118_Supplemental_Files [file giaa118_supplemental_files.zip › FigS6_2b.png]

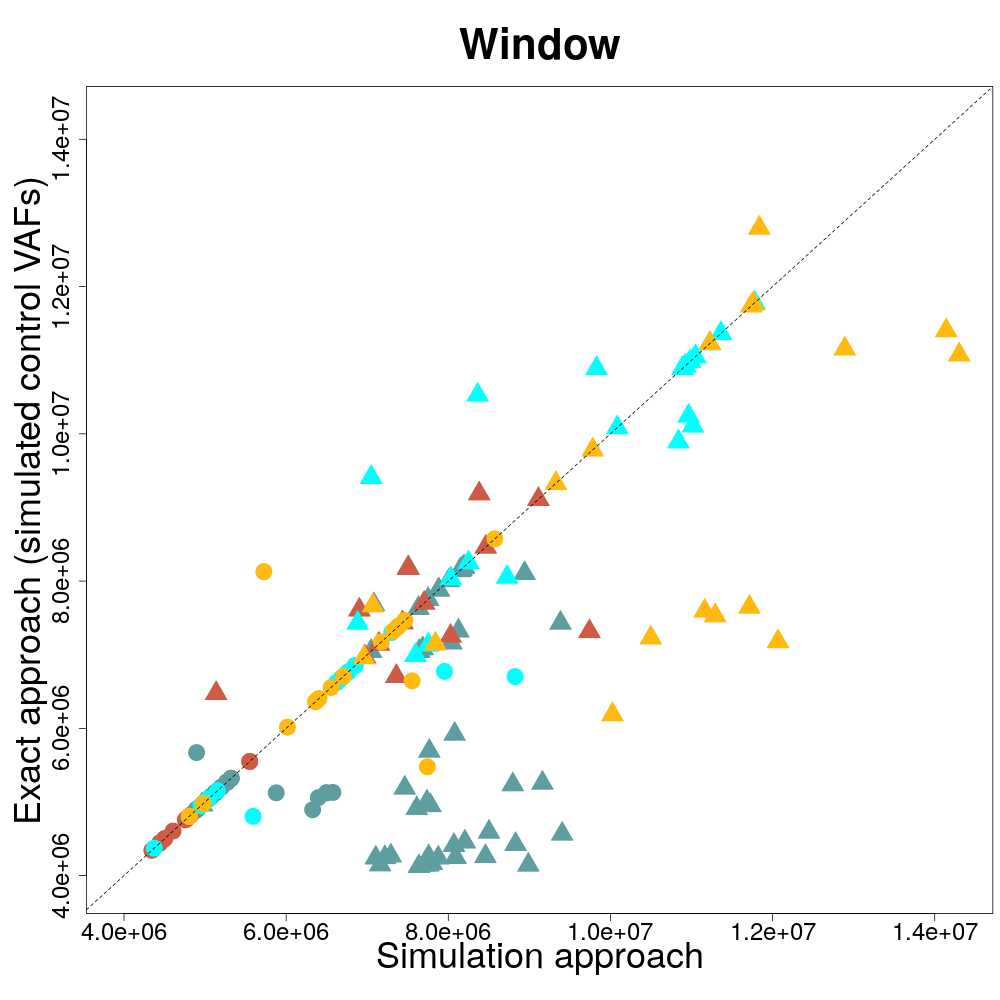

Supplement: giaa118_Supplemental_Files [file giaa118_supplemental_files.zip › FigS6_2c.png]

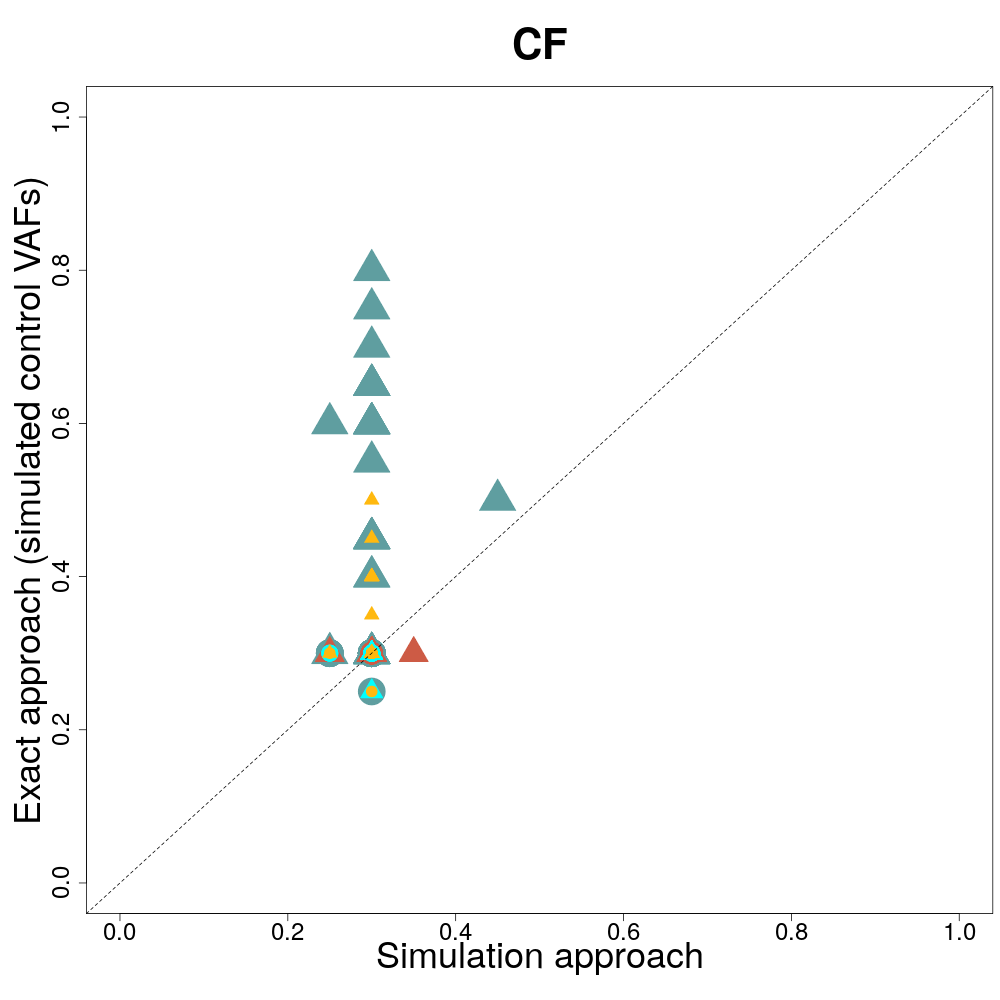

Supplement: giaa118_Supplemental_Files [file giaa118_supplemental_files.zip › FigS6_2d.png]

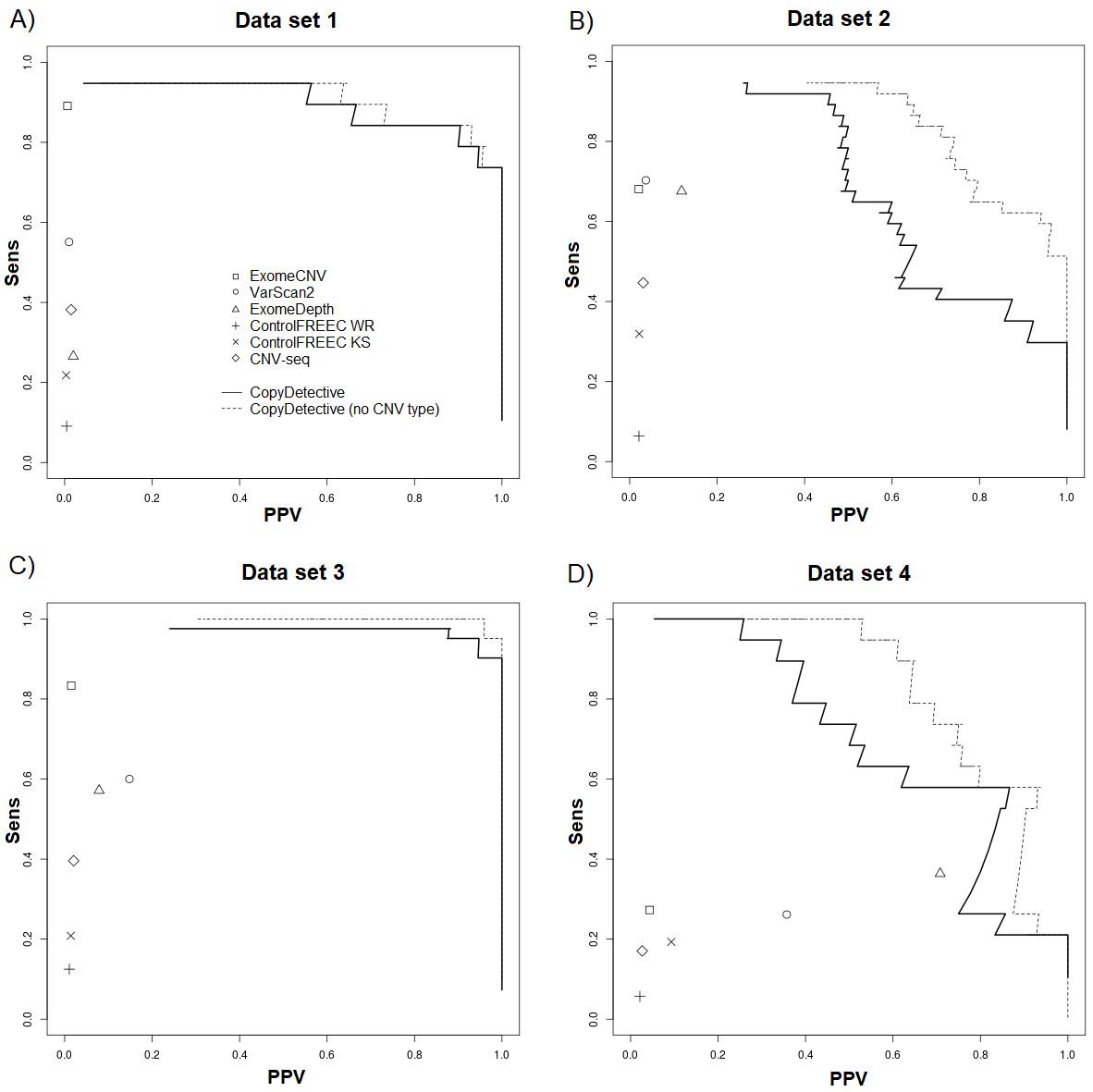

Supplement: giaa118_Supplemental_Files [file giaa118_supplemental_files.zip › FigS7.png]

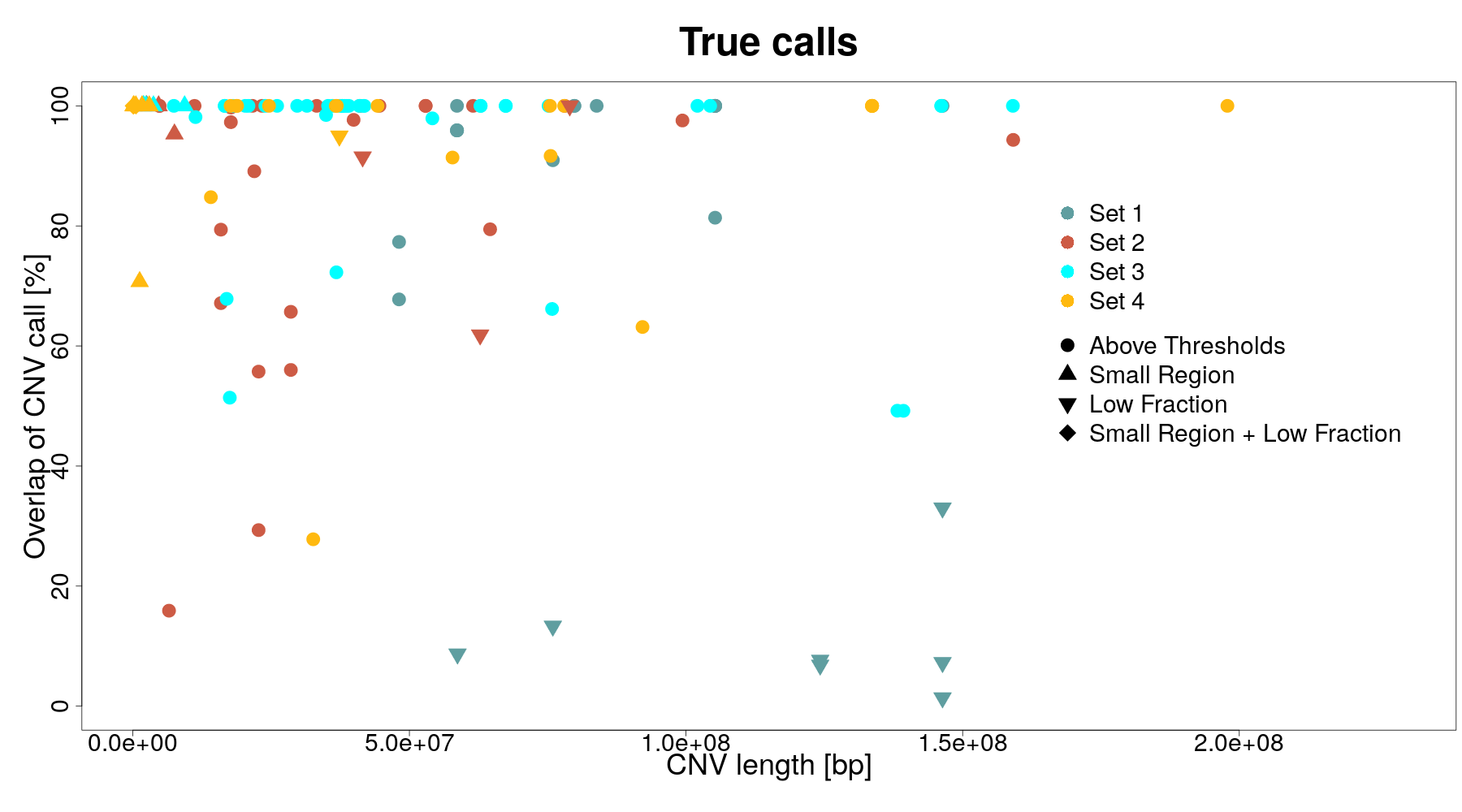

Supplement: giaa118_Supplemental_Files [file giaa118_supplemental_files.zip › FigS8A.png]

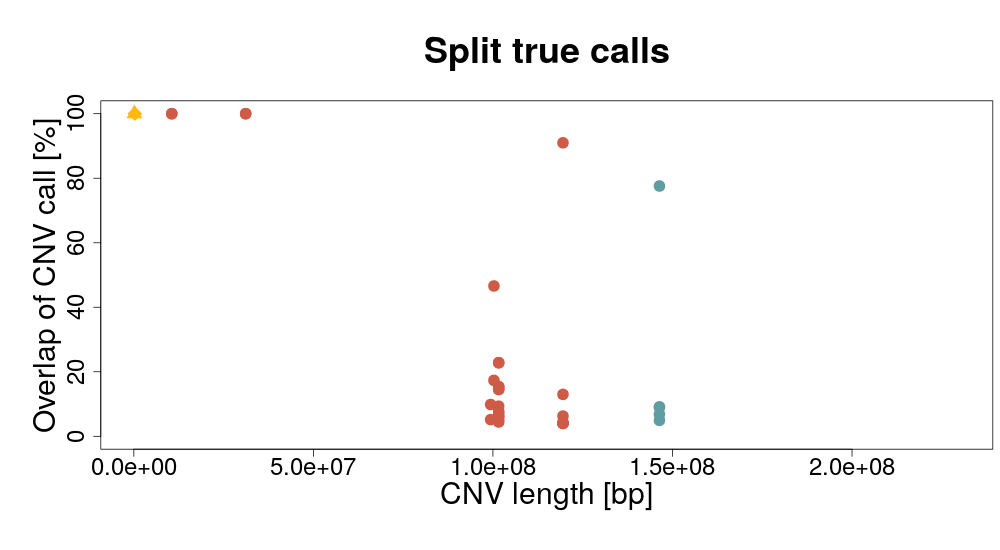

Supplement: giaa118_Supplemental_Files [file giaa118_supplemental_files.zip › FigS8B.png]

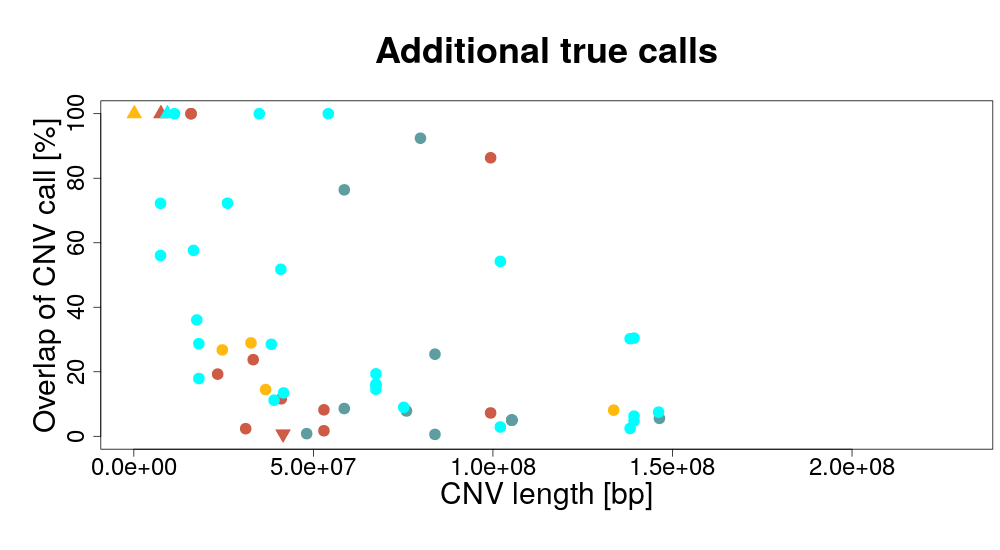

Supplement: giaa118_Supplemental_Files [file giaa118_supplemental_files.zip › FigS8C.png]

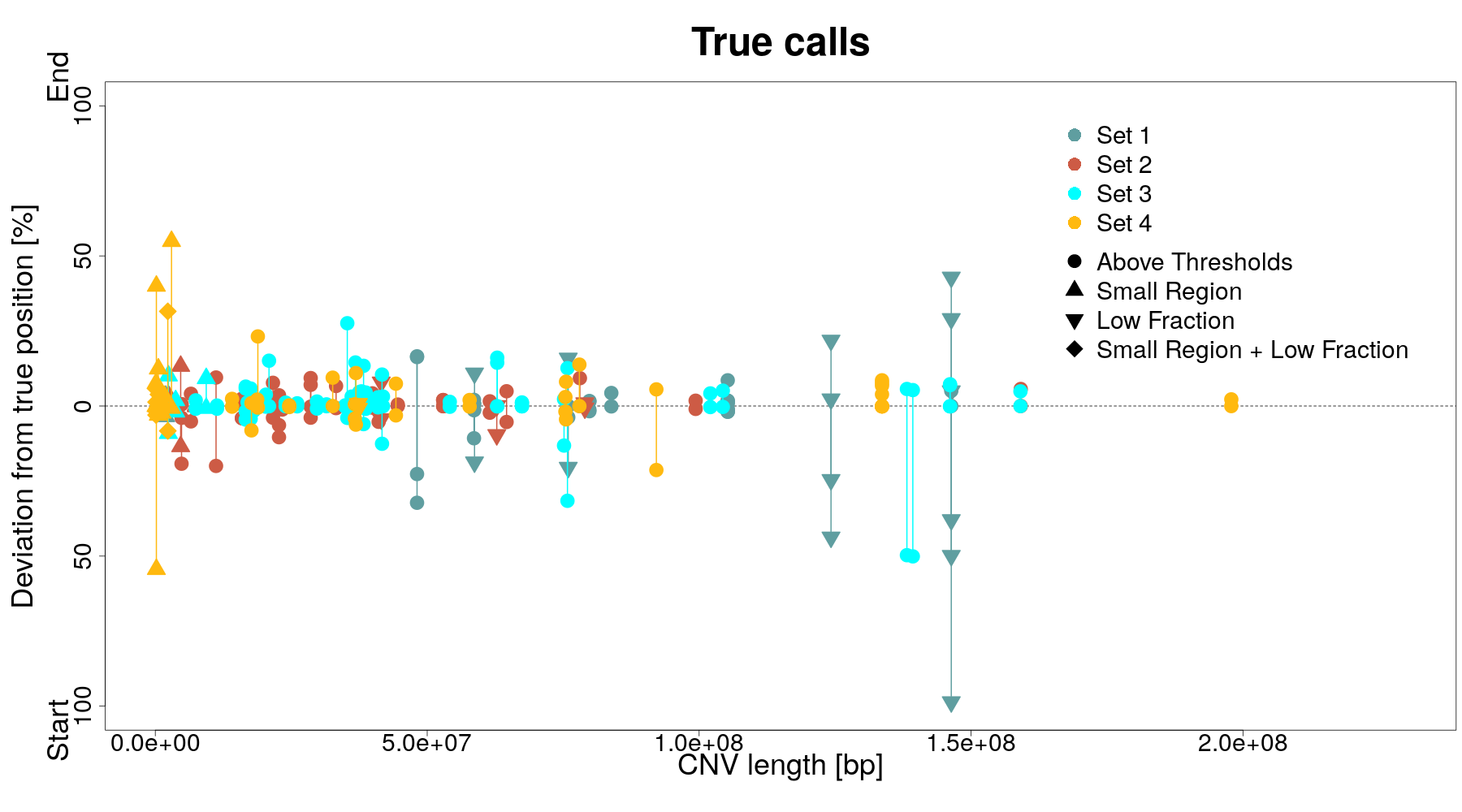

Supplement: giaa118_Supplemental_Files [file giaa118_supplemental_files.zip › FigS9A.png]

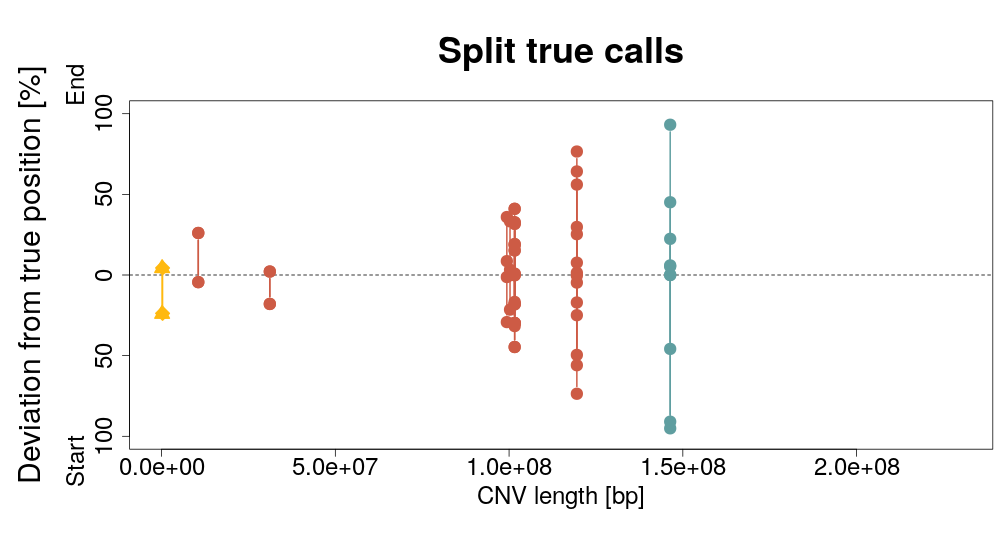

Supplement: giaa118_Supplemental_Files [file giaa118_supplemental_files.zip › FigS9B.png]

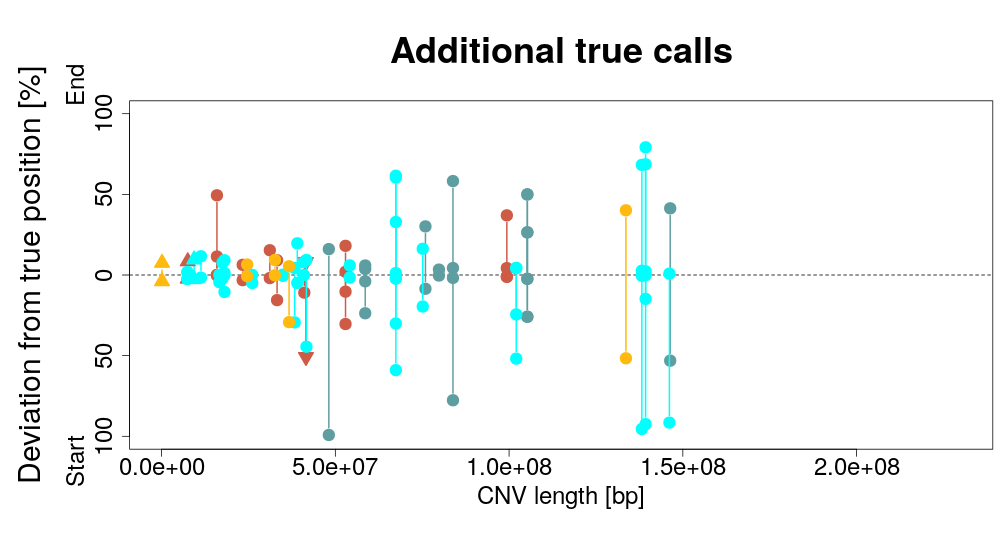

Supplement: giaa118_Supplemental_Files [file giaa118_supplemental_files.zip › FigS9C.png]
