# Supplementary material for: CopyDetective: Detection threshold–aware copy number variant calling in whole-exome sequencing data
Source: Gigascience. 2020 Nov 2;9(11):giaa118. doi: 10.1093/gigascience/giaa118 (PMC7604644; doi:10.1093/gigascience/giaa118)
Supplement: giaa118_Response_to_Reviewer_Comments_Original_Submission [file giaa118_response_to_reviewer_comments_original_submission.pdf]

Author's Response To Reviewer Comments

Close

Reviewer: 1

Major comments:
1. My main concern is that calling CNV based on allelic frequency information can impose some limitations. First, the method would be dependent on the accuracy of the variant calling methods. How robust is the proposed method against variant calling errors in calling SNPs? The accuracy of the polymorphisms is not considered in the study analyses. Second, as also stated in the manuscript, the detected copy number is limited. Also, focal CNVs that are very important in cancer can be missed due to limitation of the minimum CNV length and window size.

Changes: We added an analysis of the “Robustness of the CNV calling results” (section 2.9 in SupplementaryInformation.pdf), with a subsection analyzing the “Accuracy of polymorphism calling” (subsection 2.9.1). We consider the effect of 5% false positive polymorphism calls compared to our main results and 5% false negative polymorphism calls. We analyze the effect on detection thresholds as well as on CNV calling.
Additionally, we added a paragraph to our discussion on the detection of small CNVs by CopyDetective and limitations resulting from the detection thresholds.

We share the reviewer’s opinion that focal CNVs are very important in cancer and may be missed due to the detection thresholds determined and applied by CopyDetective. However, along with this limitation comes an advantage: users receive information on the minimum CNV length and the minimum cell fraction that can still be detected with their WES data, following CopyDetective’s algorithm that also considers user-defined sensitivity. Thus, it can be easily decided on whether the analyzed WES data is sufficient to detect the CNVs of interest or if additional experiments have to be performed.
Regarding limitations with respect to copy number, it would be great to extend our algorithm to detect CNVs of value 0 or 4. To do so, coverage would have to be evaluated in more detail. The coverage indicator of CopyDetective may serve as a starting point. However, to implement such an approach, we would need validated test data (matching WES samples + array information on validated CNVs) containing several CNVs with a value of 0 or 4. Despite extensive search we could not find such a data set yet. The 100 samples we analyzed in our manuscript contain only 3 CNVs of value 0 (data set 3) and no CNVs of value 4 or higher. Unfortunately, all CNVs of value 0 are located in regions where additional duplications (value 3) were detected, which complicates evaluation of coverage and development of an extension of our algorithm. We will keep on searching for an appropriate data set and hope to develop an extension of our current algorithm shortly.

2. Lists of detected CNVs and CNV characteristics such as CNV length, start, end and CNV values are not given.

Changes: A list of all detected CNVs (raw and filtered output), including all characteristics determined by CopyDetective has been added. A comparison to Additional File 1, Tables S10-13 is also included. The list is now available as Additional file 2 (SupplementaryData1.xlsx).

3. Even though not a complex statistical or computational method is used, It is not easy to follow the method. I think it is partially due to the presentation. Better explanation of the method with more informative figures can be helpful.

Changes: We revised the description of our method, especially the first “Quality analysis”-step. We revised Figures 1-3 (esp. Figure 2), making them more informative and easier to follow.

Minor comments:

1. the fonts of texts in the figures are too small and are not readable.

Changes: We revised all Figures in the main manuscript and in Additional file 1 (SupplementaryInformation.pdf), increasing font size and improving readability.

2. In the "Quality analysis" section, authors explained 3 steps for quality analysis to determine thresholds of the CNV length and cell fractions (CFs). Authors need to add an explanation of why these quality control steps are important and how they effect the performance of CNV calling. How sensitive are the results to changes of the thresholds? How robust are the proposed method against these thresholds?

Changes: We added an analysis of the “Robustness of the CNV calling results” (section 2.9 in SupplementaryInformation.pdf), with a subsection on “Changing detection thresholds” (subsection 2.9.2). Starting from the thresholds that were determined to reach user-defined sensitivity (here: 0.95), we doubled and halved the values for the CNV length and the cell fraction. Results on CopyDetective’s performance are reported and discussed.

3. Page 3, first column, second paragraph. It is stated "For every CF, a case-control-pair is simulated". It is not clear what are simulated. Are short reads simulated? Or SNPs are inserted into the already aligned reads?

Changes: We revised the paragraph, adding more information on what is simulated by CopyDetective. Additionally, we revised Figure 2.

To keep run-time low, we do not simulate the actual short reads or add SNPs to the aligned reads, which would require additional variant calling. The important parameters that have to be simulated – as CopyDetective’s analysis is based on them – are coverage and VAF of each SNP.
We noticed that Figure 2A could be misleading with respect to simulation as reads with SNPs are visualized. That was not our intention. We just aimed at exemplifying CopyDetective’s simulation step. We revised Figure 2, hoping that the simulation step gets clearer now.

4. Page 3, first column, third paragraph. Authors need to elaborate more on the t-test. The test apply to what?

Changes: We revised the paragraph and added more information on the weighted t-test. We additionally revised Figure 2, adding details on the applied t-test to Figure 2A, including the precise hypotheses that are tested as well as the weights.

5. Page 3, first column, forth paragraph. The analysis is for WES data. In WES, sequencing data are available only for exonic regions which are about 2% of a genome. Can authors explain that how their data on CFs can be extended to whole genome when the information about the whole genome is missing. How a minimum CNV size can be confidently determined when there is no information about VAF and coverage for most part of the CNV region?

Changes: We added subsection 2.3.1 “Covering the whole genome with whole-exome data” to Additional file 1 (SupplementaryInformation.pdf), evaluating the possibilities to detect CNVs in the whole genome, while just analyzing WES data. Additionally, we added a paragraph to our discussion.

6. Page 3, first column, forth paragraph. It is not clear what the window size is for. And again the method is for WESE data. These kind of analysis are more appropriate for WGS data when a complete information of whole genome is available.

Changes: We revised the description of the window size in paragraph four and in Figure 2, clarifying how it is calculated and why it is necessary to define it. Additionally, we added a remark to the paragraph, stressing the fact that we are just analyzing polymorphisms in WES data, not in WGS data.

We share the reviewer’s opinion that WGS data is of great importance for detecting CNVs. It would be

very interesting to test CopyDetective on a WGS data set (matched samples with preferably high coverage + validated CNV information; unfortunately, we did not have such a set for testing). We would expect to observe even better results compared to the WES data we analyzed in our manuscript. As information on more polymorphisms would be available, lower detection thresholds could be applied. Yet, we observed that in several projects WGS data is not available. And if it is available, coverage is usually low (<30x) and no matching control sample is available. Thus, we think it is highly useful to have an algorithm that is able to tap the full potential of WES data, calling CNVs along with precise detection thresholds. Analysis of 100 samples shows that CopyDetective succeeds in reporting CNVs with coordinates and cell fractions close to biological truth, although we can just rely on WES data.

7. Page 3, second column. The section "CNV calling" starts with "Once the thresholds have been estimated, actual CNV calling is performed." But it is not stated how the thresholds are estimated.

Changes: We revised the paragraph. The thresholds are now mentioned by name. Additionally, we refer to the “quality analysis”-step, which now contains a more detailed description on how the thresholds are estimated.

8. In the Result section and in the supplementary material, it is stated that a detected CNV is called true if there is an overlap with the ground truth CNV. How much overlap is considered?

Changes: We added information to the result section and the supplementary information that any overlap (in theory even 1 bp) is considered.

We thought about applying a threshold for minimum overlap at first. However, in the end we opted against this possibility.
Most CNV calling tools that we consider for comparison (ExomeCNV, ExomeDepth, VarScan and CNVseq) report a high number of partly very short CNVs. If these calls are overlapping a true (long) variant, they would usually cover only a low percentage of the mutated region. Thus, they would be filtered out due to low overlap and sensitivity of the comparison tools would be erroneously low.
We want to show that CopyDetective performs better than the comparison approaches considering both sensitivity and PPV, even if we follow a most lenient approach for sensitivity calculation for all tools. As for CopyDetective we show (precise numbers now provided in SupplementaryData1.xlsx) that reported CNVs are characterized by high overlap with the validated CNVs.

9. Discussion and Conclusion, first paragraph, last sentence. What do the authors mean by "desire sensitivity"?

Changes: We revised the last sentence of the paragraph. Additionally, we revised the description of the detection thresholds and their relation to sensitivity in the methods section.

By desired (now: user-defined) sensitivity we mean that the user is able to define sensitivity, similar to sample size calculations for clinical trials, where the necessary number of patients is calculated to observe a present effect with sensitivity of e.g. 0.95. If sensitivity is supposed to be higher, more patients will have to be recruited. As for CopyDetective, higher thresholds would have to be applied.

10. Table 3 needs more explanation.

Changes: We moved Table 3 to the supplement (Additional file 1, Table S15), revising and simplifying the displayed information. Explanatory text was added. In the main manuscript, former Table 3 was replaced by new Figure 4, showing the quality values for true positive and false positive CNV calls in all four data sets. Additionally, we revised and extended the explanation of Figure 4.

11. I recommend the authors provide some information about the characteristics of the detected CNVs and the groud truth CNVs such as the length and the value of the copy numbers.

Changes: A list of all detected CNVs (raw and filtered output), including all characteristics determined by CopyDetective has been added. (characteristics: chromosome, start, end, variant, p value, estimated

cell fraction, standard deviation of the estimated cell fraction, number of evaluated polymorphisms, number of merged windows, quality, coverage indicator). A comparison to Additional File 1, Tables S10-14 is also included (characteristics: chromosome, start, end, type and value of the CNV, called by CopyDetective true/false, above detection thresholds, quality, estimated cell fraction). The list is now available as Additional file 2 (SupplementaryData1.xlsx).

12. In Table 2, the numbers of detected CNVs by the proposed method are lower than those of the other tools, while the sensitivities are higher. it suggests that the detected CNVs by the proposed method are very long covering many CNVs. Giving information about the overlap for calling a true CNV and the CNV lengths can be helpful. Also, if CNVs with different amplification or deletion labels compared to the ground truth CNV labels are consider as TRUE CNV, the evaluation results are not correct.

Changes: In addition to supplementary Figure S10A-C, we added information on the overlap (percentages and base pairs) to novel Additional file 2 (SupplementaryData1.xlsx).
We revised the caption of Table 2 and the description in the manuscript. CNVs with different amplification or deletion labels are not evaluated as true calls for the calculation of sensitivity, PPV or the F1 score. Just due to space reasons they are not reported in a separate column, but in the column of true positive calls, in brackets next to the number of actually true positive calls. In Additional file 2 (SupplementaryData1.xlsx) we marked them with the comment “False type”.
To simplify Table 2, we decided to remove true positive calls for CopyDetective (with and without correct CNV type) if the overlapping validated CNV is characterized by a CF and/or length below the detection thresholds. These calls can be found in Additional file 2, comment “truth below threshold”.

It is true that the CNVs detected by CopyDetective are relatively long. This is due to the applied threshold for window size and to the automatic merging step. Considering supplemental Figure S7 it can be observed that especially ExomeDepth and ExomeCNV (but also VarScan) tend to report a high number of partly very short CNVs. The validated truth, however, usually shows rather long CNVs.
We did not apply any self-coded merging steps to the tools evaluated for comparison. We followed their default pipelines as described in Additional file 1, section 1.4.
We understand the reviewer’s concerns that CNVs reported by CopyDetective could potentially cover many CNVs. However, detailed results that are now available as Additional file 2 show that this is not the case. In a vast majority of cases, the CNVs detected by CopyDetective match biological truth really well and do not cover more than one true CNV. In seven cases (two in set 1, five in set 2) CopyDetective splits one validated CNV to ≥2 distinct CNVs. Four out these seven cases consider different samples from the same patient. The split CNVs reported by CopyDetective stay stable, which may indicate that they are truly split mutations. Only in two cases (set 2) we observe one CNV call by CopyDetective covering two validated CNV calls. However, again the observation remains stable over both samples, which may indicate a true result reported by CopyDetective.
Regarding CNVs with different amplification or deletion labels, we are sorry for the misunderstanding. We revised the description of the table. Of course, these calls have not been counted as true positive calls. They neither affect calculation of sensitivity, nor PPV. Yet, these CNV calls pose a special case. Assuming we have a true deletion in 50% of the cells, CopyDetective will most likely report both a deletion in 50% and a duplication in 100% of the cells. The reported duplication should not be counted as a false positive CNV. It is just another explanation of the observed changes in VAF. But of course, it cannot be counted as a true positive CNV either. Therefore, we just report it separately, in brackets next to the actual true positive calls.

Reviewer: 2

Comments:
1. I found an issue in first step of presented method, where individual detection thresholds are determined.
If I understood correctly, for the case-control pair , incrasing number of heterozygous polymorphisms (from 1 to 100) for cell fractions 5%, 10%, ..., 100% (5% step), was evaluated for deletions and duplications, t-test was used. Evaluation was performed 500 times.
My question is: why these analyzes use simulation instead of analytical calculations. Having a random variable distribution, we are able to calculate parameters, like min_SNP, using analytical methods or distribution tables without simulation. In addition, our calculations are able to return exact fractions of cells (not with an accuracy of 5% as before), because we assume that the allele frequency variants

(VAFs) are linearly dependent on the cell fraction (CF).

Changes: We revised our algorithm and added a switch: the user may now choose between a simulation approach and an exact approach. Information on the exact approach was added to the methods section (quality analysis). An evaluation of the CNV calling results based on the exact approach can be found in Additional file 1, section 2.5 "CNV calling using the exact approach" (SupplementaryInformation.pdf).

The reviewer is of course correct that analytical calculations can be used to determine parameters like min_SNP. We decided to provide a simulation approach in the first place as run-time of this approach is usually much lower compared to an exact approach. Dependent on the number of simulations n that is chosen, the t-test has to be performed n-times for windows with 2 SNPs, n-times for windows with 3-SNPs etc. to estimate min_SNP. In case of an exact approach, assuming a dataset with m called SNPs, the t-test has to be performed (m-1)-times for windows with 2 SNPs, (m-2)-times for windows with 3-SNPs etc. In the data sets we evaluated, m is usually ~10,000 and thus much larger compared to n.
We now added the possibility to select the exact approach for analysis as an alternative to the simulation approach. We added a detailed analysis of the CNV calling results (simulation vs exact approach). This analysis revealed a very interesting observation: the VAFs of polymorphisms in our control samples are, on average, slightly lower than the expected value 0.5. Taking the exact VAFs as a basis for calculations, higher thresholds and a lower number of detectable CNVs can be observed. Working with an expected value of 0.5, however, detection thresholds most comparable to the simulation approach are calculated. The updated version of CopyDetective contains an additional switch for the exact approach: a user may choose between using the exact VAFs of polymorphisms in the control samples or working with an expected value of 0.5.
Regarding the accuracy of 5% for CF: we revised the description of the algorithm in our methods section and in the supplement. Of course, CopyDetective can also work with an accuracy of 1%. A step-size of 5% was just chosen as a default setting as we think it is a fair compromise between accuracy and run-time.

2. I found the typo: 'if if' in page 6, column 1, line 7 from bottom.

Changes: Thank you for noticing. We corrected it.

Editor

Comment:
Please register any new software application in the bio.tools and SciCrunch.org databases to receive RRID (Research Resource Identification Initiative ID) and biotoolsID identifiers, and include these in your manuscript.

Changes: We registered the CopyDetective in the bio.tools and SciCrunch.org databases. RRID and biotoolsID were added to the main manuscript (section: Availability of source code and requirements).

Close
