# Supplementary material for: CopyDetective: Detection threshold–aware copy number variant calling in whole-exome sequencing data
Source: Gigascience. 2020 Nov 2;9(11):giaa118. doi: 10.1093/gigascience/giaa118 (PMC7604644; doi:10.1093/gigascience/giaa118)
Supplement: giaa118_Response_to_Reviewer_Comments_Revision_1 [file giaa118_response_to_reviewer_comments_revision_1.pdf]

Author's Response To Reviewer Comments

Close

There were no more reviewer comments to be addressed.

Close
