# Supplementary material for: CopyDetective: Detection threshold–aware copy number variant calling in whole-exome sequencing data
Source: Gigascience. 2020 Nov 2;9(11):giaa118. doi: 10.1093/gigascience/giaa118 (PMC7604644; doi:10.1093/gigascience/giaa118)
Supplement: giaa118_Reviewer_1_Report_Original_Submission — Sheida Nabavi -- 7/3/2020 Reviewed [file giaa118_reviewer_1_report_original_submission.pdf]

Reviewer Report

Title: CopyDetective: Detection Threshold Aware CNV Calling in WES Data

Version: Original Submission **Date:** 7/3/2020

Reviewer name: Sheida Nabavi

Reviewer Comments to Author:

The manuscript proposed a method to call somatic copy number variations (CNVs) for whole exome sequencing (WES) data. It introduced an interesting idea of determining individual detection thresholds for every sample. The proposed method first, examines the quality of candidate CNVs in terms of coverages of tumor and match normal samples, CNV length and CNV value. Based on these quality analyses, the method automatically defines specific thresholds for calling CNVs for each individual sample. The CNVs are called using variant allele frequencies by comparing heterozygous polymorphisms in matching case-control samples and the fraction of affected cells for every CNV. The weighed t-test is applied to the cell fraction data to call CNVs. Then, the method merges or filters CNV regions by considering the coverage and SNP information, . The authors compared the performance of their method with those of five commonly used CNV detection methods using 4 real datasets with total number of 100 samples.

The paper is written well and the proposed method is comprehensively evaluated. I have the following comments.

Major comments

-My main concern is that calling CNV based on allelic frequency information can impose some limitations. First, the method would be dependent on the accuracy of the variant calling methods. How robust is the proposed method against variant calling errors in calling SNPs? The accuracy of the polymorphisms is not considered in the study analyses. Second, as also stated in the manuscript, the detected copy number is limited. Also, focal CNVs that are very important in cancer can be missed due to limitation of the minimum CNV length and window size.

- Lists of detected CNVs and CNV characteristics such as CNV length, start , end and CNV values are not given.

- Even though not a complex statistical or computational method is used, It is not easy to follow the method. I think it is partially due to the presentation. Better explanation of the method with more informative figures can be helpful.

Minor comments

- the fonts of texts in the figures are too small and are not readable.

- In the "Quality analysis" section, authors explained 3 steps for quality analysis to determine thresholds of the CNV length and cell fractions (CFs). Authors need to add an explanation of why these quality control steps are important and how they effect the performance of CNV calling. How sensitive are the results to changes of the thresholds? How robust are the proposed method against these thresholds?

- Page 3, first column, second paragraph. It is stated "For every CF, a case-control-pair is simulated". It is not clear what are simulated. Are short reads simulated? Or SNPs are inserted into the already aligned

reads?
- Page 3, first column, third paragraph. Authors need to elaborate more on the t-test. The test apply to what?
- Page 3, first column, forth paragraph. The analysis is for WES data. In WES, sequencing data are available only for exonic regions which are about 2% of a genome. Can authors explain that how their data on CFs can be extended to whole genome when the information about the whole genome is missing. How a minimum CNV size can be confidently determined when there is no information about VAF and coverage for most part of the CNV region?
- Page 3, first column, forth paragraph. It is not clear what the window size
is for. And again the method is for WESE data. These kind of analysis are more appropriate for WGS data when a complete information of whole genome is available.
- Page 3, second column. The section "CNV calling" starts with "Once the thresholds have been estimated, actual CNV calling is performed." But it is not stated how the thresholds are estimated.
- In the Result section and in the supplementary material, it is stated that a detected CNV is called true if there is an overlap with the ground truth CNV. How much overlap is considered?
- Discussion and Conclusion, first paragraph, last sentence. What do the authors mean by "desire sensitivity"?
- Table 3 needs more explanation.
- I recommend the authors provide some information about the characteristics of the detected CNVs and the groud truth CNVs such as the length and the value of the copy numbers.
- In Table 2, the numbers of detected CNVs by the proposed method are lower than those of the other tools, while the sensitivities are higher. it suggests that the detected CNVs by the proposed method are very long covering many CNVs. Giving information about the overlap for calling a true CNV and the CNV lengths can be helpful. Also, if CNVs with different amplification or deletion labels compared to the ground truth CNV labels are consider as TRUE CNV, the evaluation results are not correct.

Level of Interest

Please indicate how interesting you found the manuscript: Choose an item.

Quality of Written English

Please indicate the quality of language in the manuscript: Choose an item.

Declaration of Competing Interests

Please complete a declaration of competing interests, considering the following questions:

- Have you in the past five years received reimbursements, fees, funding, or salary from an organisation that may in any way gain or lose financially from the publication of this manuscript, either now or in the future?

- Do you hold any stocks or shares in an organisation that may in any way gain or lose financially from the publication of this manuscript, either now or in the future?
- Do you hold or are you currently applying for any patents relating to the content of the manuscript?
- Have you received reimbursements, fees, funding, or salary from an organization that holds or has applied for patents relating to the content of the manuscript?
- Do you have any other financial competing interests?
- Do you have any non-financial competing interests in relation to this paper?

If you can answer no to all of the above, write 'I declare that I have no competing interests' below. If your reply is yes to any, please give details below.

'I declare that I have no competing interests'

I agree to the open peer review policy of the journal. I understand that my name will be included on my report to the authors and, if the manuscript is accepted for publication, my named report including any attachments I upload will be posted on the website along with the authors' responses. I agree for my report to be made available under an Open Access Creative Commons CC-BY license (http://creativecommons.org/licenses/by/4.0/). I understand that any comments which I do not wish to be included in my named report can be included as confidential comments to the editors, which will not be published.

Choose an item.

To further support our reviewers, we have joined with Publons, where you can gain additional credit to further highlight your hard work (see: https://publons.com/journal/530/gigascience). On publication of this paper, your review will be automatically added to Publons, you can then choose whether or not to claim your Publons credit. I understand this statement.

Yes Choose an item.
